# Stratification of amyotrophic lateral sclerosis patients: a crowdsourcing approach

**DOI:** 10.1038/s41598-018-36873-4

**Published:** 2019-01-24

**Authors:** Robert Kueffner, Neta Zach, Maya Bronfeld, Raquel Norel, Nazem Atassi, Venkat Balagurusamy, Barbara Di Camillo, Adriano Chio, Merit Cudkowicz, Donna Dillenberger, Javier Garcia-Garcia, Orla Hardiman, Bruce Hoff, Joshua Knight, Melanie L. Leitner, Guang Li, Lara Mangravite, Thea Norman, Liuxia Wang, The ALS Stratification Consortium, Rached Alkallas, Catalina Anghel, Jeanne Avril, Jaume Bacardit, Barbara Balser, John Balser, Yoav Bar-Sinai, Noa Ben-David, Eyal Ben-Zion, Robin Bliss, Jialu Cai, Anatoly Chernyshev, Jung-Hsien Chiang, Davide Chicco, Bhavna Ahuja Nicole Corriveau, Junqiang Dai, Yash Deshpande, Eve Desplats, Joseph S. Durgin, Shadrielle Melijah G. Espiritu, Fan Fan, Philippe Fevrier, Brooke L. Fridley, Adam Godzik, Agnieszka Golińska, Jonathan Gordon, Stefan Graw, Yuelong Guo, Tim Herpelinck, Julia Hopkins, Barbara Huang, Jeremy Jacobsen, Samad Jahandideh, Jouhyun Jeon, Wenkai Ji, Kenneth Jung, Alex Karanevich, Devin C. Koestler, Michael Kozak, Christoph Kurz, Christopher Lalansingh, Thomas Larrieu, Nicola Lazzarini, Boaz Lerner, Wojciech Lesinski, Xiaotao Liang, Xihui Lin, Jarrett Lowe, Lester Mackey, Richard Meier, Wenwen Min, Krzysztof Mnich, Violette Nahmias, Janelle Noel-MacDonnell, Adrienne O’Donnell, Susan Paadre, Ji Park, Aneta Polewko-Klim, Rama Raghavan, Witold Rudnicki, Ehsan Saghapour, Jean-Bernard Salomond, Kris Sankaran, Dorota Sendorek, Vatsal Sharan, Yu-Jia Shiah, Jean-Karl Sirois, Dinithi N. Sumanaweera, Joseph Usset, Yeeleng S. Vang, Celine Vens, Dave Wadden, David Wang, Wing Chung Wong, Xiaohui Xie, Zhiqing Xu, Hsih-Te Yang, Xiang Yu, Haichen Zhang, Li Zhang, Shihua Zhang, Shanfeng Zhu, Jinfeng Xiao, Wen-Chieh Fang, Jian Peng, Chen Yang, Huan-Jui Chang, Gustavo Stolovitzky

**Affiliations:** 1 Icahn School of Medicine at Mount Sinai, New York, NY USA; 2 Teva Pharmaceuticals, Netanyah, Israel; 3 Prize4Life, Haifa, Israel; 4 IBM Research, Yorktown Heights, NY USA; 5 Massachusetts General Hospital, Boston, MA USA; 6 Information Engineering Department, University of Padova, Padova, Italy; 7 University of Turin, Turin, Italy; 8 Pompeu Fabra University, Barcelona, Spain; 9 Institute of Neuroscience, Trinity College, Dublin, Ireland; 10 Sage Bionetworks, Seattle, WA USA; 11 Accelerating NeuroVentures, Boston, MA USA; 12 Amazon, Seattle, WA USA; 13 Zillow, Seattle, WA USA; 14 Department of Computer Science, University of Illinois at Urbana-Champaign, Champaign, IL USA; 15 Department of Information and Learning Technology, National University, Tainan City, Taiwan; 16 Department of Computer Science and Information Engineering, National University, Tainan City, Taiwan; 17 Faculty of Information Technology, Monash University, Clayton, Australia; 18 Department of Human Genetics, McGill University, Montreal, Canada; 19 Ontario Institute for Cancer Research (OICR), Toronto, Canada; 20 Departement d’Economie Ecole Polytechnique, Paris, France; 21 Interdisciplinary Computing and Complex BioSystems (ICOS) research group, Newcastle University, Tyne, UK; 22 Veristat Inc, Southborough, MA USA; 23 Medical Research, Kfar Malal, Israel; 24 Department of Computer Science, Ben-Gurion University of the Negev, Beersheba, Israel; 25 Department of Industrial Engineering and Management, Ben-Gurion University of the Negev, Negev, Israel; 26 Analytica Laboratories, Hamilton, New Zealand; 27 Department of Computer Science and Information Engineering, National Cheng Kung University, Tainan City, Taiwan; 28 Princess Margaret Cancer Centre, Toronto, Ontario Canada; 29 Department of Biostatistics, University of Kansas Medical Center, Kansas City, KS USA; 30 MIT, Department of Mathematics, Cambridge, MA USA; 31 Perelman School of Medicine, University of Pennsylvania, Philadelphia, PA USA; 32 Centre de Recherche en Economie et Statistique (CREST), Paris, France; 33 Department of Biostatistics and Bioinformatics, Moffitt Cancer Center, Tampa, FL USA; 34 Program on Bioinformatics and Systems Biology, Sanford Burnham Prebys Medical Discovery Institute, La Jolla, CA USA; 35 Institute of Informatics, University of Białystok, Ciołkowskiego, Białystok, Poland; 36 Department of Engineering, University of Cambridge, Cambridge, UK; 37 RTI International, Research Triangle Park, NC Triangle Park, USA; 38 KU Leuven, Department of Public Health and Primary Care, Kortrijk, Belgium; 39 Department of Biochemistry, University of Colorado, Boulder, CO USA; 40 Origent Data Sciences, Inc., Vienna, VA USA; 41 National Center for Mathematics and Interdisciplinary Sciences, Academy of Mathematics and Systems Science, Chinese Academy of Sciences, Huairou, China; 42 Stanford University, Center for Biomedical Informatics Research, Stanford, CA USA; 43 Department of Statistics, Tel-Aviv University, Tel Aviv-Yafo, Israel; 44 Helmholtz Zentrum München, Institute of Health Economics and Health Care Management, Munich, Germany; 45 Department of Bioinformatics, University of Bialystok, Ciolkowskiego, Bialystok, Poland; 46 Shanghai Key Lab of Intelligent Information Processing and School of Computer Science, Fudan University, Shanghai, China; 47 Microsoft Research New England, Cambridge, MA USA; 48 School of Computer, Wuhan University, Wuhan, China; 49 Computational Centre, University of Białystok, Ciołkowskiego, Białystok, Poland; 50 Children’s Mercy Hospital, Kansas City, MO USA; 51 LinkedIn, Sunnyville, CA USA; 52 Interdisciplinary Centre for Mathematical and Computational Modelling, University of Warsaw, Pawińskiego, Warsaw, Poland; 53 Department of Biomedical Engineering, School of Advanced Technologies in Medicine, Isfahan University of Medical Sciences, Isfahan, Iran; 54 Ceremade Universite Paris-Dauphine, Paris, France; 55 Université Paris-Est, Laboratoire d’Analyse et de Mathématiques Appliquées, Créteil, France; 56 Stanford University, Department of Statistics, Stanford, CA USA; 57 Stanford University, Department of Electrical Engineering, Stanford, CA USA; 58 Department of Computer Science and Engineering, University of Moratuwa, Moratuwa, Sri Lanka; 59 Department of Computer Science, University of California, Irvine, CA USA; 60 Paul G. Allen School of Computer Science & Engineering, University of Washington, Seattle, WA USA; 61 Department of Computer Science, City University of Hong Kong, Hong Kong, China; 62 Dept of Computer Science, Bren School of Information and Computer Sciences, University of California, Irvine, CA USA; 63 University of Pennsylvania, Philadelphia, PA USA; 64 University of Maryland, Baltimore, MD USA; 65 Centre for Computational System Biology, ISTBI, Fudan University, Shanghai, China

## Abstract

Amyotrophic lateral sclerosis (ALS) is a fatal neurodegenerative disease where substantial heterogeneity in clinical presentation urgently requires a better stratification of patients for the development of drug trials and clinical care. In this study we explored stratification through a crowdsourcing approach, the DREAM Prize4Life ALS Stratification Challenge. Using data from >10,000 patients from ALS clinical trials and 1479 patients from community-based patient registers, more than 30 teams developed new approaches for machine learning and clustering, outperforming the best current predictions of disease outcome. We propose a new method to integrate and analyze patient clusters across methods, showing a clear pattern of consistent and clinically relevant sub-groups of patients that also enabled the reliable classification of new patients. Our analyses reveal novel insights in ALS and describe for the first time the potential of a crowdsourcing to uncover hidden patient sub-populations, and to accelerate disease understanding and therapeutic development.

## Introduction

Amyotrophic lateral sclerosis (ALS) is a neurodegenerative disorder which causes the death of motor neurons that control voluntary muscles, leading to progressive muscle weakening and paralysis and death within an average of only 3–5 years from symptom onset^1^. Existing therapeutic options extend survival by merely a few months^2,3^. One of the biggest challenges today is the well-established heterogeneity of ALS^1,4^, with patients displaying widely different patterns of disease manifestation and progression, and genetic analyses suggesting heterogeneity of the underlying biological mechanisms^5–8^. This heterogeneity has detrimental effects on clinical trial planning and interpretation^3^, on attempts to understand disease mechanisms, and on clinical care, as it increases uncertainty about prognosis and optimal treatment. Thus, successfully stratifying ALS patients into clinically meaningful sub-groups can be of great value for advancing the development of effective treatments and achieving better care for ALS patients.

Early classification systems for ALS patients were based on clinical presentation of the disease and were intended for ascertainment of an ALS diagnosis, but had limited capacity to predict disease prognosis or suggest underlying disease mechanisms^4,9,10^. More recent attempts towards ALS patient classification focused on prediction of clinical outcomes but were often limited by small sample sizes and sparse clinical information^11–14^. In the current study, we sought to use the power of state of the art machine learning algorithms applied to a large-scale, diverse and clinically detailed database of ALS patients to uncover and characterize clinically relevant subpopulations of ALS patients.

Two complementary data sources were used in this challenge. The first was data from ALS national or regional registers from Ireland and the Piemonte and Valle d’Aosta region in Italy, representing ALS community data collected as part of standard clinical visits. The second dataset was ALS clinical trial data, from the Pooled Resource Open-Access ALS Clinical Trials platform (PRO-ACT, www.ALSdatabase.org, developed by Prize4Life and Massachusetts General Hospital), an open-access database containing harmonized and de-identified data of over 10,000 ALS patients from 23 completed clinical trials^15^.

The PRO-ACT database was previously used for a crowdsourcing computational challenge: The 2012 DREAM-Phil Bowen ALS Prediction Prize4Life challenge (The ALS Prediction Challenge)^16^. This Challenge invited participants to develop computational algorithms that could predict ALS disease progression, with the best performing algorithms achieving a prediction accuracy that would allow a 20% reduction in the number of patients needed for a trial^16^ and are currently being tested in real-world clinical trial applications^17,18^.

Building upon the success of the earlier prediction challenge, the 2015 DREAM ALS Stratification Prize4Life Challenge (The ALS Stratification Challenge) sought to extend the scope of prediction algorithms by inviting participants to stratify the ALS patient population into distinct clusters and develop separate predictive models for each subpopulation. The ALS Stratification Challenge included both disease progression and survival as predicted outcome measures and used both clinical trial and community-based registries data. A prize of $28,000, collected through a crowdfunding effort, was offered to best performing algorithms (see Supplementary material part 1 and 2 for detailed description of both datasets and the challenge description as given to participants, respectively).

In this publication we describe the results of the challenge including analysis of the best performing algorithms’ performance and methods, as well as novel methods to uncover the patient sub-populations, their statistical significance and relative importance of different predictive features obtained from cross-model assessment.

## Results

### Challenge outline

The ALS Stratification Challenge was developed and ran through a collaboration between the nonprofit organizations Dialogue for Reverse Engineering Assessments and Methods initiative (DREAM, http://dreamchallenges.org/) and Prize4Life (www.prize4life.org.il) using the Sage Bionetworks Synapse platform (www.synapse.org). The challenge ran between June and October 2015 and drew 288 registrants, eventually leading to final submissions by 30 teams (88 individual participants) from 15 countries (see Supplementary material part 3 for participant survey).

The challenge was based on two datasets: (1) ALS clinical trials data collected through the PRO-ACT database, and (2) community-based ALS clinical data collected through ALS registries. Both datasets contained longitudinally sampled demographic and clinical information with some additional genetic (specific mutation) and family history data in the registries and detailed laboratory tests in PRO-ACT (See Supplementary material part 1 and 2). We solicited predictions across four sub-challenges, namely of (1) disease progression or (2) survival probability using PRO-ACT data and (3) disease progression or (4) survival probability using ALS registries data.

Challenge participants were asked to use patients’ data from the first 3 months of records to predict disease progression at 12 months or probability of survival at 12, 18 & 24 months. Disease progression was defined as the slope of the ALS Functional Rating Scale (ALSFRS or ALSFRS-R) between 3 and 12 months (see online methods). To avoid overfitting of algorithms^19^, data from the publicly available PRO-ACT database was used as the training set, and a separate set of data from six additional clinical trials, which were not previously publicly available, was used for validation. The registry data, which was never before made publicly available, was divided randomly (split evenly across the two registries) into training and validation sets.

**Figure 1:**
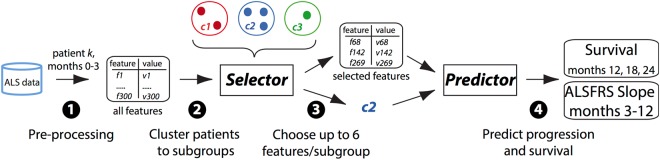
Outline of algorithms design. Algorithms used either PRO-ACT or ALS registries data, and first (1) applied various data pre-processing and imputation methods. Next, (2) algorithms could cluster the patient population into any number of sub-groups and (3) select the most informative features for each cluster (up to a maximum of 6 features). Then (4) a “predictor” component had to use values of the selected features to predict either disease progression or survival for any given patient. In the scoring of the challenge, the algorithms made predictions for patients that were not part of the original datasets available for algorithms training, and the accuracy of these predictions was assessed.

The challenge introduced an additional requirement that predictions are limited in the number of used clinical features (Fig. 1). The requirement for limiting the number of features was highlighted by our clinical advisors to facilitate the application of predictive algorithms in natural clinical setting^13^. A preliminary analysis indicated the benefit from clustering (in terms of improved prediction accuracy) tends to increase when the number of features is restricted, and this effect plateaued at around 6 features (see Supplementary material part 4). Thus, participants were asked to write algorithms that first selected the most informative features (up to 6 features), and in a second step used only the data from these selected features to make progression or survival predictions (Fig. 1).

To enforce the separation between these steps we required participants to implement their algorithms in Docker containers (https://docs.docker.com) executed on the IBM Z cloud (https://www.ibm.com/it-infrastructure/z/capabilities/enterprise-security). Algorithms were thus run in a secured environment where the participants could not see the validation data or other jobs.

### Comparative assessment of prediction methods

**Figure 2:**
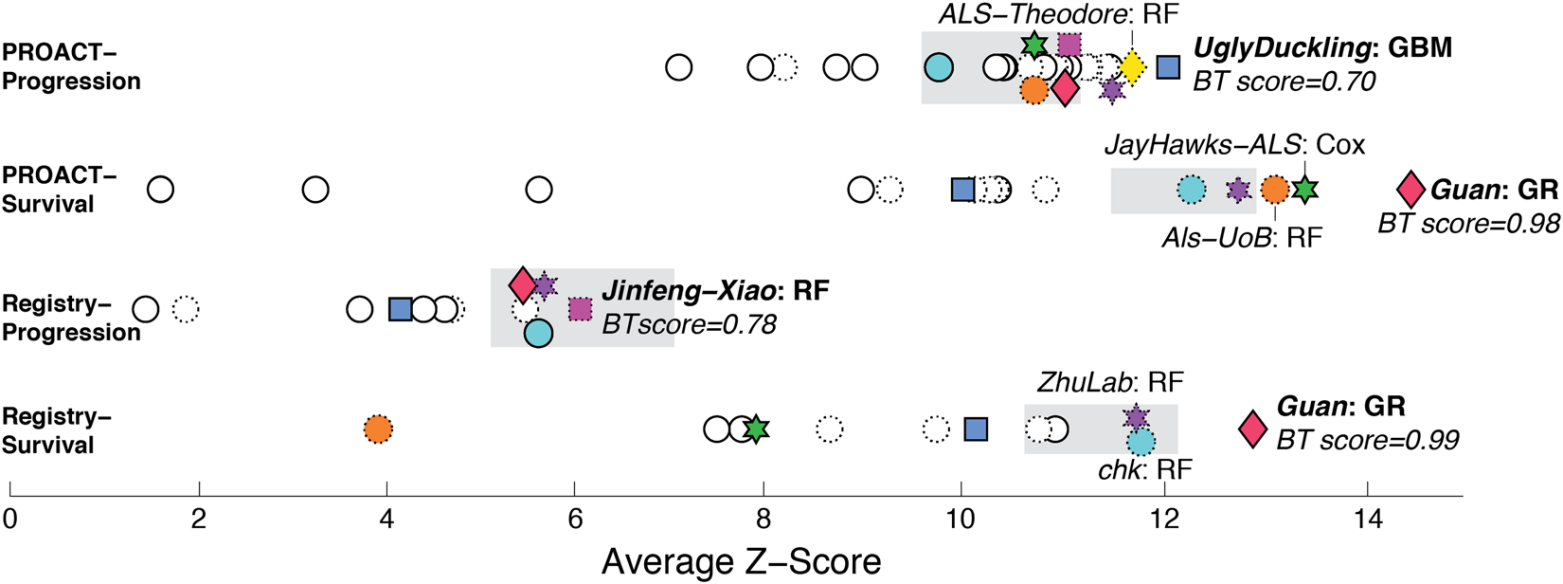
Overview of the performance of submitted and baseline algorithms across the four sub-challenges. Submissions were assessed by Z-scores combining RMSD, concordance index and Pearson’s correlation (see online methods for details on validation and testing). Performance was also compared to two baseline algorithms which were based on the top performing prediction algorithms submitted to the 2012 ALS prediction challenge, adapted to the requirements of the new challenge (see Supplementary material part 4). Grey boxes denote the performance of the best-performing baseline algorithm (left and right boundary of the box represent intervals of its performance ± the bootstrapped standard deviation). Teams that achieved the top three scores in any sub-challenge are indicated by colored symbols and shown by the same symbol in all sub-challenges. The BT score (right side of the figure) denotes the percentage of bootstrap samples where the top ranking team outperformed the second ranking team. The underlying method is indicated (RF = random forest, GBM = generalized boosting model, Cox = Cox model, GR = Gaussian regression). Submissions based on random forests, the most frequently used method, are denoted by symbols with dashed outlines.

In general, the top performing teams in each sub-challenge (except for the registry progression sub-challenge) significantly outperformed the best baseline algorithm (Fig. 2, See Supplementary material part 5 for full results). Random forest was by far the most commonly used prediction method, with overall very good results, scoring among the first three ranks in each subchallenge (Fig. 2). It was the method used by the best performing team in the registry progression sub-challenge and by most algorithms ranked between the 2nd and 8th places across all four sub-challenges (Fig. 2). Another successful method included a Gaussian process regression model with an arithmetic mean kernel, which was the best performing for the two survival challenges but did not perform so well for the progression challenges.

The performance of the top teams, as was observed in other crowdsourcing challenges^20,21^, varied substantially even if the same machine learning method was used, and depended to a large extend on data pre-processing, especially feature selection and representation of time-resolved features. Here, the best performing teams represented time-resolved information by a combination of simple summary statistics (for instance minimum, maximum and average of the feature values). For feature selection, the best teams evaluated the contribution of the complete set of selected features (as opposed to selecting them one by one), for example through evaluating sets of features by their combined information gain or by aggregating their weight along the paths of all trees in a random forest.

Survival predictions deserve special consideration, as one team substantially out-performed other participants in both sub-challenges. Survival predictions are particularly challenging due to the right-censored outcome variable survival time: data can be terminated by either patient death or by trial drop out. The standard Cox proportional hazards model, routinely used to explore the dependency between clinical features and survival, ignores such censored cases. In the current challenge, Yuanfang Guan generalized this right censored problem via a novel strategy GuanRank by complete ranking of training examples regardless of the censoring status, enabling GuanRank to be built-in to any base-learners (in this case, Gaussian Process Regression)^22^. This defined the outcome variable more precisely, which led to a more adequate training of regression models (here: Gaussian process models) explaining the algorithm’s superior prediction performance. Indeed, the strategy outperformed a standard Cox model by 20% accuracy^22^, and outperformed the respective second best team in 98.4% and 99.4% of the bootstrap samples (in the PROACT and registry subchallenges, respectively, compare Fig. 2).

Notably, for the sub-challenges running on the PRO-ACT database, the best performing algorithms significantly outperformed the winning method from the first challenge^16^. This is even more noteworthy given that the validation set was not randomly divided from the training set, but actually included the more difficult and realistic criteria of application of the algorithms to a data comprising of six new never used before trials.

### Predictive clinical features

**Figure 3:**
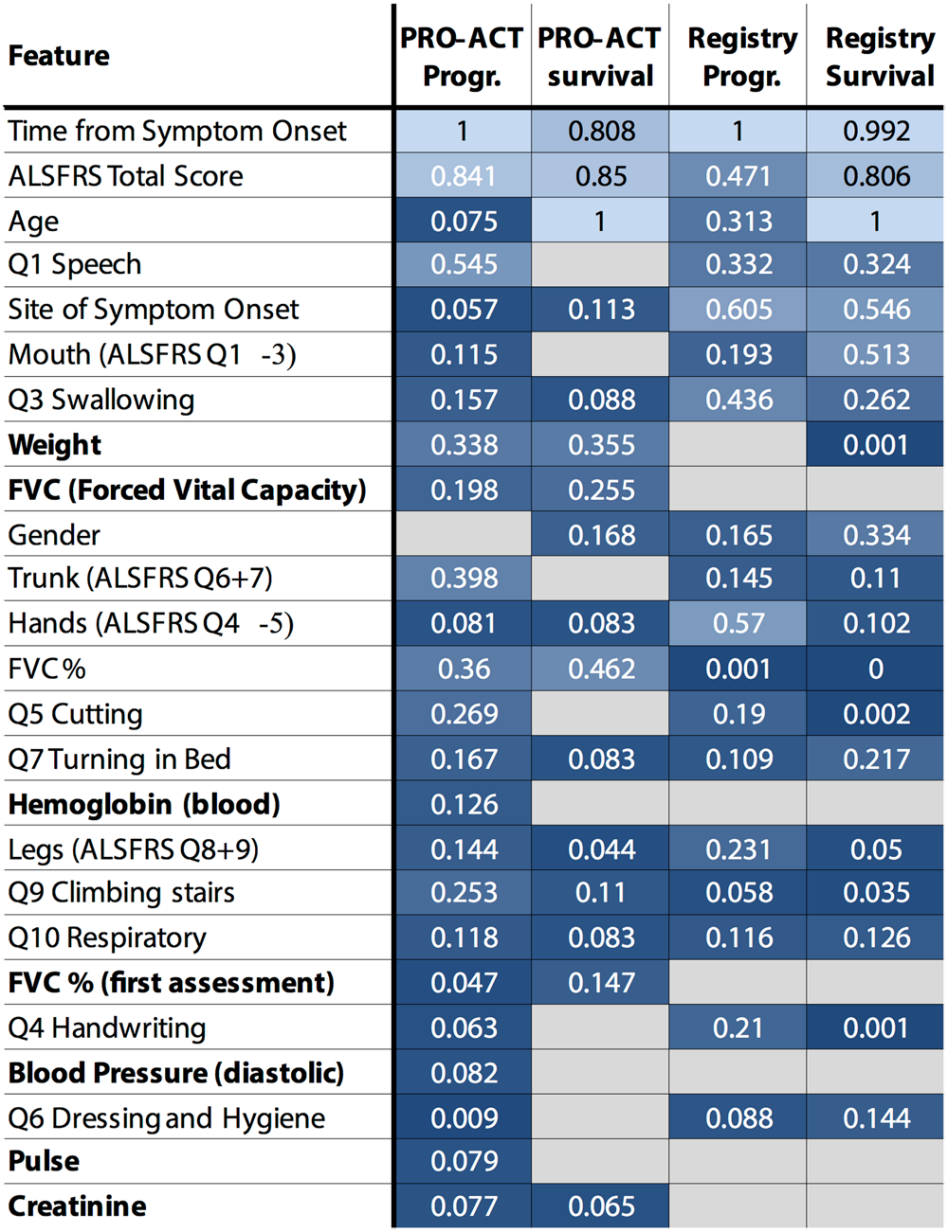
Overview of the features most frequently used by the algorithms within and across subchallenges. For each subchallenge, we assessed the number of times each feature was used for prediction across all submitted algorithms (shown as probability. The features are ranked-ordered by this probability, averaged across all sub-challenges where darker colors denote lower probabilities). Cases where a given feature was not used at all for a given sub-challenge are shown in grey (probability 0). Features that are recommended to be assessed by clinicians more often to aid prognosis are marked in bold.

The challenge’s requirement of using only up to six features for prediction encouraged participants to identify the most informative features for prediction of disease progression or survival (Fig. 3). The most frequently used features across all sub-challenges were those that are well described in the literature as being strongly related to ALS prognosis: time from disease onset and total ALSFRS score^23–25^. Age and gender were more informative for predicting survival rather than disease progression, in line with literature^22–24,26–28^. While age and gender were generally predictive in this data as well, they were not specifically more predictive for one particular subgroup of patients, and therefore less relevant for stratification. Bulbar function is also known to be particularly informative for clinical outcome prediction^24–26^ and ALSFRS questions 1 and 3 (bulbar functions) were selected more frequently compared to any other functional domains across all sub-challenges. Interestingly, ALSFRS question Q2 (salivation) was rarely used, in line with previous works indicating this question might be less well correlated with total ALSFRS scores and/or disease progression, due to effective available treatment options^29^.

Data availability was another important factor for feature selection. For example, while weight or BMI, known to correlate with ALS prognosis^30,31^, was frequently selected for predictions for the PRO-ACT dataset, it was recorded in only < 10% of the cases in the registry data, making it unusable for predictions (Fig. 3). Similar potential predictive benefit of features was observed with respect to features evaluating breathing capacity, which is the main cause of death for ALS patients^32^, but not routinely collected for registry patients. This suggests that clinicians could potentially improve their insight into individual patient prognosis by incorporating a few rather accessible measures into routine clinical monitoring (relevant features are bolded in Fig. 3). Indeed, since the challenge ran, clinics involved in the Italian registry used in this challenge have been careful to add measurements of patient weight, BMI and respiratory functions.

### Clinically relevant patient clusters

In this challenge we used a crowdsourcing approach to explore different stratification schemes for ALS patients and use them to identify clinically significant patient sub-populations. The main goal of stratification, however, was not necessarily to impact prediction accuracy, and indeed we did not observe any consistent advantage (in terms of improved prediction accuracy) for clustering in any of the evaluation metrics. Instead, as described in the following, we developed novel strategies utilizing this crowdsourcing approach to reveal, across a large variety of methods, consistent clusters of patients, to support understanding the ALS pathology, improving clinical care and planning better, more efficient clinical trials. This sort of analysis can only be accomplished in the context of a large communal effort that allows comparison of independently designed algorithms working on sufficiently large datasets, as was the case in this challenge.

**Figure 4:**
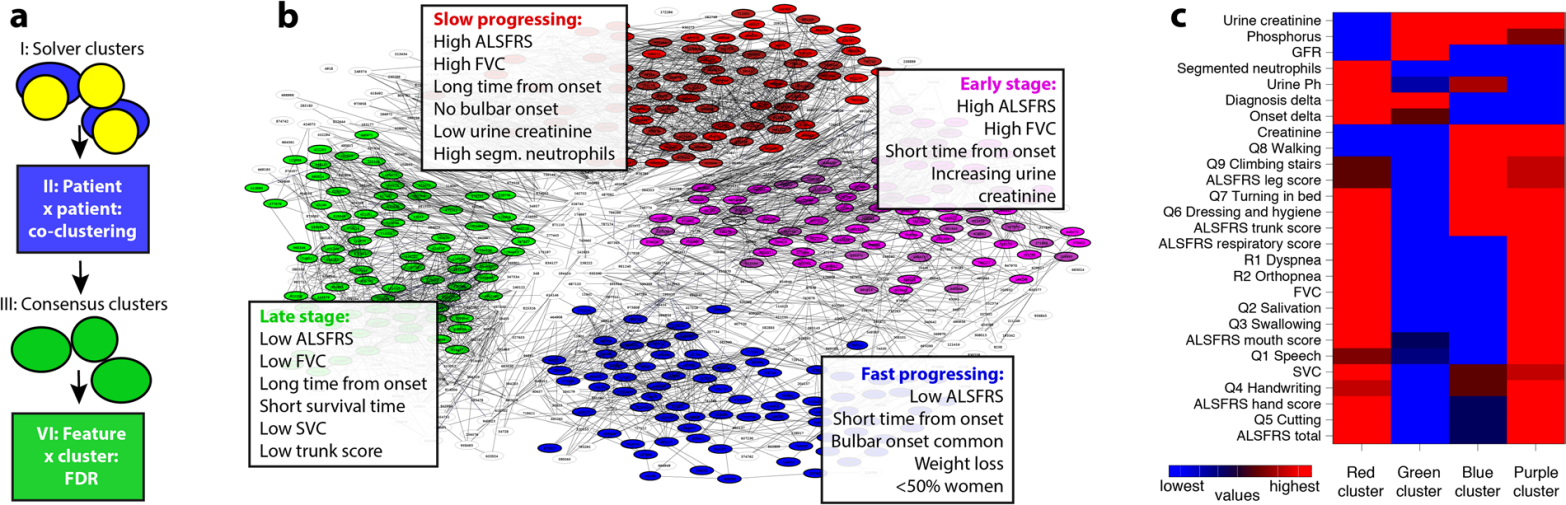
Overview of the consensus clustering. (**a**) Outline of consensus clustering method: the tendency of patients to co-cluster was assessed across cluster-sets generated independently by the different solvers (I). The resulting connectivity matrix (II) was then used as input for obtaining consensus clusters (III) by k-means. Finally, False discovery rates (FDRs) were estimated by ANOVA (IV) on 100 randomized datasets to assess, which features were differentially distributed between consensus clusters, see online methods and Supplementary material part 6. (**b**) Graph-based clustering of the connectivity matrix for the PRO-ACT progression sub-challenge. Nodes in the graph represent patients and are colored based on their k-means cluster, if they correspond to the 50% of “core” patients closest to their respective cluster centroid. Edges denote pairs of patients with a significant chance of being co-clustered by solvers. (**c**) We compared features (names starting with Q/R are ALSFRS component scores from the original or revised scale) between pairs of clusters (columns in heatmap) by t-tests/FDRs. Different colors within heatmap rows indicate values that are significantly different between clusters (FDR < 5%) on the scale from the lowest (blue) to highest (red). Notable results are listed explicitly in Panel b.

In comparison, individual clustering methods led to heterogeneous results and the numbers of clusters per method varied from 2 to more than 100. Integrating clustering across a large variety of methods on the other hand, revealed a small set of discrete consensus clusters (Fig. 4, see online methods). In both sub-challenges based on PRO-ACT, patients within consensus clusters were significantly more strongly connected than expected by chance (pairs of patients co-clustered significantly with FDR < 5% are depicted as edges in Fig. 4b). The stronger clustering effect in the PRO-ACT vs. the registry sub-challenges is likely a reflection of sample size (10,000 + vs. ~1,500 patients, respectively).

Overall, consensus clusters could be broadly regarded as classifying patients as slow progressing (“red”), fast progressing (“blue”), early stage (“purple”) or late stage (“green”). We chose to focus on clinical characteristics of the PRO-ACT progression sub-challenge, since these consensus clusters reached the highest level of statistical significance, but similar clusters were found across all sub-challenges.

In order to demonstrate how the identified clusters can be utilized in a clinical setup, we also examined whether new patients can be assigned into their respective cluster reliably. By using the most significant 20 features (FDR < 0.01%) we were able to re-associate 84% of the core patients (the patients closest to their respective cluster center, compare Fig. 4). Note, that the consensus clustering that was derived solely from the participants clustering, while the re-clustering is solely based on the values of the clinical features (see online methods). In other words, based on the commonalities of the methods generated by separate teams we were able to uncover a network of sub-groups that can be recreated with good success, strongly suggesting clinical usability in a field where patient stratification is pivotal to development of an effective ALS treatment.

In the following, we characterize these clusters and describe the relationship between patient strata and the most discriminative clinical features: One cluster, the “red” or “slow progressing” cluster included patients who, despite having experienced symptoms for a relatively long time (2.2 years from disease onset, on average) still maintained relatively high functional capabilities (average ALSFRS-R scores of 40.25 at the beginning of the clinical observation period), with functional impairments mostly limited to limbs and little bulbar or respiratory involvement. Accordingly, these patients had slow disease progression (annual average loss of −0.48 on the ALSFRS-R scale). These are the patients with the best prognosis for ALS and therefore merit closer investigation, as they might hold clues for clinical or biological features underlying enhanced resilience. First, only few (3%) of these “slow progressing” patients had bulbar onset. While bulbar onset had been frequently correlated with poorer prognosis^22–24^, this cluster analysis suggests that bulbar patients will rarely be classified as a slow progressing disease presentation. A second important observation is low creatinine levels (average of 67 mcmol/L in the first three month of data collection, which is considered abnormally low level compared to the desired range of 74.3–107mcmol/L). Creatinine was reported as a predictor in the previous challenge^16^, and these results suggest that it might also serve as a predictor of this special case of patients with improved prognosis.

A very similar cluster was observed in the analysis of clusters derived from the PRO-ACT survival sub-challenge, with patients living with ALS for an average of 1.5 years while displaying little functional decline (average total ALSFRS-R score 39.4), largely intact breathing functionality (FVC 94.4% of normal on average) and an ALSFRS progression rate of −0.72 points/months.

To characterize clusters and the involved patients for clinical relevance, we compared all pairs of clusters (using ANOVA and t-test, resulting in multiple-testing corrected false discovery rates or FDRs) to assess which features had values specifically different between the clusters (Fig. 4c). We also examined the correlation between feature values and clinical outcome (progression rate) in each cluster to identify features which were important for prediction in some clusters but not in others. Based on the clusters clinical features analysis, the two features unique for this “slow progressing” cluster were urine creatinine and segmented neutrophils (Fig. 4b,c). Both of these features are therefore potential biomarkers of slow progression in ALS. Neutrophils were indeed found to be connected to ALS progression in some studies^33,34^ but not others^35^ and the stratification to sub-groups might shed further light on these results.

Another cluster that was superficially similar but in fact quite different was the “purple” or “early stage patients”. In our pair-wise comparison of features across clusters, the “early stage” and “slow progression” clusters (“purple” and “red” clusters respectively) were similar in having high ALSFRS-R and FVC scores in the first 3 months of assessment, indicating little functional impairment (Fig. 4b,c). However, the distinctive feature of patients in the “purple” cluster was the fact that they were early in their disease, on average ~10 months from symptom onset. Thus, the largely preserved functional state of these patients could be attributed to their early disease stage, rather than a slow progression rate. Indeed, with time these patients became fast progressors (−0.93 ALSAFRS-R points decline monthly on average) and had marginally lower than normal creatinine levels (72.25 mcmol/L on average on the first three months of data). Curiously, urine creatinine was correlated with disease progression (r^2^ = 0.467 p = 0.01, see Supplementary material 7 for figures) only for this cluster, suggesting again that urine creatinine might be useful predictor of disease progression already early in the disease. The relationship between urine creatinine and serum creatinine and with both to muscle breakdown is not straight forward^36^. The correlations to creatinine and Urine creatinine in the “red” (slow progressors) and “purple” (early patient) cluster suggests a scheme to enable early stage assessment of expected disease progression that can aid clinical trial recruitment and clinical care planning, both highly needed early in the disease.

A third “green” cluster of “late stage” patients included patients that were clinically advanced in their disease while being, on average, only 1.7 years from disease onset. These patients were severely disabled in all functional domains (average ALSFRS-R score of 29 points at the beginning of the clinical observation period) and were displaying early signs of breathing dysfunction (average FVC 77%) and shorter survival time (1.5 years on average from first recording time, with an average disease duration of 3.2 y).

These patients had a significant correlation between their ALSFRS “trunk” score (questions about dressing and hygiene and turning in bed) and disease progression (r^2^ = 0.314, p = 0.001), which might be indicative of their advanced disability status. A second feature unique predictive for this cluster was Slow Vital Capacity (SVC) (Fig. 4c, examples for both features are available in Supplementary material part 7). These two classifiers should be taken into consideration clinically as they might be stronger indicators that the patients are reaching the final stages of their disease. The “trunk” score indeed represents complex functions that require the combined efforts of upper and lower motor neurons, and is therefore more clearly impaired later in the disease. SVC, while highly correlated with FVC, might become predictive later in the disease when respiratory function diminishes. They could also be used for clinical trial exclusion criteria to improve patient survival throughout the trial. Indeed, SVC was recently suggested as an indicator of respiratory failure in ALS^37^.

The last cluster, “blue” or “fast progressing” patients, represents the most critical patients, who have been experiencing symptoms for only 10 months on average, but were already significantly impaired in all functional categories (average ALSFRS-R score of 35.75) at the beginning of the clinical observation period. These patients continue to have a very fast disease progression rate (ALSFRS progression slope of −1.05points/month) and an overall average survival of only 2.7 years from disease onset. Half of the patients in this cluster had bulbar onset (compared to 20% bulbar onset across all patients) and were more likely to have lower scores in all ALSFRS-R functional domains (leg, hand, trunk, bulbar and respiratory functions) and to have significant weight loss over the 1-year observation period (average of 5 kg lost per year).

Importantly, a similar yet more severe cluster was observed in the PRO-ACT survival data, with patients showing diminished disease states (initial ALSFRS-R of 24 ALSFRS points) and survival (average of 443 days from trial onset). Women were more likely to be fast progressors, making up 53% of this cluster, compared to 40% women across all clusters, even beyond their higher likelihood to have bulbar onset (32% of women). This cluster of patients with the poorest prognosis was also found in the registry consensus clustering for both the progression and the survival data.

When looking at features that were significantly different between almost all pairs of clusters, a noticeable observation is the discriminative power of ALSFRS question 1 (speech) and the combined “mouth” measure (averaging ALSFRS questions 1–3), highlighting again the important role of bulbar function in discriminating ALS consensus clusters. Overall this cluster helps integrate information, some already accepted (such as the association of bulbar onset and respiratory signs with poorer prognosis) and some suggested (the potential predictive roles of creatinine, urine creatinine, neutrophil and others) in a statistically supported unified framework, enabling discerning fast and slow progressing patients earlier in their disease course, as well as markers helping to identify patient reaching the final stages of their disease.

## Discussion

Disease heterogeneity, and particularly large unexplained variance in disease progression rate and survival, is a hallmark feature of ALS disease^38^. The hypothesized existence of distinct subgroups of ALS patients and their importance for ALS research and clinical care were highlighted in recent years by clinical trials in which only a subset of patients responded to the tested treatment^3,39^. Despite some recent advances from genetic studies^40,41^, there is currently no generally accepted stratification scheme for ALS patients, and more importantly, no consistent way to tailor survival and disease progression estimates to individual patients. The ALS Stratification Challenge was a global crowdsourcing effort aimed to develop new tools and insights for understanding patient subpopulations as they relate to ALS disease progression and survival. Thirty teams submitted algorithms to the challenge, with the winning solutions outperforming currently available prediction algorithms (adapted from the previous ALS Prediction challenge)^16^.

Compared to the ALS Prediction Challenge^16^, the current challenge included additional data and design features that made its resulting algorithms more robust and more relevant for clinical application, including the use of community-based data, the limitation on the number of features used for prediction, and the prediction of survival as well as disease progression. Another requirement highly relevant to the application for future clinical trials: the validation of algorithms on a dataset derived from completely independent clinical trials.

The current challenge invited participants to develop prediction algorithms either based on clinical trial data or from ALS registries containing data collected through ALS clinics. This is the first time that registry data was made publicly available, and the design of the challenge enabled us to directly compare performance of prediction algorithms when applied to PRO-ACT vs. registry data. We suggest a number of clinical features (Fig. 3), such as FVC and weight, which could be added to routine clinical assessment to potentially improve prediction accuracy and aid clinicians in predicting individual patient prognosis. Conversely, a number of features that could be found exclusively in the registries data, including common genetic mutation data and detailed onset site assessments, were both highly informative for prediction and should be considered for incorporation into ALS clinical trial screening or baseline assessment.

The main goal of this challenge was to uncover clinically meaningful subgroups of ALS patients, a challenging task since no known “ground truth” exists for ALS patient stratification. In this study we designed a novel “bottom-up” method for the identification of consensus patient clusters and the determination of discriminating features (see online methods). We did not make any a-priori assumptions regarding patient sub-populations, but instead defined patient clusters by a “consensus vote” based on participants’ submitted algorithms. Challenge participants were free to base their clustering on any subset of the available clinical data, choose any type of clustering method and any number of clusters. While clustering was not strongly related to immediate benefit in algorithms’ prediction accuracy, it did reveal consistent patterns of patient classification that are of great clinical interest and that was robust enough to enable classification of new patients with high degree of success. We suggest that these clusters could be used to identify subgroups of patients to guide further research of disease mechanisms and the planning of individual patient care programs and ALS clinical trials. As most clinical trials aim to enroll patients early in their disease to ensure a sufficient therapeutic window, separating patients that will be slow or fast progressors early is critical to enable correct clinical trial development and interpretation. Similarly, signed of end of life in patients can aid patient decision making and clinical care substantially.

The results of this study can help accelerate disease understanding in several ways: the stratification scheme suggested in this analysis offers novel insights that can integrated in the development of novel ALS therapeutics, aiding patient selection and result interpretation. Novel differentiating features such as creatinine or SVC can also help shed light on mechanisms related to disease progression, as well as mechanisms related specifically to end of life in ALS, a topic of critical clinical importance. Ideally, in the future, clinical data such as described here would be further integrated with data obtained from different types of high-throughput technologies (such as transcriptomic, genomics, metabolomics), allowing for the identification of predictive biomarkers for early diagnosis and treatments. Several such highly needed large scale initiatives are being developed now^42^.

Given the covert nature of ALS patient stratification, only a large-scale crowdsourcing effort, where different and independent teams apply diverse methods on a similar and large enough dataset can uncover such an underlying population structure free from a-priory assumptions. This communal approach indeed revealed a few sub-groups of patients which not only tended to cluster together across different algorithms but also displayed similar characteristics across different sub-challenges - clusters which may be the basis for a new stratification framework for ALS patients. Overall, we could significantly differentiate four patient groups: slow progressing patient and fast progressing patients, as well as patients with an average progression rate which were either early or late in their disease at the beginning of the recorded clinical observation period.

We examined the features most often chosen for prediction by the different challenge participants to assess their predictive power. This analysis revealed several features that could help classify patients into sub-groups. While some features are already well described, such as age, gender^22–24^ and respiratory capacity.^22,29,43–45^ and other, such as limb motor function, specific ALSFRS-R scores^46^ and creatinine^16,29,47–49^ and specific ALS staging scores were at least suggested as predictive, our results not only supported these findings but help put them in to a more usable and testable context. For example, creatinine was found to be predictive specifically for patients early in their disease. Segmented neutrophils were also suggested by our analysis as a relevant novel predictor, specifically for slower progressing patients, while SVC and ALSFRS “trunk” scores were associated with outcomes only specifically for patients later in their disease.

Overall, these results suggest a novel stratification scheme for ALS, with relevant classifiers and group-specific predictors. Stratification is highly needed to advance clinical development, for clinical care and to allow more personalized treatment. The tools and insights presented in this study can offer a first attempt for improvement in clinical trial development, selection and interpretation, accelerating the development of a much-needed treatment for a dire aliment such as ALS. More broadly they open the door to a new avenue of research using crowdsourcing approaches to uncover patient sub-groups unattainable by other means.

## Methods

### Datasets

The challenge made use of two datasets: data collected during clinical trials (PRO-ACT) and data collected during routine visits to ALS clinics (ALS registries). Both datasets included both static and time resolved measurements covering a wide range of data types and clinical measurements (full data dictionaries for both datasets can be found in the supplementary material). The time in which measurements were taken was noted in days relative to clinical trial onset or to first clinic visit available on records (time “delta”). Data was provided to challenge participants in tabular form. Each line represented the measurement of a single feature for a single patient at a particular time point Outcome measures (ALSFRS slope for progression and survival) for the training datasets were provided to challenge participants in separate tables.

#### PRO-ACT

The Pooled Resource Open-Access ALS Clinical Trials (PRO-ACT) Database was formed in 2011 by Prize4Life in collaboration with the Neurological Clinical Research Institute (NCRI) of Massachusetts General Hospital, the Northeast ALS Consortium, and with funding from the ALS Therapy Alliance. PRO-ACT contains data collected during phase II/III ALS clinical trial, volunteered by PRO-ACT Consortium members. Data from 17 clinical trials^15^ was used for the previous prediction challenge^16^ and later made publicly available via the PRO-ACT web platform (www.ALSdatabase.org). This data was used for algorithms’ training in the current challenge. Data from 6 additional clinical trials^50–55^, never before made publicly available, was homogenized to comply with PRO-ACT data format standards and used for algorithms’ validation and assessment. Similar to the design of the previous challenge, the data used for validation was randomly subdivided into a test set (leaderboard data, 400 patients) and a validation set (1,488 patients). Challenge participant could submit versions of their code to be tested on the test set (limited to once per week per team to avoid overfitting) with the results published on a public leaderboard which served to provide feedback to participants. After the challenge was completed all submitted algorithms were assessed using the validation set data (see main manuscript, Fig. 2).

#### ALS registries data

Community-based data, never before publicly released, used in this challenge was collected through two ALS registries: 1) The Irish National ALS Register including data collected from ALS clinics in Ireland. 2) The Piemonte and Valle d’Aosta Register for ALS, including data collected from ALS clinics in Piemonte and Valle d’Aosta region of Italy. Data from the two registries was merged, harmonized and converted to the same format as the PRO-ACT data. Data was divided into a training (986 patients) and a validation (493) set. Stratification of data ensured a similarly proportional representation of patients from the Irish and Italian cohorts but was otherwise done randomly. Due to the small number of patients available in this dataset there was no test set (leaderboard data) available to challenge participants.

### Definition of predicted outcome measures - “ground truth” calculation

#### Disease progression

ALS disease progression was defined as the slope of the total ALSFRS score, similar to the definitions used in the 2012 prediction challenge^16^. Briefly, ALSFRS was calculated as:

$$\frac{ALSFRS({t}_{2})-ALSFRS({t}_{1})}{{t}_{2}-{t}_{1}}$$

where t_1_ and t_2_ were the time points at the first and last clinic records in the relevant time period 92–365 days in which total ALSFRS scores were recorded. Whereas time data in the both challenge datasets was given in days, it was converted to months for the calculation of ALSFRS slope, according to the following: t_(months)_ = (t_(days)_/365) *12). Patients had to have at least two clinical records in the relevant time period for their data to be used for validation. Participants were required to write algorithms that would predict ALSFRS slope between 3 and 12 months, based on data collected in the first three months of clinical records.

#### Survival

Survival was defined as time until death or until tracheostomy surgery (the introduction of invasive breathing tube- time where without intervention the patient was unlikely to survive), whenever this information was available. For patients who had no time of death logged on the clinical records the time of the last clinical visit was recorded in the survival records with a status indicating they were alive.

Patients whose final records were on or before day 90 (from onset of clinical trial or of clinical records) were excluded from the survival analysis. Challenge participants were required to write algorithms that predicted the likelihood of survival for each patient at three time points; 12, 18 and 24 months from the onset of clinical records.

### Predictions assessment and scoring scheme

All methods for performance assessment were based on evaluating how close the algorithms’ predictions were compared to the respective ground truth, averaged across all patients. We used three different evaluation metrics to assess submitted algorithms’ performance in the disease progression sub-challenges. Two methods, the root mean square deviation (RMSD) and Pearson’s correlation (PCC) were used and described in the previous ALS prediction challenge^16^. In the current challenge we added a third evaluation metric: the concordance index (CI), which evaluates the similarity of ranks between predicted and actual ordered lists of measurements. The concordance index was the only metric used to evaluate performance in the survival prediction sub-challenge, since it is commonly used in survival analysis and is best suited for assessing censored data^56^. When there is no censored data or ties, the *c*-index between a predicted list of survival times of *n* patients, $Pred=\{{p}_{1},{p}_{2},\ldots ,{p}_{n}\}$, and the actual survival times for the same *n* patients, $Actual\,=\,\{{a}_{1},{a}_{2},\ldots ,{a}_{n}\}$, is calculated as:

$$CI=\frac{2}{n(n-1)}\sum _{i < j}h(i,j)$$

where,

$$h(i,j)=\{\begin{array}{c}1,\,\mathrm{if}\,({a}_{i} > {a}_{j}\,\& \,{p}_{i} > {p}_{j})\,OR\,({a}_{i} < {a}_{j}\,\& \,{p}_{i} < {p}_{j})\\ 0,\,\mathrm{if}\,({a}_{i} > {a}_{j}\,\& \,{p}_{i} < {p}_{j})\,OR\,({a}_{i} < {a}_{j}\,\& \,{p}_{i} > {p}_{j})\end{array}$$

Please see supplementary data on details accommodating for the possibilities of ties and censored data.

The three performance assessment scores (or three CI values, in the case of survival prediction) were combined using Z-score transformation. We generated 100,000 random sets of predictions for each of the four sub-challenges, by shuffling the assignment of “ground truth” progression or survival values to patients. We then calculated the RMSD, PCC and CI scores for each of these random shuffles and used the resulting three sets of 100,000 values to calculate the mean and standard deviation (SD) of each of the three scoring metrics. These mean and SD values were used to calculate the Z-scores for the three assessment metrics for each of the submitted algorithms, given by:

$${z}_{score}=(score-mean)/SD$$

$${z}_{slope}={z}_{CI}+{z}_{PCC}-{z}_{RMSD},\,{z}_{survival}={z}_{12}+{z}_{18}+{z}_{24}$$

Outcomes of the survival sub-challenges were further validated by a receiver operating characteristic analysis (time ROC analysis). For the purpose of the challenge, time ROC was calculated using the R-package timeROC (https://cran.r-project.org/web/packages/timeROC/timeROC.pdf). On the plus side, the timeROC analysis incorporated the three survival time frames to be predicted (12, 18, 24 months), and performance is thus specific to the time frame in contrast to the analysis via CI, where the same set of predictions would evaluate the same irrespective to the time frame. However, the CI is better suited to represent the right censured nature of the survival data. The rank ordering of submitted algorithms for both survival sub-challenges was very similar when comparing the outcomes of the combined z-transformed CI index and the time ROC analysis. Results of the time ROC analysis could be found in the supplementary material.

### Consensus clusters and determination of discriminating features

For each subchallenge, we integrated the teams’ clustering into a consensus clustering. First, we created a square patient x patient co-clustering matrix *M* where each matrix element *m*_*i,j*_ contained a normalized score that expressed how often the corresponding patients *i* and *j* appear together in a single cluster across all team submissions. The normalized score takes the size of the teams’ clusters into account such that two patients clustered together in a smaller cluster receive a larger weight. The scores *m*_*i,j*_ were calculated as the sum of contributions (eq. 1), across all submissions *s*, where patients *i* and *j* appear together in the same cluster:

$${m}_{i,j}=\,{\sum }_{s=1}^{size\_s}\mathrm{log}\,\frac{p}{{C}_{s}\,}if\,i,\,j\,in\,the\,same\,cluster\,in\,S;0\,otherwise,$$

where *p* is the total number of patients in the given sub-challenge and *c*_*s*_ is the number of patients in the cluster of submission *s* that includes both *i* and *j* (to normalize by cluster size).

We next determined the statistical significance of *m*_*i,j*_ values, i.e. to determine whether patients *i* and *j* are clustered together more often than expected by chance. We randomly assigned patients into clusters of the same size as contained in the original submissions and repeated this random sampling process 100 times. We then calculated *m*_*i,j*_ scores for each set of randomly assigned clusters, giving us 100 *m*_*i,j*_ scores for each *i*, *j* patient pair. We used these permuted *m*_*i,j*_ value to calculate a false discovery rate for any given *m*_*i,j*_ score derived from the participants’ submissions data, by assigning:

$$FDR(i,j)=\frac{100\ast {N}_{perm}\ast {N}_{true}}{{N}_{false}}$$

where *N*_*perm*_ is the number of permutations, *N*_*true*_ and *N*_*false*_ refer to the number of scores computed from submitted and permuted data, respectively, that were less or equal than the given score. This approach to FDR calculation was adapted from significance analysis of microarrays^57^ and described in detail in previous work^58^. Subsequently a FDR threshold of 5% was applied to identify significantly pairs of patients that significantly tend to be clustered together.

A graph of significant pairs was then plotted using the graphviz software package in order to visually determine a plausible number of clusters *k*. To generate the final patient clusters, we performed k-means clustering for *k* clusters based on the matrix M using average linkage and Pearson’s correlation metric. Thus, the correlation metric calculates the distance between patients *i* and *j* by comparing the corresponding rows, denoted as *m*_*i*_ and *m*_*j*_, in the matrix *M*. This analysis identified three consensus clusters for both survival and the registry progression sub-challenges and four clusters for the PROACT progression sub-challenge.

### Detection of features differentially distributed across patient groups

After finding a small number of consensus clusters of patients for each sub-challenge, we determined the features that discriminate between consensus clusters by statistical tests. Values of longitudinal variables (e.g., ALSFRS) were averaged across time, and we looked at average values of two different time periods consistent with the challenge’s overall design: 0–3 months and 3–12 months. We applied one-way analysis of variance (ANOVA) to continuous features (e.g. age of onset) and Fisher’s exact test to discrete features (e.g. site of onset). This leads to two matrices, *C* and *D*, capturing the values of the continuous and discrete features, respectively. Rows in these matrices correspond to the features, columns to the patients. In addition, each feature vector (containing the values for that feature across the *p* patients) was randomly permuted a hundred times and statistical tests were applied to the 100 randomized datasets to compute FDRs (analogous to the description above) separately for the results from Fisher’s exact test and ANOVA.

**Figure 5:**
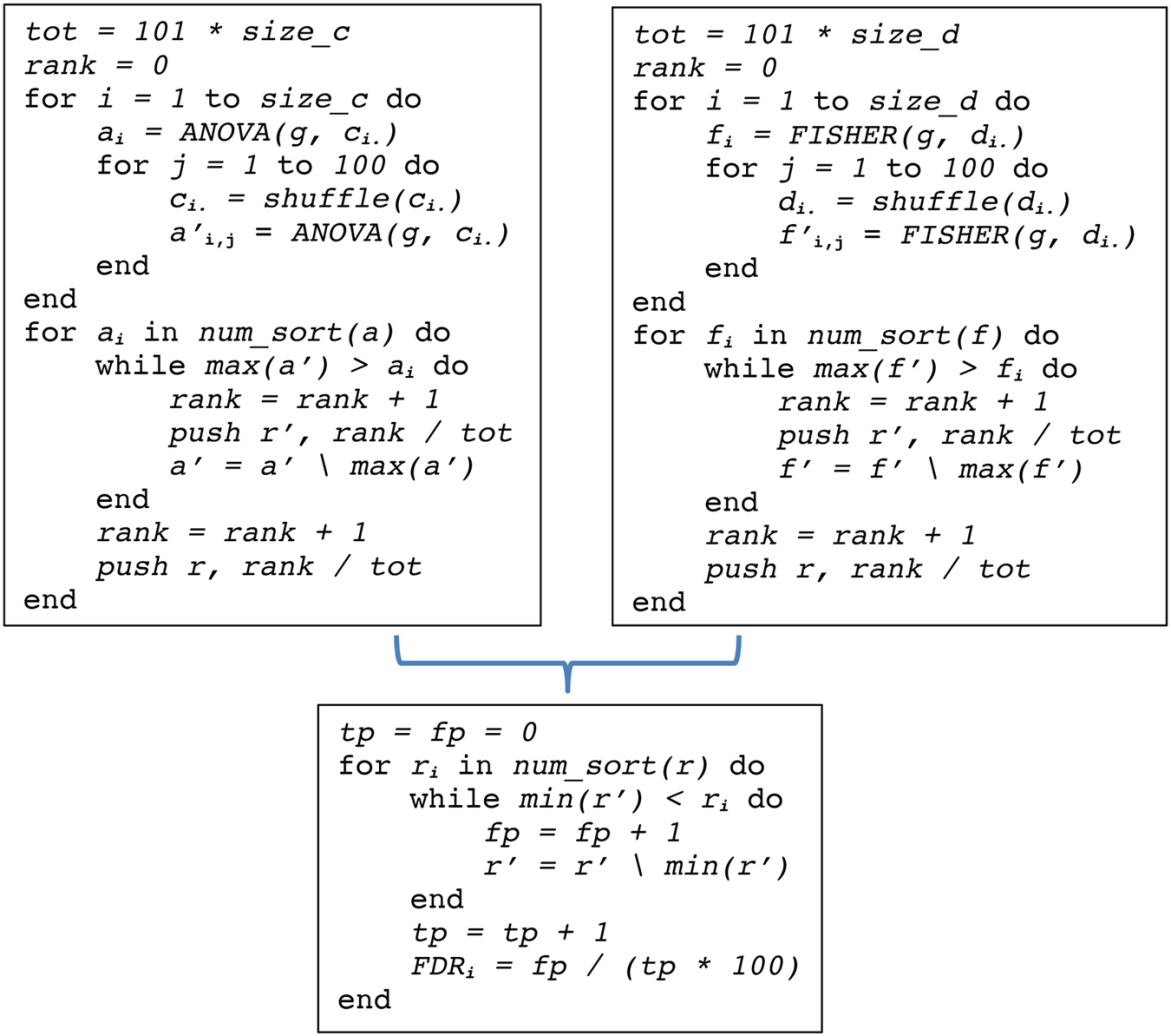
Pseudo code analysis of differentially distributed features. The pseudocode in the left panel illustrates the computation of the ANOVA test statistic *a* at the example of the continuous features in *C*. The design *g* specifies the mapping of patients to clusters. A permuted test is calculated by shuffling values in rows of the matrix *C* 100 times, computing their associated test statistics *a’* and pushing their relative ranks and the relative ranks of *a* separately into arrays *r’* and *r*, respectively. The backslash notation denotes the removal of an element, i.e. in a’ = a’\max(a’), the entry with the highest value is removed from a’. Analogously, the Fisher test statistic *f* is calculated for the discrete features in *D* (pseudo code in the right panel). Finally, FDRs are calculated by comparing the relative ranks from the true statistics *r* vs. the relative ranks from the permuted statistics *r’* across discrete and continuous features (pseudocode in lower panel).

We then regard the relative ranks from the ANOVA and Fisher as new test values. From these, we compute FDRs now across discrete and continuous features together. By this integrated statistical assessment, we obtained FDR values for all features. Features were deemed statistically significant across all clusters if they exhibited FDR values of <5%. See below pseudo code (Fig. 5) for details.

Subsequently, t-tests were applied to identify all pairs of clusters where ANOVA determined that continuous features exhibited significantly differential values (FDR < 5%). The same permutations were applied to the feature vectors as above, such that we were able to transform t-test p-values into false discovery rates analogously. As before, we regard a feature as statistically significantly different between two clusters if such a comparison resulted in an FDR <5%. FDRs do not need multiple testing correction as it is already built into the permutation test. Note that we applied the ANOVA “trick” here: pairwise comparisons were only performed (and thus subject to multiple testing correction) for features with overall significance across all clusters. This leads to a less severe multiple testing correction and accordingly to a more sensitive test for the pairwise comparisons in contrast to the case were pairwise comparisons would have been performed and corrected for all features.

In order to summarize the differences between the clusters more succinctly, we aimed to integrate the pairwise comparisons, by collapsing the metrics based on pairs of clusters into a metrics based on individual clusters. We created a simple rank order statistics for each feature and cluster by counting pairwise comparisons in the following way: If a comparison between a pair of clusters (*a*, *b*) is significant, and the given feature displays higher values in *a* as compared to *b*, we increase the rank of *a* via *r*(*a*) = *r(a*) + 1 and decrease the rank of *b* via *r*(*b*) = *r(b*) − 1. After all pairwise comparisons are integrated, a heatmap is created (Fig. 4c) by linearly scaling each feature to [−1 … +1] range.

### Assignment of patients to consensus clusters (re-clustering)

The purpose of re-clustering is to assign new patients to the previously defined consensus clusters. While the consensus clusters were derived based on the submissions of the individual challenge participants, the re-clustering is based on the values of the features, i.e. new patients are assigned to clusters where feature values of patients and average feature values of the consensus clusters match best. The features to be matched are those features previously determined to be discriminating. The procedure involves two steps, (1) normalization of feature values and (2) matching. Feature values are normalized by subtracting the average and dividing by the standard deviation of each feature, i.e. they are transformed into z-scores. Subsequently, patients are tested against the averaged and z-score normalized feature vector of each consensus cluster via the uncentered correlation. Each patient is then assigned to the cluster that resulted in the highest value of the uncentered correlation. A tenfold cross validation (10CV) has been applied to determine assignment accuracy. Here, 10% of all patients (CV test set) have been removed in each step of CV before differentiating features were determined. After doing this 10 times, accuracy was determined based on the assignment of patients across the 10 test sets.

## Supplementary information

Supplementary Info 1

Supplementary Info 2

Supplementary Info 3

Supplementary Info 4
