# Supplementary material for: Stratification of amyotrophic lateral sclerosis patients: a crowdsourcing approach
Source: Sci Rep. 2019 Jan 24;9:690. doi: 10.1038/s41598-018-36873-4 (PMC6345935; doi:10.1038/s41598-018-36873-4)
Supplement: Supplementary file 1 — Supplementary Info 1 [file 41598_2018_36873_MOESM1_ESM.pdf]

Supplementary Methods and Results to Stratification of amyotrophic lateral sclerosis patients: a crowdsourcing approach

Robert Kueffner, Neta Zach, Maya Bronfeld, Raquel Norel, Nazem Atassi, Venkat Balagurusamy, Barbara Di Camillo, Adriano Chio, Merit Cudkowicz, Donna Dillenberger, Javier Garcia-Garcia, Orla Hardiman, Bruce Hoff, Joshua Knight, Melanie L. Leitner, Guang Li, Lara Mangravite, Thea Norman, Liuxia Wang, the ALS Stratification Consortium, Jinfeng Xiao, Wen-Chieh Fang, Jian Peng, Chen Yang, Huan-Jui Chang and Gustavo Stolovitzky

Contents

Supplementary material part 1- Challenge data 3

Table 1.1- Datasets used in the challenge: number of patients and available clinical information 3

Supplementary material part 2- Challenge information as given to participants 25

Figure 2.1- overview of the challenge 31

Supplementary material part 3- Summary of the ALS Stratification Challenge Participants Survey 33

Figure 3.1- Geographical Distribution 34

Figure 3.2- Career Stage 35

Figure 3.3- Primary Research Field 35

Figure 3.4- Team Size 36

Figure 3.5- Prior knowledge about ALS 36

Table 3.1- Sources of information on ALS: 36

Figure 3.6- Participants Views Regarding Challenge Impact 37

Table 3.2- Participants Views Regarding Challenge Impact 37

Figure 3.7- Challenge Marketing 38

Figure 3.8- challenge difficulty 39

Figure 3.9- Challenge Barriers 39

Figure 3.10- Participants' Motivations to Continuing to Work on ALS Data 41

Figure 3.11- Occurrence of Different Motivations Being Ranked as Top Motivation for Further Work 41

Table 3.3- Ranking of challenge motivations 42

Supplementary material part 4- Baseline algorithms 43

Figure 4.1 – Preparation of baseline algorithm 1 43

Table 4.1- Top features used for prediction by Baseline algorithm 1 for predicting PRO-ACT progression 45

Figure 4.2- Performance of baseline algorithm 1 for the PRO-ACT progression sub-challenge 45

Figure 4.3 -Performance of baseline algorithm 1 for the PRO-ACT survival sub-challenge 46
Table 4.2- Top features used for prediction by Baseline algorithm 1 for predicting PRO-ACT survival 46
Supplementary material part 5- Full results, algorithm description and aggregated performance 48
Table 5.1- Algorithms submitted to the four sub-challenges 48
Table 5.2- Full results- PRO-ACT survival sub-challenge 49
Table 5.3- Full results- PRO-ACT progression sub-challenge 49
Table 5.4- Full results- Registry survival sub-challenge 49
Table 5.5- Full results- Registry progression sub-challenge 50
Figure 5.1- Performance of aggregated predictions. 51
Figure 5.2- Aggregated performance for the PRO-ACT prediction sub-challenge 51
Figure 5.3- Aggregated performance for the Registry prediction sub-challenge 52
Figure 5.4-Aggregated performance for the PRO-ACT survival sub-challenge 52
Figure 5.5-Aggregated performance for the registry survival sub-challenge 52
Supplementary material part 6- Examples for correlation with prognosis occurring only in one cluster......54
Figure 6.1- Example 1- correlation between Trunk ALSFRS measurements and disease progression are only visible in “late stage” or "green" cluster 54
Figure 6.2- Example 2- correlation between respiratory functions and disease progression are only visible in “good prognosis” or "red" cluster 55
Figure 6.3- Example 3- correlation between creatinine levels in serum and disease progression are only visible in “early stage” or "purple" cluster 56
Figure 6.4 Features especially predictive across clusters 56
Figure 6.5 Features distinct only for the "late stage" or "green" cluster 58

Supplementary material part 1- Challenge data

Table 1.1- Datasets used in the challenge: number of patients and available clinical information

	PROACT	Registries
Number of patients in data set:		
Training	8,835	986
Leaderboard	400	-
Validation	1,488	493
**Total**	**10,723**	**1,479**
Available Features:		
Clinical outcomes (ALSFRS, FVC, survival)[a,b]	+	+
Demographics	+	+
ALS personal history (onset, diagnosis, etc.)	+	+
Family history (ALS & other neurological conditions)	+	+
Medical History (non-ALS)	-	+
Vital signs [c]	+	+
Laboratory data	+	-
Treatment and medication (other than Riluzole)	+	-
Riluzole	+	+
Genetic testing	-	+
Cognitive status	-	+
Adverse events [d]	+	+
Assistive technology	-	+

[a] PROACT also includes SVC data for some patients.
[b] Registry also includes ALS staging data.
[c] Both datasets include weight information and each dataset includes additional unique vital signs features.
[d] Different data logging standards were used for the different data sources, thus adverse events information is only partially overlapping between datasets.

PRO-ACT data description

The data contains information from ALS patients that participated in clinical trials who were donated to the PRO-ACT database. The training set contains data from ALS clinical trials donated in the past to the PRO-ACT database and the final validation set data include patients from clinical trials donated to PRO-ACT that are not currently available on the PRO-ACT website(added after the challenge was completed). Of that data 200 patients were spiked into the training set. Note that some rare features might only be available in some of the datasets.

All of the data is de-identified to protect patient privacy. Different types of information may be available for different patients because the data was generated in multiple clinical trials and clinics. Some patients received placebo treatments, while others received experimental treatments (medication), however the medications tested in these specific trials were found to be no better than placebo with respect to their effects on ALS progression or other clinical outcome measures.

Each subject is identified by a SubjectID and the specific assessment for this subject is identified by a record (each subject has multiple records). The assessments are separated into data types:

- ALSFRS(R)
- Death Report
- Demographics
- Family History of ALS
- Forced Vital Capacity
- Laboratory Data
- Riluzole use
- Slow Vital Capacity
- Subject ALS History
- Treatment Group
- Vital Signs
- Concomitant medication use
- Adverse events

The time at which an assessment was taken (a record was created) is listed as the assessment's delta. Delta is given as days since the trial onset(which is listed as delta 0). A negative delta lists events occurring before the official beginning of the measurement (for example symptom onset would always have negative delta as they predate diagnosis as ALS patients). Events that do not change over time (such as demographic information) don't have a delta associated with them.

In the data file you will find a SubjectID for each patient, indicating which subject it is. You will find the same SubjectID across different data types and different assessments e.g. if the same patient had both vital signs and lab tests measured, those respective records will include the same SubjectID.

Beyond the SubjectID, the data contain different assessments and their respective results including data types. Specific measure, value, unit of measurement, and delta (time in days from trial onset when the assessment was made). You can identify these variables through the column name in the data file.

The data format lists

PatientID|datatype(form name)|feature name|feature value|feature unit|delta

For example:

7824|ALSFRS(R)|ALSFRS Total|30||0

7824|Vital Signs|Blood Pressure (Systolic)|140|MMHG|14

7824|Vital Signs|Pulse|76|BEATS/MIN|14

Patient 7824 had, at delta= 14 (day 14 from beginning of measurement), the following vital signs: a blood pressure(Systolic) of 140MMHG and a Pulse of 76 BEATS/MIN. At a delta of 0 (first day of measurements) their ALSFRS total is 30.

Treatment

The exact medications used in the clinical trials data are not specified, as part of our effort to avoid identification of the patients involved, however, information is available as to whether any individual patient received medication or placebo, and this information is listed in the Treatment Group datatype as [**Treatment Group- Active**; indicating experimental treatment was given or **Treatment Group- - Placebo**; indicating placebo was given].[**Treatment Group Delta**] refers to the time duration between the first time the patient was assessed during the trial and the time medication (or placebo) was first given. The first time the patient was assessed during the trial (typically screening visit) is indicated as Time 0. The time medication/placebo was given, is the time in days since that first day. Note that different patients started their respective trials at different days.

Family and Medical History

ALS affects approximately 5 out of every 100,000 people worldwide. In about 5-10% of cases, ALS is seen in multiple family members and this form is known is 'familial ALS'.Multiple mutations (over 35) have been identified for such patients. In the remaining cases, called sporadic cases, there is no more cases observed in the families (and only a minority of these patients had the mutations identified in the familial cases). For this reason, the datatype Family History contains information about close family members (up to second degree family, but different studies differ in the exact to which second degree relatives were assessed) and whether or not they had ALS (Y indicated ALS in the family, N indicates no ALS in the family)

Demographics

Demographic information is available in the Demographics datatype, including [**Age, Gender, Race**] at screening (time 0). Age is available in years at trial onset, Gender as M/F and Race as [American Indian, Asian, Black, Hawaiian, White, Other or Unknown]

Subject ALS history

The major symptoms of ALS broadly include muscle weakness, paralysis, drooling, gagging, muscle cramps, involuntary muscle contractions/twitches called fasciculations, speech problems, and breathing problems. As ALS progresses, patients lose their ability to control voluntary muscle function. Symptoms progress from muscle weakening, twitching, and an inability to move the arms, legs, and body, into full paralysis. When the muscles in the chest area stop working, it becomes hard or impossible to breathe on one's own.ALS typically does not affect the senses (sight, smell, taste, hearing, touch).

Time of symptom onset is listed in the data as [**Onset_delta**]- time in days between symptom onset and trial onset. Time of diagnosis with ALS is listed in the data as [**Diag_Delta**]-time in days between diagnosis and trial onset. Note that both of these events (onset and diagnosis) always occurred prior to the start of the trial, therefore the deltas for each of these events are always negative.

The site of disease onset [**onset site**] as experienced by the patient can be a limb ("limb onset") or the muscles controlling speaking and swallowing ("bulbar onset") or occasionally both. Information is available in the database regarding a given patient's site of onset [**Limb, Bulbar, Other, Limb and Bulbar, and Spine** (which is synonymous with limb onset; different terminology was used for different patients)].

Symptoms and outcome measures (FVC, SVC, ALSFRS, and Survival datatypes)

Symptom severity is frequently assessed using two functional scales: ALSFRS (ALS Functional Rating Scale) and its modified version ALSFRS-R. The ALSFRS scale is a list of 10 assessments regarding motor function, with each measure ranging from 0 to 4, with 4 being the highest (normal function) and 0 being no function. The score for

the individual questions are then summed together to generate a number, and that is the ALSFRS score. ALSFRS-R is a modified version of the ALSFRS. Whereas in the ALSFRS there are 10 assessments, in the ALSFRS-R one of the assessments, #10 (respiratory function) was further divided into three questions to better reflect the importance (weighting) of respiratory changes within the scale. Therefore ALSFRS-R, contains 12 questions (9 of these identical to the traditional ALSFRS) and a maximal score of 48. Please note that some of the patients in the dataset will have ALSFRS scores and some will have ALSFRS-R.

The individual questions comprising the ALSFRS or ALSFRS-R scores are available in challenge description [**Q1_Speech, Q2_Salivation, Q3_Swallowing, Q4_Handwriting, Q5a_Cutting_without_Gastrostomy, Q5b_Cutting_with_Gstrostomy** (gastrostomy is a feeding tube; The scores were also added to form **Q5_cutting**), **Q6_Dressing and Hygiene, Q7_Turning in Bed, Q8_Walking, Q9_Climbing Stairs, Q10_Respiratory**(only available for those measured with ALSFRS)**, R1_Dyspnea, R2_Orthopnea, R3_Respiratory Insufficiency**]. The total sum is available as [**ALSFRS_Total, ALSFRS_R_Total**]. The time between the first time a patient was observed (Time 0) and the time of each assessment of ALSFRS or ALSFRS-R over the course of the trial is listed as [**Delta_Days_visit**].

In cases where one question was missing, but scores available for that question from preceding and proceeding measures, the score was imputed by the original data donors. This sometimes results in non-integer scores (such as 1.5, 2.5, etc).

Due to the limitation on number of features to be used in this challenge, we added several composite scores combining several intercorrelated ALSFRS questions: Q1-3 are [**mouth**], Q4-5 are [**hand**] Q6-7 are [**trunk**], Q8-9 are [**leg**] and either Q10 or R1 ,whichever is available, is [**respiratory**]. For ALSFRS-R there is also the composite score [**respiratory-R**], combination of questions R1-3.

The file ALSFRS slope includes the gold standard for slope prediction for the training set.

In addition to ALSFRS, there is another frequently used measure of ALS disease status called forced vital capacity or **FVC**. Forced vital capacity is the volume of air that can forcibly be blown out after full inspiration, measured in liters. FVC is available- in the datatype Forced Vital Capacity- for some of the patients. FVC is typically reported in the literature as either liters of vloulme of air or as percentage of the liters expected for a non- patient (matched for age, gender and height; 120% is an athlete, 100% is normal, 80% is deteriorating, and 50% is very low breathing capacity/ready for a ventilator). Here we have both. [**FVC Normal** (the expected value for a non-ALS patient (control) matched by gender, age and height), and then attempts to measure FVC in liters – [**FVC1** and **FVC_percent_1**] are the first attempt to measure FVC, in liters and in percent from normal. [**FVC2** and **FVC_percent_2**] are the second attempt and [**FVC3** and **FVC_percent_3**] are the third attempts. It is common to regard the highest (best) of these attempts. Alternatively, the average of the attempts is available as [**FVC and FVC_percent**]. [**Delta_Visit_days]** is time from trial onset, in days. Another measure of lung

function is slow vital capacity (SVC). Slow vital capacity is the maximum volume of air that can be exhaled slowly after slow maximum inhalation, also measured in liters [**SVC**] or percent from normal [**SVC percent**] and the time of assessment is given as [**Delta_Visit_days]**. SVC is typically greater than FVC.

Finally, time of death is available- in the file Survival Response- whether the subject died [**Status; 1=died**] while monitored and if that is =1 (indicating the subjected indeed died), also the time of death, measured in days from trial onset [**time_event**]. For the subjects that didn't died (status=0, either indicated by the trial managers or by the last time the patient was assessed).

<u>Vital signs</u>

Vital sign data collected for each patient-available in the datatype Vital Signs- within the different trials include: [**Delta_days_visit**]- the time when they were assessed compared to Time 0. Blood pressure and pulse: [**bp_diastolic** (Diastolic blood Pressure**), bp_systolic** (Systolic blood pressure); units - mmHg]. Height and weight: [**Height**(units- cm), **Weight**(units- kg), **BMI].**Body temperature- [**Temperature**(Units- C)]. Pulse: [**Pulse** (units- beats per minute)]. Respiratory rate- [**Respiratory Rate** (units-Breaths per minute)]

<u>Concomitant Medication Use</u>

Concomitant Medications are medications use by the patients in the clinical trials that are not the medication tested in the trial. These could be due to the patients other conditions, acute or chronic, that are not ALS, supplements favored by the patients or medication related to any adverse events form the treatment. Information includes **[Medication coded],**with their corresponding**[Dose], [Unit], [Route]** and **[Frequency]**, as well as [**Start Delta**] and [**Stop Delta**] when such information was available.

Note that while the general data format for concomitant medication is the same as the rest of the data, the feature value and delta columns are more complex. The feature value represents three pieces of information, namely Dose, Frequency and Route. The delta column represents start delta and stop delta:

PatientID| Concomitant Medication|Medication Coded|Dose;Frequency;Route|Unit|Start Delta;Stop Delta

One specifically noteworthy medication is Riluzole, the only approved medication for the treatment of ALS. Riluzole use is available as the datatype Riluzole_Use [**If_use_Riluzole**, values are yes and No; **Riluzole_use_delta** is time of recording of Riluzole usage, in days from trial onset. It doesn't indicate the time when Riluzole use started, only when it was recorded]

Adverse Events

Adverse events are all events recorded in the patients' clinical records during the trial, form bruises and headaches to stroke. These events may or may not be related to the treatment at hand. They are further described by their severity [**Severity**] and their outcome [**Outcome**] of the adverse event.Information about the events is structuredin the form of ahierarchical representation, where the levels in the hierarchy, listed from most specific to most generic, are named **Lowest_Level_Term**, **Preferred_Term**, **High_level_term**, and**High_Level_Group_Term**. In the representation of adverse events, we use the High_Level_Group_Term as feature name, i.e. this represents the level of granularity by which information on adverse events can be queried. Additionally, the adverse event's start time [**Start_Date_Delta**] and end time [**End_Date_Delta**] is given.

Note that while the general data format for adverse events is the same as the rest of the data, the feature value and delta columns are more complex. The feature value represents five pieces of information, namely Lowest_level_term, Preferred_term, High_level_term, Severity and Outcome. The delta column represents start delta and stop delta. Unit is left blank as it is not applicable for adverse events:

PatientID|Adverse Event|High_level_Group_term|Lowest_level_term;
Preferred_Term;High_level_term;Severity;Outcome |Unit|Start Delta;Stop Delta

Lab data

For each lab test there is [**lab_test_name, lab_test_value, lab_test_unit, delta_days_visit**(time from the start of the trial)]. Note that a lab test result can be within the normal range and may still be relevant for predicting ALS progression (depending on where it falls within the normal range), and also that normal ranges vary according to different sources. Also note that there may be cases where mistakes were made in data entry leading to abnormally high or low (non-physiological) levels of certain measures in this database, so be mindful of this in your analysis and interpretation.

As part of our data cleaning process for the lab data, units were converted, synonymous tests appearing with different names were merged. Note the some of the rare features are only available in the training set but not in the validation set (a list is available below)

Lab test data include:
Urine:

- Urine pH- the level of acidity of the urine. Levels range between 4.5 to 8 (optimal is 6).

- Urine Protein- detects excessive protein escaping into the urine, to help evaluate and monitor kidney function, and to detect kidney damage. Normal range is 0-20 mg/dL. Note that in the database non-physiological values are most likely due to mistakes in data entry .
- Urine Specific Gravity- (sometimes listed as Specific gravity) relates to the degree of concentration of the urine, indicative of kidney function. Normal ranges are 1-1.03.
- Urine Glucose- levels of glucose in urine (measured by mg/dL). Normally, they should be zero.
- Urine WBC (white blood cells)- Should be negative. Presence may indicate higher than normal activation of the immune system (such as in the case of infection).
- Urine Leukoesterase- measuring specifically leukocyte WBC's in the blood- should be <10 U/L.
- Urine Blood- measure of hemoglobin. Should be negative.
- Urine RBCs (red blood cells)- measure of bleeding. Should be < 3.0.
- Urine casts- another measure of bleeding. Should be negative.
- Urine Ketones- the levels of Ketone bodies found in urine, indicating starvation or carbohydrate deprivation leading to protein breakdown. Should be negative.
- Items regarding Urine Appearance include [Urine appearance, Urine Color, Urine Clarity
- Items regarding infection in Urine include [Urine Bacteria, Urine Culture and Urine Mucus].

Other measures include availability of extracts in Urine:[Urine Albumin, Urine bilirubins, Urine Hemoglobin, Urine Glucose, Urine Urobilinogen, Urine Urea, Urine Uric Acid, Urine Uric Acid Crystal, Urine Crystals, Urine Calcium Oxalate Crystals, Urine Amorphous Crystals, Urine Nitrite, Urine Potassium, Urine Sodium. (Note that some measures are listed only as – or as 'Normal' or on the scale of 'Trace', 'Small', 'Moderate' and 'Large; without any precise numerical value).

Blood

Blood proteins:

- Albumin- a small protein produced in the liver, is the major protein in blood serum. Used to assess liver disorder or kidney disease or to evaluate nutritional status. Both increases and decreases can be significant. Normal ranges are 35- 50 g/L.
- Protein- A measure of all blood protein including Albumin. Used to assess nutritional status or to screen for certain liver and kidney disorders. Both increases and decreases can be significant. Normal ranges are 60 – 84 g/L.

Electrolytes: Measured in an electrolyte panel to assess electrolyte levels found in disease and nutritional imbalances. The concentrations of sodium and potassium are tightly regulated by the body, as is the balance among sodium, potassium, chloride, and bicarbonate. Electrolyte (and acid-base) imbalances can be present with a wide variety of acute and chronic illnesses. Both increases and decreases can be significant.

- Sodium – Abnormal levels are associated with kidney malfunction and many other pathophysiological changes. Normal ranges are 133 - 146 mmol/L (note that values are sometimes reported as lower than physiologically reasonable beyond reasonable range).
- Potassium- Normal ranges are 3.5 - 5.4 mmol/L (note that values are sometimes high beyond reasonable range).
- Bicarbonate (CO_2)- Associated also with acid-base (pH) imbalance. Normal ranges are 18 - 23 mmol/L.

- Chloride- Associated also with acid-base (pH) imbalance. Normal ranges are 98 - 106 mmol/L (Note that some measures are listed only as –, without any precise numerical value).
- Anion Gap: The balance of anions and cations. Normal ranges are <11 mmol/L. If the gap is greater than normal, then high anion gap metabolic acidosis is diagnosed.
- Magnesium- measures to assess to likelihood of magnesium, poisoning. Normal ranges are or 0.6-0.82 mmol/L.

Kidney Tests: BUN and creatinine are waste products filtered out of the blood by the kidneys. Increased concentrations in the blood may indicate a temporary or chronic decrease in kidney function.

- Blood Urea Nitrogen (BUN), also known as Urea. This is a product of the kidney's normal function found in urine. Normal ranges are 1.2-3 mmol/l. (note that values are sometimes high beyond reasonable range).
- Uric Acid- Digestive product dissolved by the kidneys. High levels might indicate kidney dysfunction or other pathophysiological changes (like gout) and are generally thought to be unhealthy within themselves. Normal ranges are 180-480 umol/L. Note that low levels of uric acid have been shown to predict worse prognosis in ALS and Uric acid is the only lab test currently known to be related to prognosis in ALS.
- Creatinine- normal ranges are 53-106 mmol/L for males. Normal BUN/ Creatinine ratios are 5-35.(note that values are sometimes high beyond reasonable range).

Liver Tests: ALP, ALT, GGT and AST are enzymes found in the liver and other tissues. Bilirubin is a waste product produced by the liver as it breaks down and recycles aged red blood cells. All can be found in elevated concentrations in the blood with liver disease or dysfunction.

- Alkaline phosphatase (ALP)- also listed as SPGT. Also associated with bone dysfunction. Normal ranges 50 - 160 U/L.(note that values are sometimes high beyond reasonable range and that some measures are listed only as –, without any precise numerical value).
- ALT (alanine amino transferase, also called SGPT)- Also associated with diseases of the biliary system. Normal ranges are 1 - 21 U/L.(note that values are sometimes high beyond reasonable range and that some measures are listed only as –, without any precise numerical value).
- Gamma-glutamyltransferase-(GGT)- Elevated serum GGT activity can be found in diseases of the liver, biliary system, and pancreas. Normal ranges are 5 - 40 U/L.
- AST (aspartate amino transferase, also called SGOT). Normal ranges are 7 - 27 U/L.(note that values are sometimes high beyond reasonable range and that some measures are listed only as –, without any precise numerical value).
- Bilirubin (Total, Direct and Indirect)- Also associated with Anemia. Normal ranges are -5-17 umol/l for Bilirubin (Total=direct+indirect), 1-5 umol/L for bilirubin (Direct) and 4 -12 for Bilirubin (Indirect).

Complete blood count: used as a broad screening test to check for such disorders as anemia, infection, and many other diseases.

- White Blood Cell (WBC)- a count of the actual number of white blood cells per volume of blood. Both increases and decreases can be significant. Normal ranges are 4.3-10.8 *109/L cells.
- White blood cell differential looks at the types of white blood cells present. There are five different types of white blood cells, each with its own function in protecting us from infection. Quantities of the various white blood cell types are listed below as either percentage or volume:

- Neutrophils- also named Segmented Neutrophils. Normally the most abundant type of white blood cell in healthy adults, important in fighting inflammation. Normal ranges are 1.3-5.4 *109/L (Absolute Neutrophil Count) cells or 45-62% (Neutrophils).Note that some measures are listed only as – or as 'Normal', without any precise numerical value.
- Band Neutrophils- are important in inflammation. Normal ranges are 0-0.7*109/L cells (Absolute Band Neutrophil count)or 3-5%(Band Neutrophils). Note that some measures are listed only as – or as 'Normal', without any precise numerical value.
- Lymphocytes- make up about 25% of the total white blood cell count but can vary widely. Lymphocytes occur in two forms: B cells, which produce antibodies, and T cells, which recognize foreign substances and process them for removal. Normal ranges are 0.7-3.9 *109/L cells (Absolute Lymphocytes Count) or 16-33% (Lymphocytes). Note that some measures are listed only as – or as 'Normal', without any precise numerical value.
- Monocytes - function in the ingestion of bacteria and other foreign particles. Monocytes make up 5-10% of the total white blood cell count. Normal ranges are 0.1-0.8 *109/L cells (Absolute Monocyte Count) or 3-7%(Monocytes).Note that some measures are listed only as – or as 'Normal', without any precise numerical value.
- Eosinophils- are believed to function in allergic responses and in resisting some infections. Normal ranges are 0-0.5 *109/L cells (absolute Eosinophil count) or 1-3% (Eosinophils). Note that some measures are listed only as –, without any precise numerical value.
- Basophils- normally constitute 1% or less of the total white blood cell count but may increase or decrease in certain diseases. Normal ranges are 0-0.4*109/L (Absolute Basophil Count) or 0-0.75%(Basophils). Note that some measures are listed only as – or as 'Normal', without any precise numerical value.

- Red Blood Cells (RBC) - a count of the actual number of red blood cells per volume of blood. Both increases and decreases can point to abnormal conditions. Normal ranges are 4.2 - 6.9 *109/L.
- RBC (red blood cell) Morphology- Abnormal morphology is found in certain blood diseases such as sickle-cell anemia.
- Hemoglobin - measures the amount of oxygen-carrying protein in the blood. Lower levels are associated with Anemia. Normal ranges are Male: 130 - 180 g/L; Female: 120 - 160 g/L.
- Hematocrit - measures the percentage of red blood cells in a given volume of whole blood. Normal ranges are Male: 45%-62%; Female: 37%-48% (out 100%).
- Mean Corpuscular Hemoglobin Count-a measure of concentration of Hemoglobin in a given volume of red blood cells (calculated by Hemoglobin/Hematocrit). Normal levels are 320-360 g/L or 32-36%.
- Mean Corpuscular Volume- Average red blood cell volume. Normal levels are 80-99 fL.
- Mean Corpuscular Hemoglobin- Average levels of hemoglobin per red blood cell. Normal levels are 27-31 pg/cell.
- Platelets- the number of platelets in a given volume of blood. Both increases and decreases can point to abnormal conditions of excess bleeding or clotting. Normal ranges are 150-350 *109/L cells (note that values are sometimes high beyond reasonable range).

- Transferrin iron-binding blood plasma glycoproteins that control the level of free iron in the blood. Both abnormally high and abnormally low levels may be indicative of Anemia. Normal ranges are 204–360 mg/dL

Heart disease and muscle degradation

- CK (Creatine Kinase)- Increases may indicate a heart attack or other muscle damage. Normal ranges are Male: 38 - 174 u/L; Female: 96 - 140 u/L (note that values are sometimes high beyond reasonable range, and that some measures are listed only as – or as 'Normal', without any precise numerical value).
- Triglycerides- measured to assess the risk of developing heart disease, with increased triglyceride levels correlating with increased risk. Normal ranges depend on age: Ages 10-39 0.61-1.3 mmol/L; ages 40-59 0.77-1.7 mmol/L; age 60+ 0.9-1.7 mmol/L. Generally recommended to be kept <1.1 mmol/L (note that values are sometimes high beyond reasonable range).
- Total Cholesterol- measured to assesses the risk of developing heart disease, with increases in cholesterol correlating with increased risk. Normal ranges are 3-5 mmol/L, recommended to be kept no higher than 3.9 mmol/L.
- Lactate dehydrogenase- an enzyme involved in tissue breakdown, most commonly heart muscle damage, but also other tissues. Normal ranges are 50-150 U/L.

Blood sugar

- Glucose-level of glucose in the blood. Both increased and decreased levels can be significant. Normal ranges are 3.8-6 mmol/L (Note that some measures are listed only as Trace, Small, Moderate or Large, without any precise numeric value).
- HbA1c (Glycated Hemoglobin)- is a form of hemoglobin that is measured primarily to identify the average plasma glucose concentration over prolonged periods of time. It is formed in a non-enzymatic glycation pathway by hemoglobin's exposure to plasma glucose. Units are percentages. Level ≥ 6.5% serves as a criterion for the diagnosis of diabetes (Note that some measures are listed only as 'Normal', without any precise numeric value).

Mineral balance

- Calcium- routine metabolic panel to assess kidney, bone, or nerve disease. Both increased and decreased levels can be significant. Normal ranges are 2.2-2.5 mmol/L (note that values are sometimes high beyond reasonable range).
- Phosphorus –related to level of Calcium. Associated with kidney function, nutritional status, and a variety of chronic illnesses. Both increased and decreased levels can be significant. Normal ranges are 1-1.5 mmol/L.(Note that some measures are listed only as –, without any precise numerical value)

Immune response

- Hepatitis - A,B,C antigen and antibody. Positive antibody levels indicate vaccination. Positive antigen levels indicate that the person is more infectious.
- Immunoglobulins (Immunoglobulin A, G, M)-antibody isotypes. IgG isthe major one found in blood. Normal range: for Immunoglobulin A 85-385mg/dL, for Immunoglobulin G 565-1765 mg/dL and for Immunoglobulin M 55-375 mg/dL.
- Gamma Globulin- a class of globulin of which Immunoglobulin G is the most common. Normal levels are 2-3 g/dL. Indicative of inflammation.

- Hormones
- TSH (Thyroid Stimulating Hormone)- A hormone that stimulates the thyroid gland to produce factors which stimulate the metabolism of almost every tissue in the body. Both increased and decreased levels can be significant. Normal ranges are 0.4-3 U/L.
- Free T3- activated by TSH. Normal ranges are 3.1-7.7 pmol/L.
- Free T4- activated by TSH. Normal ranges are 9-18 pmol/L.
- Beta HCG- a hormonal marker of pregnancy. Should be <5 U/L in non-pregnant pre-menopausal women, and <9.5 U/L in post-menopausal women.

Coagulation measures

- Prothrombin time (clotting). Normal ranges are 10-13 sec.
- International normalized ratio (INR). Normal ranges are 0.8-1.2.

Others

- Amylase- an enzyme involved in breaking down of starch. Increase levels indicate a variety of digestive problems, most commonly pancreas inflammation and ulcers. Normal ranges are 30-110 U/L.
- Salivary Amylase- Percentage of amylase that is Salivary by nature.
- Pancreatic Amylase- Percentage of amylase that is Pancreatic by nature. Pancreatic and Salivary Amylase should add up to 100%

National Registry data description

The data contains information from ALS patients in the community, monitored by ALS clinics and Italy (from t**he Piemonte and Valle d'Aosta Register for ALS**) and Ireland (from the Irish National ALS register). The data is de-identified to protect patient privacy. Different types of information may be available for different patients because the data was generated in multiple clinical trials and clinics.

Each subject is identified by a SubjectID and the specific assessment for this subject is identified by a record (each subject has multiple records). The assessments are separated into different files according to type:

- ALSFRS(R)
- Death Report
- Demographics
- Family History
- Subject ALS history
- Medical History
- Forced Vital Capacity
- Riluzole use
- Vital Signs

- Genetic testing
- Cognitive tests

The time at which an assessment was taken (a record was created) is listed as the assessment's delta. Delta is given as days since the first visit to the clinic. A negative delta lists events occurring before the official beginning of the measurement (for example symptom onset would always have negative delta as they predate diagnosis as ALS patients).

In the data file you will find a SubjectID for each patient, indicating which subject it is. You will find the same SubjectID across different data types and different assessments e.g. if the same patient had both vital signs and lab tests measured, those respective records will include the same SubjectID.

Beyond the SubjectID, the data contain different assessments and their respective results including data types. Specific measure, value, unit of measurement, and delta (time in days from clinic visit onset when the assessment was made). You can identify these variables through the column name in the data file.

The data format lists:

SubjectID|form_name|feature_name|feature_value|feature_unit|feature_delta

For example: I858|ALSFRS|Q8_Walking|4|NA|55

Patient I858 had, at delta= 55 (day 14 from beginning of measurement), ALSFRS Q8==4, no unit of measure.

Demographics

Demographic information is available in the Demographics datatype, including [**Age, Gender,**] at first clinic visit (time 0).

Subject ALS history (ALSHX)

The major symptoms of ALS broadly include muscle weakness, paralysis, drooling, gagging, muscle cramps, involuntary muscle contractions/twitches called fasciculations, speech problems, significant weight loss ("wasting"), and breathing problems. ALS typically does not affect the senses (sight, smell, taste, hearing, touch). Specific symptoms of patients are listed in the data- in the datatype Subject ALS History- as [**Onset Site** (which body part is manifesting the symptom] and [**ALS_First_Symp**].

As ALS progresses, patients lose their ability to control voluntary muscle function. Symptoms progress from muscle weakening, twitching, and an inability to move the arms, legs, and body, into full paralysis. When the muscles in the chest area stop working, it becomes hard or impossible to breathe on one's own.

The site of disease onset as experienced by the patient can be a limb (“limb onset”) or the muscles controlling speaking and swallowing (“bulbar onset”) or occasionally both. Information is available in the database regarding a given patient’s site of onset (Limb/Bulbar/Limb and Bulbar). ALS first symptoms options are listed here:

Lower limb-left, and Lower limb-right
Lower limb-right
Respiratory
Upper limb
Upper limb-left
Upper limb-left, and Upper limb-right
Upper limb-right
Weight loss
Bulbar-speech, and Bulbar-swallow
Bulbar-speech, and Weight loss
Bulbar-swallow
Cognitive/behavioural
Cognitive/behavioural , Lower limb-left, and Lower limb-right
Cramping and Lower limb
Cramping, and Lower limb-right
Cramping, and Upper limb-left
Cramping, and Weight loss
Cramping, Fasciculation, and Upper limb
Cramping, Fasciculation, Fatigue, and Lower limb-left
Head drop
Lower limb
Lower limb-left
Bulbar

Bulbar-speech

Over time, the disease progresses to other sites. An ALS diagnosis is currently confirmed only when symptoms appear in more than one site. The gap in time between onset of symptoms and diagnosis is on average more than a year. The time from disease onset, time from diagnosis, or time from both is available for many of the patients [**Onset Delta**- the time between disease onset and the first time the patient was tested in a trial; **Diagnosis Delta**- the time between clinical diagnosis and the first time the patient was tested in a trial; the difference between these two values is the time between onset and diagnosis]. The status of diagnosis is available as [**Diag_stat**] and could (Definite/Suspected/ Possible/ Probable).

For some patients detailed information is available regarding any intervention relating to the patient disease status. These include breathing interventions such as noninvasive ventilation [NIV] and the number of hours it is used [**NIV_hours_night, NIV_hours_day, NIV_hours_ total**], Feeding intervention, gastrostomy- the insertion of a feeding tube [**PEG**- percutaneous endoscopic gastrostomy or **RIG**- radiologically inserted gastrostomy], salivation intervention [**Botox** or **Radiation**], Shoulder pain [**Shoulder_Pain, Shoulder_Pain_Rest, Shoulder_Pain_Night, Shoulder_Pain_function** and **Shoulder injection**] and palliative care [**Pallative**], as well as the use of Riluzole [**if_use_Riluzole**, available in the datatype Riluzole].

For some patients, detailed information exists about the use of assistive technology, including Walking aids, communication aids, Respiration aids and posture aids. The options are listed here:

**Walking aids**	**Communication aids**	**Respiratory aids**	**Posture aids**
Stick/crutch, Frame/Rollator, and Wheelchair/Scooter	Tablet computer and Eye Gaze	Suction machine	Soft collar
Stick/crutch, Frame/Rollator, and Splints	Tablet computer	Cough assist machine and Suction machine	Neck brace/Headmaster and Soft collar
Stick/crutch, Frame/Rollator, and Ankle Foot Orthosis	Pen and paper, Lightwriter, and Tablet computer	Cough assist machine and Breath stacking	Neck brace/Headmaster

Stick/crutch, Ankle Foot Orthosis, Wheelchair/Scooter, and Frame/Rollator	Pen and paper and Tablet computer	Cough assist machine	
Stick/crutch, Ankle Foot Orthosis, Splints, Frame/Rollator, and Wheelchair/Scooter	Pen and paper and Lightwriter	Breath stacking, Cough assist machine, and Suction machine	
Stick/crutch, Ankle Foot Orthosis, Splints, and Wheelchair/Scooter	Pen and paper	Breath stacking and Suction machine	
Stick/crutch, Ankle Foot Orthosis, Splints, and Frame/Rollator	Lightwriter and Pen and paper	Breath stacking and Cough assist machine	
Stick/crutch, Ankle Foot Orthosis, Frame/Rollator, Wheelchair/Scooter, and Splints	Lightwriter	Breath stacking	
Stick/crutch, Ankle Foot Orthosis, Frame/Rollator, and Wheelchair/Scooter	Eye Gaze and Tablet computer		
Stick/crutch, Ankle Foot Orthosis, and Wheelchair/Scooter	Eye Gaze		
Stick/crutch, Ankle Foot Orthosis, and Frame/Rollator			
Stick/crutch and Wheelchair/Scooter			
Stick/crutch and Splints			
Stick/crutch and Frame/Rollator			
Stick/crutch and Ankle Foot Orthosis			
Ankle Foot Orthosis			
Splints and Ankle Foot Orthosis			
Splints and Frame/Rollator			
Splints and Stick/crutch			
Splints and Wheelchair/Scooter			
Splints, Frame/Rollator, and Ankle Foot Orthosis			
Splints, Stick/crutch, and Ankle Foot Orthosis			

Splints, Stick/crutch, and Frame/Rollator			
Stick/crutch			
Stick/crutch, Ankle Foot Orthosis, and Splints			
Ankle Foot Orthosis, Frame/Rollator, and Wheelchair/Scooter			
Ankle Foot Orthosis, Frame/Rollator, and Stick/crutch			
Ankle Foot Orthosis and Wheelchair/Scooter			
Ankle Foot Orthosis and Stick/crutch			
Ankle Foot Orthosis and Splints			
Ankle Foot Orthosis and Frame/Rollator			
Ankle Foot Orthosis, Splints, and Frame/Rollator			
Ankle Foot Orthosis, Splints, Frame/Rollator, and Wheelchair/Scooter			
Ankle Foot Orthosis, Stick/crutch, and Splints			
Ankle Foot Orthosis, Stick/crutch, and Wheelchair/Scooter			
Ankle Foot Orthosis, Stick/crutch, Frame/Rollator, and Wheelchair/Scooter			
Ankle Foot Orthosis, Wheelchair/Scooter, and Stick/crutch			
Stick/crutch, Frame/Rollator, Wheelchair/Scooter, and Ankle Foot Orthosis			
Stick/crutch, Frame/Rollator, Wheelchair/Scooter, and Splints			
Stick/crutch, Splints, and Ankle Foot Orthosis			
Stick/crutch, Splints, and Wheelchair/Scooter			
Stick/crutch, Splints, Frame/Rollator, and Wheelchair/Scooter			
Stick/crutch, Splints, Frame/Rollator, Wheelchair/Scooter, and Ankle Foot Orthosis			
Wheelchair/Scooter			
Wheelchair/Scooter and Ankle Foot Orthosis			
Wheelchair/Scooter and Frame/Rollator			

Medical History (MEDHx)

For some patients, information is available about co-occurrence of other diseases such as [**Diabetes], [Thyroid] and [Hypertension]** in the form **MedHx**. For the cases of diabetes and hypertension, those that have it are listed as Y and those that do not as N. For the case of Thyroid, the options are Hypo (Hypothiroidism), Hyper (Hyperthyroidism), HASHI (Hashimoto's thyroiditis) or N (no Thyroid dysfunction). For diabetes If the information isn't available for a specific patient than it indicates that the information is unavailable. For other patients, information about co-morbidities with other neurological conditions [**MedHx_Neuro**] is available. Potential neurological conditions include (Other Neurological/ Multiple Sclerosis/ Depression/ Bipolar Disorder /Alcoholism).

<u>Family History and genetic testing</u>(FamilyHx, Mutation)

ALS affects approximately 5 out of every 100,000 people worldwide. In about 5-10% of cases, ALS is seen in multiple family members and this form is known is 'familial ALS'. Multiple mutations (over 35) have been identified for such patients and some information about them is available here. In the remaining cases, called sporadic cases, there is no more cases observed in the families (and only a few of these patients are carriers of the mutations identified in the familial cases).

Information about the mutations is available in the datatype GENETICS, in the feature [**Mutation**]. For some patients several mutations were assessed: SOD1, TARBDP, OPTN, MATRIN, FUS, ATAXIN, C9ORF72. If mutations were not assessed, the value is "Unavailable", if none of these mutations were assessed, the patient is listed as WT (wild type). For other patients only the status of C9orf72 mutation was assessed. In that case, the patients can be listed as C9orf or C9orf Negative. The status of other mutation is unavailable**.**

Family History (FamilyHx) contains information whether a patient has a family history of ALS [**family_ALS_hist]** - Those that do are listed a Y. The list is expected to be inclusive so that if a patient is not listed as Y (listed as NA or N), family history of ALS was not observed.

If there is family history of ALS, there is sometimes a compound feature called ALS_Type:

SubjectID|FamilyHx|ALS_Type|Type of ALS;relativeID,certainty of family history|unit|delta

SubjectID|FamilyHx|ALS_Type|ALS/ALSFTD;familymemberID,Certain/likely|unit|delta

The type can be ALS or ALSFTD (ALS with frontotemporal dementia, affecting decision making and executive functions, see more below), the second part of the compound feature is the SubjectID of the affected family member (most should be available in the data given that this is a national registry). The third part of the compound feature is whether the ALS diagnosis of the family member is Certain or likely. In this case, if information about ALS_type is not available, it was not assessed, so it is unknown which type of ALS the affected family member had.

Another type of family information contained in the FamilyHx data type is information about other Neurological conditions in the family [**FamilyHx_Neuro_Other_Type**], and this is again a compound feature:

SubjectID|FamilyHx|FamilyHx_Neuro_Other_Type| Type of neurological/neuropsychiatric disease; Relationship to patient; Degree of relatedness; Gender|Unit|delta

For example:

SubjectID|FamilyHx|FamilyHx_Neuro_Other_Type| Dementia; parent; First; Female |NA |delta

The first part of the compound feature is type of neurological or neuropsychiatric condition (list below), the second part is the relationship to the patients (list below), the third is the degree of relatedness (first degree, second or third degree relatives) and the fourth part is the gender of the family member.

Family members assessed are: Parent, Sibling, Grandparent, Aunt/Uncle, Child, Cousin, Grand-aunt, Grand-Uncle, Grandchild, Grand-niece/Grand-nephew, Niece/Nephew

Neurological condition assessed are:

Suicide and Depression
Suicide
Schizophrenia
Parkinson's Disease and Schizophrenia
Parkinson's Disease
Other Neuropsychiatric
Other Neurological and Depression
Other Neurological
Multiple Sclerosis
FTD
Depression and Suicide
Depression and Schizophrenia
Depression and FTD
Depression
Dementia and Parkinson's Disease
Dementia and Depression
Dementia
Bipolar Disorder
Autism
Alzheimer's Disease and Dementia
Alzheimer's Disease
Alcoholism, Other Neuropsychiatric, and Depression
Alcoholism, Depression, and Suicide

Alcoholism, Dementia, and Parkinson's Disease
Alcoholism and Parkinson's Disease
Alcoholism and Other Neuropsychiatric
Alcoholism and IVDA
Alcoholism and Depression
Alcoholism and Alzheimer's Disease
Alcoholism

<u>Symptoms and outcome measures (FVC, ALSFRS, and Survival datatypes)</u>

Symptom severity is frequently assessed using two functional scales: ALSFRS (ALS Functional Rating Scale) and its modified version ALSFRS-R. The ALSFRS scale is a list of 10 assessments regarding motor function, with each measure ranging from 0 to 4, with 4 being the highest (normal function) and 0 being no function. The score for the individual questions are then summed together to generate a number, and that is the ALSFRS score.

ALSFRS-R is a modified version of the ALSFRS. Whereas in the ALSFRS there are 10 assessments, in the ALSFRS-R one of the assessments, #10 (respiratory function) was further divided into three questions to better reflect the importance (weighting) of respiratory changes within the scale. Therefore ALSFRS-R, contains 12 questions (9 of these identical to the traditional ALSFRS) and a maximal score of 48. Please note that some of the patients in the dataset will have ALSFRS scores and some will have ALSFRS-R. ALSFRS and ALSFRS-R information is available in the datatype ALSFRS(R).

The individual questions comprising the ALSFRS or ALSFRS-R scores are available in challenge description [**ALSFRS Speech, Salivation, Swallowing, Handwriting, Cutting (with and without Gastrostomy)** (gastrostomy is a feeding tube), **Dressing and Hygiene, Turning in Bed, Walking, Climbing Stairs, Respiratory**]. In [**ALSFRS-R Speech, Salivation, Swallowing, Handwriting, Cutting (with and without Gastrostomy), Dressing and Hygiene, Turning in Bed, Walking, Climbing Stairs, Dyspnea, Orthopnea, Respiratory Insufficiency**]. The total sum is available as [**ALSFRS Total, ALSFRS-R Total**]. The time between the first time a patient was observed (Time 0) and the time of each assessment of ALSFRS or ALSFRS-R over the course of the trial is listed as [**ALSFRS-Delta** (regardless of whether it was ALSFRS or ALSFRS-R)].

Due to the limitation on number of features to be used in this challenge, we added several composite scores combining several intercorrelated ALSFRS questions: Q1-3 are [**mouth**], Q4-5 are [**hand**] Q6-7 are [**trunk**], Q8-9 are [**leg**] and either Q10 or R1, whichever is available, is [**respiratory**]. For ALSFRS-R there is also the composite score [**respiratory-R**], combination of questions R1-3.

The file ALSFRS slope includes the gold standard for slope prediction for the training set.

Another tool to assess disease progression is the ALS staging system, available as part of the datatype ALSHx. In this system, the patient gets a score of 0/1 for the following four questions [**ALS_Staging_Move,**

ALS_Staging_Eat, ALS_Staging_Breath, ALS_Staging_Com] and a total score [**ALS_Staging_Total**] ranging between 0 to 4

In addition to ALSFRS, there is another frequently used measure of ALS disease status called forced vital capacity or **FVC**. Forced vital capacity is the volume of air that can forcibly be blown out after full inspiration, measured in percentage of vital capacity out of expected normal vital capacity [**fvc_ercent**] derived by dividing the patient capacity with that expected by an average individual of that age, gender (so 120% is an athlete, 100% is normal, 80% is deteriorating, and 50% is very low breathing capacity/ready for a ventilator).

Finally, time of death is available- in the file Survival Response- whether the subject died [**Status; 1=died**] while monitored and if that is =1 (indicating the subjected indeed died), also the time of death, measured in days from trial onset [**time_event**]. For the subjects that didn't died (status=0, either indicated by the trial managers or by the last time the patient was assessed).

<u>Vital signs</u>

Weight (in kg) [**Weight**] and SNIP [**SNIP-] An alternative or additional test of inspiratory muscles strength is maximal sniff nasal inspiratory pressure**, units are cm H2O

<u>Cognitive status</u>

Some ALS patients have accompanying cognitive and behavioral changes associated with teir disease. If severe, these changes can lead to a diagnosis of ALSFTD: ALs with Frontotemporal dementia, a dementia that affects primarily executive functions, leaving other cognitive functions such as memory intact. For some patients information about cognitive status is available in the datatype COG.

One feature is whether the patients were assessed for cognitive changes. One possible assessment tool is the full cognitive battery [**Full Battery**], administered by a neuropsychologist. Another possible assessment tool is the Edinburgh Cognitive ALS Screen [**ECAS**]. For the full battery the possible value is Yes (in case used), but for the ECAS,there is a compound feature including three parts: ECAS_Non_Spes_Score; ECAS_Spes_Score; ECAS_Version. The first part is the score for the non FTD specific items, the second is for the FTD specific items and the third is which version of the ECAS was used.

The cognitive status assessed is marked as [**Cog_Stat**], with (Normal/Executive changes) as possible values. The behavioral status assessed is marked as [**Behav_Stat**], with (Normal/Abnormal) as possible values. If a dementia was observed, the type of dementia is listed as [**Dementia_Type**] which could be (Behavioral FTD/Alzheimer's disease).

Supplementary material part 2- Challenge information as given to participants

Amyotrophic lateral sclerosis, or ALS (also known in the US as Lou Gehrig's Disease and as Motor Neuron Disease in the UK) is a fatal neurodegenerative disease that involves the degeneration and death of the nerve cells in the brain and spinal cord that control voluntary muscle movement. This leaves patients struggling with a progressive loss of motor function while typically leaving cognitive functions intact. Death typically occurs within 3 - 5 years of symptom onset. Only about 25% of patients survive for more than 5 years after diagnosis and only 10% will survive over 10 years. The root cause of the disease is still unknown. ALS will kill one in 1000 individuals, and most common age of onset is 40-60. The ALS Stratification Challenge is an open crowdsourced analysis effort to find predictors of disease progression that can be used to aid clinical care, identify new disease predictors and potentially significantly reduce the costs of future ALS clinical trials.

Predicting disease prognosis and ALS Challenges

The average life span of an ALS patient is two to five years. However, approximately 10% of patients live significantly longer owing to a much slower disease progression. An example of typical/fast disease progression is Lou Gehrig, who lived 2 years following diagnosis; the astrophysicist Stephen Hawking, who has lived with ALS for the last 49 years, is an example of a slow progressor. In this challenge we aim to identify subgroups of ALS patients that differ in their disease progression and survival, and the predictive features for these patients. No risk factors have been conclusively identified for developing ALS other than having a family member who has a hereditary form of the disease (which is the case for only ~10% of the patients). There is no known cure for ALS. The only FDA-approved drug for the disease is Riluzole, which has been shown to prolong the life span of ALS patients by a few months. Several predictors of prognosis have already been identified and are listed below.

The success of the 2012 ALS Prediction Challenge

In 2012, Prize4Life and DREAM launched a first challenge called the **DREAM Phil Bowen ALS prediction Prize4Life challenge**, which used the first iteration of the PRO-ACT dataset. That first Challenge asked solvers to predict the rate of ALS disease progression for individuals and featured a 25,000 winner's prize to incentivize participation (later increased to 50,000). Algorithms were developed and evaluated in a statistically rigorous blinded assessment. The Challenge drew over 1,000 registrants from 63 countries and led a meaningful impact for both clinical trials and disease understanding. With respect to the clinic, the Challenge's best performing algorithms were able to reduce by 20% the number of patients needed for effective ALS clinical trials: such a reduction translates to millions of dollars saved for future clinical trials that leverage the winning algorithms. Furthermore, the Challenge's winning algorithms identified novel features of ALS pathology that are predictive of disease progression. Results from this Challenge are described in the 2015 Nature biotechnology publication.

While the ALS Prediction Prize brought in significant benefit for the ALS community, it focused on predicting the disease progression over time for the entire population of ALS patients. As it turned out, such models typically performed best for the "average" patient but were less able to predict the disease course of very slow or fast progressing patients. Prediction for the entire population is therefore limited by the inherent heterogeneity of the ALS manifestation. Therefore, with the **DREAM ALS Stratification Prize4Life Challenge** (ALS Stratification Challenge) we now aim at the development of tools that accurately assign

individual patients to specific sub-groups of patients with clear clinical implications for either survival or disease progression. There are good reasons to believe that there are identifiable, meaningful subgroups of ALS patients within the population that are more homogenous, but have not been fully characterized yet. Understanding what differentiates such subgroups is of great research and clinical interest. The challenge is based on the so far largest collection of ALS clinical trial datasets contributed by Prize4Life. It leverages DREAM's crowdsourcing expertise, community of researchers, and SB software. The challenge further builds on the Synapse platform for computational and Challenge infrastructure, secure data storage, data access/distribution conditions, large-scale collaborative analysis and results sharing.

Why run a stratification challenge?

The goal of the ALS Stratification Challenge is to identify subgroups of patients with distinct clinical outcomes that can be distinguished by the clinical features. Challenge participants will create models to (1) cluster patients according to the outcome clinical targets, and (2) based on this classification, they will identify for each patient a small subset of predictive features and predict the outcome clinical targets. Performance will be assessed based on the quality of the predictions. We believe that the stratification algorithms generated in this Challenge have the potential to aid clinical care, identify new disease predictors that can lead to novel biomarker development, and potentially significantly reduce the costs of future ALS clinical trials in allow specialized treatments for specific subgroups of ALS patients.

Challenge Questions

The ALS Stratification Challenge will focus on identifying subgroups of patients with distinct clinical outcome targets that can be distinguished by specific clinical features. For predictions, algorithms are first required to select a patient specific subset of the available clinical features. Only these features (predictors) may then be used for subsequently predicting the clinical outcome. In addition, we ask the solvers to assign each patient into patient groups or clusters (there are no limits on the number of clusters). By an a posteriori analysis of these predictors and the assigned patient group we will determine novel patient subgroups, and analyze to which extent they represent ALS subtypes.

Participants are asked to use data collected from patients over a 3 month period to predict one of the following:

1. Disease progression, as measured using the ALS functional rating scale (ALSFRS), the score used for monitoring ALS patients. Participants are provided with ALSFRS measured between 0-3 months and are asked to predict the slope of ALSFRS changes between 3-12 months.
2. Survival, given as probability of death within a 0-12 months, 0-18 and 0-24 months from trial onset.

There are two data sources within this challenge - data collected within clinical trials from the PRO-ACT database and data collected directly from patients within a community through the Irish and Italian ALS Registries. The PRO-ACT data was made available at Challenge launch and the Registry data will be made available in July 2015. Because the data collected within these two sources are quite different, participants are asked to develop predictive models separately within each data source. Prizes will be awarded separately

to the top performing team for each of the following four sub-challenges. Participants may choose to submit predictions for any number of sub challenges.

Subchallenge 1: Predict 3-12 month ALSFRS slope using clinical trial data collected through the PRO-ACT database.

Subchallenge 2: Predict Survival using clinical trial data collected through the PRO-ACT database.

Subchallenge 3: Predict 3-12 month ALSFRS slope using community based data collected through the National Registries.

Subchallenge 4: Predict Survival using community based data collected through the National Registries.

Challenge Data

Data sources are the PRO-ACT database and two national registries. All data was collected between 1990 and 2015. The data was de-identified following the HIPAA de-identification conventions for personal health information: any potential patient initials and/or dates of birth were removed, new randomized subject numbers were created, and wherever possible, trial-specific information was removed in the merging of datasets, including trial center identity and location, trial dates, or other identifying information.

Several general notes about the data:

Longitudinal data: Note that the time at which an assessment was taken (a record was created) is listed as the assessment's delta. Delta is given as days since the trial onset. A negative delta lists events occurring before the official beginning of the measurement (for example symptom onset would always have negative delta as they predate ALS diagnosis). Patients are identified by a PatientID that is the same across different assessments e.g. if the same patient had both vital signs and lab tests measured, those respective records will include the same PatientID.

Format: Data is provided in a tabular format. Each line represents a single feature with several pipe-delimited columns. The columns in this tabular format specify the patient ID (column 1), the feature context (form name in column 2), the unique feature name (column 3), the feature value (column 4), and, if applicable, the feature's unit (column 5) and time delta in days (column 6).

Clinical Trial Data from PRO-ACT

Subchallenges 1 and 2 use clinical trial data collected through the PRO-ACT database. The training set is 7200 patients from PRO-ACT database that are already available open access. Predictions will be tested (on the test and validation data sets) using data collect from 1900 patients in 6 additional clinical trials not yet available in PRO-ACT. In order for the participants to assess differences between training and validation data, the training data also contains records for 200 patients taken from the validation set, provided in a separate file (note that some rare features might not be the same across datasets). All validation data will be placed into the public domain through PRO-ACT at the conclusion of this Challenge.

The PRO-ACT training data is provided in a .zip file containing six files.
File 1: Training Data- a subset of the PRO-ACT database (~7000 patients)

File 2: ALSFRS Slope Gold Standard containing the Dependent Variable to be Predicted in Subchallenge 1 (see description of the calculation below)

File 3: Survival Response Gold Standard containing the Dependent Variable to be Predicted in Subchallenge 2 (see description of the calculation below)

File 4: Additional Training Data a subset of the validation (~200 patients)

File 5: ALSFRS Slope Gold Standard for Additional Training Data

File 6: Survival Response Gold Standard for Additional Training Data

Community based Data from National Registries

Sub-challenges 3 and 4 will use community-based data collected through two national registries:
1) The Irish National ALS Register including data, currently unreleased, collected from ALS clinics in Ireland.
2) The Piemonte and Valle d'Aosta Register for ALS, including data, currently unreleased, collected from ALS clinics in Piemonte and Valle d'Aosta region of Italy.

The data from the two registries were merged, harmonized and converted to the same format as the PRO-ACT data. The training set is 986 patients and the validation set includes 493 patients. The training and validation set were stratified regarding the number of patients from the Irish and Italian registry but otherwise randomly divided. All validation data will be placed into the public domain through PRO-ACT at the conclusion of this Challenge.

The Registry training data is provided in a .zip file containing thee files.

File 1: Training Data- a subset of the registries database (986 patients)

File 2: ALSFRS Slope Gold Standard containing the Dependent Variable to be Predicted in Sub-challenge 3 (see description of the calculation below, 452 patients)

File 3: Survival Response Gold Standard containing the Dependent Variable to be Predicted in Sub-challenge 4 (see description of the calculation below, 966 patients)

Clinical Targets

The goals for subchallenge 1,3 are predicting ALSFRS slope, and for subchallenges 2,4 predicting survival.

Predicting ALSFRS Slope

The Amyotrophic Lateral Sclerosis Functional Rating Scale (ALSFRS) is an instrument for evaluating the functional status of patients with Amyotrophic Lateral Sclerosis. It is used to monitor functional change in a patient over time.

Symptom severity is frequently assessed using two functional scales: ALSFRS (ALS Functional Rating Scale) and its modified version ALSFRS-R. The ALSFRS scale is a list of 10 assessments regarding motor function, with

each measure ranging from 0 to 4, with 4 being the highest (normal function) and 0 being no function. The score for the individual questions are then summed together to generate a number, the ALSFRS score. The maximum possible score is 40.

ALSFRS-R is a modified version of the ALSFRS. Whereas in the ALSFRS there are 10 assessments, in the ALSFRS-R one of the assessments, #10 (respiratory function) was further divided into three questions to better reflect the importance (weighting) of respiratory changes within the scale. Therefore ALSFRS-R, contains 12 questions (9 of these identical to the traditional ALSFRS) and a maximal score of 48. Please note that some of the patients in the dataset will have ALSFRS scores and some will have ALSFRS-R. ALSFRS and ALSFRS-R information is available in the ALSFRS file. Thus, ALSFRS and ALSFRS-R are composite scores derived from measures of the following symptomatic categories:

1. speech
2. salivation
3. swallowing
4. handwriting
5. cutting food and handling utensils (with or without gastrostomy)
6. dressing and hygiene
7. turning in bed and adjusting bed clothes
8. walking
9. climbing stairs
10. breathing (in the Revised version this is separated in to R1.Dyspnea, R2.Orthopnea and R3.Respiratory insufficiency)

To assure similarity in predictions across patients across these scores, participants are asked to predict ALSFRS slope based on 10 questions (either 1-10 in ALSFRS or 1-9 +R1 (dyspnea) in ALSFRS-R) with a maximal score of 40.

For patients with ALSFRS scores, their ALSFRS Total sum [**ALSFRS Total**] should be used. For patients with ALSFRS-R scores, the total is generated using the sum of the following parameters: [**ALSFRS-R Speech, Salivation, Swallowing, Handwriting, Cutting** (with and without Gastrostomy), **Dressing and Hygiene, Turning in Bed, Walking, Climbing Stairs, Dyspnea**] (the results of questions R2 and R3 are discarded when calculating the sum). In both cases, the number should range between 0-40.

The following describes the slope calculation systematically:

1.1. General

- Add 5a. *Cutting without Gastrostomy* and 5b. *Cutting with Gastrostomy*
- Remove all ALSFRS values for the time points in which NOT all 10 ALSFRS questions are available

  - Convert days to months: m= (days/365) *12)

1.2. Definition:

o First Visit: Assign first visit > 3 months (≥ 92 days) from the first time ALSFRS was fully measured (Reference Visit) as "First Visit"

♣ Note: for the calculation, set the first visit with 10 ALSFRS questions as the Reference Visit for slope calculation and hence calculate all differences relative to this visit. Note that the Reference Visit is not necessarily at delta=0.

♣ Remove record if there are less than 2 visits during the first three months.

o Last Visit:

♣ If there are multiple visits > 12th month, assign the earliest visit > 12th month (from the Reference Visit for slope calculation) as ‘Last Visit’.

♣ Remove record if no last visit matching these criteria exists.

Algorithms will be evaluated based on the accuracy of slope prediction using the Concordance index, Pearson Correlation and Root mean Square of Deviation (RMSD).

Predicting Survival

Survival in ALS is time until either death or tracheostomy (the introduction of invasive breathing tube- time where without intervention the patient was unlikely to survive).
In the PRO-ACT data, different patients were monitored for different durations of time. If they have died, time of death is recorded in days from trial onset. If they were alive, the time of last assessment is recorded, assuming its greater than 90 days. Survival rates are ~15-25% across trials.

In the Sub Challenge 2 and 4, participants will predict three measurements- probability of survival for 0-12 months, probability of Survival for 0-18 months and probability of survival for 0-24.

Algorithms will be evaluated using the Concordance Index. Please note that this assessment is based on the ranking of predictions according to their value, not based on the value itself. Predicting the same exact value multiple times leads to ties in the ranking and might thus reduce final scores.

Please indicate clearly in your Method Write Up if you were predicting the probability of dying or the probability of staying alive.

Building the Models

Goal of this challenge is the prediction of the disease progression of ALS patients in terms of the clinical targets ALSFRS slope as well as survival. As two different datasets are provided (trials and registries), this setup results in four separate sub-challenges. For a given sub-challenge, a participant will build a dedicated predictive model. We impose the constraint that only a limited number of 6 features are allowed for the prediction of a given patient, that is, a different subset of features may be chosen specifically for each patient, or perhaps specifically for different groups of patients. Alternatively, a participant may decide to use the same 6 features across all patients. Note that the sub challenges are separate, so that a different subset of features may be chosen for a given patient depending on whether ALSFRS slope or survival is predicted. A feature is characterised by the feature_name column in the data format. That is, if a given feature is specified you will get all the matching data, for instance in the case of longitudinal data you will get all time points.

The predictive model for a given sub challenge consists of two executables that can read and write text files in the tabular format defined above. The two programs perform the following tasks:

● The first program (feature selection program or “selector” in short) reads an input file of three months of data for a given individual patient (patient k). In order to perform its function, the selector will be provided with all clinical features that are available for this patient. The selector assigns the patient to a cluster of patients (for instance, from the set of clusters derived based on the training data) specific to the outcome clinical target t. In addition, the selector chooses a small subset of six features to be used by the second program. The selector then writes a tabular text file containing the cluster ID as a single value in the first line (or '0' if no clustering is involved) and, subsequently, the selected features in the format specified above.

● The second program (clinical target prediction program or “predictor” in short), reads the text file produced by the first program and uses the cluster ID as well as the six features and their corresponding values to predict the clinical target t for patient k (see overview Figure)

Figure 2.1- overview of the challenge

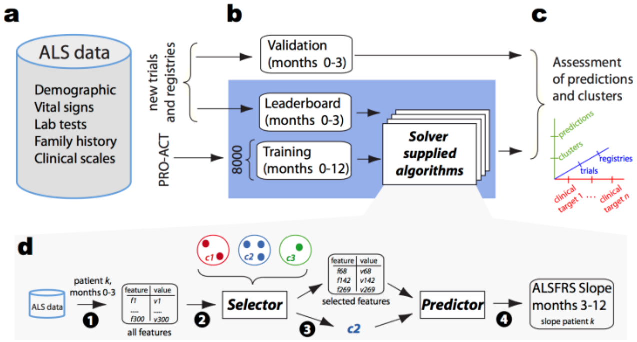

Thus, the solvers algorithms will be provided with the data for a given patient at a time and will then provide a single prediction. Please note that for the leaderboard and final evaluation, we will enforce that the output of the selector may only comprise original features and their original feature values. For instance, if your prediction approach relies on the imputation of missing feature values, we ask you to implement the imputation in the predictor script (you may use the cluster number in order to specify the base set of patients to be used for the imputation).

Predictions of the different clinical targets will be divided into separate sub-challenges. Thus, the selector and predictor submitted to a given sub-challenge will be called for each patient in the validation set. All in all, scoring is performed across all patients in the validation set.

Generally, submitted algorithms will only see patients’ longitudinal data from a limited time period (the first three months after a patient entered a trial), while the remaining data (months three to twelve) will be kept hidden from the participants and will only be used for the assessment of the predictions. An additional, separate training set of patients with full data of the first twelve months or more is provided to enable the solvers to develop and train their algorithms.

The goal of this challenge is to detect patient specific subgroups and to characterize them via defined subsets of features that are especially predictive. As there is currently no gold standard available for ALS patient subgroups, the scoring of participants submissions focuses on their ability to predict clinical outcomes using only a small subset of features. The restriction to a small number of features can lead to the emergence of patients and to more interpretable predictions. In order to assess the submissions in a fair way, challenge setup and scoring has to determine precisely to what extend the performance of the predictions is based on the use of the limited subset of features allowed for each patient or subgroup of patients.

However, there are still possibilities of exploiting information from the remaining features in ways that would lead to data leakage from the selector (who sees all the features) to the predictor. One way of leakage could conceivably exploit the cluster number that is passed to the predictor. A second possibility would be that the selected features encode a partial outcome prediction. Our preliminary tests revealed that such data leakage cannot be prevented by the challenge setup alone. We would therefore like to ask participants to avoid such data leakage. Importantly, an intentional data leakage stands against the terms and conditions signed when joining the challenge. The organizers reserve the right to correct the scores of the final submission if we detect additional information passed from the selector to the predictor.

Supplementary material part 3- Summary of the ALS Stratification Challenge Participants Survey

I. EXECUTIVE SUMMARY: ALS Stratification Prize Survey

1) Description of the prize

The DREAM ALS Stratification Prize4Life Challenge is a collaboration between Prize4Life, Sage Bionetworks and DREAM, with sponsorship from Biogen Idec, Eli Lilly and IBM, which run from June 22 to Sept 30 2015. The challenge sought to accelerate the development of algorithms that would identify meaningful sub-groups of ALS patients and through that predict, using 3 month of information, ALS progression and survival. Such predictive algorithms could then be used identify for the first time distinct groups of patients, could aid clinicians in serving patients in the clinic, and serve to reduce the costs and improve the accuracy of ALS clinical trials.

Overall, 288 participants registered to the challenge and 30 teams made 80 final submissions to the challenge four sub-challenges. Three teams were declared best performing.

We conducted a survey of registered prize participants after the close of the prize in order to learn more about our prize participants, to assess the impact of the prize, and to gauge active interest in this or future Prize4Life or DREAM and Sage challenges. The survey was launched on November 11 2015 (5 weeks after the end of the challenge) and was open for one month. Overall, 28 participants completed the survey, including 24 participants that have made a final submissions, representing over 60% of the final submitting teams.

Important points

- **Participants were diverse in age and gender and geographical location. Most were academic, from undergraduate students to professors, and their primary research focus was quantitative. Most did not have past experience in challenges and preferred to work in small teams of 1-2 participants.**
- **Participants had limited prior knowledge about ALS, with 85% knowing little about ALS. Overall, the participants saw great future utility and agreed or strongly agreed that the challenge will impactful with regard ALS awareness, increased research interest and better predictive algorithms.**
- **The majority of the participants responding to the challenge came through word of mouth, with another large group coming through DREAM's network of challenge participants.**
- **The majority of the participants (81%) found the challenge "Hard but challenging in a good way" and satisfaction was high. Perceived barriers included challenge design, data quality and submission through the IBM cloud, although all received high satisfaction rates too.**
- **Most participants (75%) would be interested in future work on ALS, as part of a challenge or not. Cash prizes are not a major driver of that decision. Primary motivations are "publicizing your method on a top-tier journal and having impact on patient lives".**

II. Survey Analysis

General: 28 Challenge participants participated in the survey. Of 288 challenge registered this represents <10% of the registrants. However, 24/28 of the survey participants made a final submission to the challenge, with representatives of 19 of the 30 (63%) final teams. Therefore, the survey results are most valuable in interpreting the views of challenge finalists, as oppose to challenge registrants who did not make a final submission.

1) Team Demographics

We gathered basic demographic data to learn more about our prize participants and to better understand the types of teams that the treatment prize successfully attracted to compete.

Overall, 28 participants from 13 countries participated in the survey (to compare- 30 teams from 15 countries had submitted a final solution)

Figure 3.1- Geographical Distribution

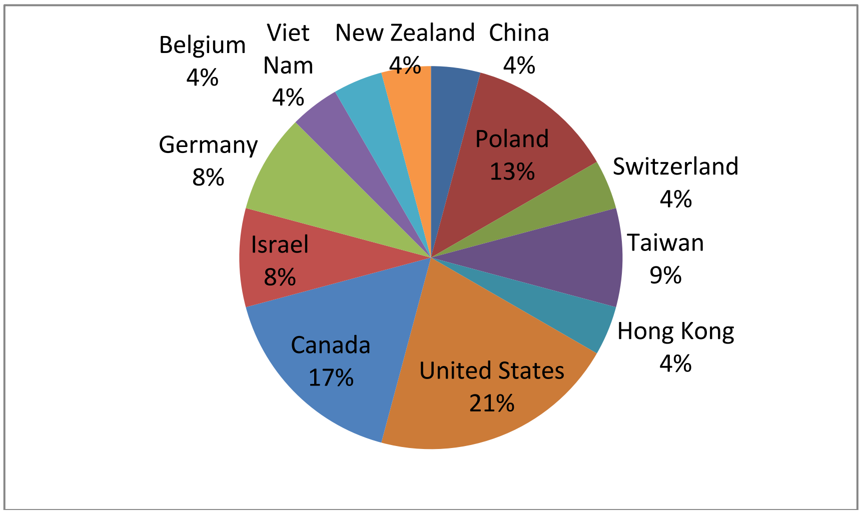

46% (13/28) of the participants were between the ages of 20 and 30, 39% (11/28) were 30 to 40 and 11% (3/28) were 40 to 50 years old. 32% (9/28) were women and 68% (19/28) were men.

The challenge drew a diverse crowd from different geographical, gender and age distribution.

2) Participants Background

91% (22/24) of the participants worked for academic institutions, 4% (1/24) worked for industry and 4% (1/24) for a private research institute.

Of the 91% working for academic institution, graduate students were the most common (26%), followed by staff scientists (22%), assistant professors (19%), undergraduate students (11%), post doctoral fellows (7%) or post graduate certification students (4%).

Figure 3.2- Career Stage

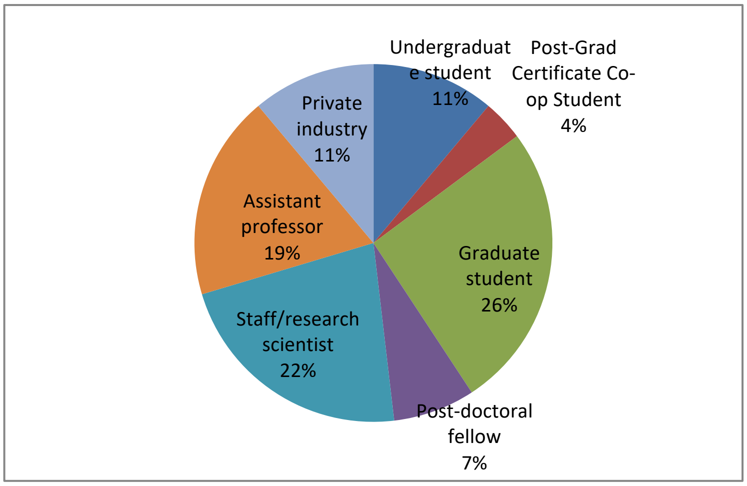

The majority of participants came from quantitative field of research, with primary research fields being machine learning (28%) computational biology (22%), computer science (21%), statistics (12%) or Mathematics (2%). Only 7% had primary research in the fields of medicine and 3% in biology, and none of the participants that responded to the survey had a primary research focus in ALS.
In “Others”, three participants listed primary research as being in Artificial Intelligence, chemistry and genomics.

Figure 3.3- Primary Research Field

Figure 3.4- Team Size

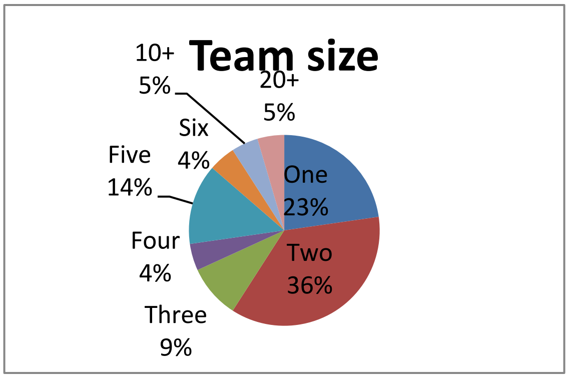

The challenge was popular mostly among academics, in diverse stages of their career and with a quantitative research focus (None with ALS research focus prior to the challenge). Participants preferred to work on small teams of 1-2 collaborators.

3) Knowledge about ALS

Figure 3.5- Prior knowledge about ALS

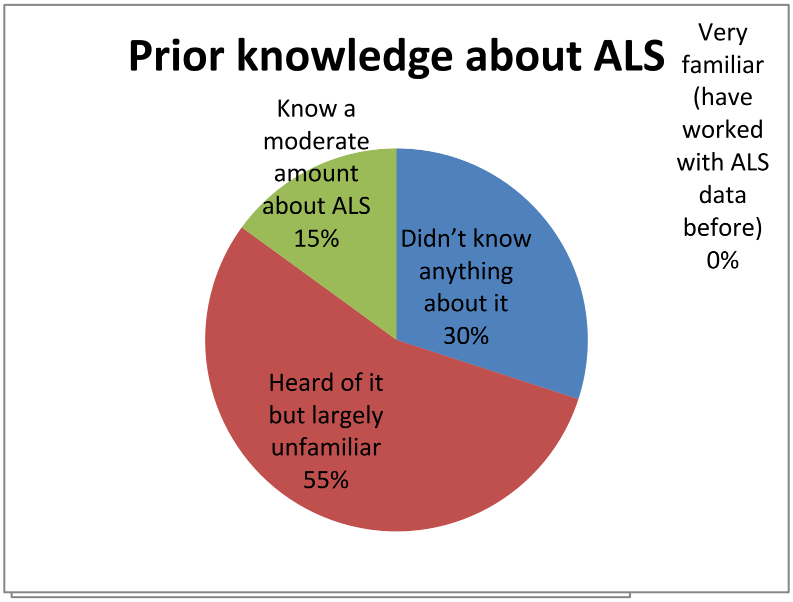

Participants started the challenge knowing only little about ALS- 30% declared that they knew nothing about it, 55% that they "Heard of it but were largely unfamiliar with it", only 10% knew a moderate amount and none had experience working in the field.

The participants made efforts to complement their lack of ALS expertise. Asked which sources of information (more than one answered allowed) about ALS they used for the challenge, most answered that they have used the challenge's supplementary information (45%; 9/19) and information available online (26%; 5/19). Beyond that, 21% (4/19) also read scientific papers, and 5% (1/19) consulted with Medical experts.

Table 3.1- Sources of information on ALS:

Experts in the field of medicine	1
Information I found online	5
Scientific papers	4
Supplementary information provided in the Challenge	9

Most participants (85%) had only limited previous knowledge about ALS. They used supplementary information, online information and scientific publications to supplement their ALS knowledge.

4) Views on the Challenge's Utility

We asked the participants regarding their views on the challenge utility. Specifically, they were asked "Please indicate how strongly you agree with the ALS Prediction Prize's ability to do the following":

Figure 3.6- Participants Views Regarding Challenge Impact

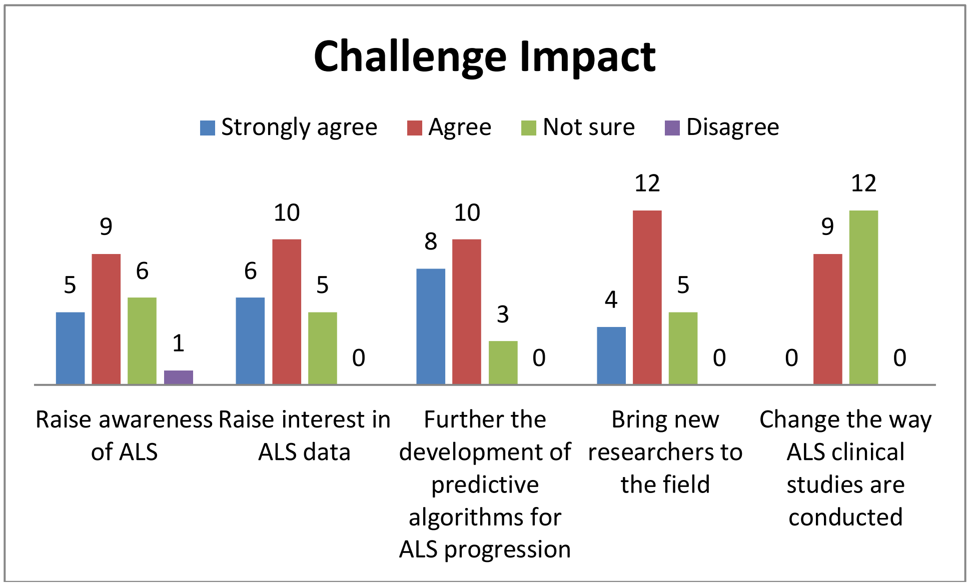

Table 3.2- Participants Views Regarding Challenge Impact

	Raise Awareness of ALS	Raise Interest in ALS Data	Further the Development of Predictive Algorithms for ALS Progression	Bring New Researchers to the Field	Change the Way ALS Clinical Studies are Conducted
Strongly Agree	5	6	8	4	0
Agree	9	10	10	12	9
Not Sure	6	5	3	5	12
Disagree	1	0	0	0	0

- 67% of the participants agreed or strongly agreed with the challenge's ability to raise awareness of ALS and only 5% disagreed.
- 76% of the participants agreed or strongly agreed with the challenge's ability to raise interest in ALS data and none disagreed.
- 85% of the participants agreed or strongly agreed with the challenge's ability to further the development of predictive algorithms for ALS progression, and none disagreed.
- 76% of the participants agreed or strongly agreed with the challenge's ability to bring new researchers to the field, and none disagreed.

- 44% of the participants agreed or strongly agreed with the challenge's ability to change the way ALS clinical studies are conducted, and none disagreed.

Overall, the participants saw great future utility and almost all agreed or strongly agreed that the challenge will impactful with regard ALS awareness, increased research interest and better predictive algorithms.

5) Learning about the Challenge

Participants learned about the challenge from diverse and sometimes multiple sources:

- Most participants (54%, 15/28) first learn about the challenge by word of mouth (10/28) or through their university instructor.
- DREAM's emails and website brought 50% (14/28) of the participants to the challenge
- Prize4Life website and social media brought 14% (4/28) of the participants to the challenge
- General press brought in 11% (3/28) of the participants
- 4% (1/28) came through other sources (participant commented "wikipedia")

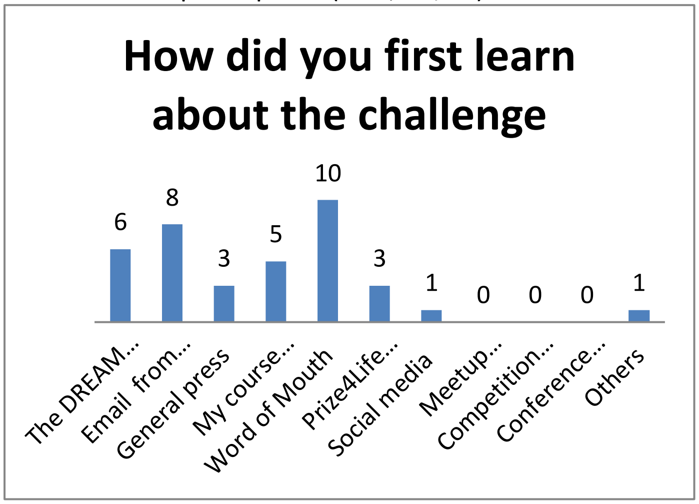

Figure 3.7- Challenge Marketing

Looking at previous challenges the participants competed in, 43% (12/28) had participated in a past DREAM challenge, and 57% (16/28) did not.

The majority of the participants responding to the challenge came through word of mouth, with another large group coming through DREAM and with past experience in DREAM challenges. Other sources were Prize4Life, as well as general press.

6) Experience with the Challenge

Participants spent significant amount of time on the challenge, even those that didn't submit:

- One team (4%; 1/22) has spent only 3-5 hours
- 36% (8/22) spent 20 hours
- 27% (6/22) have spent 100 hours
- 27% (6/22) have spent 100-200 hours
- One team (4%; 1/22) spent over 300 hours

Generally, the participants found the challenge hard but engaging. 81% (2/21) deemed it "Hard but challenging in a good way", 9% (2/21) as being of medium difficulty and 9% (2/21) at being too hard.

Figure 3.8- challenge difficulty

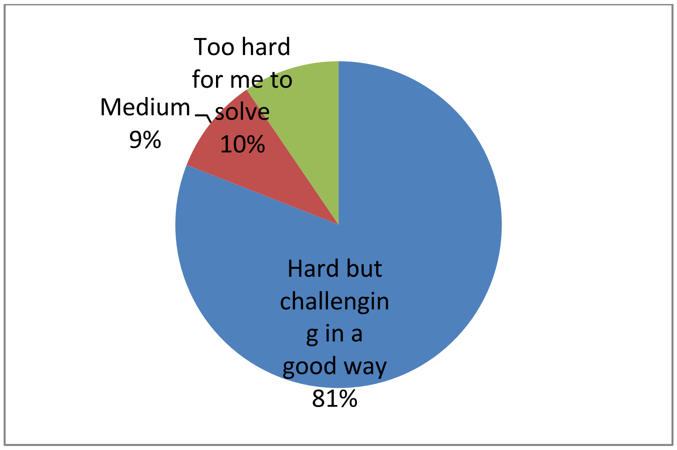

Asked regarding any barriers when competing for the challenge (for participants that did make a final submission), the most common barriers identified includes problems with the challenge design (21%), problems with the data (21%), and problems with submitting to the IBM cloud (21%). Other barriers were limited algorithmic experience (13%), short time frame (8%), lack of clarity about challenge design (8%) and competing priorities (8%).

Figure 3.9- Challenge Barriers

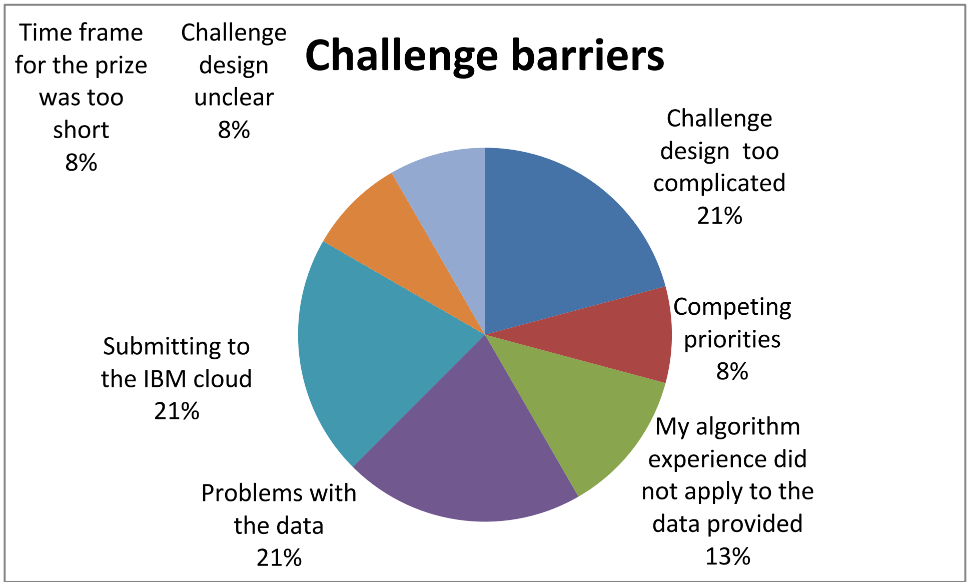

Specifically for the four responders that didn't make a final submission, their reasoning was lack of time (1/3), limit data description (1/3) and not enough insight from results (1/3).

- Generally most participants (65% 15/23) agreed with the statement “the challenge was well organized” while 35% (8/23) did not.
    - Reasons for disagreement included problems with challenge communication (3/8), with the challenge data (3/8) or with the challenge website.
- Generally most participants (66%, 14/21) agreed with the statement “The duration of the Challenge gave me enough time to analyze the data” while 33% (7/21) did not.
    - Reasons for disagreement included personal time limitations (3/6), problems with the data (2/6) and a need for 1-2 more months (1/6)

When participants were asked to openly suggest changes for a future prize 2/6 mentioned better data quality, 2/6 mentioned better ways to assess the data (without submitting to the cloud) and 2/6 suggested different scoring metric.

The participants put in effort even when not submitting. Most responders chose to make submission and general satisfaction was high, but still they encountered barriers such as data problems, challenge design and difficulties in making a final submission.

7) Participants’ Motivation

Many participants (75%) would be interested in further work on ALS data:

- 39% (11/28) answered “I am interested to participate in future DREAM Challenges that use data sets similar to the one(s) used in the Challenge(s) that I joined”
- 36% (10/28) answered “I am interested to use this data for my own research efforts”
- 25% (7/28) answered “I have no further interest to access this data”

As for their motivation to continue to work:

- The most common reason, shared by 39% (7/18) of the responders, was “Evidence that it would be clinically important”(the most common reason)
- 33% (6/18) if there were more data,
- 11% (2/18) if there were more academic interest in ALS research,
- 11% (2/18) would do so anyhow
- 5% (1/18) would not work on the data further

Figure 3.10- Participants' Motivations to Continuing to Work on ALS Data

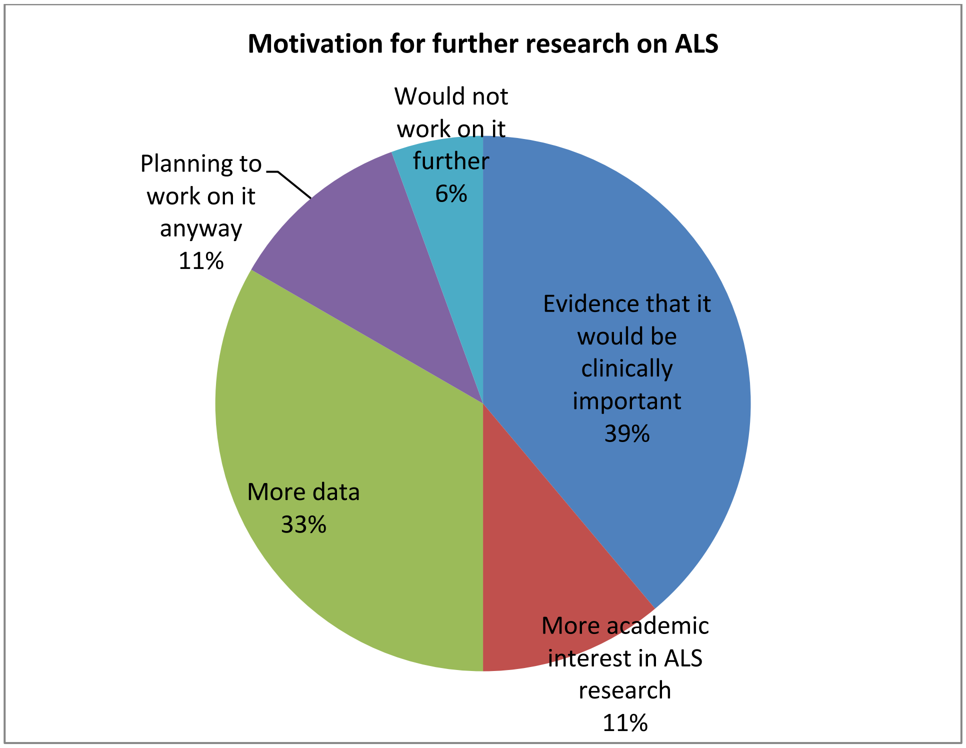

We asked participants to rank different motivations they may have to participate in a challenge, with 1 being the top rank and 6 being the bottom rank.
The top motivation ranked most often was "Opportunity to publish a paper on your model in a top tier journal". It was ranked 1 by 59% (10/17) of the participants. The second was "Opportunity to do something that would be meaningful for ALS patients". It was ranked 1 by 35% (6/17) of the participants. No other motivation was rank first (one responder chose to not give any answer top ranking)

Figure 3.11- Occurrence of Different Motivations Being Ranked as Top Motivation for Further Work

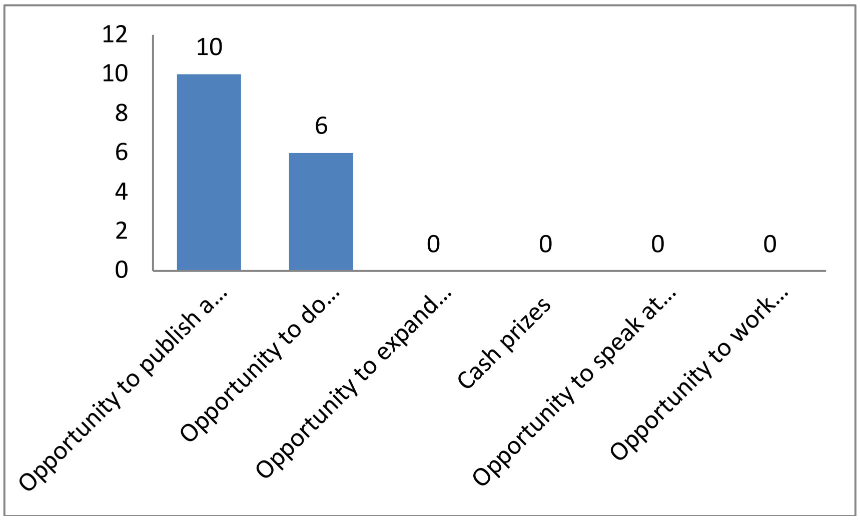

Table 3.3- Ranking of challenge motivations

Motivation	Average ranking
Opportunity to publish a paper on your model in a top tier journal	1.384615385
Opportunity to do something that would be meaningful for ALS patients	2.461538462
Opportunity to expand my network and connect with other experts in the field	3.5
Cash prizes	4.0833333
Opportunity to speak at the DREAM conference about your top-scoring model	4
Opportunity to work with unpublished data	4.18

We also asked what size of a prize was needed to attract the participants who answered the survey to this challenge:

- 50% (10/20) participants indicated that they would participate in a challenge for recognition and journal publication alone.
- 15% (3/20) participants indicated that they would participate in a challenge for $5,000.
- 15% (3/20) participants indicated that they would participate in a challenge for $10,000.
- 25% (5/20) participants indicated that they would participate in a challenge for $20,000.

Most participants would be interested in future work on ALS, as part of a challenge or not. Cash prizes are not a major driver of that decision and most would consider it regardless. Primary motivations are "publicizing your method on a top-tier journal and having impact on patient’s lives".

Supplementary material part 4- Baseline algorithms

For the challenge two baseline algorithms were used. One, "baseline algorithm 1" was designed to be a standard "of-the shelf" algorithm and its development is outlined below. The second "baseline algorithm 2" was an adaptation of the winning algorithm from the ALS Prediction Prize (Kueffner et al. 2015- algorithm is described and available as supplementary material of that publication – please see below for adaptations of this algorithm required for the current Stratification Prize challenge). Unsurprisingly baseline algorithm 2, which was developed specifically for similar data, often outperformed the "off the shelf" algorithm.

Baseline algorithm 1- Development

Figure 4.1 – Preparation of baseline algorithm 1

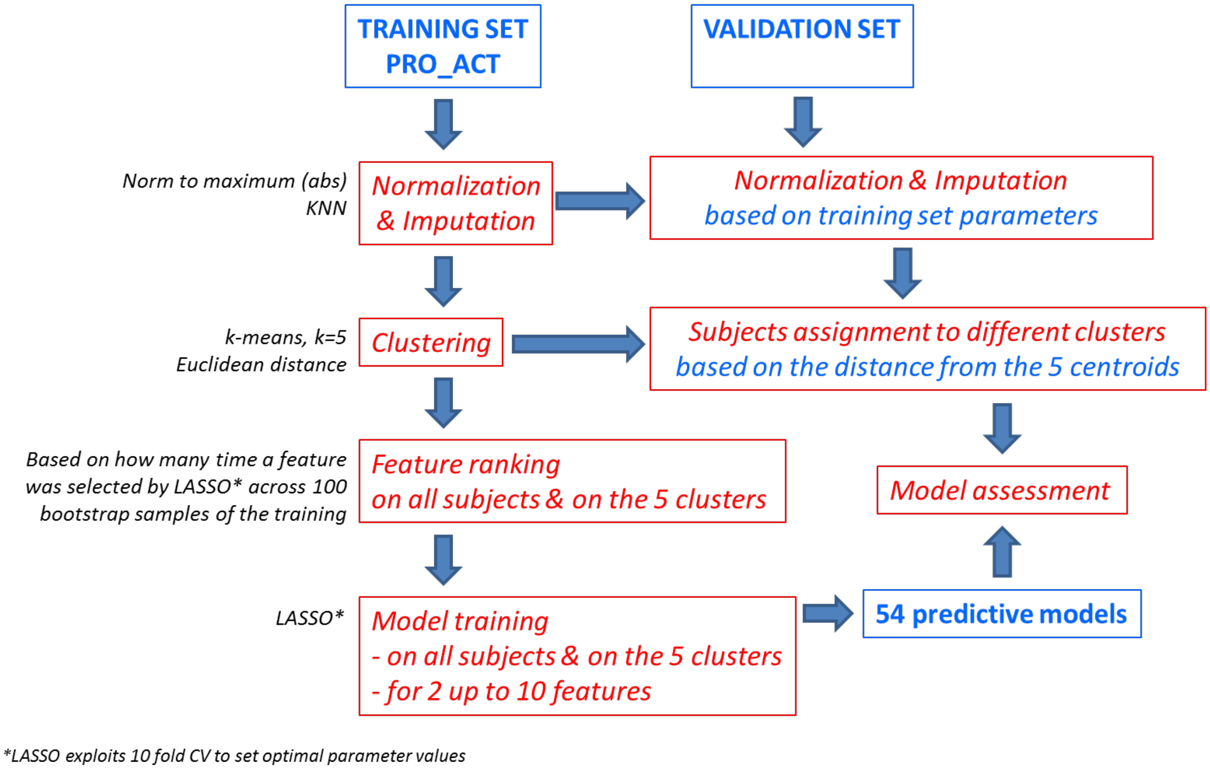

As can be seen the "off-the-shelf" algorithm still required substantial work on data standardization and clustering decision making. In this case five clusters were chosen as they led to diverse differences in performance (Figure 4.2), using both RMSE and concordance index (CI) as measures.

Imputation

Variable were normalized to their maximum value and data was imputed using k-nearest-neighbors (k=10).

Decriptive statistics (for Longitudinal)

As regard ALSFRS and FVC feature, for which we have different acquisitions at different time points, we defined aggregated variables such as slope, mean and coefficient of variations (considering data from the first 3 months).

Pre-processing
Features like Gender(M/F), treatment_group(Active/Placebo), family_ALS_hist(No/Yes), etc... were converted into 0/1; Dummy features were created for features like Race or Trial, so for example, each dummy_feature "American Indian" "Asian" "Hispanic" etc. assumes values 0/1 (corresponding to TRUE/FALSE). Variables were removed with more than 60% NAs, then subjects with >80%NAs, finally variables with >40% NAs. This led to a total of 145 features.

Feature selection and model prediction

To perform variable selection, the R package glmnet (generalized linear model via penalized maximum likelihood) was used. The regularization path is computed for the lasso penalty at a grid of values for the regularization parameter lambda. Lambda is chosen using cross-validation equal to lambda.min, which is the value of lambda that gives minimum mean cross-validated error. Goals were to (i) To rank and select robust variables; and (ii) To assess possible prediction performance on external, previously unseen, test sets. Therefore, glmnet was run on B different bootstrap datasets (B=100 runs), each time sampling the subjects for the training-set and using the remaining for the test-set. At each run, the number of selected variables was obtained and an assessment of the performance on the external test set. Variable ranking and selection was performed on 3 clusters of subjects, for each of the two outcomes, i.e. prediction of ALSFRS_slope, survival (0/1) at time t (in this case the probability of death at time t is predicted). In particular, the average performance using concordance index, on the 100 bootstrap test-sets was assessed, for different numbers of selected features, ranging from 2 to 10. The optimal number of features (with a constraint of maximum 6 features) for each cluster was chosen (here, always 6 features were selected) and the model was fit on the entire training set to get the final parameter value.

Clustering
Normalized data was clustered using hierarchical clustering and defining different numbers of clusters. For prediction, the three top clusters were used

Baseline algorithm 1- performance and feature number

Table 4.1- Top features used for prediction by Baseline algorithm 1 for predicting PRO-ACT progression

RANK	all-subjects	cluster C1	cluster C2	cluster C3	cluster C4	cluster C5
**1**	onset_delta	onset_delta	onset_delta	onset_delta	onset_delta	onset_delta
**2**	**InitialWeightSlope**	**Age**	InitialSlopeFVC_percent	**InitialWeightSlope**	InitialMeanFVC	InitialMeanFVC_percent
**3**	InitialCVALSFRS_Total	InitialSlopeQ8	InitialCVQ5	InitialMeanFVC_percent	**InitialPulseMean**	InitialCVQ9
**4**	**InitialMeanFVC_percent**	InitialQ9	InitialMeanFVC_percent	InitialSlopeFVC	InitialWeightSlope	InitialMeanFVC
**5**	**InitialPulseMean**	treatment_group	InitialCVQ1	InitialPulseSlope	InitialCvFVC	InitialCVALSFRS_Total
**6**	**InitialSlopeQ5**	InitialALSFRS_Total	InitialQ6	InitialCVQ5	InitialQ3	InitialQ7
**7**	Age	**if_use_Riluzole**	**InitialWeightSlope**	InitialCVQ8	InitialSlopeFVC_percent	InitialWeightSlope
**8**	InitialQ3	**InitialPulseCv**	InitialCVQ2	InitialCvFVC	InitialSlopeALSFRS_Total	InitialALSFRS_Total
**9**	InitialSlopeQ6	InitialCVQ4	InitialQ7	**Hispanic**	InitialCVALSFRS_Total	InitialPulseMean
**10**	InitialQ7	InitialSlopeFVC_percent	InitialSlopeQ6	Bulbar	InitialMeanFVC_percent	InitialQ5

Figure 4.2- Performance of baseline algorithm 1 for the PRO-ACT progression sub-challenge

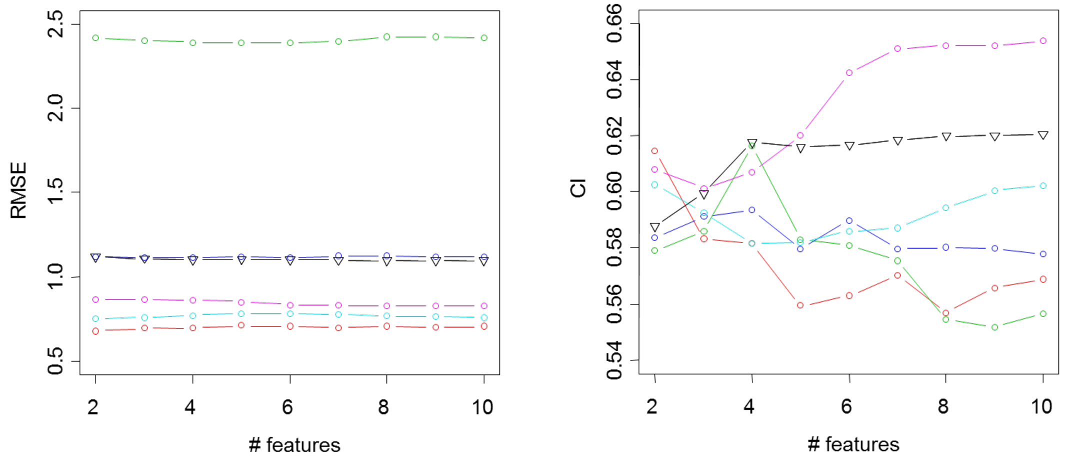

black: all-subjects; red: cluster C1; green: cluster C2; blue: cluster C3; cyan:cluster C4; Pink: cluster C5

As can be seen in the analysis of baseline algorithm 1 performance (in both Figure 4.2 and table 4.1) the difference between clusters varies greatly depending and that variance increases as then number of features used for prediction increases and then plateaus around 6 features. As the main goal of the ALS stratification

challenge was not to improve prediction overall (that was already explored in the previous ALS prediction challenge) but rather to explore emerging distinct patient clustering, we wanted to choose a number of features that would differentiate the clusters while still be small enough to encourage participants to use clusters. **Based on the analysis above, we settled on a threshold of six features only.**

For predicting survival, baseline algorithm 1 used a Cox model, fitted by a LASSO model, looking at prediction for 12,18 and 24 months, and cluster number settled as 4 to allow sufficient training data.

Figure 4.3 -Performance of baseline algorithm 1 for the PRO-ACT survival sub-challenge

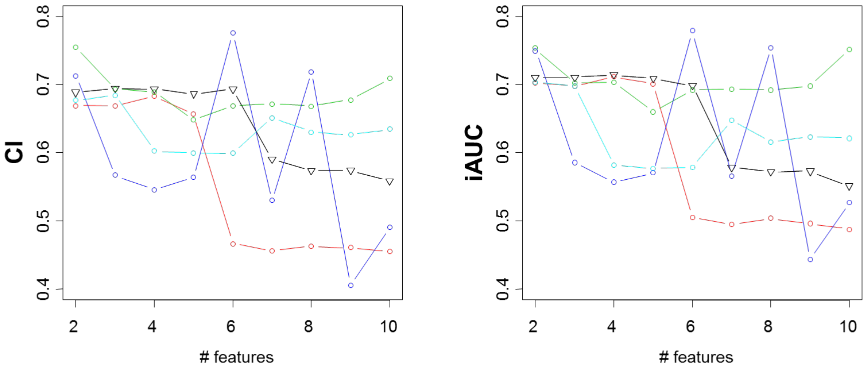

Black: all-subjects;red: cluster C1; green: cluster C2; blue: cluster C3; cyan:cluster C4.

The strong similarity between CI and iAUC is expected since CI is a generalization of the area under the ROC curve to regression problems

Table 4.2- Top features used for prediction by Baseline algorithm 1 for predicting PRO-ACT survival

RANK	all-subjects	cluster C1	cluster C2	cluster C3	cluster C4
**1**	onset_delta	onset_delta	onset_delta	InitialSlopeFVC_percent	InitialSlopeALSFRS_Total
**2**	InitialSlopeFVC_percent	**InitialWeightSlope**	InitialWeightSlope	InitialMeanFVC	onset_delta
**3**	**InitialWeightSlope**	InitialMeanFVC_percent	InitialCvFVC	InitialCvFVC	InitialWeightSlope
**4**	InitialSlopeALSFRS_Total	InitialWeightMean	Age	Age	Age
**5**	Age	InitialSlopeALSFRS_Total	InitialSlopeFVC_percent	InitialQ9	InitialWeightMean
**6**	InitialWeightMean	Age	treament_group_delta	onset_delta	InitialALSFRS_Total
**7**	**InitialPulseMean**	treament_group_delta	InitialPulseMean	InitialSlopeALSFRS_Total	InitialMeanFVC_percent
**8**	InitialMeanFVC_percent	InitialQ3	InitialMeanFVC_percent	InitialCVALSFRS_Total	InitialPulseMean
**9**	InitialMeanFVC	Gender	InitialWeightMean	InitialWeightMean	InitialWeightCv
**10**	InitialDiastMean	if_use_Riluzole	InitialSlopeQ8	InitialQ8	treatment_group

Baseline algorithm 2

Descriptive statistics (for Longitudinal)

For each of the question scores, body_part score and total ALSFRS score: mean, standard deviation, change of scores first to last visit, slope

Feature selection

Use random forest to select top 6 important variables: procedure is done for each cluster

Model prediction

We use Breiman and Cutler's random forests (http://cran.r- project.org/web/packages/randomForest/randomForest.pdf) for classification and regression R package in this algorithm. For details on the random forest prediction, see Kueffner et al. (2015) Crowdsourced analysis of clinical trial data to predict amyotrophic lateral sclerosis progression, supplementary Text and Figures, p.19.

Clustering

For a given number of clusters K (k=2,3,4), apply Kmeans on the 10 top selected variables to split the patients into K clusters (i) For each cluster, run random forest on all variables and select top six features. (ii) Re-run random forest using the variables derived from the six features, and make prediction on patients held out from the training set. Calculate the metrics to measure the goodness of fit, and the metrics are rmse, correlation and concordance index between prediction and true values. Select the number of clusters K, which produces the best fit.

Supplementary material part 5- Full results, algorithm description and aggregated performance

Table 5.1- Algorithms submitted to the four sub-challenges

Sub-challenge	Database	Prediction	Number of patients (validation)*	Number of submitted algorithms	Number of clustering algorithms	Mode number of clusters per algorithm** (range)
1	PRO-ACT	Progression	638	26	12	4 (2-14)
2	PRO-ACT	Survival	1260	15	4	4 (2-8)
3	Registries	Progression	223	16	4	3 (3-131)
4	Registries	Survival	485	13	2	*NA* (6, 159)

* Number of patients from the validation set that were used for the assessment, scoring and performance ranking of submitted algorithms. Patients could only be used for validation if they had at least two complete ALSFRS scores taken between delta time 0 to 12 months or survival records for sub-challenges 1 & 3 or 2 & 4, respectively (compare to number of validation set patients in Table 1).

** Only for algorithms that used clusters

Submitted algorithms performance

In general, the top performing teams in each sub-challenge (except for the registry progression sub-challenge) significantly outperformed the best baseline algorithm. In order to find the best algorithm among those that performed better than baseline we bootstrapped the algorithms' predictions and compared the performance of the bootstrapping samples. The advantage of the best performer was particularly striking in both survival prediction challenges, where the winning algorithm outperformed the respective second best in more than 98% of the bootstrap samples (see supplementary).

Four algorithms by three teams (Guanlab, UglyDuckling and Jinfeng_Xiao) were declared the winners and shared a $28,000 prize, awarded at the Nov. 2015 DREAM conference (www.iscb.org/recomb-regsysgen2015).

It is noteworthy to add that for the sub-challenges running on the PRO-ACT database, the best performing algorithms significantly outperformed the winning method from the first challenge (Kueffner et al. 2015). This is even more noteworthy given that the validation set was not randomly divided, but actually included the more difficult and realistic criteria of application of the algorithms to a data comprising of six new never used before trials.

Table 5.2- Full results- PRO-ACT survival sub-challenge

Name	ID	Challenge	#cluster	#feature-sets	#patients	score1	score2	score3	z1	z2	z3	rank	
baseline 1						0.644:0.016	0.645:0.016	0.642:0.016	22.945:2.507				
baseline 2						0.721:0.015	0.730:0.014	0.735:0.014	36.351:2.252				0.984
Yuanfang Gua	3319559	proact_survival	1	1	1260	0.77315413	0.77315413	0.77315413	14.4291645	14.4817961	14.4817961	1	43.3927568
Rama Ragha	3327490	proact_survival	4	4	1260	0.75320851	0.7523428	0.75145264	13.3793955	13.3864633	13.3396124	2	40.1054712
Wojciech Les	3324341	proact_survival	2	2	1260	0.72810287	0.75871581	0.75257268	12.0580457	13.7218848	13.3985621	3	
Xiaotao Liang	3327729	proact_survival	1	1	1260	0.73959678	0.73959678	0.73959678	12.6629886	12.7156202	12.7156202	4	
Christoph Kur	3325789	proact_survival	1	1	1260	0.71223148	0.73821751	0.73985112	11.2227096	12.643027	12.7290062	5	
Jaume Bacar	3323880	proact_survival	8	3	1260	0.69044684	0.6940124	0.71987616	10.0761497	10.3164423	11.6776926	6	
DREAMer	3321630	proact_survival	1	1	1260	0.69214403	0.69214403	0.69214403	10.1654753	10.2181069	10.2181069	7	
ceve	3324175	proact_survival	4	4	1260	0.6873068	0.698429	0.6893268	9.91088444	10.5488948	10.0698315	8	
Jouhyun Jeon	3325206	proact_survival	1	7	1260	0.70463083	0.70830399	0.65791173	10.822675	11.0686309	8.41640667	9	
Samad Jahar	3323761	proact_survival	1	1	1260	0.68764918	0.6894882	0.68494933	9.92890402	10.0783265	9.83943837	10	
Omar (OmarY	3323587	proact_survival	1	1	1260	0.68523301	0.68523301	0.68523301	9.80173729	9.85436887	9.85436887	11	
Anatoly Cherr	1967302	proact_survival	1	1	1260	0.68511073	0.67027135	0.65723187	9.79530173	9.06691338	8.38062494	12	
dave.wadden	3328877	proact_survival	1	8	1260	0.67213973	0.66358043	0.66011269	9.1126172	8.71475937	8.5322468	13	
Dinithi Sumar	3329879	proact_survival	1	1	1260	0.61698361	0.58728528	0.60202684	6.20966344	4.69922547	5.47509695	14	
**Xihui Lin (lin**	**3321842**	**proact_survival**	**1**	**1**	**1260**	**0.56407247**	**0.56281058**	**0.548025**	**3.4248666**	**3.41108317**	**2.63289489**	**15**	
Xiang Yu (ope	3327120	proact_survival	1	1	1260	0.5236579	0.46165434	0.5982314	1.29778431	-1.9129294	5.27533707	16	

Table 5.3- Full results- PRO-ACT progression sub-challenge

Name	ID	Challenge	#cluster	#feature-sets	#patients	score1	score2	score3	z1	z2	z3	rank	
baseline 1						0.532:0.013	0.137:0.036	0.680:0.017	15.134:2.577				
baseline 2						0.603:0.013	0.293:0.035	0.575:0.017	30.694:2.461				0.964
Omar (OmarY	3323587	proact_progressio	1	1	638	0.62687559	0.36676851	0.5492109	10.0673529	9.16921275	-16.634653	1	35.8712186
**Xihui Lin (lin**	**3321842**	**proact_progress**	**1**	**2**	**638**	**0.62097016**	**0.34780947**	**0.5523891**	**9.61308945**	**8.69523675**	**-16.4477**	**2**	**34.7560264**
Xiaotao Liang	3327729	proact_progressio	1	1	638	0.61892295	0.33840612	0.5572919	9.45561146	8.460153	-16.1593	3	34.0750647
Xiang Yu (ope	3327120	proact_progressio	1	1	638	0.61744167	0.34077982	0.55675125	9.34166705	8.5194955	-16.191103	4	
Samad Jahar	3323761	proact_progressio	1	1	638	0.6222792	0.33125994	0.56120694	9.71378451	8.2814985	-15.929004	5	
Jonathan Gor	3327813	proact_progressio	4	26	638	0.61370157	0.32726783	0.55681522	9.05396687	8.18169575	-16.18734	6	
Yuelong Guo	3327909	proact_progressio	2	1	638	0.61361299	0.34227102	0.56461921	9.04715292	8.5567755	-15.728282	7	
Jinfeng Xiao	3325593	proact_progressio	6	5	638	0.61022229	0.32071013	0.55982732	8.78633	8.01775325	-16.010158	8	
DREAMer	3321630	proact_progressio	1	1	638	0.61058646	0.31649802	0.55920672	8.81434291	7.9124505	-16.046664	9	
Yuanfang Gua	3319559	proact_progressio	1	1	638	0.60695462	0.32326455	0.55897893	8.5349709	8.08161375	-16.060063	10	
yoav bar sina	3329481	proact_progressio	8	4	638	0.60849495	0.32088147	0.56270208	8.65345795	8.02203675	-15.841054	11	
ceve	3324175	proact_progressio	4	4	638	0.60960222	0.31648064	0.56505915	8.73863234	7.912016	-15.702403	12	
Thomas Lari	3329158	proact_progressio	1	1	638	0.61154609	0.30627913	0.56909655	8.88816072	7.65697825	-15.464909	13	
Wojciech Les	3324341	proact_progressio	2	2	638	0.60060629	0.31678097	0.56407724	8.04663771	7.91952425	-15.760162	14	
Rama Ragha	3327490	proact_progressio	6	8	638	0.61295355	0.30784225	0.57645974	8.99642684	7.69605625	-15.03178	15	
Jaume Bacar	3323880	proact_progressio	4	1	638	0.61030103	0.2919746	0.56854614	8.79238685	7.299365	-15.497286	16	
Ido Hadanny	3329872	proact_progressio	5	6	638	0.59959745	0.29638276	0.56978745	7.96903438	7.409569	-15.424268	17	
Yeeleng Vang	3321656	proact_progressio	14	11	638	0.60175785	0.30340614	0.57658212	8.13521908	7.5851535	-15.024581	18	
Yu-Jia Shiah	3328928	proact_progressio	1	5	638	0.60412986	0.27350176	0.57048542	8.31768156	6.837544	-15.38321	19	
Jeremy Jacob	3322614	proact_progressio	9	4	638	0.5970778	0.26608525	0.58760516	7.77521531	6.65213125	-14.376167	20	
Christoph Kur	3325789	proact_progressio	1	1	638	0.59004542	0.26263359	0.57758466	7.23426327	6.56583975	-14.965608	21	
Wing Chung \	3328096	proact_progressio	1	1	638	0.58891355	0.19749474	0.58753704	7.14719612	4.9373685	-14.380174	22	
Barbara Huar	3324616	proact_progressio	1	4	638	0.574711	0.22010521	0.59347407	6.05469256	5.50263025	-14.030937	23	
Rached Alkal	3327721	proact_progressio	7	19	638	0.56522295	0.19267418	0.59691663	5.32484265	4.8168545	-13.828434	24	
RLB	3331605	proact_progressio	1	1	638	0.55806263	0.17251774	0.59127457	4.77404823	4.3129435	-14.16032	25	
Anatoly Cherr	1967302	proact_progressio	1	1	638	0.57937629	0.20593945	0.67634784	6.41356067	5.14848625	-9.1560095	26	

Table 5.4- Full results- Registry survival sub-challenge

Name	ID	Challenge	#cluster	#feature-sets	#patients	score1	score2	score3	z1	z2	z3	rank	
baseline 1						0.700:0.015	0.700:0.015	0.700:0.015	35.516:2.598				
baseline 2						0.677:0.014	0.692:0.014	0.701:0.014	33.797:2.376				0.994
Yuanfang Gua	3319559	reg_survival	1	1	485	0.71679361	0.71679361	0.71679361	12.811389	12.8702125	12.811389	1	38.4929906
Christoph Kur	3325789	reg_survival	1	1	485	0.68783349	0.69898375	0.70480836	11.1078523	11.8225734	12.106374	2	35.0367998
Xiaotao Liang	3327729	reg_survival	1	1	485	0.69677406	0.69677406	0.69677406	11.6337684	11.6925919	11.6337684	3	
Xiang Yu (ope	3327120	reg_survival	1	1	485	0.68786404	0.68061382	0.67787463	11.1096493	10.7419897	10.522037	4	
Jouhyun Jeon	3325206	reg_survival	1	2	485	0.67757932	0.67702945	0.6830679	10.5046662	10.5311441	10.8275235	5	
Omar (OmarY	3323587	reg_survival	1	1	485	0.66817034	0.66817034	0.66817034	9.95119643	10.01002	9.95119643	6	
Samad Jahar	3323761	reg_survival	1	1	485	0.65760051	0.65080852	0.67527799	9.32944147	8.98873653	10.3692937	7	
Jaume Bacar	3323880	reg_survival	6	1	485	0.58248137	0.65873081	0.68632642	4.91066855	9.45475325	11.0192013	8	
Rama Ragha	3327490	reg_survival	1	1	485	0.62959757	0.62959757	0.62959757	7.68221014	7.74103367	7.68221014	9	
DREAMer	3321630	reg_survival	1	2	485	0.62712313	0.62712313	0.62712313	7.53665479	7.59547832	7.53665479	10	
dave.wadden	3328877	reg_survival	1	6	485	0.60232781	0.62735734	0.63846686	6.07810632	7.60925516	8.20393323	11	
Wojciech Les	3324341	reg_survival	1	1	485	0.54877602	0.55769622	0.58336728	2.92800102	3.5115426	4.96278096	12	
Davide Chicc	3330147	reg_survival	159	229	485	0.48583561	0.47958332	0.48136532	-0.774376	-1.0833343	-1.037334	13	

Table 5.5- Full results- Registry progression sub-challenge

Name	ID	Challenge	#cluster	#feature-sets	#patients	score1	score2	score3	z1	z2	z3	rank	
baseline 1						0.579:0.024	0.182:0.062	0.770:0.046	13.483:2.563				
baseline 2						0.595:0.023	0.289:0.074	0.702:0.046	17.734:2.839				0.784
Jinfeng Xiao (	3325593	reg_progression	6	3	223	0.58691756	0.30122593	0.69844703	3.86598099	4.4959094	-9.3015134	1	17.6634038
Xiaotao Liang	3327729	reg_progression	1	1	223	0.58623485	0.242646	0.710228	3.83629793	3.62158209	-8.9649143	2	16.4227943
Christoph Kur	3325789	reg_progression	1	1	223	0.58922171	0.22073233	0.70540905	3.96616131	3.29451239	-9.1025986	3	
Wenwen Min	1961142	reg_progression	1	1	223	0.58269329	0.23355917	0.71639097	3.68231706	3.48595776	-8.7888295	4	
Yuanfang Gua	3319559	reg_progression	1	1	223	0.58614951	0.21139201	0.71234665	3.83258755	3.15510463	-8.9043815	5	
Samad Jahar	3323761	reg_progression	1	1	223	0.56575354	0.16906806	0.73675188	2.94580615	2.52340388	-8.2070893	7	
Yu-Jia Shiah (	3328928	reg_progression	1	1	223	0.56652159	0.14326683	0.73212892	2.9791996	2.1383109	-8.3391737	8	
DREAMer	3321630	reg_progression	1	2	223	0.5432241	0.13894572	0.71709444	1.9662652	2.07381672	-8.7687302	9	
Omar (OmarY	3323587	reg_progression	1	1	223	0.54898447	0.12191409	0.74184206	2.21671601	1.81961328	-8.0616553	10	
Wing Chung W	3328096	reg_progression	1	1	223	0.5582864	0.05786091	0.76667326	2.6211477	0.86359567	-7.3521925	11	
Jaume Bacar	3323880	reg_progression	4	2	223	0.50136542	0.00087172	0.83912375	0.14632264	0.01301075	-5.2821785	12	
Xiang Yu (ope	3327120	reg_progression	1	1	223	0.47819594	-0.0840033	0.80306458	-0.8610462	-1.2537801	-6.3124405	13	
Davide Chicc	3330147	reg_progression	131	125	223	0.38807817	-0.0223127	1.0869212	-4.77921	-0.3330257	1.79774848	14	
Wojciech Les	3324341	reg_progression	1	1	223	0.13816351	-0.1158553	0.75118308	-15.645065	-1.7291837	-7.7947692	15	
Rama Ragha	3327490	reg_progression	3	3	223	0.56993514	0.07193702	1.55735989	3.12761489	1.07368687	15.2388539	16	
dave.wadden	3328877	reg_progression	1	1	223	0.00887523	0.05056056	1.15929436	-21.266294	0.75463522	3.86555303	17	

Aggregated predictions*:*

It was previously shown that combining the predictions of several algorithms can often increase the performance of each algorithm individually and even outperform the top ranking algorithms [Marbach_Stolovitzky_PNAS_2010]. We aggregated predictions of all algorithms for each sub-challenge and scored the resulting averaged predictions according to the same metrics used for evaluation of the original submissions (see online methods). The aggregated predictions were extraordinarily robust, always outperforming the individual methods and often outperforming the best team (Figure 3). A more detailed inspection of the performance of the aggregate reveals an interesting benefit for clustering. In both the PRO-ACT progression and PRO-ACT survival sub-challenges we observe an increase in performance of the aggregate with the addition of a method that used clustering for prediction (team ranked 6 and teams ranked 2 & 3, respectively) . The best performing team in the registry progression sub-challenge used clustering in their prediction algorithm and we see little, if any, improvement above this level of performance with the aggregation of additional (non-clustering) methods.

Figure 5.1- Performance of aggregated predictions.

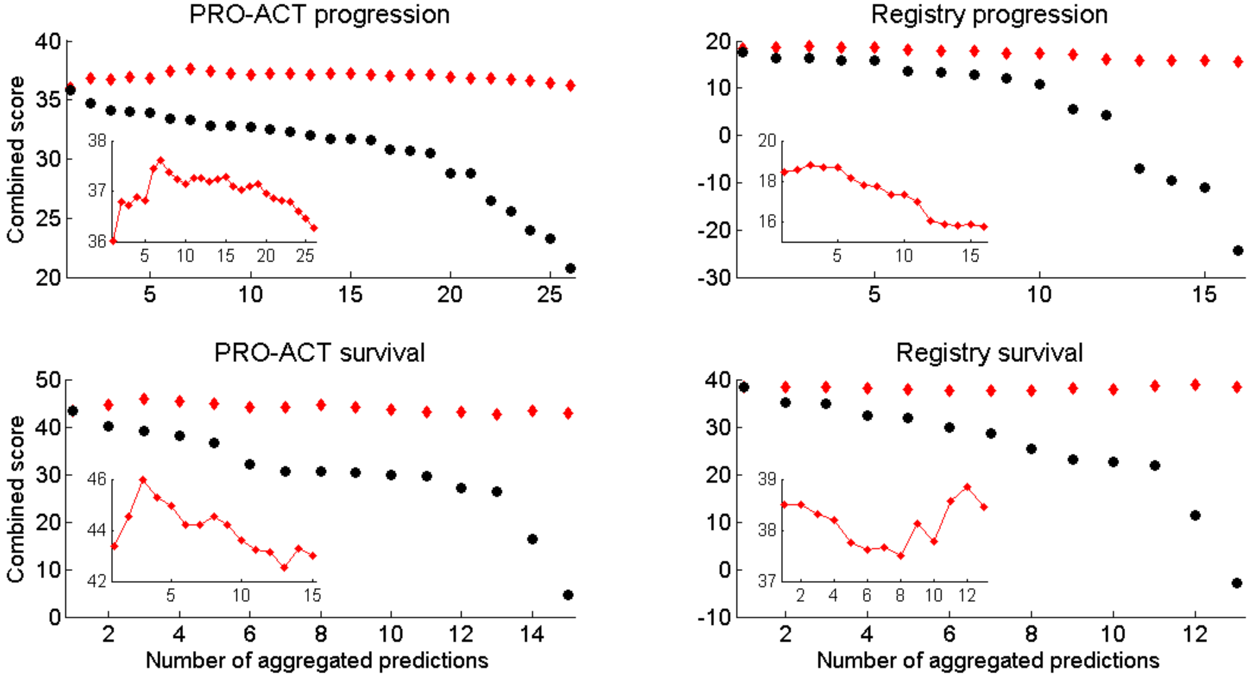

The black circles represent the combined z-score of individual teams, rank-ordered according to prediction accuracy from best (team number 1) to worst performers. Red diamonds correspond to the combined z-scores of aggregated predictions, obtained by averaging the predictions of the two best teams, the three best teams, the four best teams, etc. Insets show scores of the aggregated predictions only on a zoomed-in scale to better visualize the relative effect each team has on performance of the aggregated prediction.

Detailed aggregate results - each individual score and then the combined score. Inset shows the aggregate scores only (for all teams)

Figure 5.2- Aggregated performance for the PRO-ACT prediction sub-challenge

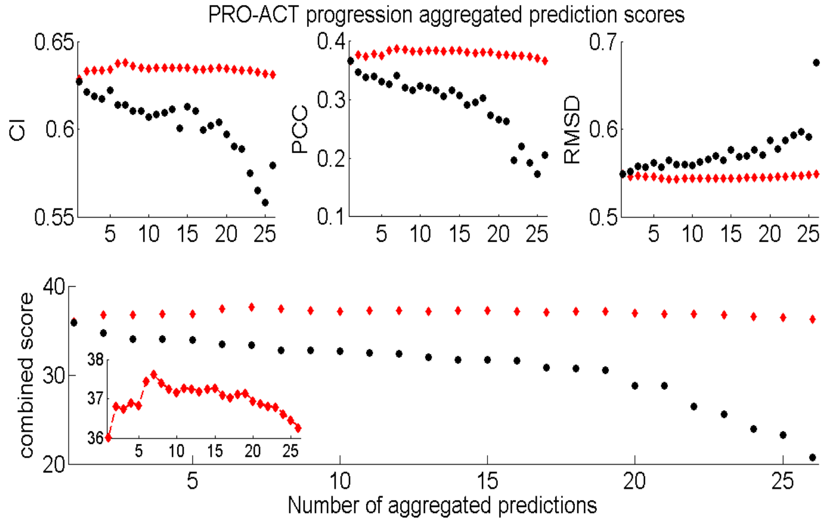

Figure 5.3- Aggregated performance for the Registry prediction sub-challenge

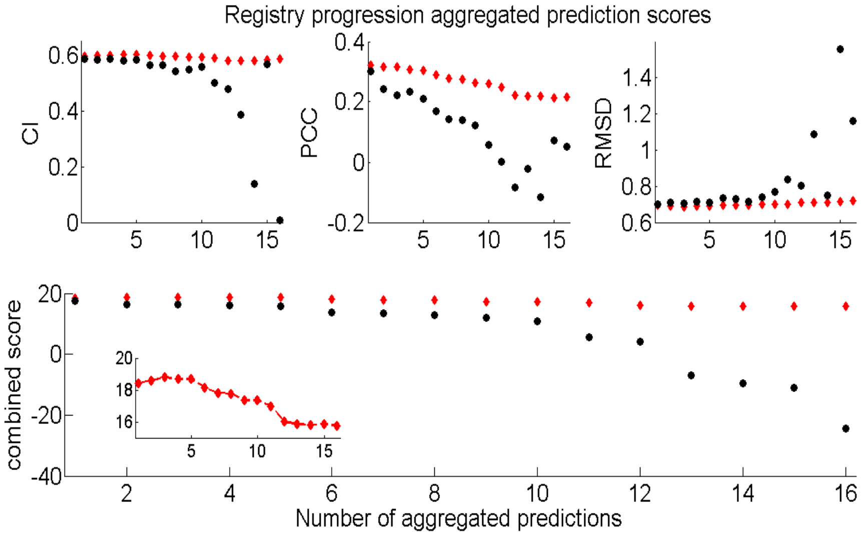

Figure 5.4-Aggregated performance for the PRO-ACT survival sub-challenge

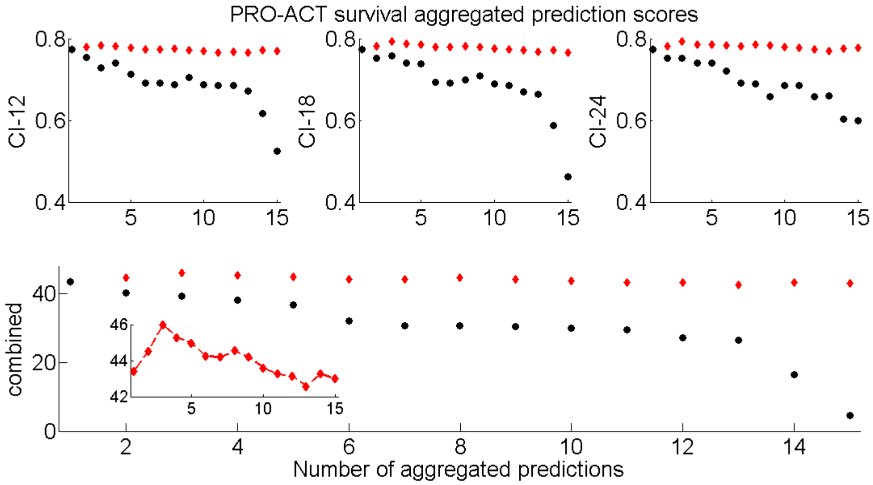

Figure 5.5-Aggregated performance for the registry survival sub-challenge

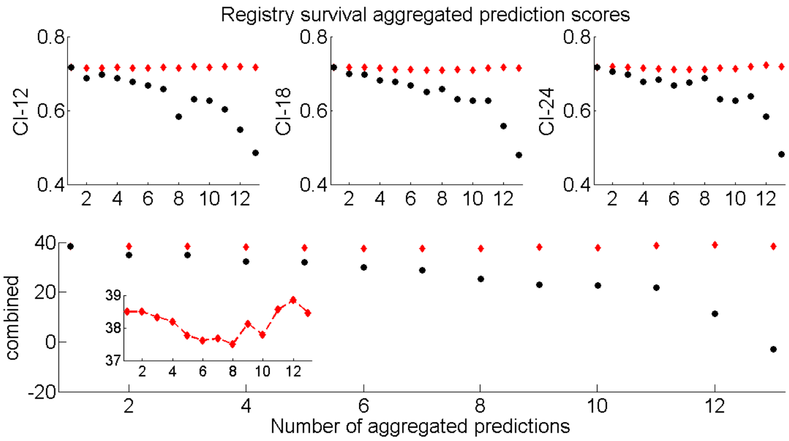

Registry survival aggregated prediction scores
CI-12
CI-18
CI-24
combined
Number of aggregated predictions

Supplementary material part 6- Examples for correlation with prognosis occurring only in one cluster

Below we show the correlation discussed in the main text, between features, assessed separately for each cluster, and disease progression. All data is derived from the sub-challenge around predicting disease progression using the PRO-ACT database, where 4 consensus clusters were identified and significantly different from each other.

The examples only include cases where correlation was significant and only found for one cluster.

Correlation is shown for all four clusters, with the relevant cluster is marked with a square.

Figure 6.1- Example 1- correlation between Trunk ALSFRS measurements and disease progression are only visible in “late stage” or "green" cluster

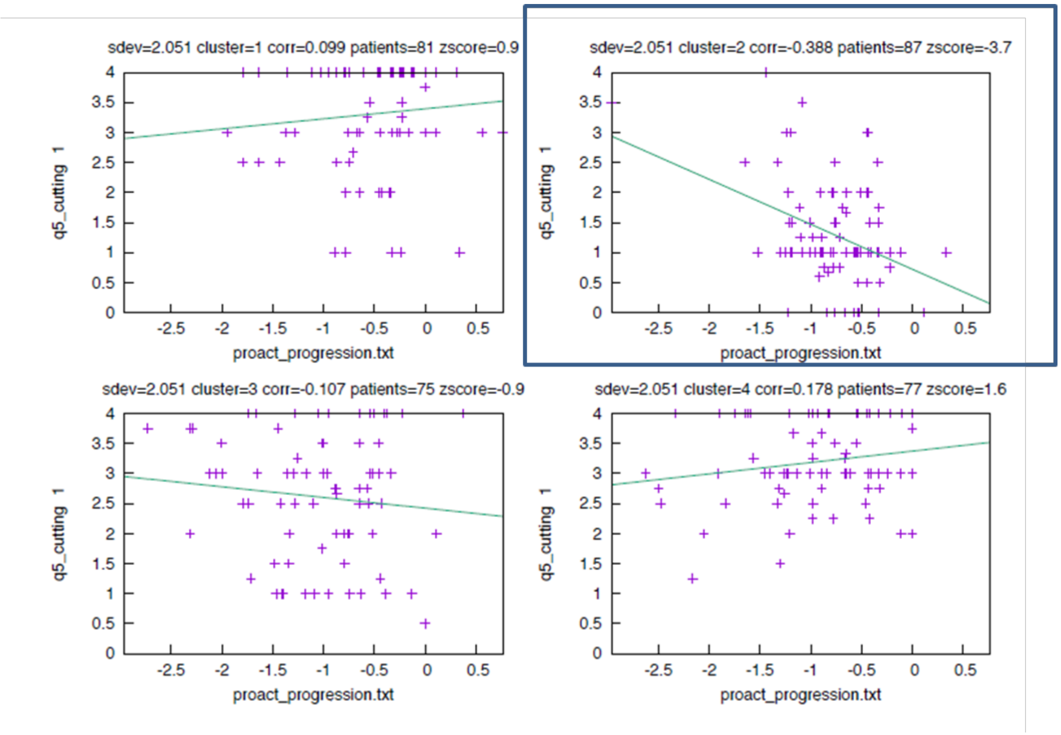

Figure 6.2- Example 2- correlation between respiratory functions and disease progression are only visible in "good prognosis" or "red" cluster

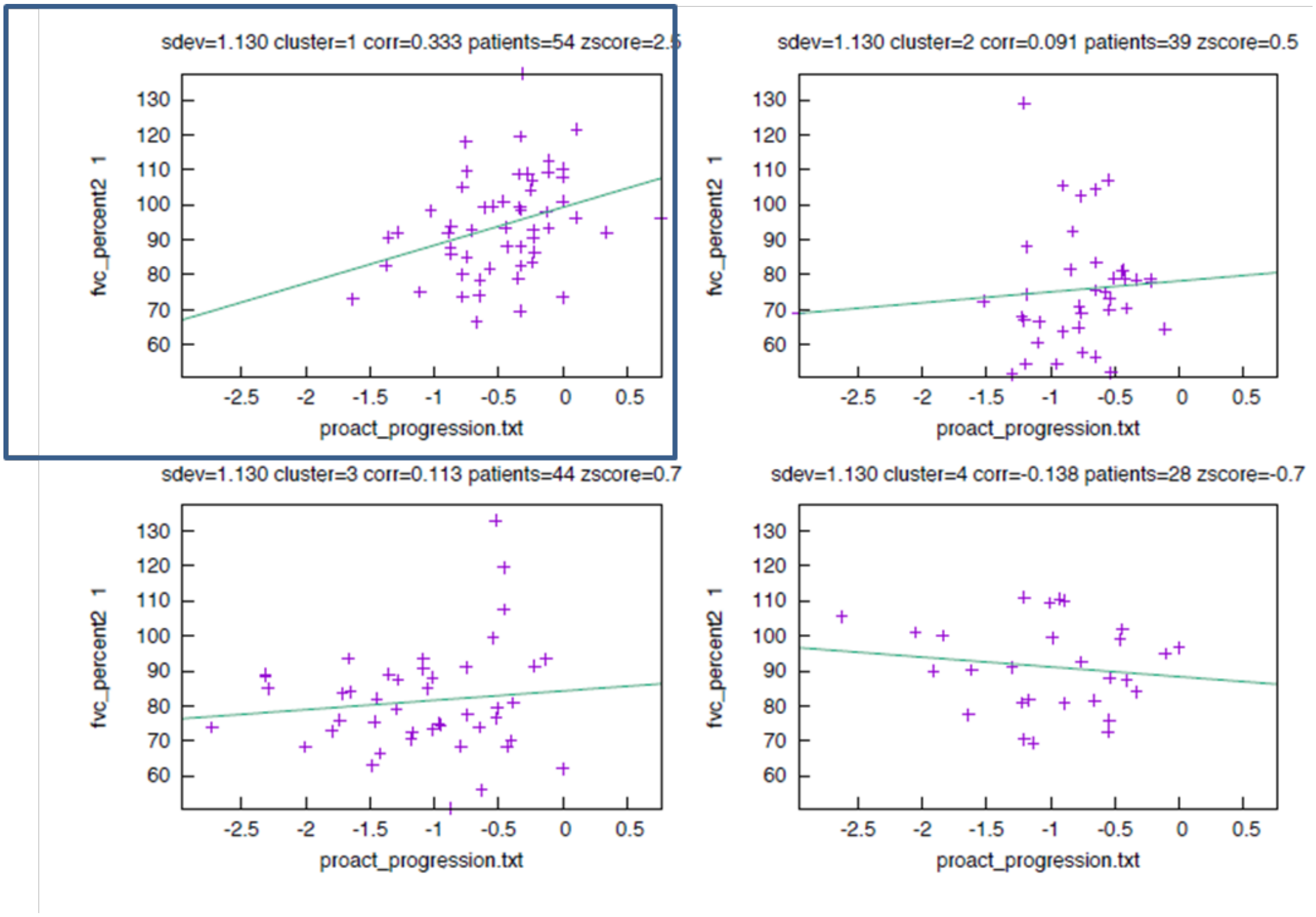

Figure 6.3- Example 3- correlation between creatinine levels in serum and disease progression are only visible in “early stage” or "purple" cluster

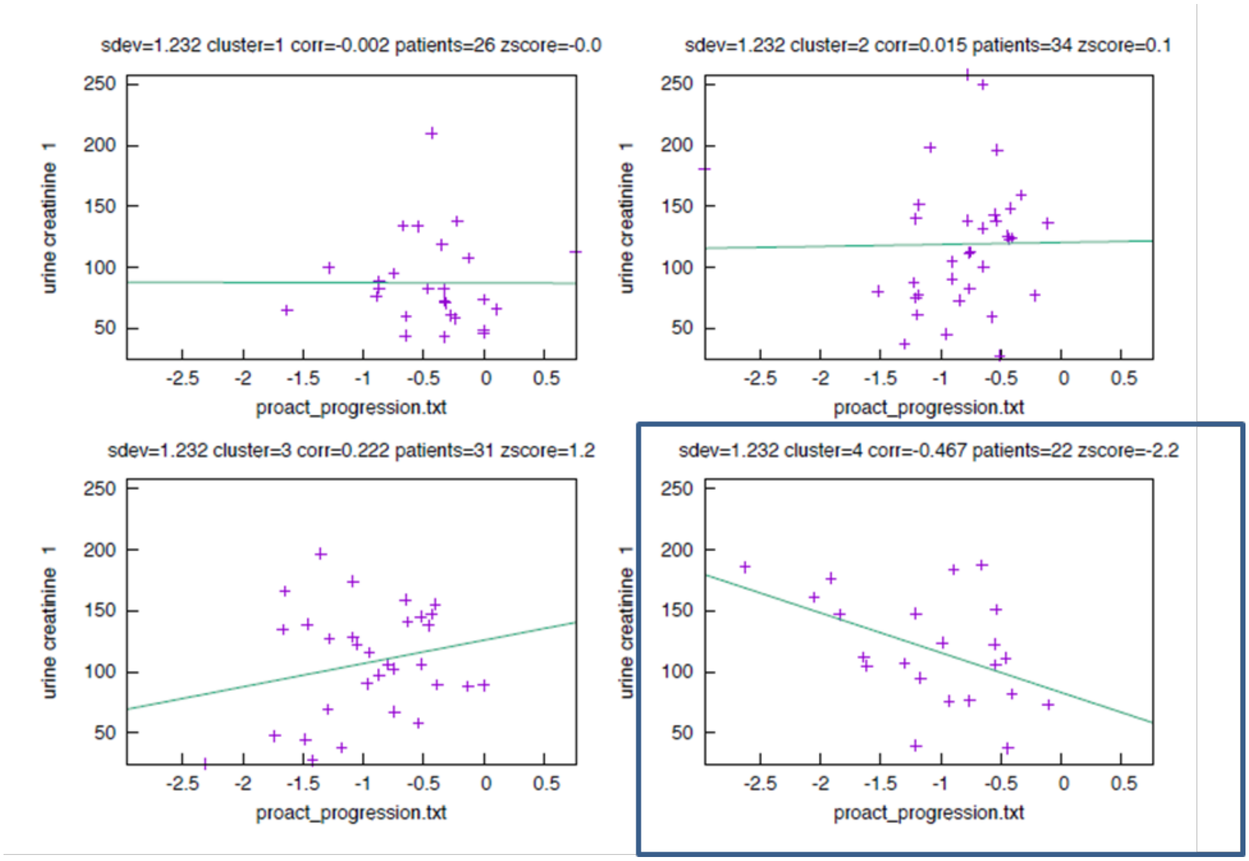

Pairwise comparison between features

We also assessed the differences between the clusters using ANOVA to assess pair wise differences for specific features (figure 4c in main text).

One difference described in the main text are features whose values are distinctly different only for a certain cluster. In the examples below the full data across of cluster patients is presented to demonstrate the consistency of patient clusters.

Figure 6.4 Features especially predictive across clusters

We determined features whose values were the most important for classification(figure 4c)- that were significantly difference in our pair-wise comparison among the most clusters. Unsurprisingly the most predictive feature was time of onset (which was significantly difference between the "red", "blue" and "green" clusters). However, equally discriminative were also ALSFRS question 1 (speech) and our combined measure mouth, averaging ALSFRS questions 1-3 (speech, swallowing and salivation), highlighting again the important

role of bulbar function in discriminating ALS consensus clusters. The distribution of values across the patients in each cluster our outlined below

Feature most distinct among clusters was onset delta

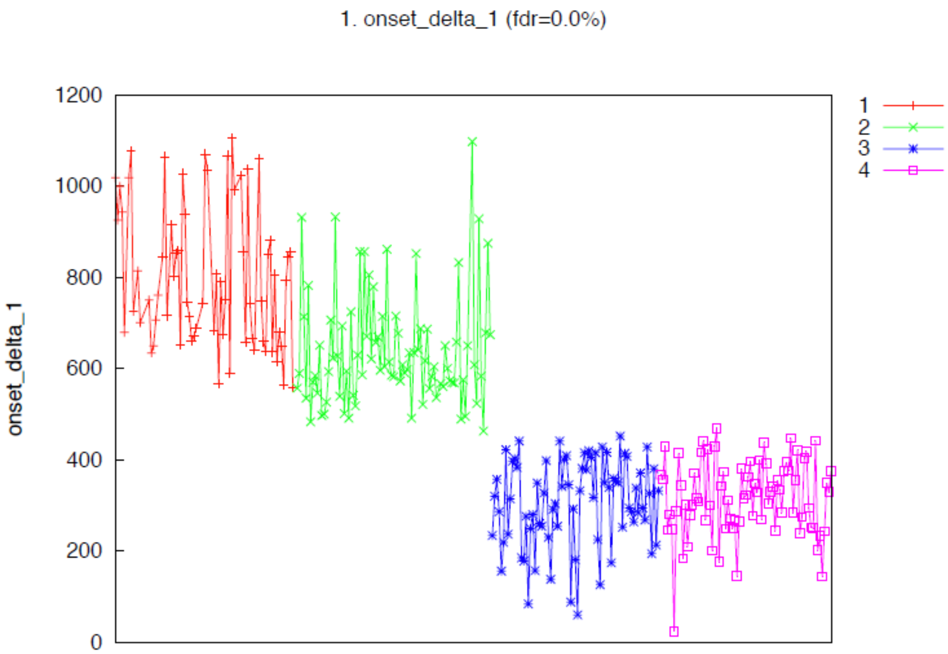

Second most distinct was ALSFRS total for the first 3 months:

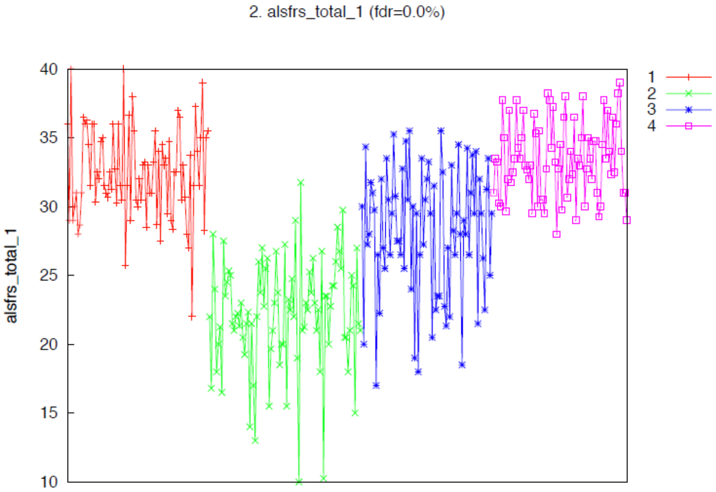

Third most distinct was ALSFRS Q1 (speech)

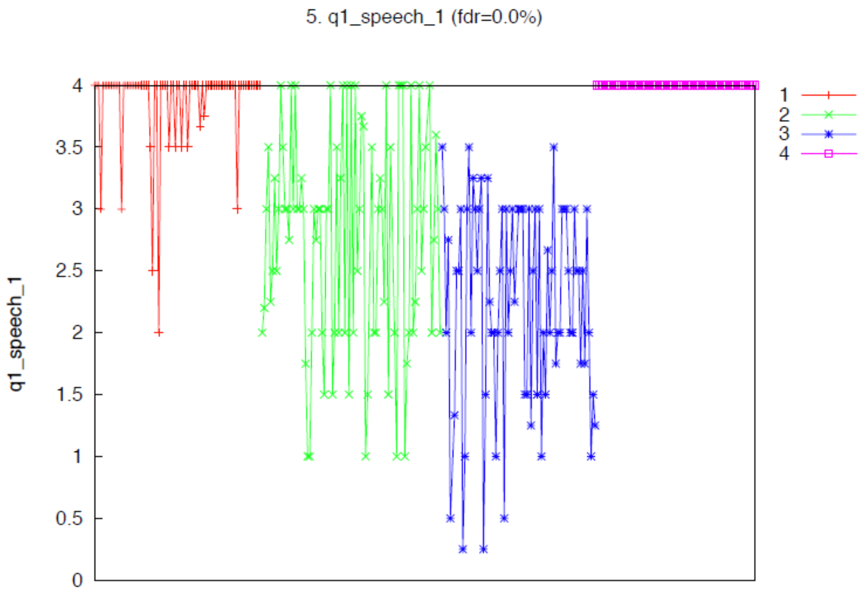

Fourth was the combined ASLFRS "mouth" scores (ALSFRS Q1-3, summed):

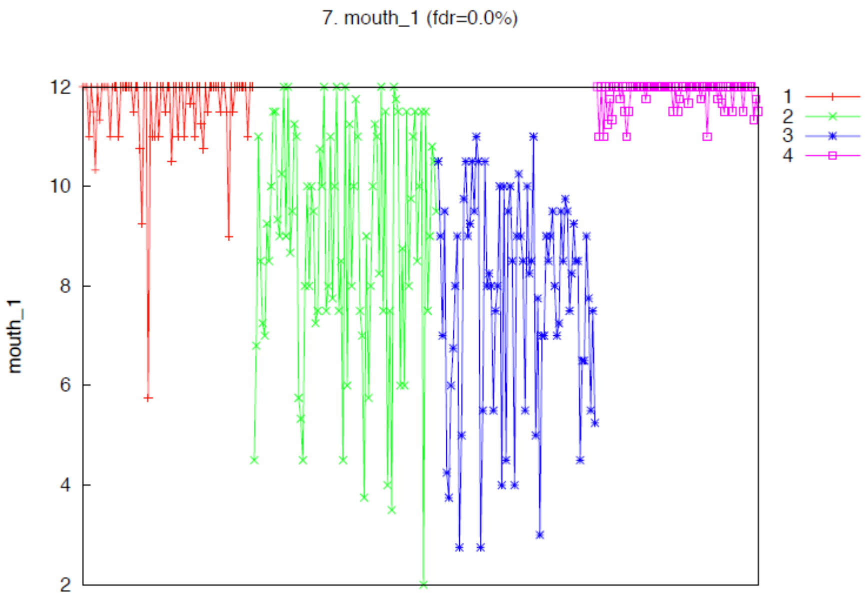

Figure 6.5 Features distinct only for the "late stage" or "green" cluster

Feature distinctly different for the "green" cluster alone includes:

(1) "trunk" scores, a combine measure including the average on ALSFRS question #6 and #7 ("turning in bed" and "dressing and Hygiene" respectively). This score was created in the data processing for the challenge and

was available for participants. Plots are available for the combined score, as well as for "turning in bed" and "dressing and hygiene" separately

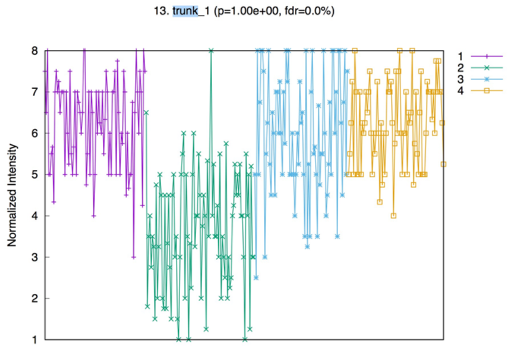

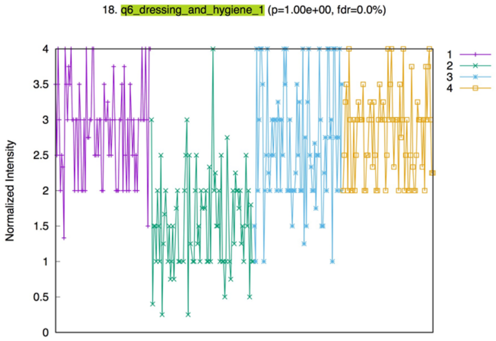

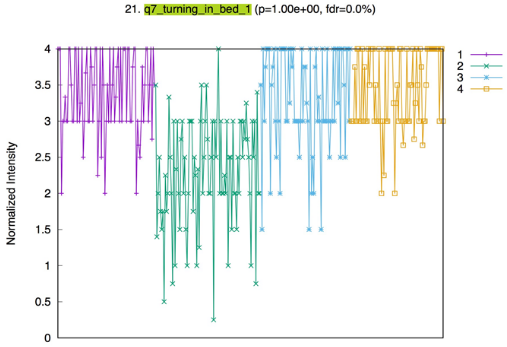

(2) Slow Vital capacity (SVC), a measurement of respiratory function. Routinely. SVC is assessed in three seperately repeating assessment and either the average measure or the highest assessment is used. In the example below all three assessment can be shown.

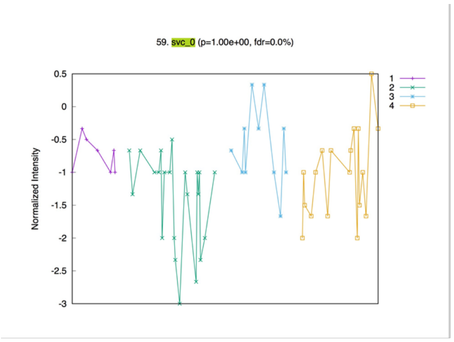
59. svc_0 (p=1.00e+00, fdr=0.0%)
Normalized Intensity
0.5
0
-0.5
-1
-1.5
-2
-2.5
-3
1
2
3
4

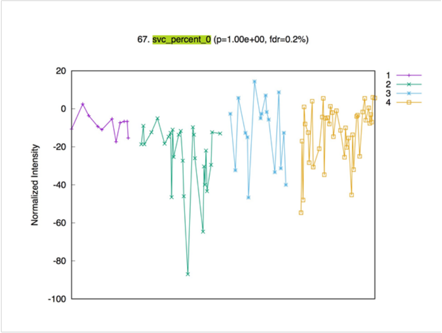
67. svc_percent_0 (p=1.00e+00, fdr=0.2%)
Normalized Intensity
20
0
-20
-40
-60
-80
-100
1
2
3
4

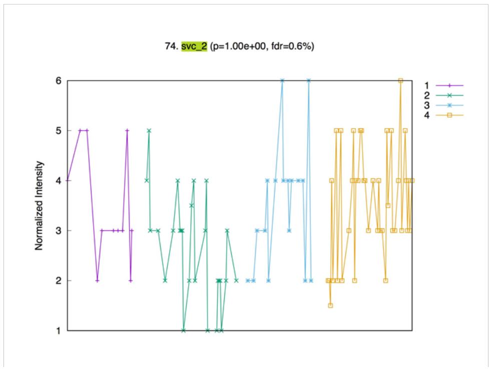
74. svc_2 (p=1.00e+00, fdr=0.6%)
Normalized Intensity
6
5
4
3
2
1
1
2
3
4
