# Supplementary material for: Stratification of amyotrophic lateral sclerosis patients: a crowdsourcing approach
Source: Sci Rep. 2019 Jan 24;9:690. doi: 10.1038/s41598-018-36873-4 (PMC6345935; doi:10.1038/s41598-018-36873-4)
Supplement: Supplementary file 2 — Supplementary Info 2 [file 41598_2018_36873_MOESM2_ESM.pdf]

Supplementary Material: writeups of the methods of all challenge participants

Stratification of amyotrophic lateral sclerosis patients: a crowdsourcing approach

Robert Kueffner, Neta Zach, Maya Bronfeld, Raquel Norel, Nazem Atassi, Venkat Balagurusamy, Barbara Di Camillo, Adriano Chio, Merit Cudkowicz, Donna Dillenberger, Javier Garcia-Garcia, Orla Hardiman, Bruce Hoff, Joshua Knight, Melanie L. Leitner, Guang Li, Lara Mangravite, Thea Norman, Liuxia Wang, the ALS Stratification Consortium, Jinfeng Xiao, Wen-Chieh Fang, Jian Peng, Chen Yang, Huan-Jui Chang and Gustavo Stolovitzky

Yuanfang Guan's solution to 2015 Prize4Life-DREAM ALS challenge
- Summary:
- Background:
- Methods:
- Conclusion:
- Author's statement:

JayHawks: ALS Stratification Prediction Modeling with CART-based Clustering of Subgroups
- Background/Introduction
- Methods
  - Data Cleaning and Variable Selection
  - Clustering
  - Model Training
  - Final Competitive Models
  - Submission Format
- Discussion
- Authors Statement
- Acknowledgements

ALS Stratification Challenge, team Als_UoB
- General methods
  - Feature construction
  - Feature selection
    - Step 1
    - Step 2.
    - Step 3.
  - Model building
  - Clustering
- Model for Question 1.
- Model for Question 2.
- Model for Question 3.
- Model for Question 4.
- References

ZhuLab: Solution for ALS Stratification Prize4Life Challenge
- Introduction

- Methods
  - Data pre-processing
  - Feature selection
  - Data imputation
  - Running the final model
- Conclusion/Discussion
- References
- Authors Statement

Team chk: A Model for Predicting ALSFRS Slope and Survival of ALS Patients
- Abstract
- Introduction
- Methods
- Insights gained

The ICOS submission to the DREAM ALS stratification challenge: RGIFE+RandomForest+k-means/hierarchical clustering
- Background/Intro
- Methods
  - The RGIFE feature reduction method
  - Data cleaning and feature generation
  - Generation of training and validation sets.
  - First RGIFE stage: Global feature reduction
  - Clustering of patients
  - Second RGIFE stage: finding a different set of variables for each cluster
  - Selection of the final clustering using the validation set.
  - Generation of confidence intervals/probabilities for the submitted predictor
  - Code
  - Clinical variables used for each challenge
    - Sub-challenge 1.
    - Sub-challenge 2.
    - Sub-challenge 3.
    - Sub-challenge 4.
- Conclusion/Discussion
- References
- Authors Statement
- Acknowledgements

Team Donuts ALS Stratification Challenge
- Introduction
- Methods

Featurization
Feature selection
ProAct Models
Registry Models

Team DreamWeaver: Tree-based solution to the ALS Stratification Prize4Life Challenge
Background/Intro
Methods
Data pre-processing
Data Cleansing
Construction of the training set
Selector
Results
Predictor
Discussion
References
Authors Statement

TeamSimon_ALS: DREAM ALS stratification Prize4Life Challenge

Team UglyDuckling: A Boosting approach and Cox model for Predicting Slope and Survival of ALS
Abstract
Introduction
Methods
Data pre-processing
Feature selection
ALSFRS Slope Prediction
Survival Probability Prediction
Conclusion
Instructions for Source Code
Code Guidance
Orange Operation
References
Authors Statement
Acknowledgements

Team chern: Modelling ALS progression using time-dependent Hurst exponent
Introduction
Methods
Conclusion

References

Team CompassRose: Feature Selection and Prediction in ALS Disease Progression
Background and Introduction
Methods
Variable Selection and Transformation
Static data
Episodic data
Time Series data
Differences in the Registry data
Modeling and Feature Selection
Variable Selection
Prediction
Conclusion/Discussion
Authors Statement
References

Team dinithins: ALS Survival Prediction With Decision Trees
Introduction
Methods
Background of data
Data Preprocessing
Method
Discussion
References
Authors Statement
Acknowledgements

Team openneuron: Model using Bayesian Additive Regression Trees for predicting ALS slope and survival
Abstract
Introduction
Method
Data preprocessing
PROACT dataset
National Registries dataset
imputation and cluster
Feature selection in each cluster
Model building
Model predictor
Discussion

Author statement
Reference
Team Davide Chicco: A k-means clustering method for prediction of ALS slope progression and survival
Background/Intro
Methods
Conclusion/Discussion
References
Authors Statement
Re-execute tests
Team zsh-2015: A random forests model for predicting survival of ALS patients using PRO-ACT database
Summary
Introduction
Data preprocessing
Model
Feature section
Discussion
Author Statement
Reference
DREAM ALS Stratification Prize4Life Challenge: Team ALS-Theodore
Data Processing
Methods
Sub-challenge 1:
Sub-challenge 2:
Conclusion/Discussion
Code
Acknowledgements
ML-BGU: A layered random forest approach for clustering ALS patients and predicting disease progression
Introduction
Methods
Feature Selection
Feature extraction
Missing data
Splitting the database
First Layer - dimension reduction and clustering
Second Layer - cluster specific predictor importance and training of the models

Selector
Predictor
Compiling the source code
Discussion

Team Jyguo: Model ALS disease progression using sigmoid function
Introduction
Methods
Conclusion/Discussion
References

Team Jinfeng Xiao: Random Forest Based Solution to ALS Stratification Challenge
Introduction
Methods
1. Put together all data for each patient.
2. Encode covariants into features
A) Static categorical covariates with uniform delta
B) Static continuous covariates with uniform delta
C) Longitudinal covariates
3. Fill in missing data
4. Feature selection and prediction
A) Sub-challenges 1 & 3: Weighed path across the forest
B) Sub-challenges 2 & 4: Importance function of randomForestSRC
Results
Sub-Challenge 1
Sub-Challenge 2
Sub-Challenge 3
Sub-Challenge 4
References
Acknowledgements

Team barsinai: A Combined Model for predicting ALSFRS slope
Abstract
Background/Intro
Feature Generation
Feature Selection
Prediction
Methods
Conclusion/Discussion
References

Team Veltys: A Mixture Model for Predicting ALSFRS Slope
1- Introduction
2- Methods
  2.1 - Data pre-processing
  2.2 - Statistical models
  2.3 - Fitting the model
  2.4 - Predicting the ALSFRS slope
 3- Discussion
 4- Running scripts
 References
Team uci-cbcl: An Ensemble Method for Predicting Amyotrophic Lateral Sclerosis Progression
  Abstract
 Introduction
 Methods
   Regression models
   Data pre-processing
   Feature selection
   Stratification
   Ensemble model
 Conclusion
 References
 Authors Statement
Team jjacob_cub: A model for prediction of progression slope in newly diagnosed ALS patients. (Submission for subchallenge 1)
Team Wonwingchung: Lasso regression model on predicting the progression on ALS patients
 Introduction
 Method
  Handling longitudinal features
  Data filtering
  Handling missing value
  Data normalization
  Model
  Model performance evaluation
  Program execution
 Conclusion & Discussion
  Limitation
  Further Enhancement

- Author statement
- References

Team Crazy Alvin
- 1. Background
- 2. Methods
    - 2.1 Data Pre-Processing
    - 2.2 Compiling and Running Code
        - TRAINING
        - TESTING
- 3. Conclusion/Discussion
- References
- 4. Authors Statement

Team Veristat
- Title: A Bayesian Tree Model for Predicting the ALSFRS Slope using the PROACT dataset
- INTRODUCTION:
- METHODS:
    - OVERVIEW:
    - DATA PROCESSING:
    - VARIABLE SELECTION
- Final Model:
- CONCLUSION /DISCUSSION:

Yuanfang Guan's solution to 2015 Prize4Life-DREAM ALS challenge

Yuanfang Guan

Department of Computational Medicine and Bioinformatics, University of Michigan, Ann Arbor

Will you be able to make your submission public as part of the challenge archive? Yes

Summary:

This is the winning solution for both sub-challenges concerning survival prediction. I wrote solutions for all four sub-challenges. For sub 2 and 4, I predicted death. My final submission was on 10/04/2015, with the label 'final5'.

Background:

Please see https://www.synapse.org/#!Synapse:syn2873386/wiki/391426

Methods:

For each, I trained a Gaussian Process Regression to predict outcomes. For all sub-challenges, a single cluster was used. In GRP, instead of standard SE kernel, I took the arithmetic mean instead of geometric mean across dimensions.

Used Gaussian Process Regression to predict outcomes. In GRP, instead of standard SE kernel, I took the arithmetic mean instead of geometric mean across dimensions. For survival data, I invented a probabilistic ranking of all training patients based on K-M curves, which can be built into any base-learner. For full details see http://guanlab.ccmb.med.umich.edu/yuanfang/Guan_rank.pdf

A thorough explanation of GPR in addressing heterogeneity across multiple data cohorts is included in https://www.synapse.org/#!Synapse:syn2368045/wiki/64596 which was first introduced to my lab by my previous postdoc Fan Zhu, who worked his Ph.D. thesis on tuning GPR weights. The key is to address the issues when multiple cohorts display different missing data and demographics. Fan later worked

with me using GPR for Alzheimer's Disease Diagnosis prediction (Sub-challenge 3) and further description is included in https://www.synapse.org/#!Synapse:syn2527678/wiki/69937 I chose GPR in this challenge for the same reason for the two cohorts. The implementation was from scratch from https://www.synapse.org/#!Synapse:syn5298211, https://www.synapse.org/#!Synapse:syn5298209, https://www.synapse.org/#!Synapse:syn5298210

I prefer my own implementation, as public implementation in other software cannot achieve this performance. It is runnable in dockerized Octave (free)

Missing data: imputed with average.

For survival data, I invented a probabilistic ranking of all training patients based on K-M curves, which allows me to build in any base-learner (thus the method can be independent from Cox or Survival random forest). The method of this ranking and my presentations at the RECOMB conference are included in https://docs.google.com/presentation/d/1Ke3TuJmEkmfB6x-sHKn8RbPH6_YhhgZ6swRUiKV2WWo/edit#slide=id.g21a95fd849_0_0 (May 15, 2017 updated version corrected a small previous bug)

Feature selection: top features excluding overlapping ones; e.g. if bulbar/onset_site is selected, limb/onset_site is excluded.

For Sub 1, the six features are: ALSFRS_Total, fvc_percent, onset_delta, onset_site, Q1_Speech, Q3_Swallowing.

For Sub 2, the six features are: Age, ALSFRS_Total, fvc1, fvc_percent, Q3_Swallowing, weight.

For Sub 3, the features were the same, as sub 1. As was found in later stage that ALSFRS_R_Total, instead of ALSFRS was the prediction target, I multiplied the prediction result by 1.07 to compensate for this scaling.

For Sub 4, the six features are: Age, ALSFRS_R_Total, onset_site, mouth, respiratory, onset_delta

Conclusion:

The method turns out to rank number 1 (winning solution) in both survival challenges.

Author's statement:

Yuanfang Guan designed and implemented the method

JayHawks: ALS Stratification Prediction Modeling with CART-based Clustering of Subgroups

Authors: Rama Raghavan[1], Richard Meier[1], Janelle R. Noel[1], Junquang Dai[1], Stefan Graw[1], Alex Karanevich[1], Joseph Usset[1], Devin C. Koestler[1], Brooke L. Fridley[1]*
Affiliations: [1]Department of Biostatistics, The University of Kansas Medical Center, Kansas City, KS, USA

ALS Stratification Prediction Modeling with CART-based Clustering of Subgroups
Background/Introduction
Methods
Data Cleaning and Variable Selection
Clustering
Model Training
Final Competitive Models
Submission Format
Discussion
Authors Statement
Acknowledgements

Background/Introduction

Our primary objective was to expand on the work that was done in the 2012 ALS Prediction Challenge. Specifically, we aimed to develop prediction models that accounted for potential subgroups of patients for the Pooled Resources Open-Access Clinical Trail (PRO-ACT) and National Registries datasets that had the capability of predicting ALSFRS slope from 3-12 months (Subchallenges 1 & 3) and probability of survival within 0-12 month, 0-18 months and 0-24 months (Subchallenges 2 & 4). Our team performed all of our analysis using R statistical software and have uploaded our source code to Github (https://github.com/richard-meier/JayHawks_ALS-Stratification-Prize4Life-Challenge).
Following the constructs of the challenge, our methodologies consisted of a clustering component and a model prediction component. Prior to implementation of the clustering of model building, we sought to clean the data sets and make some decisions on what types of variables might be most suitable for the aforementioned components. After data cleaning, our team investigated many different scenario combinations—combinations of clustering techniques and different prediction models. The clustering techniques that we examined to detect potential subgroups were principle components analysis (PCA), recursively partitioned mixture models (RPMM), and classification and regression trees (CART) - based clustering. These clustering methods used “stat”, “rpmm”, and “rpart” & “OIsurv” R packages, respectively. Concurrently, for ALSFRS slope prediction we studied many linear models that were built using variable and/or variable interaction inclusion/exclusion processes. ALSFRS slope models were evaluated in terms

of root mean squared error (RMSE) using cross-validation. The prediction models for the probability of survival in Subchallenges 2 & 4 consisted of Cox proportional hazard models (Cox-PH models) which also used variable and/or variable interaction inclusion/exclusion processes. All of the Cox-PH models were fit using the coxph function in the “survival” R package. Probability of survival models were assessed using their integrated area under the curve (iAUC) and c-Index using cross-validation.

Early in the competition, it was decided to treat the respective slope and survival probability questions in the same way regardless of the dataset that was being used. Throughout the leaderboard submission rounds for Subchallenge 1, we noticed that there was only minimal difference between our models based on RPMM clustering. Thus, it was collectively decided that the CART-based clustering method would be implemented for our final submission round for all of the subchallenges. From the CART-based clustering, we were able to see which variables in our dataset were likely leading to potential sub-groups. It should be noted that all of the longitudinal variables that were included in either the clustering or modeling building component were summarized using the 0-3 months mean, median, or minimum value. A quick over-view of our strategy can be found in **Figure 1** below.

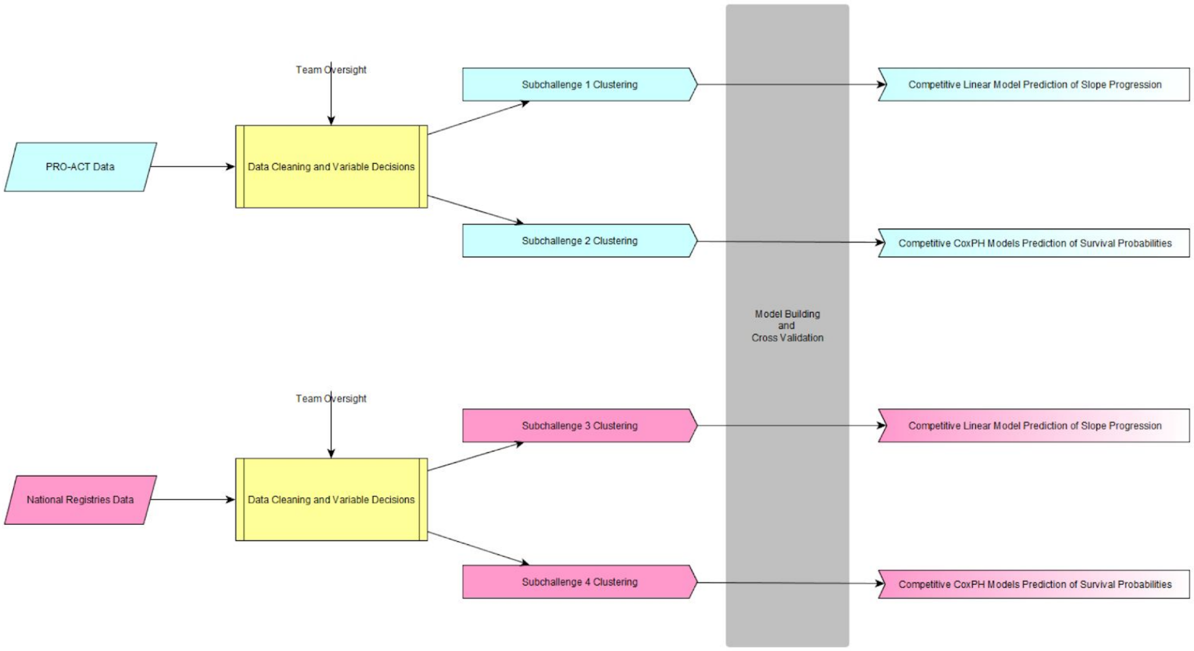

Figure 1: Strategic Workflow for ALS Stratification Challenge

Methods

Data Cleaning and Variable Selection

Our team's scheme for pre-processing the data consisted of 3 steps: 1) filtering existing features and creating critical new features, 2) collapsing longitudinal data to a single estimated value, and 3) imputation for missing data values. In step 1, we immediately removed the "Adverse Events" and "Concomitant Medication" forms from the PRO-ACT data set due to the limited time of the challenge. Furthermore, we removed any existing features with more than 75% missingness in both the PRO-ACT and National Registries data sets. The data sets were subset to only those patients that had the respective outcome of the subchallenge question of interest—ALSFRS slope or survival responses for PRO-ACT and National Registries, respectively. With these narrowed features, we checked all records for each of the features to insure that they had the same feature unit across different time points. We also created a "priorSlope" variable and an "onsetAge" variable to be included in Subchallenge 3 & 4. The "priorSlope" variable is the ALSFRS slope from onset_delta to the maximum available day before the first 3 month of the study, and "onsetAge" is the patient's age in years at onset of disease. "priorSlope" is calculated using "onset_delta" and "ALSFRS_Total" and "onsetAge" is calculated using "Age" and "onset_delta". While cleaning the data we noticed that there were some issues within each of the data sets. We speculated that these issues may have been a product of the way in which the data collection forms were setup or the variation present from data that different sites collected on their respective patients. We discovered that several of the reported slopes for the patients were not calculated as stated in the challenge notes. Specifically, there were several subjects who did not meet the inclusion/exclusion criteria for calculating slope though they were assigned a slope in the provided slopes data set. Concurrently, several subjects who did qualify to have their slope calculated based upon the longitudinal data in the "all_forms" data set were not assigned a slope.

Our step 2 collapses the continuous longitudinal data variables into a single estimated value based upon the mean, median, or minimum value depending on the nature of the feature of interest. Most features were collapsed using their mean. The "ALSFRS_Total" variable, in the PRO-ACT data set, was summarized as the minimum value from 0-3 months. Lastly, collapsed features were imputed. Numerical features were imputed utilizing the k-nearest neighbors' method and than scaled to standard normal, while categorical features were imputed with the mode population level.

Clustering

For leaderboard submissions of Subchallenge 1, we investigated several different clustering techniques. We used PCA, RPMM, and CART- base clustering techniques. We had 4 successful submissions which are summarized in **Tables 1** and **2**. It was noticed that our best submission based upon the scoring criteria was that which PCA was used. PCA determined that 2 clusters likely existed. However, in discussions later in the challenge, our team decided that clustering based on outcome might be more meaningful for finding subgroups that impact prediction. We decided to utilize CART-based clustering for both the slope and survival outcomes in each of the data sets. The CART-based clustering is intuitive to interpret and allows us to easily hardcode a classification rule for new patients after performing clustering on the training set. Our CART classifications for our final submission model can be found in **Figures 2** through **5** below. The boxes around portions of the tree denote a given cluster. The clusters were "hardcoded based upon the classification rules located at each branch leading to the cluster. The trees were cut in such a way that the size of the clusters were deemed appropriate for model building. Different clusters and prediction models were tired and cross validated before the final submission clusters were decided upon. It was assumed that the clusters found from the survival CART-based clustering would hold for all survival probabilities within 0-12 month, 0-18 months and 0-24 months (Subchallenges 2 & 4). In **Figures 3 & 4**, one can observe the differences of the K-M curves with respect to rates of survival. The R code for all of the CART models can be found at the Github Project Page.

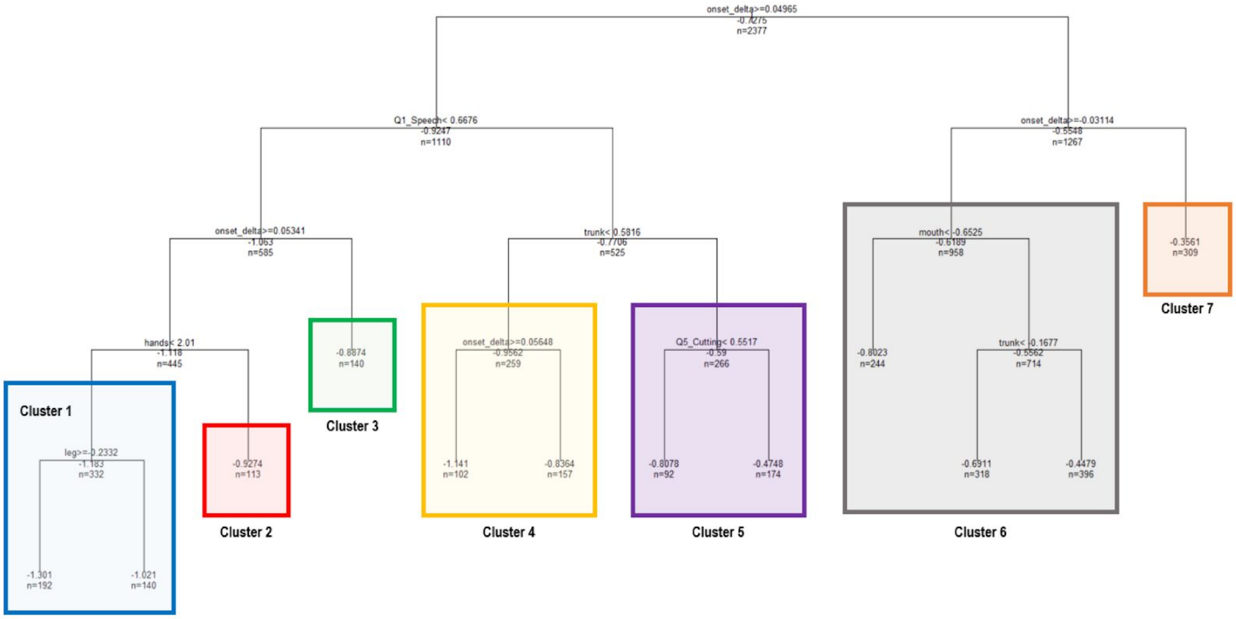

Figure 2: CART for PRO-ACT Slope Progression Prediction (Subchallenge 1)

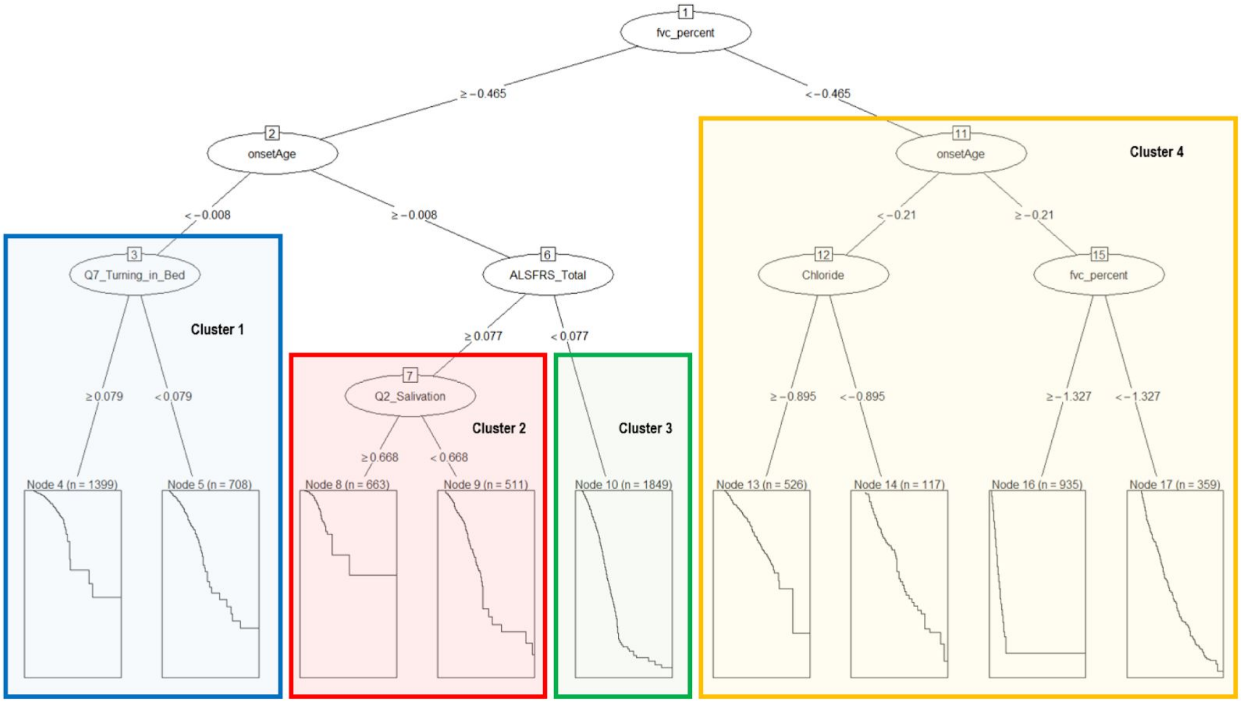

Figure 3: CART for PRO-ACT Survival Probabilities Prediction (Subchallenge 2)

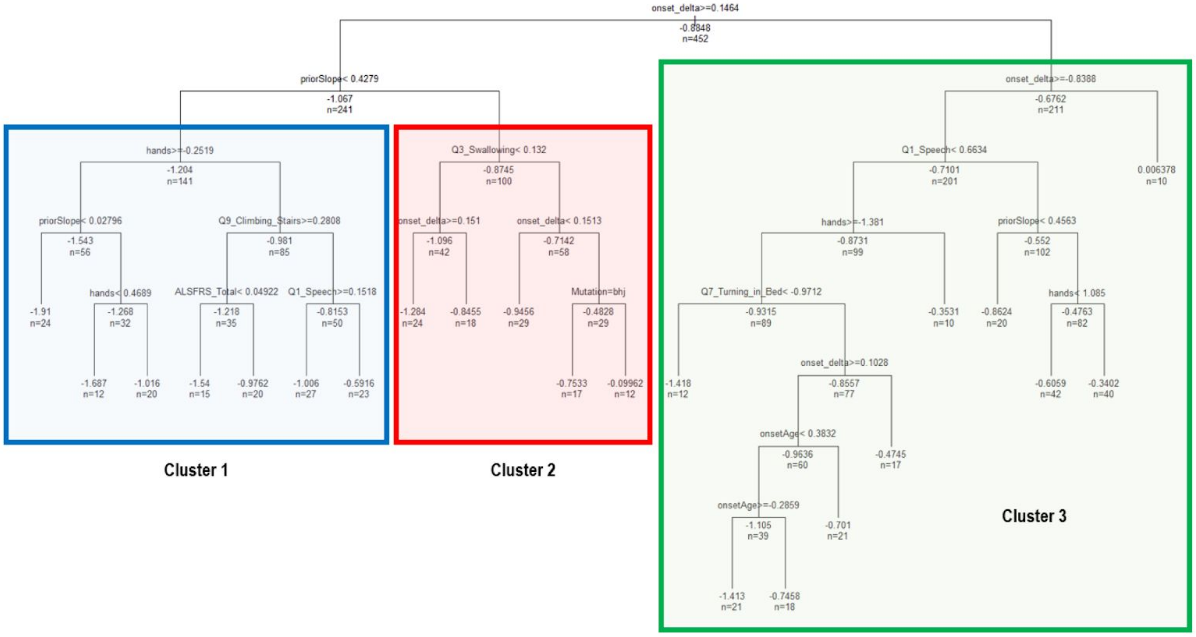

Figure 4: CART for National Registries Slope Progression Prediction (Subchallenge 3)

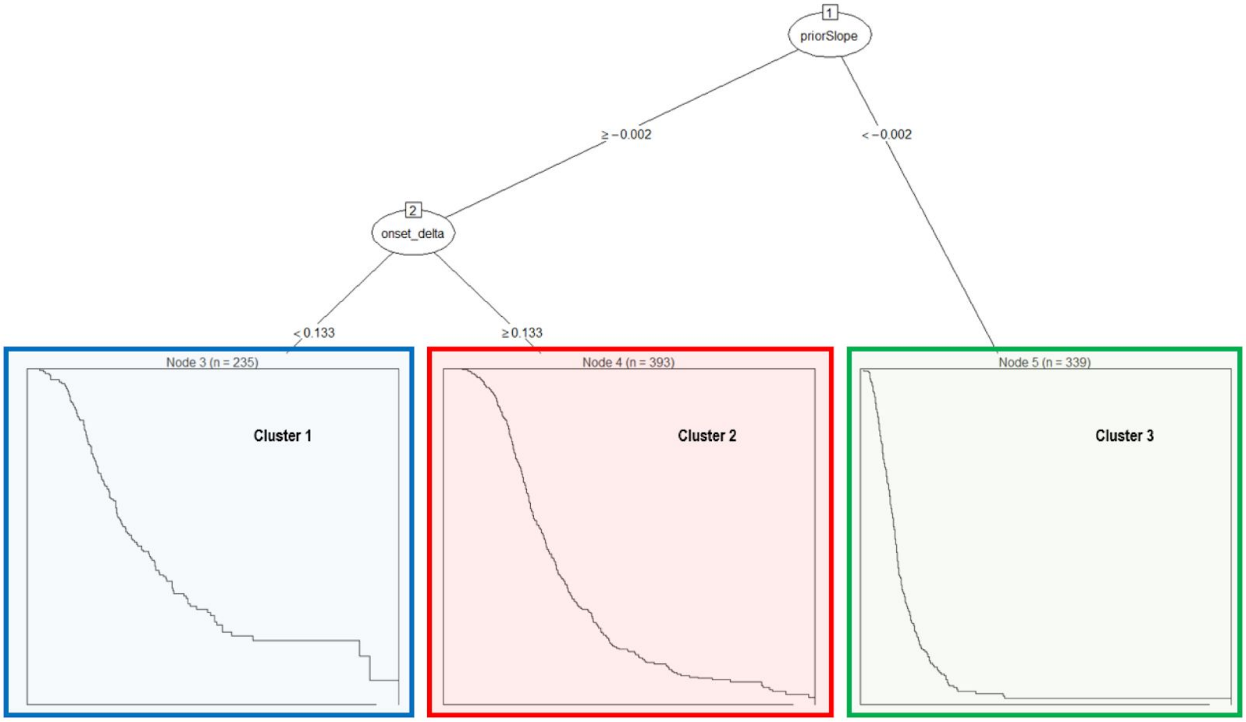

Figure 5: CART for National Registries Survival Probabilities Prediction (Subchallenge 4)

Model Training

To get a baseline performance of the winning model of the 2012 challenge, we used a similar initial prediction model that did not have any clusters to address the question in Subchallenge 1. Granted, this was out of the scope of the objective of this challenge to predict patient subgroups, but it allowed us to obtain an estimate of how well the prediction worked without subgroups. This initial slope progression prediction model turned out to perform the worst out of the four of our successful submissions to Subchallenge 1. The submission ranked 59 at the end of the leaderboard round with a c-Index, Pearson Correlation, RMSD equal to 0.5173, -0.07849, 35.4234, respectively. As the challenge progressed and our team began to investigate the different clustering techniques mentioned above, we aimed to improve the performance of our prediction models for each of the clusters once initial models were structured. This was accomplished through a step-wise procedure for all of our models in all Subchallenges. The scheme for our model optimization is located below (**Figure 6**).

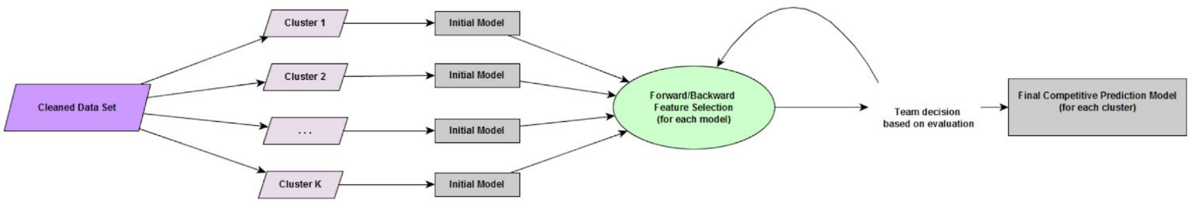

Figure 6: Scheme for Optimization of Model Performance

In the step-wise procedure, performance of a previous model for a given cluster was evaluated after either performing an inclusion or exclusion step. The team then decided whether to modify or keep the previous model based on the result of each step. Once no significant performance gain could be observed anymore the step-wise procedure was terminated.
The inclusion step individually added each feature from the feature set and/or interactions with features already in the previous model and ranked the new model candidates according to their performance in 5000fold crossvalidation. The exclusion step individually excluded each term currently present in the model and estimated performance based on 5000fold crossvalidation for each of the new, trimmed models. Our optimization criterion for Subchallenges 1 & 3 was the minimization of RMSE; while the criteria for Subchallenges 2&4 were the maximization of iAUC and c-Index.

Final Competitive Models

Below is a summary for the prediction model features that correspond to their respective clusters and Subchallenges. These models are those that resulted from the step-wise procedure.
Subchallenge 1: PRO-ACT Slope Prediction

Cluster 1: Q9 Climbing Stairs, Treatment Group, Phosphorus

Cluster 2: Gender, Creatinine, Alkaline Phosphatase, **White** Blood **Cell**, Creatinine x **White** Blood **Cell**^a^, Potassium, **White** Blood **Cell** x Potassium

Cluster 3: ALSFRS **Total**^b^, CK, ALSFRS **Total** x CK, FVC **Normal**

Cluster 4: Onset Delta, Blood Pressure Systolic, Q10 Respiratory, Alkaline Phosphatase

Cluster 5: Onset Delta, Q5 Cutting, FVC **Normal**

Cluster 6: Month, Trunk, FVC, Mouth x Trunk

Cluster 7: Trunk, Race, Blood Pressure Diasoltic, **If** Use Riluzole

Subchallenge 2: PRO-ACT Survival Probability Prediction

Cluster 1: FVC, Prior Slope^c^ Onset Age^d^, FVC **Normal**, Chloride

Cluster 2: trunk, Prior Slope, Onset Age, FVC Percent, Race, Trunk x Race

Cluster 3: FVC, Prior Slope, Onset Age, FVC Percent, Gender

Cluster 4: FVC, Prior Slope, Onset Age, Hemoglobin, Chloride

Subchallenge 3: National Registry Slope Prediction

Cluster 1: ALS Staging Com, ALSFRS **Total**, Q1Speech, Q9 Climbing Stairs, Hands

Cluster 2: Q1 Speech, Gender, Q4 Handwriting, MEDHx Diabetes, ALS Staging **Total**, ALS Staging Move

Cluster 3: Onset Site, ALS Staging Eat, Prior Slope, Q6 Dressing and Hygiene, Trunk, Gender

Subchallenge 4: National Registry Survival Probability Prediction

Cluster 1: Prior Slope, Onset Age, ALS Staging **Total**, Prior Slope x ALS Staging **Total**, Age, Respiratory

Cluster 2: Prior Slope, Onset Age, Q1 Speech, Gender

Cluster 3: Prior Slope, Onset Age, hands, MEDHx Thyroid

a Features that are strung together by “x” indicate an interaction between the terms in the model
b ALSFRS Total has been calculated as the minimum value for a patient from 0-3 months
c Prior Slope is calculated as the ALSFRS slope from onset delta to 365/3 days
d Onset Age reflects the calculation of the age of onset of disease

Submission Format

Submissions to the progression subchallenges included the ALSFRS_slope values obtained when predicting with our linear models.
Submissions to the survival subchallenges included probabilities of survival for the given time points. This can also be inferred from the data, since pobabilities naturally decrease for later timepoints.

Discussion

Throughout the duration of the challenge, our team had four successfully scored submissions for the leaderboard. Each of the submissions contained different clustering techniques; as well as, variations of predictor variables in the models. The four submissions are summarized in **Table 1 & 2**. Our team’s first submission with no clusters performed the worst as already mentioned. This was an expected result as we already knew that subgroups of patients are likely to exist in patients with ALS. The submission that performed the best was that which used PCA as the clustering technique with 2 clusters (**Table 2**).

Though this was the best leaderboard submission, we also wanted to investigate other clustering techniques; such as, RPMM. In using RPMM, our team examined different numbers of cluster with models to see how performance changed. The data was found to be very heterogeneous which led to a wide variety of possibilities to subset patients into different clusters. Thus, there was little agreement between the different clustering techniques on how to subset the data. We suspected that one of the reasons for high heterogeneity were artifacts in the data that were acquired during data collection, since some of the features had small subsets of outliers that differed from the majority of patients by several magnitudes.
Since unsupervised clustering methods did not seem to be very beneficial for prediction, CART-based clustering was employed for the final submission. Since clustering is performed based on differences in the outcome, the resulting clusters seemed more meaningful for prediction. This became especially apparent, when in survival analysis CART automatically separated patients into high, medium and low risk groups.

entityId	Team	Date/Time	Clustering Technique	Number of Clusters	Predictor Variables by cluster
syn4921939	JayHawks - ALS	08/31/2015 11:00:13PM	Principle Components Analysis	2	"Cluster 1 - onset_delta, Creatinine, pulse, Bilirubin (Total) Cluster 2 - onset_delta, trunk, leg, Phosphorus, pulse"
syn4939744	JayHawks - ALS	09/14/2015 07:20:09PM	RPMM	8	"Cluster 1 - priorSlope, onset_delta, ALSFRS_Total, priorSlope x ALSFRS_Total, treatment_grou.Placebo, Q4_Handwriting; Cluster 2 - onset_delta, Creatinine, Q1_Speech, Bicarbonate, onset_delta x Bicarbonate; Cluster 3 - priorSlope, onset_delta, ALSFRS_Total, priorSlope x ALSFRS_Total, treatment_group.Placebo, mouth, onset_delta x mouth; Cluster 4 - priorSlope, Bicarbonate, pulse; Cluster 5 - priorSlope, Chloride, Q9_Climbing_Stairs, onset_delta; Cluster 6 - priorSlope, leg, pulse, bp_systolic; Cluster 7 - priorSlope + Phsphorus, CK, Alkaline.Phosphatase, CK x Alkaline.Phosphatase; Cluster 8 - onset_delta, hands"
syn4931489	JayHawks - ALS	09/07/2015 02:39:00PM	RPMM	2	"Cluster 1 - Bicarbonate, Platelets, fcv, onset_delta, trunk, onset_site.Limb; Cluster 2 - fcv_percent, onset_delta, trunk,

					onset_site.Limb, if_use_Riluzole.Yes"
syn4904995	JayHawks - ALS	08/24/2015 10:28:01PM	No Clusters (All Patients Assigned to the same cluster)	1	"onset_site, onset_delta, gender, ALSFRS_Total, FVC, Creatinine"

Table 1: Summary of Clustering Approaches Used for Submissions

entityId	Team	Date/Time	Concordance Index	Pearson Corr.	RMSD	Rank
syn4921939	JayHawks - ALS	08/31/2015 11:00:13PM	0.606510015	0.3094672	0.577323165	17
syn4939744	JayHawks - ALS	09/14/2015 07:20:09PM	0.597329224	0.26753028	0.597039109	27
syn4931489	JayHawks - ALS	09/07/2015 02:39:00PM	0.57261171	0.19186138	0.601966685	33
syn4904995	JayHawks - ALS	08/24/2015 10:28:01PM	0.517270159	-0.07849761	35.423399	59

Table 2: Final Scoring From Leaderboard for Subchallenge 1

In regards to our CART-based clustering, we see that "onset_delta", plays a large role in the separation of subgroups of patients - "priorSlope" and "onsetAge" also utilize "onset_delta" in their calculation. It can also be observed that the slope progression prediction features are very different from cluster to cluster, whereas for the survival probability predictions, most clusters share similar features with only minor in the model. We believe that this speaks to the difficulty in accurately predicting a patient's ALSFRS slope for 3-12 months. Slope of subgroups may be driven by different features, which are either not present in the dataset or which we potentially removed from our analysis due to high missingness.

Authors Statement

Rama Raghavan - Managed scripts to be submitted to the cloud, handled any errors that occurred while reading in testing data, and validated final code.
Richard Meier - Led organization of overall code, implemented code for inclusion/exclusion of variables, and assisted in clustering efforts.
Janelle Noel - Managed data cleaning, worked on prediction models and clustering algorithms, and final write-up.
Junqiang Dai - Helped with overall data cleaning and decision making of variables to include in our usable data set.
Stefan Graw - Assisted with data cleaning

Alex Karanevich - Assisted with data cleaning and investigation of data attributes, specifically in regards to how slope was calculated.
Joseph Usset, Devin Koestler, and Brooke Fridley advised group as to which methods to try and how to proceed through submission rounds.
All authors have collectively collaborated throughout this challenge process.

Acknowledgements

Taking part in this DREAM ALS Stratification Prize4Life Challenge was made possible by the Department of Biostatistics at The University of Kansas Medical Center (KUMC), The University of Kansas Cancer Center (KUCC), and the Kansas IDeA Network of Biomedical Research Excellence (K-INBRE).

ALS Stratification Challenge, team Als_UoB

Wojciech Lesiński1), Aneta Polewko-Klim1), Agnieszka Golińska1), Krzysztof Mnich2), Witold Rudnicki12)
1)Department of Bioinformatics,
2)Computational Centre
University of Białystok, Ciołkowskiego 1M, Białystok, Poland

General methods

The modelling is split in three steps - feature construction, feature selection and finally model building and selection. In the first step we construct features describing the time series data. To this end we construct a wide and redundant set of descriptors for each variable measured in multiple time points, mostly following the approach proposed by the winning team of the ALS DREAM 7 Challenge [1].

Then we apply the feature selection step. It is based on the information entropy, we compute the information gained for the decision variable due to knowledge of each single variable and each possible pair of variables. Then we remove redundant features, and finally we perform final verification of feature importance by comparing the importance of the selected features with the importance of the non-informative ones.

The final model is build using random forest classifier [2], taking into account limitation of the challenge, namely that the final model can be built only using six original features. We build several hundreds of models for each question, and test them using cross-validation. The models, that achieve best scores in cross-validation are selected and are used for prediction of unseen data. For question 1, information from both cross-validation procedure and from validation on the leaderboard set is used for selection of the best models. The more detailed description of each step and the application for questions 1-4 is given below.

Feature construction

For each original variable that was measured in several time points we construct derivative variables that describe both time-dependent and time independent descriptors, mostly following the descriptors proposed by winning team in the DREAM 7 ALS Challenge [1]. These descriptors are:

- maximal value
- minimal value
- mean value
- median of the squared values
- standard deviation
- sum of all elements
- first value

- last value
- slope (defined as difference between last and first element divided by the time difference)
- slope2 (obtained from linear fit to data)

The number of derivative descriptors is larger than the number of data points in the series. We decided to construct so many derivative for two reasons. Firstly, one doesn't know in advance which derivative variable may be relevant. The mean value and slope are two most obvious choices, however, one cannot exclude that either minimal, or maximal value could be the most important variable. Moreover, while we certainly create a set of highly correlated, and hence redundant, variables, the feature selection process is designed to remove non-informative and redundant variables.

Feature selection

Feature selection is based on a three-step procedure.

Step 1

In this step variables that can help in increasing our knowledge of the decision variable are identified. To this end we perform exhaustive search of variables and pairs of variables that carry statistically significant information on the decision variable. At this stage we don't apply very strict statistical criteria, in particular the Bonferoni correction for multiple tests is not performed. We don't apply the Bonferoni correction, because we have previuosly artificially expanded the number of variables, by creating numerous highly correlated variables. Therefore the number of independent variables, and hence independent tests is artificially inflated. Moreover, this number is not known precisely, since the correlation structure of the redundnant variables is not
measured. The problem of finding the statistical significance of the results is dealt with in the third step, using control of the false discovery level.
In the single dimensional case we use information gain,
defined as:

$$IG(V_i)=H(D)-H(D|V_i)$$

where

$$H(D)=\sum_k p(D_k)\log_2(p(D_k))$$

and

$$H(D|V_i)=\sum_{k,l} p(D_k \wedge V_{il})\log_2(p(D_k \wedge V_{il}))$$

These formulas require discrete decision variable, hence we need to discretize the ALSFRS_Slope for questions 1 and 3. To this end we define that ALSFRS_Slope is *high*, when it is higher than median, and *low*otherwise.
In two dimensional search we use two measures. Firstly we identify all pairs of variables, for which the total information gain defined as:

$$IG_{Tot}(V_i,V_j)=H(D)-H(D|V_i,V_j)$$

where

$$H(D|V_i,V_j)=\sum_{k,l,m} p(D_k \wedge V_{il} \wedge V_{jm})\log_2(p(D_k \wedge V_{il} \wedge V_{jm}))$$

is statistically significant. However, this is too loose a filter, since a random variable in pair with strongly informative variable may create a pair that meets the criteria. To weed out these non-informative

variables we apply second criterion, namely information gain on the decision variable, due to adding knowledge on the second variable, to the one already informative variable. We call this measure information gain from best, since it is defined as the difference between total information gain due to knowledge of two variables, and information gain due to the *more informative* variable of the pair under scrutiny:

$IG_{FB}=IG_{Tot}(V_i,V_j)-max(IG(V_i),IG(V_j))$

To estimate the statistical significance of the results, we take advantage that the information gain conforms to the Chi-squared distribution.

It is interesting to note, that the most informative variable by far was *onset_delta* - the variable that is not related in any way to the ALS mechanism.

However, it is quite simple to explain after observing that patients with very high negative values of onset delta, were usually the ones with low ALSFRS_slope - there were very few patients with high negative onset_delta and steep slope. This happens because only the patients with slow progression of the disease were able to survive long enough from the onset to the beginning of the study.

Step 2.

The result of the first step are two lists: a list of all variables and a list of all pairs of variables that carry the statistically significant information about the information variable. The procedure used for generation of the variables and loose criteria for the filtering informative variables and pairs of variables, results in a large redundant list of variables.

In the second step we apply heuristic greedy reduction of both lists. To this end we first start with the pair with highest $IG_{Tot}(V_i,V_j)$. The we remove all pairs of variables that don't add information to the best pair. To this end we compute the following difference:

$IG_{Tot}(V_i,V_j;V_k,V_l)-IG_{Tot}(V_i,V_j)$.

The pairs of variables that don't contribute additional information are removed from the list. The procedure is then repeated for the second best pair, third best pair and so on, until all redundant pairs are removed from the list. Then the analogous procedure is performed for the list of single informative variables. This procedure results in a list of most informative, non-redundant variables.

Step 3.

The list of non-redundant variables is finally verified by checking whether their informativeness could arise due to random fluctuations. To this end we generate 20 randomized copies of each variable on the final list, and compute information gain for variables and pairs of variables. The highest information gain achieved by the randomized copy of any variable is used as a threshold for icluding the variables into the final list.

Similarly only those pairs of variables, that have information gain higher than that achieved by any pair that contains randomized copy, are included in the final list of pairs.

The final list of variables contains all variables that are either on the list of single informative variables, or on the list of informative pairs of variables.

Model building

The non-redundant set of informative variables was used in the construction of the final model. In the first step, the missing values for each variable
were replaced by it's median. Variables were clustered into groups derived from the same original variables.
For example set of informative variables obtained for the first question contained four variables derived from the original variable ALSFRS_Total, namely minimum (ALSFRS_Total_min), standard deviation (ALSFRS_Total_sd), first element (ALSFRS_Total_first_el) and slope (ALSFRS_Total_slope).
Then all possible combinations of six groups were generated. For each combination model was constructed using random forest algorithm [2].
Performance of the model was measured using cross validation and, in the case of question 1, using leaderboard set. The performance of each
model was estimated using only the records for which data for all variables was available. The best model with high coverage was selected
as a primary model, then the best model that could be used for patients not covered by the first model, was selected as secondary model.

Clustering

Clustering was performed solely using information on the availability of data.
Initially we have tried to spliting patients into two clusters, one similar to long-living patienst with very large values of onset delta, the other similar to patients with low onset delta and fast progress of disease.
To this end, in question 1 we selected patients with *onset_delta* longer than 1000 days, as positive examples, and patients with onset delta shorter than 500 days and *ALSFRS_slope* lower than median (low *ALSFRS_slope* corresponds to quick progress of the disease).
The random forest was traind to discriminate between members of these classes, the accuracy of the model was roughly 35%.
To verify quality of clustering we have used the predictions as a new variable, that replaced the original *onset_delta* variable for prediction of the ALSFRS_slope. Unfortunately adding this variable did not improve to predictions. of the *ALSFRS_slope* variable.
On the other hand - any clustering of objects that could be used for improving predictions would certainly be utilised by random forest algorithm, hence it is unlikely that clustering objects before runnign models could improve results by much when random forest is used to build regression models.

Model for Question 1.

Eleven groups of variables were identified as relevant for decision:
*onset_delta

*ALSFRS_Total {ALSFRS_Total_min, ALSFRS_Total_sd, ALSFRS_Total_first_el, ALSFRS_Total_slope}
*Creatinine {Creatinine_sqmedian_v,Creatinine_last_el_v}
*fvc {fvc_min_v, fvc_slope}
*hands {hands_sqmedian, hands_slope}
*Q1_Speech {Q1_Speech_sd, Q1_Speech_sqmedian, Q1_Speech_slope, Q1_Speech_slope2}
*Q3_Swallowing {Q3_Swallowing_sqmedian, Q3_Swallowing_first_el, Q3_Swallowing_last_el, Q3_Swallowing_slope}
*Q4_Handwriting {Q4_Handwriting_max_v, Q4_Handwriting_min_v}
*Q5_Cutting {Q5_Cutting_min, Q5_Cutting_last_el, Q5_Cutting_slope}
*Q6_Dressing {Q6_Dressing_mean, Q6_Dressing_last_el, Q6_Dressing_slope}
*Q9_Climbing_Stairs: {Q9_Climbing_Stairs_sqmedian}
From these 6 groups we created all 84 combinations and selected two models:
Model.1: {onset_delta, hands, Q1_Speech, Q9_Climbing_Stairs, **fvc**, Q5_Cutting}
Model.2: {onset_delta, hands, Q1_Speech, Q9_Climbing_Stairs, **ALSFRS_Total**, Q3_Swallowing}
The first model, that gives one of the best results in cross-validation and gave best results for the leaderboard set uses derivatives of the *fvc* variable that are available only for roughly 60% of the data in the leaderboard set. Therefore the second model, that also gives very good results on cross-validation and best results for the leaderboard set, among the models that don't use *fvc* variable was selected for predictions when information on *fvc* is not available.
It is interesting to note, that the all best models contain *onset_delta* variable. The best models developed for Question 1, and containing *onset_delta* were able to explain nearly 20% of variance in cross-validation, whereas the best models constructed without this variable could explain at most 10% of variance in data.

Model for Question 2.

Eight groups of variables were identified as relevant for decision:
*onset_delta: {onset_delta, onset_site}
*ALSFRS_Total: {ALSFRS_Total_last_el}
*Chloride: {Chloride_mean_v, Chloride_sum_el}
*Creatinine: {Creatinine_min, Creatinine_sum_el}
*fvc: {fvc_sum_el, fvc_sqmedian, fvc_mean, fvc_last_el}
*fvc_percent: {fvc_percent_sqmedian, fvc_percent_sum_el, fvc_percent_last_el, fvc_percent_slope}
*weight: {weight_last_el, weight_slope2, weight_slope}
*Gender
Model.1: {onset_delta, ALSFRS_Total, Creatinine, weight, fvc_percent, fvc}
Model.2: {onset_delta, ALSFRS_Total, Creatinine, names.weight names.Gender, names.Chloride}
For model in this Question eight basic groups of variables have been selected. Models are based on random forest (built for each possible combination of six groups of variables selected from eight basic groups of variables). We chose two models: first one with the best results in cross-validation and second one which covers objects from training set, not covered by first model. A key decision for classification to cluster was presence of fvc and fvc_percent.
In this subchallenge we have determined probability of death.

Model for Question 3.

Six groups of variables were identified as relevant for decision:
*trunk: {trunk_slope2, trunk_first_el_v}
*Q3_Swallowing: {Q3_Swallowing_max_v, Q3_Swallowing_max_v}
*Q4_Handwriting: {Q4_Handwriting_min_v}
*Q8_Walking: {Q8_Walking_first_el_v}
*hands: {hands_sd_v, hands_min_v}
*ALSFRS_R: {ALSFRS_R_Total_min_v}
Model: {trunk, Q4_Handwriting, Q3_Swallowing, Q8_Walking, hands, ALSFRS_R}
The selection procedure described above selected six basic groups of variables as essential. As a result we were able to build a single model with all the features. Missing data have been replaced by medians. Unfortunately, the quality of model measured by the correlation and RMSD was weaker than in the case of subchallenge 1.

Model for Question 4.

Six groups of variables were identified as relevant for decision:
*ALSFRS_R_Total: {ALSFRS_R_Total_sqmedian_v, ALSFRS_R_Total_sum_el_v}
*ALSFRS_Total: {ALSFRS_Total_sqmedian_v, ALSFRS_Total_last_el_v, ALSFRS_Total_sd_v, ALSFRS_Total_sum_el_v}
*hands: {hands_max_v, hands_min_v}
*MEDHx_Thyroid,
*Q6_Dressing_and_Hygiene: {Q6_Dressing_and_Hygiene_last_el_v}
*R3_Respiratory_Insufficiency: {R3_Respiratory_Insufficiency_sum_el_v}
Model: {ALSFRS_R_Total, ALSFRS_Total, hands, MEDHx_Thyroid, Q6_Dressing_and_Hygiene, R3_Respiratory_Insufficiency}
Feature selection was performed separately for death cases in the 12, 18 and 24 months. Then we selected groups of features identified as important in at least two cases. We chose six such groups, which was enough to build a single model (as in the case of subchallenege 3).
In this subchallenge we have determined the probability of death.

References

[1] Küffner R. et al. (2015) Crowdsourced analysis of clinical trial data to predict amyotrophic lateral sclerosis progression, *Nature Biotechnology* **33(1)**, 51-57
[2] Breiman, L. (2001) Random Forest, *Machine Learning*, **45**, 5-32,

ZhuLab: Solution for ALS Stratification Prize4Life Challenge

Xiaotao Liang[1], Shanfeng Zhu[1]

1. School of Computer Science, Fudan University, Shanghai, China

- We are willing to make our submission public.
- For sub-challenge 2 and 4 we predicted the probability of survival.

Introduction

For each sub-challenge, we had no clustering, selected 6 features, and utilized the random forest model[1] to predict ALSFRS slope or survival.

Methods

Data pre-processing

The two files `data/analyze.py` and `data/generate.py` were used to process the data.

- Encode the features whose values were text as integers.
- Perform data normalization for some features.
- If the number of missing values of a feature was not larger that a threshold, any subjects that had missing values of this feature were removed.
- Convert the time-resolved features into static features, the minimum and maximum of the values.[2]

Feature selection

For each sub-challenge, we first selected some features which have a small number of missing values or doesn't have any missing values by hand.

In sub-challenge 1, 2, we merged the spike data set and the leaderboard data set into a validation set. For different combination of 6 features from the features selected above, we trained the random forest model on the PRO-ACT data set, tested its performance on the validation set for three times, and found out the best combination of features.
In sub-challenge 3, 4, we selected features in the same way as sub-challenge 1, 2, except that 3-fold cross validation was taken on the registry data set when testing.
It should be noted that we ran a random model to calculate the values of statistics for calculating the Z-scores and aggregated the three Z-scores into one score in order to be able to compare the performances between different combination of features or different models.
In the final model, the features selected were:

- Sub-challenge 1: `ALSFRS_Total, onset_delta, mouth, Q1_Speech, Q7_Turning_in_Bed, Q9_Climbing_Stairs.`
- Sub-challenge 2: `ALSFRS_Total, onset_delta, Age, fvc_percent, treatment_group, if_use_Riluzole.`
- Sub-challenge 3: `ALSFRS_R_Total, onset_delta, onset_site, Q6_Dressing_and_Hygiene, Q7_Turning_in_Bed, Q8_Walking.`
- Sub-challenge 4: `ALSFRS_Total, onset_delta, onset_site, Age, Mutation, ALS_Staging_Total.`

Data imputation

We imputed missing data by the build-in function of the randomForestSRC package[1].

Running the final model

1. Put the original data files into the directory `data`.

```
all_forms_PROACT.txt
all_forms_validate_spike.txt
all_forms_validate_leader_full.txt
all_forms_registry_training.txt
surv_response_PROACT.txt
surv_response_validate_spike.txt
surv_response_validate_leader.txt
surv_response_registry_training.txt
```

1. Change into the directory `data` and then run `prepare_dataset.sh` to calculate the ALSFRS slope and merge the data set.
2. Write the feature names of the 6 selected features into the file `data/feature_list.txt`.
3. Train the model depending on which sub-challenge you are working on.

- sub-challenge 1: `bash submit.sh slope rfsrc none`
- sub-challenge 2: `bash submit.sh surv rfsrc none`
- sub-challenge 3: `bash run_registry.sh slope rfsrc`
- sub-challenge 4: `bash run_registry.sh surv rfsrc`

1. Modify the value of the variable `target` in `predictor.sh` as `slope` or `surv`, depending on what will be predicted.
2. Prediction

```
bash select.py input_file_path output_file_path
bash predictor.py input_file_path output_file_path
```

Conclusion/Discussion

There are two goals for this challenge. One is to detect patient specific subgroups, and the other is to find out the most predictive features. For the first goal, it is hard to tell whether a model leads to data leakage. Actually, in my view, as long as the cluster number was utilized, data leakage could not be avoided. In order to avoid data leakage, we tried to cluster patients only using the six selected features, but we didn't find any specific subgroups. So we didn't cluster patients in our final model. For the second goal, because of the constraint that only a limited number of 6 features were allowed for the prediction, we discarded using existing feature selection algorithms and turned to enumeration. We enumerated different combination of features and tried to find out the best one. In this way, we focused on the combination of features while not individual features.

References

1. Ishwaran H. and Kogalur U.B. (2015). Random Forests for Survival, Regression and Classification (RF-SRC), R package version 1.6.1. http://cran.r-project.org/web/packages/randomForestSRC/index.html
2. Robert Küffner. et al. Crowdsourced analysis of clinical trial data to predict amyotrophic lateral sclerosis progression. Nature Biotechnology 33, 51–57 (2015).

Authors Statement

X.L. implemented the model and wrote the Wiki. X.L. and S.Z. worked together for developing the model.

Team chk: A Model for Predicting ALSFRS Slope and Survival of ALS Patients

Christoph Kurz
Institute of Health Economics and Health Care Management
Helmholtz Zentrum München, Germany

Abstract

Our model utilizes a combination of boosted linear models, support vector machines and random forest for predicting the progression of the ALSFRS score. A random forest approach is used for the prediction of the survival of ALS patients.

Introduction

The challenge was split into four subchallenges (sub). Two challenges concerned the prediction of the ALSFRS slope (sub1 and sub3), the other two asked for the prediction of survival probabilities (sub2 and sub sub4).

Methods

For each subchallenge we chose six variables. We first generated a data set containing all variables and then performed three variable selection methods to find the six best variables.
These three methods included a lasso glm, stepwise AIC linear variable selection and random forest feature importance analysis. Some features have high missingness so we could not use them. These are the variables we finally came up with for each challenge:

- ProactProgression: onset_delta, weight, Hemoglobin, fvc, fvc_percent, ALSFRS_Total
- ProactSurvival: onset_site, onset_delta, fvc_percent, fvc, Age, ALSFRS_Total

- NatRegProgression: hands, leg, mouth, respiratory, onset_delta, ALSFRS_R_Total
- NatRegSurvival: onset_site, onset_delta, mouth, respiratory, Age, ALSFRS_R_Total

If the feature is longitudinal (e.g. ALSFRS_Total) we engineered the following variables: standard deviation, mean, max, min, difference between min and max, slope, length, minmax slope, first value, last value.
Many of these new variables are highly correlated so we removed those with rho > 0.95.
These are the methods we used in each subchallenge:

- ProactProgression: ensemble of boosted linear model and support vector machine (svm)
- ProactSurvival: random forest
- NatRegProgression: ensemble of boosted linear model, support vector machine (svm) and random forest
- NatRegSurvival: random forest

we found that the patient structure of the Proact data is very different from the leaderboard and validation data. We therefore performed propensity score matching to find the most
similar patients. This lead to a great reduction of training data (maybe too great).
missing values were imputed using the R package *mice*.
The selector was only used to pass the variables, no clustering etc. has been used.

Insights gained

- Patient structure of Proact and leaderboard is very different.
- The main challenge was the huge amount of missing data. Often about 50% of one variable is missing. Overall results are very unstable.
- I had some difficulties building a consistent model for the predictor script because you have to address so many cases with different data quality.
- The server we had to use only provides limited computational power.

I am willing to make my submission public. I predicted probability of survival.

The ICOS submission to the DREAM ALS stratification challenge: RGIFE+RandomForest+k-means/hierarchical clustering

Nicola Lazzarini, Jaume Bacardit
The Interdisciplinary Computing and Complex BioSystems (ICOS) research group, Newcastle University, UK

- Will you be able to make your submission public as part of the challenge archive? Yes

Background/Intro

The core predictor for all sub-challenges are Random Forests (either classification or regression ones). The ICOS method, applied to all four subchallenges, is composed of the following steps:

1. Division of the training data into training and validation sets.
2. Application of our RGIFE iterative feature elimination method [1] to the training dataset to filter down as much as possible the number of features.
3. We cluster the training set using only the filtered features using either k-means or hierachical clustering (using the Ward method) and with a number of clusters ranging from 2 to 8.
4. The training set is divided into clusters. RGIFE is applied separately to each cluster to get the number of variables per cluster down to 6 or less
5. Using the validation set we select the clustering algorithm & number of clusters used for the submitted predictor

For subchallenges 2 and 4 our models are generating probabilities of *survival*.
Random Forest were chosen because they are very robust overall, and also because they provide information (Gini index) that is used to guide the feature reduction process.
By clustering on the data reduced in step 2, the clusters should be more representative for each of the four sub-challenges than when generated from the whole feature set

Methods

The RGIFE feature reduction method

RGIFE [1] is an iterative feature elimination method in the lines of SVMRFE [2]. That is, it uses a machine learning method to rank features and then eliminates the features at the bottom of the ranking. In the current RGIFE implementation the machine learning method is the random forest algorithm, and features are ranked using the Gini index. The main difference between RGIFE and SVMRFE is that our method incorporates a step of backtracking: When we attempt to remove a block of features, we evaluate the quality of the resulting model (using multiple iterations of 10-fold cross-validation), and if the quality is degraded we put back the features and attempt to remove a different block of features, next the ranking. Initially the size of the block of features to be removed is 1/4 of the total number of features. If there are 5 consecutive unsuccessful trials, because the quality of the resulting model is degraded, we check if there is a “soft fail”. That is, a model with a degradation in relation to the best model found so far that is below a certain threshold. If so, we accept this model. If there is no soft fail, the size of the block is divided by four. The iterative reduction process stops when the block size is 1 and there are five unsuccessful trials.
For the slope sub-challenges, root mean squared error (rmse) was used as performance metric to evaluate a model. For the survival ones, the geometric mean (gmean) of sensitivity and specificity was used instead (to tackle the issue of class imbalance). RGIFE internally imputes missing values (using a mean imputation) and binarises discrete variables before growing the random forest.

Data cleaning and feature generation

1. Before starting, all variables from the “Concomitant Medication” and “Adverse Event” sections were removed. Also, all rows with a time point >90 were removed as well.
2. For the proact dataset a lot of cleaning was applied to the “Lab Test” variables, especially to harmonise several noticeable differences between variables in the training set, on one hand, and the spike and leaderboard sets, on the other hand. All cleaning is recorded in the clean.pl perl script.
3. In order to transform time series into a uniform representation we opted for generating variables for the first and last time points and the change between these, as suggested by one of the top methods in the first DREAM ALS challenge [3]. Variables without time points were used as is.
4. For each of the four sub-challenges, if more than 50% of values of a variable were missing, the variable was dropped.

Generation of training and validation sets.

In the final submission stage, for challenges 1 and 2 the training data was the union of the training+spike subsets, and the leaderboard data was used as validation set.
For sub-challenges 3 and 4, a randomly selected 10% of the instances was used as validation set, the rest for training. The predictability of the validation set was tested on the whole feature set in order to avoid a very biased validation set. The random splitting was repeated several times until a good validation set was found.

First RGIFE stage: Global feature reduction

First RGIFE is applied to find a single reduced set of features for all instances in the training set. We did a broad parameter sweep testing several maximum depths for Random Forest (from 2 to 10). Cost-sensitive classification was used for the survival datasets, in order to tackle the class imbalance problem. The samples of class *dead* were given a higher weight than the samples of the class *alive*. We tested weights from 2 to 5. As RGIFE is stochastic, 10 repetitions were run for each dataset and configuration. In the proact datasets 5 iterations of 10-fold cross-validation were used.
For the national registry datasets it was 10 (due to its smaller size). A max tree depth of 4 was consistently selected across all datasets. For 18 and 24-month survival a cost of 2 for 'dead' samples was selected, for 12-month survival the 'dead' cost was 3 (proact) or 5 (national registry).
From all the parameter sweep, we identified the RGIFE run with the best performance (either rmse or gmean) and used it as input for the clustering. If there were several runs with similar performance but varying size, we preferred smaller feature sets. For the survival challenges, we did run RGIFE separately to the 12-month, 18-month and 24-month versions of the dataset.

Clustering of patients

For each of the four sub-challenges (and each of the three survival intervals) we clustered the dataset using only the features left after RGIFE. All clustering was done in R, using two methods: kmeans and hclust (with the "ward.D" method). To get discrete clusters from the hierarchical clustering the cutree function of R was used. For each algorithm we generated clusterings with a
size ranging from 2 to 8. Before applying the clustering the data was imputed for missing values (again, using a mean imputation), discrete variables (if some were remaining) were binarised and the data was normalised using the R scale function (substracting the mean, dividing by the standard deviation).
Snip of R code. Variable data contains the training set matrix with the features remaining after the global RGIFE step.

```
data2=scale(data)
hc=hclust(dist(data2),method="ward.D")
for(i in 2:8) {
write.table(cutree(hc,k=i),paste("clusters-hclust-",i,".txt",sep=""))
}
for(i in 2:8) {
km=kmeans(data2,iter.max=100,nstart=20,centers=i);
write.table(km$cluster,paste("clusters-kmeans-",i,".txt",sep=""));
write.table(km$center,paste("centroids-kmeans-",i,".txt",sep=""))
}
```

Second RGIFE stage: finding a different set of variables for each cluster

The training data was now split into clusters, and RGIFE was applied separately to each cluster. We used the RGIFE parameters from the best run of the first stage.

For each sub-challenge we had 14 different clusterings (kmeans/hclust and size from 2 to 8). Also, for the survival datasets the reduced feature set+clustering generated from e.g. the 12-months version of the dataset was applied to the 18 and 24-months versions, and vice-versa, given that we had to find a single clustering that was good enough for the three versions

at the same time. For all datasets and clusterings we managed to generate RGIFE reductions with 6 or less features.

Selection of the final clustering using the validation set.

Finally, the clustering (either k-means or hclust, and number of clusters from 2 to 8) used in the submitted predictor was selected using the validation data, using the same metrics (rmse/gmean) as before. For the validation we used the random forest models generated for each cluster after the second RGIFE stage. Moreover, the missing value imputation was also specific to each cluster.

The selection of the final clusters for the survival challenges was more tricky, as the same clusters had to be used for the 12, 18 and 24-month survival prediction. In this case we selected the clustering with the best average performance across the three datasets, and making sure that the number of distinct features used across the three versions for the same cluster was <=6.

Generation of confidence intervals/probabilities for the submitted predictor

For the survival sub-challenges, the probability of surviving is taken directly from the Random Forest (percentage of trees voting for class 'alive'). For the progression challenges, the confidence score is generated following http://blog.datadive.net/prediction-intervals-for-random-forests/

Code

The code used for this DREAM challenge is an eclectic mix of R, python, shell, perl, sed and awk scripting. The core Random Forest is from the scikit-learn python package. RGIFE is coded in python, and the clustering was done in R. The other scripting languages were used for preprocessing and in "glue" code to connect all pieces. The random forest models were serialised using the cPickle python module.
We used whatever existing code we had that would allow us to quickly prototype the prediction models (hence some parts of the code may look ugly or counter-intuitive). There is no need to compile anything.

Clinical variables used for each challenge

Sub-challenge 1.

The best clustering was the hierarchical clustering and 4 clusters.
cluster1: ALSFRS Total, Q1 Speech, Q5 Cutting, onset delta, fvc, weight
cluster2: ALSFRS Total, Q1 Speech, Q5 Cutting, onset delta, fvc, weight
cluster3: ALSFRS Total, onset delta, fvc
cluster4: ALSFRS Total, Q5 Cutting, onset delta, weight

Sub-challenge 2.

The best clustering was the hierarchical clustering and 8 clusters.

- 12-month survival:

cluster 1: ALSFRS Total, onset delta, fvc percent, weight
cluster 2: Age, fvc, fvc percent, weight
cluster 3: fvc percent
cluster 4: Age, fvc
cluster 5: ALSFRS Total, fvc, fvc percent, weight
cluster 6: Age

cluster 7: Age, fvc, fvc percent, weight
cluster 8: Age, fvc, fvc percent, weight

- 18-month survival:

cluster 1: ALSFRS Total, onset delta, Age, fvc percent
cluster 2: onset delta, Age, fvc, weight
cluster 3: ALSFRS Total, Age, fvc, fvc percent
cluster 4: Age, fvc, fvc percent1, fvc percent
cluster 5: ALSFRS Total, Age, fvc percent, weight
cluster 6: Age, weight
cluster 7: weight
cluster 8: ALSFRS Total, Age, fvc, fvc percent, weight

- 24-month survival:

cluster 1: ALSFRS Total, onset delta, Age, fvc percent
cluster 2: onset delta, Age, fvc, fvc percent
cluster 3: ALSFRS Total, Age, fvc percent, fvc percent
cluster 4: ALSFRS Total, Age, fvc, weight
cluster 5: ALSFRS Total, Age, fvc percent, weight
cluster 6: Age, weight
cluster 7: onset delta, Age, fvc percent, weight
cluster 8: ALSFRS Total, Age, fvc, fvc percent, weight

Sub-challenge 3.

The best clustering was k-means with 4 clusters.
cluster 1: ALSFRS R Total, onset delta
cluster 2: ALSFRS R Total, ALSFRS Total, hands, onset delta
cluster 3: ALSFRS Total, hands
cluster 4: hands, onset delta, onset site

Sub-challenge 4.

The best clustering was k-means with 6 clusters.

- 12-month survival:

cluster 1:Age
cluster 2:ALSFRS R Total
cluster 3:ALSFRS Total, onset delta
cluster 4:ALSFRS R Total, ALSFRS Total, onset delta, Age
cluster 5:ALSFRS R Total
cluster 6:ALSFRS Total, Age

- 18-month survival:

cluster 1: ALSFRS R Total, Age
cluster 2: onset delta
cluster 3: ALSFRS Total, onset delta

cluster 4: Q1 Speech, onset delta
cluster 5: ALSFRS Total, onset delta, Age
cluster 6: ALSFRS R Total, ALSFRS Total, onset delta, Age

- 24-month survival:

cluster 1: onset delta, Age
cluster 2: ALSFRS R Total, onset delta, Age
cluster 3: ALSFRS R Total, ALSFRS Total, onset delta, Age
cluster 4: ALSFRS R Total, ALSFRS Total, onset delta, Age
cluster 5: ALSFRS Total, onset delta
cluster 6: ALSFRS R Total, onset delta, Age

Conclusion/Discussion

This challenge was been very useful to test our methods on different datasets, that stress them in ways that we had not originally anticipated (and identified some bugs). Something that with more time we would have liked to try is to add adverse events or concomitant medication. Moreover, for the proact dataset we noticed several variables that are present for almost all patients in the spike and leaderboard sets but not for the training set, for instance "ALSFRS R Total". Given that all_forms_training dominates the other sets in size, the variable was not picked up for the proact clusters, but did appear in all clusters of the national registry dataset. We wonder what would happen if we were to train the predictor using only the spike/leaderboard sets. Our intuition is that the more data, the better, but what if there is a real fracture between the subsets of the proact data?

References

[1] Swan, A.L. et al., A machine learning heuristic to identify biologically relevant and minimal biomarker panels from omics data. *BMC Genomics* 2015, **16(Suppl 1)**:S2, 2015
[2] Guyon, I., Weston, J., Barnhill, S. and Vapnik, V. Gene Selection for Cancer Classification using Support Vector Machines. *Mach. Learn*. **46**, 1-3 (March 2002), 389-422, 2002
[3] Küffner, R et al., Crowdsourced analysis of clinical trial data to predict amyotrophic lateral sclerosis progression, *Nature Biotechnology***33**, 51-57 (2015)

Authors Statement

Nicola Lazzarini coded the RGIFE method at the core of our predictor, Jaume Bacardit did all the data analysis and created the code for selector and predictor.

Acknowledgements

We are grateful to the School of Computing Science at Newcastle University for the HPC cluster it has provided to the ICOS team, where all the training process of the predictor too place.

Team Donuts ALS Stratification Challenge

Yash Kiran Deshpande, Kenneth Jung, Lester Mackey, Ji Park, Kris Sankaran, Vatsal Sharan
Stanford University
Our submission will be made public, and we have predicted risk of death.

Introduction

Our approach to this challenge builds on the experience of our team member, Lester Mackey, with the previous ALS challenge. Specifically, we have largely used the same approach to processing patient data into feature vectors. Exploratory analysis showed that one of the key challenges to address was the fact that the training and validation data for the ProAct dataset are from distinct clinical trials. This was concerning to us because it seemed likely that this would result in the patients in the training and test sets being quite different from each other. Indeed, they differ dramatically with respect to one year survival rates, in addition to which features were recorded. Furthermore, it was possible to train a classifier to very reliably distinguish samples arising from the training or validation sets, indicating definite covariate shift at the very least. Thus, we spent considerable effort attempting to address this problem, finally settling on a method similar to importance sampling in which we weight samples according to the ratio of the probabilities that they arose from the target distribution (e.g., the validation dataset) versus the training distribution while training our models. We also spent some effort trying to perform domain adaptation or transfer learning from the ProAct data to the national registry data. However, these datasets were so dissimilar that our best cross validation performance in the registry data was from disregarding the ProAct data altogether. Our final models were all gradient boosted models using squared error for the progression tasks and cox proportional hazards loss for the survival tasks. We have provided all source code in a zipped tarball under this project's files.
Note that we elected to forego clustering, instead using the same set of six features for all samples in each challenge.

Methods

Featurization

We followed the methods from one of the winning submissions by our team member Lester Mackey to the previous challenge. From the raw data associated with a given subject, we extract a vector of numeric features to be used for model training or prediction. We apply different extraction protocols for time series data and for data without a temporal element (termed “static data” below):
· Static Data: Our static data features are derived from subject ALS history, family history, demographics, and treatment group.
o We include the numeric fields of “Onset Delta” and “Age” directly as features.
o We incorporate the following fields as binary features by assigning a value of 1 if the attribute is associated with the subject and a value of 0 otherwise: "Race- Asian", "Race - Black/African American", "Race - Caucasian", "Race - Other", "Aunt", "Aunt (Maternal)", "Cousin", "Father", "Grandfather (Maternal)", "Grandmother", "Grandmother (Maternal)", "Mother", "Uncle", "Uncle (Maternal)", "Uncle (Paternal)", "Son", "Daughter", "Sister", "Brother".
o For each distinct value of each of the following categorical fields, we add a binary feature to our dataset indicating whether a given subject is associated with that value of that field: “Sex”, "Symptom", "Site of Onset", "Neurological Disease", "Study Arm".
· Time Series Data: We additionally extract numeric features from the following fields which contain repeated measurements and associated delta values:
o ALSFRS total score and question scores
o FVC subject liters
o SVC subject liters
o Vital signs data: weight, height, respiratory rate, systolic blood pressure, and diastolic blood pressure.
We ignore records missing either a delta value or a measurement value. In addition, when two records have the same delta value, we keep only the first record.
For each time series field, we also calculate a derivative time series of pairwise slopes. That is, for each temporally consecutive pair of measurement values, we compute (second measurement value - first measurement value)/(second delta value - first delta value) and associate this pairwise slope measurement with the delta value (second delta value + first delta value)/2.
For each original time series and each derivative time series we include the following statistics as subject features:
· The maximum measurement value
· The minimum measurement value
· The last measurement value
· The mean measurement value
· The number of measurement values
· The sum of all measurement values
· The delta value associated with the first measurement value
· The delta value associated with the last measurement value

· The mean of the squared values of all of the measurements
· The standard deviation of the measurement values
· The slope of the time series, defined as (last measurement value – first measurement value)/(last delta value – first delta value)

We compute each statistic using only records with delta value ≤ 91 days. If a particular feature cannot be computed for a particular subject (e.g., when only one measurement value is present yielding an undefined slope feature), that feature is imputed with the median non-missing feature value across all subjects.

Feature selection

After forming a feature matrix for each task as described above, we proceeded to perform feature selection as follows. First, we ran 10-fold cross validation on the training data for each challenge, fitting either gradient boosted regression or cox proportional hazard models. For each cross validation run, we recorded the relative influence of each feature in the model resulting from that fold. The relative influence is a normalized measure of the reduction in the training objective obtained by splitting on each variable in the course of fitting the model. Since many input features are derived from a given basic feature provided by the challenge, we credited each basic feature with the sum of the mean relative influences (across the cross validation folds) of each of the features derived from them. We then used the six basic features with the highest cumulative mean relative influence. Note that this was carried out in the training data, which in the case of the ProAct tasks had very different basic features provided. This resulted in the selection of basic features such as FVC that were missing in over two thirds of the validation samples. Nevertheless, these basic features proved to provide superior performance to others that were more prevalent in the validation data but were less informative about the outcome of interest.

ProAct Models

Our main concern with these tasks was that the training and validation data are quite distinct from each other. We thus explored various methods for adapting the training data to more effectively learn about the validation data. We settled on an approach similar to importance sampling, in which each sample was weighted by the ratio of the probability that it came from the target (validation) distribution versus the training distribution, where these probabilities were estimated by an L1 penalized logistic regression model trained to distinguish training from validation samples. This model was estimated using all available variables. During training of the final models, this resulted greater emphasis being put on getting the validation samples correct than the training samples; experiments suggested that this resulted in a significant performance boost relative to a base model trained on the training data plus some random subset of the validation data, and evaluated on the remainder of the validation data. The number of iterations/trees in the boosted tree models were tuned by using fitting all training and validation samples together, weighted as described above, with half of the validation samples held out to obtain an estimate of number of iterations achieving the best out of sample error. The final models were fit to all of the training and validation samples.

Registry Models

Our models for these tasks were very straight forward gradient boosted tree regression and cox proportional hazards models. The final models were fit to the entire registry training set using a number of iterations determined by a tuning fit that evaluated performance on a held out subset of the training data.

Team DreamWeaver: Tree-based solution to the ALS Stratification Prize4Life Challenge

Tim Herpelinck and Celine Vens
KU Leuven Kulak, Kortrijk, Belgium

- Will you be able to make your submission public as part of the challenge archive? Yes
- For sub challenge 2, we predicted the probability of being ALIVE at 12,18,24 months.
- Team name: DREAMweaver

Background/Intro

We have provided submissions for sub challenges 1 and 2, i.e., the sub challenges based on the ProAct data [1]. Both sub challenges were addressed by tree-based models, on three aspects: clustering, feature selection, and prediction. For the clustering part, we used a single regression or survival tree, as such a tree provides a (hierarchical) clustering structure over the training instances, driven by the target values. For feature selection, we used the built-in feature ranking mechanism of random forests. Finally, given their excellent performance in many prediction tasks, we also used random forests for the prediction part.

Methods

Data pre-processing

The following modifications were made to the original ProAct input data using Perl.

Data Cleansing

- Duplicate lines were omitted from the dataset.

- All data regarding the concomitant medication given to the patient was removed, along with all clinical trial data acquired after the first three months (delta > 92).
- At this point, the initial training data was divided into two subsets: a slope training set and a survival training set.

Construction of the training set

- To narrow down the number of features we imposed a feature threshold of 10% on both training sets, i.e. at least 10% of the patients must have the feature for it to remain in the dataset.
- Non-numeric lab test values were converted into standard values when possible or were replaced with an arbitrary value.
- Features were divided in standard features, time dependent features (longitudinal) and adverse events:
    - The standard features include the ALS history of the subject, demographics, family history and treatment group (Demographics, ALSHX, FamilyHX, Riluzole, Treatment). Onset_site was treated as two binary features (one for limb and one for bulbar) by assigning a value of 1 if the attribute is associated with the patient and a value of 0 otherwise. In doing so, splits on this feature will correctly recognise patients with both bulbar and limbic onset.
    - For the longitudinal features, we ignore records missing a measurement value. For each original feature we include the following statistics as additional patient features: the first, last, minimum and maximum value, and the slope and intercept (computed by fitting a line through the measurements using Perl package Statistics::LineFit)
    - We transformed the adverse events into binary features: 1 if the patient has a record for the adverse event, 0 otherwise.

The output is generated as a matrix of which each row is defined as a patient and each column as one of the features mentioned above. For sub challenge 1 we obtained 481 features, for sub challenge 2 we obtained 490. Missing values were left as is in the matrix, since they are handled elegantly by the tree and random forest methods. For the final submission, we also added the leaderboard data to the matrix. This matrix is then passed on to the selector.

Selector

To identify clusters of patients with similar outcome, we constructed a regression or survival tree, using R's 'rpart' package [2]. The survival tree resulted in four clusters. We limited the depth of the regression tree to two, in order to obtain maximally four clusters. To select six features per cluster, we split the training matrix in submatrices, each one corresponding to one cluster. Then we constructed a random forest on each of these matrices, in order to obtain a feature ranking for the corresponding cluster. To this end we used the 'randomForestSRC' package [3,4], with 500 regression or 100 survival trees. We picked the top 6 features from this ranking that met the following conditions: (1) the feature is present for at least 80% of the training patients belonging to the cluster (in order to avoid missing values for the test patients; we decreased the value to 75% for the survival sub challenge), (2) there is maximum 1 feature corresponding to each original feature (in order to avoid picking e.g. both the first and the minimum value

of a longitudinal feature) , and (3) the feature is different enough from the other selected features (in order to avoid picking e.g. both salivation and mouth features), this was achieved by using a maximal Pearson correlation coefficient of 0.8 between 2 numeric features. The construction of the trees and the feature selection were done offline.

A test patient's ProAct data is first converted into the same format as the matrix rows. Then the patient is routed down the regression or survival tree, to obtain the cluster id. Finally, the corresponding feature values are written to the selector output file and passed on to the predictor.

R packages: our model was build using standard R packages documented on and downloadable from the Comprehensive R Archive Network (cran.r-project.org). We used the function package 'rpart' version 4.1-10 and 'randomForestSRC' version 1.6.1.

Results

For sub challenge one, we obtained the following regression tree:

- onset_delta>=-484.5
    - Q1_Speech_min< 3.5 -> cluster with 486 training instances and average slope -1.0487750
    - Q1_Speech_min>=3.5 -> cluster with 440 training instances and average slope -0.7579848
- onset_delta< -484.5
    - onset_delta>=-970.5 -> cluster with 946 training instances and average slope -0.6301832
    - onset_delta< -970.5 -> cluster with 492 training instances and average slope -0.3870880

The selected features for the respective clusters are:

- "Q9_Climbing_Stairs","Q2_Salivation","Q3_Swallowing","Q7_Turning_in_Bed","Q5_Cutting","ALSFRS_Total"
- "onset_delta","trunk","Q4_Handwriting","Q5_Cutting","ALSFRS_Total","Headaches"
- "ALSFRS_Total","Q9_Climbing_Stairs","trunk","Q7_Turning_in_Bed","mouth","Q5_Cutting"
- "onset_delta","trunk","Q5_Cutting","Q9_Climbing_Stairs","ALSFRS_Total","Creatinine"

As can be seen, almost all selected features originate directly from the ALSFRS assessment scale.
For sub challenge two, we obtained the following survival tree:

- fvc_last>=2.645
    - Age< 54.95 -> cluster with 2205 training instances
    - Age>=54.95 -> cluster with 3141 training instances
- fvc_last< 2.645
    - fvc_last>=1.751667 -> cluster with 1489 training instances
    - fvc_last< 1.751667 -> cluster with 810 training instances

The selected features for the respective clusters are:

- "Age","Creatinine","Hemoglobin","Q9_Climbing_Stairs","Race","ALSFRS_Total"
- "Race","onset_site_bulbar","if_use_Riluzole","Age","treatment_group","Neurological.disorders.NEC"
- "onset_delta","Gender","fvc_percent","Age","fvc","ALSFRS_Total"
- "fvc","Age","Creatinine","weight","fvc_percent","onset_site_limb"

For the last 2 clusters, up to 4 of the selected features originally included FVC-related features. According to the FVC description in the supplemental PRO-ACT description, we only retained the "fvc" and "fvc_percent" features.

Predictor

For every cluster, identified in the selector phase, we have constructed a random forest starting from the 6 features returned by the selector phase. We used the R package ‘randomForestSRC’ [3,4], which is able to build both regression and survival forests. We used 500 trees in all forests. The output of our predictor is the prediction made by the forest (for the survival forest we check the survival function at days 365,547, and 730).
Note: for sub challenge 2, we predicted the probability of being ALIVE at 12,18,24 months.

Discussion

Our submission is entirely based on tree-based methods. For clustering, a regression or survival tree is used. For feature selection and prediction, random forests are used. For both sub challenges 1 and 2, we obtained four clusters. For sub challenge 1, the clusters are defined by onset_delta and the minimum value of Q1_Speech. Almost all selected features relate to the ALSFRS evaluation score. For sub challenge 2, the clusters are defined by the last value (in the training data) of fvc, and the age. The selected features are more diverse.

References

1. Atassi N. et al. The PRO-ACT database: design, initial analyses, and predictive features. Neurology. 2014 Nov 4;83(19):1719-25.
2. Therneau, T., Atkinson, B. and Ripley, B. (2015). rpart: Recursive Partitioning and Regression Trees. R package version 4.1-10. http://CRAN.R-project.org/package=rpart
3. Ishwaran H. and Kogalur U.B. (2015). Random Forests for Survival, Regression and Classification (RF-SRC), R package version 1.6.1.
4. Ishwaran H., Kogalur U.B., Blackstone E.H. and Lauer M.S. (2008). Random survival forests. Ann. Appl. Statist. 2(3), 841--860.

Authors Statement

This work was done in the context of a summer internship by T.H., under supervision of C.V.

TeamSimon_ALS: DREAM ALS stratification Prize4Life Challenge

[1 1 1] Jouhyun (Clare) Jeon , YuJia (Sylvia) Shiah , and Fan Fan

[1] Ontario Institute for Cancer Research, MaRs Centre, 661 University Avenue, Toronto, Ontario, Canada
(we agree on making our submission public as part of the challenge archive)

Introduction

The goal of the project is to build a prediction models that can predict 312 month ALSFRS slopes (Subchallenge 1 and 3) and a prediction model that can predict survival (Subchallenge 2 and 4). To do this, we built our selector using the random forest algorithm and selected the top 6 features for each patient for all challenges. After the selector has been chosen, we then used regression model to predict ALSFRS slopes for subchallenge 1 and 3 and random forest to predict the probability of death for subchallenge 2 and 4. To achieve optimal performance, we evaluated the ten most commonly used regression models and assess their pearson correlation coefficient (PCC), rootmeansquareerror (RMSE) and Kendall rank correlation coefficient (KRCC) score for subchallenge 1 and 3. The model that has the best scores from all three criteria was then selected as our prediction model. On the other hand, we decided to use random forest for the survival challenge. The performance of the survival model was optimized by testing different *m try* and *n tree* parameters. Overall, we have tested a variety of regression models and selected different model parameters to enhance the prediction ability of both the ALSFR slope and probability of death.

Methods

Data PreProcessing

We processed all our training dataset for subchallenge 14 in R statistical environment (v3.2.1). The training datasets were all preprocessed in a similar fashion. First, we only extracted

features that have numeric values to build our models. One exception is that if the feature was a boolean data type, we then converted this into a binary input 0 and 1. Next, for features that had multiple values, we summarized the common feature by calculating the median value. To ensure that our feature can represent most of the samples, we then selected a threshold to filter our training dataset and discard any missing values. The final results for each training data set are tabulated in Table 1.

Table 1: Training Dataset sample and feature size.

Challenges	Sample Size	Feature Size
SubChallenge 1	1,426	56
SubChallenge 2	1,066	56
SubChallenge 3	392	29
SubChallenge 4	749	29

Feature Selection and Selector Generation

Random forest from the R packages randomForest (v4.610) and randomForestSRC (v1.61) were used to select our features for subchallenge 1, 3 and subchallenge 2, 4, respectively [1,2]. The default parameters were used in random forest to rank our features based on their performance. We then stored this information as a separate file and used this information for our selector to select the top six feature for each patient. An example of the top six features in each subchallenge is tabulated in Table 2.

Table 2: A list of selected features ranked by the importance.

Rank	Sub-challenge 1	Sub-challenge 2	Sub-challenge 3	Sub-challenge 4
1	onset_delta	Age	onset_delta	onset_delta
2	HEMOGLOBIN	FVC_PERCENT	Age	mouth
3	PHOSPHORUS	onset_delta	ALSFRS_Total	ALSFRS_Total
4	ALSFRS_TOTAL	FVC	ALSFRS_R_Total	ALSFRS_R_Total
5	RED.BLOOD.CELLS..RBC.	ALSFRS_TOTAL	hands	Age
6	FVC_PERCENT	LEG	leg	Q1_Speech

We then feed this feature ranking information into the selector along with patient information (features and feature values). Selector will then chooses the first 6 overlapping features that are observed in both the training set and the patient. Since our features were ranked based on feature importance, if the patient did not have the top six feature in the training set it will then pick the next features from the feature ranking list to generate a total of 6 features.

Prediction Model for ALSFRS Slope (SubChallenge 1 and 3)

All regression analyses were performed in R statistical environment (v3.2.1). Ten regression models (random forest, gmb, svmRadial, glmnet, gaussprRadial, rvmRadial, blackboost, kknn, bagEarth, and bayesglm) were test based on the 03 months training dataset. This training set was then divided into two groups to create a separate training (60%) and testing set (40%) to allow us to tune our parameters. Next, we trained our top six features on the new training set to predict the results of testing set. The top four methods that yielded the best performance based on PCC, RMSE and KRCC are used as the prediction model for ALSFRS slope. The parameter of the top four models for each challenge were then optimized using the Caret package (v6.052) (h ttp://topepo.github.io/caret/index.html) . The performance of each model was then assessed using the check_submission on the IBM Server, results are tabulated in Table 3 and Table 4. The best model select for subchallenge 1 is gaussprRadial whereas in subchallenge 3 we used the svmRadial model to predict the ALSFRS slope. The confidence scores were not calculated in any of the models so it is reported as 0. The prediction model for the top six feature that are commonly present in all patient was prerun and save into a RDS file to reduce running time on the IBM Server; whereas for patient who did not possess the common top six feature, prediction model was generated separately for those patients based on the top six feature that the patient possessed.

Table 3: Performance evaluation for different regression models for sub challenge 1

Regression Models	PCC	CIndex	RMSD
glmnet	0.8753	0.8444	0.5109

gaussprRadial	0.9619	0.8667	0.3657
bagEarth	0.8867	0.8222	0.4613
gbm	0.9028	0.8222	0.4682

Table 4: Performance evaluation for different regression models for sub challenge 3

**Regression Models**	**PCC**	**CIndex**	**RMSD**
svmRadial	0.3196	0.5789	0.5049
gaussprRadial	0.1974	0.5158	0.5456
bagEarth	0.2408	0.6053	0.5580
gbm	0.1765	0.5684	0.5621

Prediction Model for Survival (SubChallenge 2 and 4)

All survival analyses were performed in R statistical environment (v.3.2.1). Random survival forest from Party package (v 1.023) is used to generate prediction models for subchallenge 2 and 4 to predict the **p robability of death** [1,2]. Models were fit using Cforest (An implementation of the random

forest and bagging ensemble algorithms utilizing conditional inference trees as base learners). To ensure the performance of our prediction model, we optimized the parameters *m try* and *n tree* from the random forest model. Training sets were first divided into two groups. One (60% of entire Training set) is used to build the prediction model (traintrain). The other (40% of entire Training set) is used to test the generated prediction model (traintest). Top 6 features (ranked by feature importance) are then used to generate and train models. Then the performance of the parameter was evaluated using the Cindex. In total, we tested all combination of six different mtry (16) and 15 ntree (ranging from 50 to 5000) parameter settings. The best combination that gave the highest Cindex is chosen as our prediction model. Optimized parameters and results are tabulated in Table 5.

Table 5: Performance of optimized parameters

**Challenges**	**mtry**	**ntree**	**CIndex**
Subchallenge 2	6	4,000	0.77
Subchallenge 4	6	200	0.72

In addition, prefixed prediction models were generated using the selected top 6 features. If a given patient has these top 6 features, prefixed prediction model is used to measure the probability of death. If a given patient does not have top 6 features, a new model is then trained using the features which the selector provided. Trained model is applied to a given patient to predict the probability of death. Optimized *m try* and *n tree* is used to train the model and predict the probability of death. For the probability of death of 012, 018 and 024 months, month is converted into days using the equation:

days = month X 30.5

Thus, we calculate the probability of death at 366 days (0 12 months), 549 days (0 18 months) and 732 days (0 24 months).

Compile source code

To run source codes:
1. Subchallenge 1
> cd /home/sshiah/als_submission/ProActProgression/
> Rscript selector_sub1_all.R input_file_path output_file_path > Rscript predictorsub1.R input_file_path output_file_path

2. Subchallenge 2
> cd /home/Clare/als_submission/ProActSurvival
> Rscript selector_sub2_all.R input_file_path output_file_path > Rscript predictorsub2.R input_file_path output_file_path

3. Subchallenge 3
> cd /home/sshiah/als_submission/NatRegProgression/
> Rscript selector_sub3_all.R input_file_path output_file_path

> Rscript predictorsub3.R input_file_path output_file_path

4. Subchallenge 4
> cd /home/Clare/als_submission/NatRegSurvival
> Rscript selector_sub4_all.R input_file_path output_file_path > Rscript predictorsub4.R input_file_path output_file_path

Discussion & Conclusion

We have only submitted two results to the leaderboard. The final scoring and summary table is shown in Table 6 (the second submission was excluded because of time constraint).

Table 6: Final scoring for subchallenge 1 on leaderboard

Our first attempt to built our selector was based on patient subtype. ConsensusClusterPlus (v1.22.0) was used to cluster the training dataset into different patient subgroups using kmeans and Pearson's correlation as the similarity metric for each subchallenge [3]. Next, we used different algorithms: random forest, glmnet and stepwise AIC to select the top six features in each cluster and built our classifier based on that. In order to make sure that the testing patients were categorized into the correct cluster, we computed the similarity metric between the given cluster and each patient's features using

zscores/Confidence Intervals. However, to our knowledge, even though patients were assigned into different clusters, the top six features in the cluster don't always match up with the features that test patients possess. In fact, the prediction of ALSFRS slope for some patient used less than 6 features and this could have potentially contributed to our low performance on predicting the slope during the first submission. Therefore, during the second submission, we have decided to discard the clustering method and generated the top six features based on each patient and used random forest Gini importance store to rank our features. In addition, we also tried to improve our

SynapseID	Team	C Index	PCC	RMSD	Rank
syn4935700	TeamSimon_ALS	0.5683	0.0603	0.8166	38

prediction model by including more regression models and optimizing the model parameters (The first submission is based on cForest for the prediction model). In conclusion, based on the final results in Table 3, 4 and 5, we have seen a dramatic improvement on our prediction model even though the IBM Server only tested on a small subset of patients. Our best results were obtained using gaussprRadial and svmRadial regression model as predictor for subchallenge 1, 3 and random survival forest with mtry = 6, ntree = 4, 000 and 200 as predictor for subchallenge 2, 4, respectively.

Authors Statement

JJ, YJS, FF conceived the study. JJ and FF purified the datasets. YJS contributed to cluster analysis. FF contributed to generate selector model. YJS and JJ contributes to optimize and build prediction models for ALSFRS slope and survival, respectively.

References

1. Breiman, Leo. "Random forests." *M achine learning* 45.1 (2001): 532.
2. Ishwaran, Hemant, et al. "Random survival forests." *T he Annals of Applied Statistics* (2008): 841860.

3. Wilkerson, Matthew D., and D. Neil Hayes. "ConsensusClusterPlus: a class discovery tool with confidence assessments and item tracking." *B ioinformatics* 26.12 (2010): 15721573.

PREDICTOMIX: A random forest-based method for feature selection and predicting *probability of death* using clinical trial data collected through the PRO-ACT database

Samad Jahandideh[a], Ehsan Saghapour[b], Adam Godzik[ac]

[a]Bioinformatics and Systems Biology Program, Sanford-Burnham Medical Research Institute, 10901 N. Torrey Pines Rd., La Jolla, California, 92307, United States; [b]Biomedical Engineering Department, Isfahan University of Medical Sciences, Isfahan 81745, Iran; [c]Center for Research in Biological Systems (CRBS), University of California, San Diego, La Jolla, California, United States

Abstract

In this study the Gini index of random forest method was calculated for all input features to evaluate their association with overall survival. Then, we selected six features with the highest Gini indices to train our predictor model. **In this sub-challenge we predicted probability of death**. We will make our submission publically available.

Introduction

In order to tackle the question in sub-challenge 2 we used a random forest method. Random forest provides information on importance of individual features and at the same time predicts output. In this predictive modeling approach, evaluation of features and prediction is done in two separate steps. First, we ran random forest on all features extracted from training set and the Gini index of the random forest algorithm was used to evaluate importance of each single feature (Figure 1). Then, we used top six features to train random forest for prediction of survival. Different functions were used to optimize the structure of random forest.

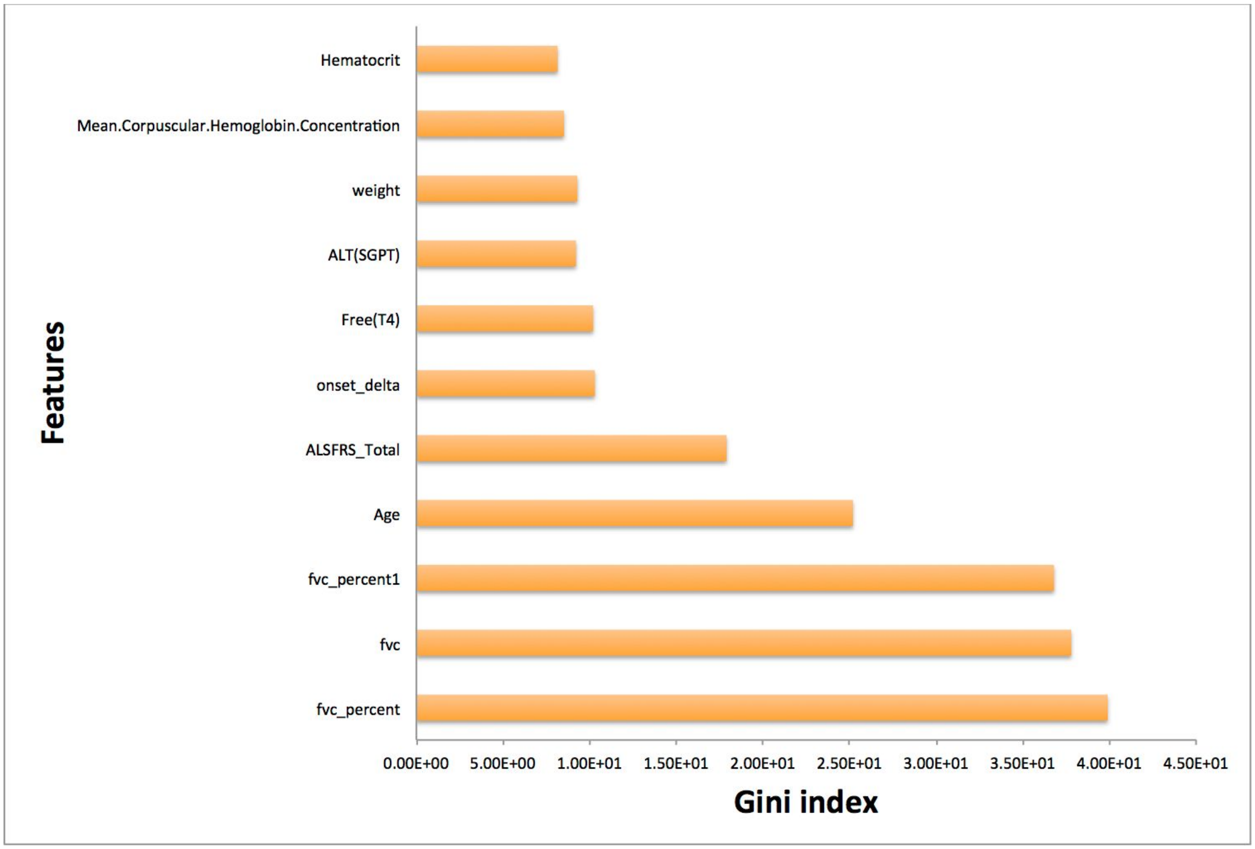

Figure 1. The importance of different variables provided by the importance function (Gini Index) from the random forest algorithm (only top ten features are shown). For each variable feature we calculated minimum, maximum, mean, and median.

Methods

All features were extracted from the training dataset. We used a 91 days cutoff to remove all information provided after 91 days. Then, for each single feature per patient we calculated minimum, maximum, mean, and median. If any data was missing, a median value based on whole data for that specific feature was calculated and used to replace the missing data. In a next step, this set of features was used to train random forest (RF) classifier (Breiman, 2001) against survival status in 0-12 months, 0-18 months, and 0-24 months. In this study, the randomForest (v.4.6-10) R package (Liaw & Wiener, 2002) was used. The number of trees were set to 2000. For other parameters of the RF method, we used default values as provided by the randomForest package. The Gini index of random forest method was calculated for all input features to evaluate their association with ALSFRS survival.

In selector we used fvc, fvc_percent, fvc_percent1, age, ALSFRS_Total, and onset delta as predictor features. Predicted values are reproducible using attached source code and trained models for every single patient. As input data this script accept raw data of a single patient.

Trained models are reproducible running these lines (in R) on attached data file.

```
library(randomForest)
train <- read.csv("/Users/samadjahandideh/Desktop/train.csv")
rf12 <- randomForest(data[,1:24], data[,25], ntree=2000)
rf18 <- randomForest(data[,1:24], data[,26], ntree=2000)
rf24 <- randomForest(data[,1:24], data[,27], ntree=2000)
```

Conclusion

Our methodology has shown acceptable performance (AUC≈0.8) on the leaderboard dataset. We have improved the performance of our method with testing our tuned model on validate and leaderboard sets. Random forest as an advanced machine learning techniques provides possibility of feature selection and prediction at the same time with lower training time compared with the other advanced techniques such as artificial neural network and SVMs.

Authors Statement

S.J. and A.G. conceived the study. E.S. and S.J. preprocessed the data. S.J. developed the predictor model. S.J. wrote the manuscript and A.G. proofread the manuscript.

References

Breiman, L. Machine Learning 45, 261-277 (2001).

Liaw, A. & Wiener, M. R News, 2(3), 18–22 (2002).

Team UglyDuckling: A Boosting approach and Cox model for Predicting Slope and Survival of ALS

Wen-Chieh Fang[1], Huan-Jui Chang[2], Chen Yang[1], Hsih-Te Yang[1,3], Jung-Hsien Chiang[1,3]
[1]Department of Computer Science and Information Engineering, National Cheng Kung University, Taiwan
[2]Department of Economics, National Cheng Kung University, Taiwan
[3]Institute of medical informatics, National Cheng Kung University, Taiwan

Abstract

Our method utilize Gradient boosting method to predict the ALSFRS Slope and utilize the Cox MPL model to predict the survival probability.

Introduction

Boosting is the state-of-the-art machine learning technique for classification and regression and Cox model is well known for survival analysis. Therefore we apply them in this Dream Challenge.

Methods

Data pre-processing

We first disregard those features which 90 percent of values are missing. For example, because 99.9 percent of the values of of feature 'Hepatitis A' are missing, we neglect this feature from the training dataset. For the remaining features with missing values, we replace any missing value with the mean of that variable for all other cases. This missing value imputation has the benefit of not changing the sample mean for that variable. Note that in National Registries dataset, the feature 'Site of onset' has two values: 'Limb' and 'Bulbar'. We simply convert the value 'Limb' into value zero and the value 'Bulbar' into value one. Missing values are imputed as value zero.
Note that for PRO-ACT dataset, we use 7200 patients data and leaderboard data as the training data. Because there is no leaderboard evaluation for community based data from National Registries, we use the original data for training.

Feature selection

For the four subchallenges, we apply equal frequency binning that divides the response variable into several groups such that each group contains approximately same number of values. We empirically choose the number of binning of the response variable for the four subchallenges as three, two, three, and three, respectively. There are two kinds of features: static features(those with one value per patient, e.g., age and gender) and 'time-resolved' features(those with different values when time varies). For the latter, we try two designated measurements, the minimum and the maximum as features. Then for both two kinds of features, we apply feature selection based on information gain to select the top-six features. In order to select optimal features, we run cross validation on the feature candidates. But for the time limitation, we only conduct cross validation on the features that predict ALSFRS Slope, that is, the features for subchallenge 1 and 3.
Table 1 lists the six features of the four subchallenges. Table 2~5 show the information gains of the top six features of the four subchallenges.
We use Orange[1], an open source machine learning and data mining software, to compute the information gain between each feature and the response variable. After we rank all the features based on the information gain, we enumerate a lot of combinations from the top-ranking features and compare their performance using cross validation. Section "Instructions for Source Code" shows how to run our source code and how to rank the features.

Table 1 The selected six features of the four subchallenges

Subchallenge	Feature name
1	" onset-delta ", " trunk ", " Phosphorus ", " Q1-Speech ", " Q5-Cutting ", " leg "
2	" Q7_Turning_in_Bed ", " Q8_Walking ", " Q9_Climbing_Stairs ", " Q10_Respiratory ", " ALSFRS_Total ", "fvc_percent1 "
3	" onset-delta ", " hands-min ", " onset-site ", " trunk ", " Q8-Walking ", " Q2-Salivation "
4	" onset-delta ", " Q1-Speech ", " onset-site ", " Q3-Swallowing ", " mouth ", " ALS-Staging-Total "

Table 2. Information gain of top six features in PRO-ACT dataset for subchallenge 1

Rank	Feature name	Information gain

1	onset-delta	0.067855
2	Q1-Speech-min	0.022115
3	Q1-Speech-max	0.018137
4	Q5-Cutting-min	0.014952
5	trunk-min	0.012234
6	Q5-Cutting-max	0.009622
7	Phosphorus-min	0.008715
8	trunk-max	0.007789
9	leg-min	0.005857
10	leg-max	0.003859

Table 3. Information gain of top six features in PRO-ACT dataset for subchallenge 2

Rank	Feature name	Information gain
1	ALSFRS-Total-min	0.043276
2	fvc-percent1-min	0.042692
3	ALSFRS-Total-max	0.034893
4	fvc-percent1-max	0.030543
5	Q10-Respiratory-min	0.029019
6	Q9-Climbing-Stairs-min	0.027413
7	Q8-Walking-min	0.025462
8	Q7-Turning-in-Bed-min	0.022417
9	Q9-Climbing-Stairs-max	0.021274
10	Q8-Walking-max	0.019354
11	Q7-Turning-in-Bed-max	0.016625

Table 4. Information gain of top six features in National Registries dataset for subchallenge 3

Rank	Feature name	Information gain
1	onset-delta	0.083814
2	hands-min	0.019599
3	onset-site	0.018284
4	trunk-min	0.017362
5	Q8-Walking-min	0.006562
6	Q2-Salivation-min	0.003379

Table 5. Information gain of top six features in National Registries dataset for subchallenge 4

Rank	Feature name	Information gain
1	onset-delta	0.178285
2	Q1-Speech-min	0.041923
3	onset-site	0.040994
4	Q1-Speech-max	0.049715
5	Q3-Swallowing-min	0.044527
6	Q3-Swallowing-max	0.040602
7	mouth-max	0.054351
8	ALS-Staging-Total-max	0.042227
9	mouth-min	0.033982

ALSFRS Slope Prediction

We apply Gradient Boosted Regression Trees (GBRT) to predict the ALSFRS slopes. GBRT computes a sequence of simple decision trees, where each successive tree is built for the prediction residuals of the preceding tree. We search the optimal parameters of GBRT by searching the parameter space for the best ten fold cross-validation score.

In our feature selector, we make sure that all the inputs do not contains feature delta > 3 months (92 days). Otherwise, we delete them.

Survival Probability Prediction

We apply Cox model with with maximum penalized likelihood [2] to predict the survival probability. In our approach, we predict the probability of survival (staying alive), not the probability of death.

Conclusion

In this challenge, we think that the feature selection is one of the most important steps and we believe that the most appropriate features dominate the performance of the model. By Oct. 20, 2015 our team will make the project publicly visible.

Instructions for Source Code

Code Guidance

The following are the directory structure of source code of the four subchallenges.
Subchallege 1
ProActProgression/
predictor.py
predictor.sh
preprocessor.R
csv_gen.R
README
selector.R
selector.sh
Subchallege 2
ProActSurvival/
predictor.R
predictor.sh
preprocessor.R
csv_gen.R
README
selector.R
selector.sh
Subchallege 3
NatRegProgression/
predictor.py
predictor.sh

preprocessor.R
csv_gen.R
README
selector.R
selector.sh

Subchallege 4

NatRegSurvival/
predictor.R
predictor.sh
preprocessor.R
csv_gen.R
README
selector.R
selector.sh

Common Steps for four Subchallenges

1. Prepare the data

```
Data should be put in a directory named "data", which located at the same directory as preprocessor.R, so before processing ALS data, please create a directory named "data" and fill the files required. "$$ TrainingDataSet.csv $$", "$$ TrainingSet.csv $$", "national-top-6-ver1.csv", and "$$ train_for_model.csv $$" are respectively produced from the output of "$$ preprocessor.R $$" in the four subchallenges. Note that the final best feature sets are determined by a open source software called orange, its website: http://orange.biolab.si/. One can see the following section "Orange Operation" to see how to use orange to derive the feature ranking. The following is the files one need to prepare for training the model.
```

Subchallege 1

data/
all_forms_PROACT.txt
all_forms_validate_leader.txt
all_forms_validate_spike.txt
ALSFRS_slope_PROACT_filtered.txt
ALSFRS_slope_validate_leader2.txt
ALSFRS_slope_validate_spike_filtered.txt
surv_response_PROACT.txt
surv_response_validate_leader.txt
surv_response_validate_spike.txt

Subchallege 2

data/
all_forms_PROACT.txt
all_forms_validate_leader.txt
all_forms_validate_spike.txt
surv_response_PROACT.txt
surv_response_validate_spike.txt
surv_response_validate_leader.txt

Subchallege 3
data/
all_forms_registry_training.txt
ALSFRS_slope_registry_train_filtered.txt
Subchallege 4
data/
all_forms_registry_training.txt
surv_response_registry_training.txt

1. Prepare the environment

```
Environment:
        * R version 3.2.1 (2015-06-18) -- "World-Famous Astronaut"
        * python 2.7.9
        * predictor.py can run on the IBM machine (python 2.7.10)



Serveral libraries have been used by preprocessor.R, so install them first:
        * python libraries:

                - pandas
                - numpy

        * R librarys:

                - foreach
                - doParallel
                - plyr

R packages can be installed through commands below:

        $ R

        > install.packages("foreach")
        > install.packages("doParallel")
        > install.packages("plyr")
```

1. Run the program

```
$$ $ Rscript preprocessor.R $$
    $$ $ Rscript csv_gen.R $$

By enter the command above, the script would prepare data for our predictor model. The
```

```
output of this r script would be 3 csv files: subjects to time-independent features
and subjects to time-dependent features ( maximum value and minimum value of each
feature ).
```

```
    $$ $ sh selector.sh  $1  $2  $$
    $$ $ sh predictor.sh  $1  $2  $$
```

```
The variables ( $$ $1 $$ and $$ $2 $$ ) are input and output of each shell script
respectively.Both $$ $1 $$ of the commands are data of a single subject. For example:
```

```
$$ $ sh selector.sh 329.txt 329.out1.txt $$
$$ $ sh predictor.sh 329.out1.txt 329.out2.txt $$
```

1. Postscript

```
* Execution time
```

```
On a 64-core machine, preprocessor.R takes approximately 3.5 hours.
```

Orange Operation

Orange-Operation.pdf

References

1. Demšar, J., Curk, T., & Erjavec, A. Orange: Data Mining Toolbox in Python; Journal of Machine Learning Research 14(Aug):2349−2353, (2013).
2. Ma, J., Heritierc, S., and L^o, S. N. On the maximum penalized likelihood approach for proportional hazard models with right censored survival data. Computational Statistics and Data Analysis, 74:142-156, (2014).

Authors Statement

Wen-Chieh Fang coordinated the team, wrote the write-up, led much of the work, and taught members the machine learning techniques ; Huan-Jui Chang operated the orange tool for feature ranking, performed the data imputation, performed the predictions for the subchallenge 1 and 3, and ran the cross validation

for RMSD and Pearson's correlation performance ; Chen Yang performed the data cleaning, performed the predictions for the subchallenge 2 and 4, accomplished the submission task, and ran the cross validation for c-index performance; Hsih-Te Yang and Jung-Hsien Chiang supervised the overall project. Wen-Chieh Fang, Huan-Jui Chang, and Chen Yang contributed to the common discussions and decision-making through-out the project.

Acknowledgements

The authors would like to thank Mu-Hung Tsai and I-Te Tsai for medical knowledge consultation.

Team chern: Modelling ALS progression using time-dependent Hurst exponent

Anatoly Chernyshev
The Research Council of Oman, Azaiba, Sultanate of Oman

Introduction

The presented model aims at development of universal, patient-independent prediction of ALS progression. "Patient-independent" means that the same set of parameters is used for every subject: ALSFRS total, onset delta (if known), and age (for the survival prediction only).
Plotting the data available the database showed that in the most cases the progression pattern follows the one depicted below.

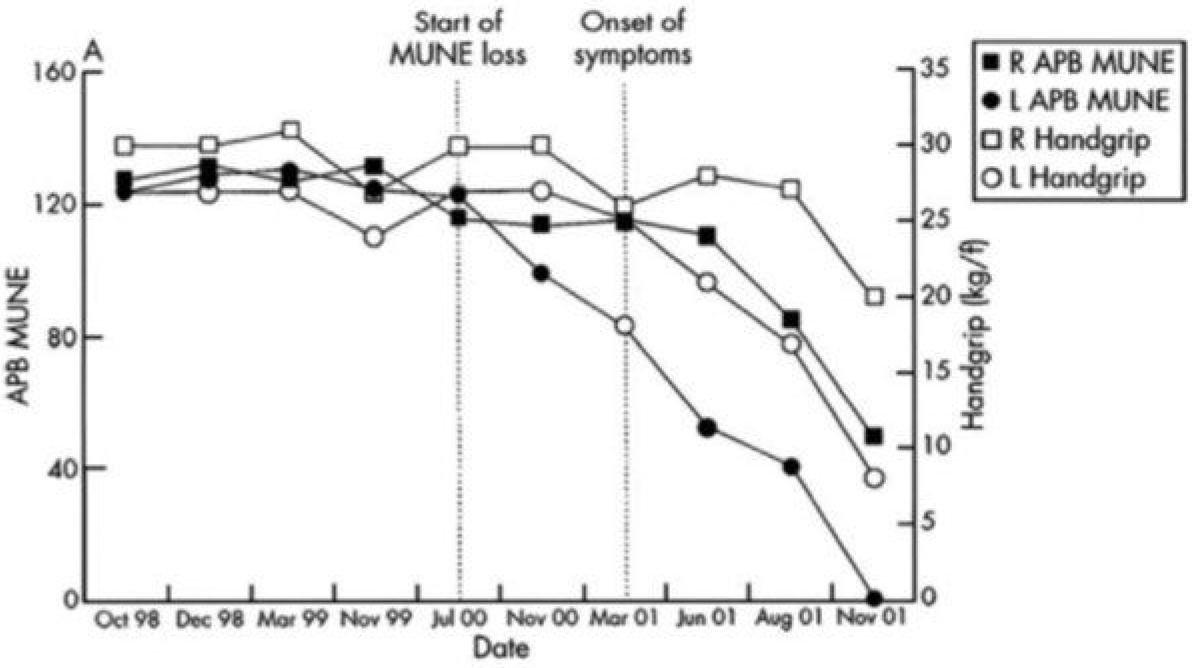

Figure 1. Preclinical loss of motor units by a human SOD1 mutation carrier as depicted by loss of number estimates (MUNE) prior to onset of symptoms [1].

These kind of curves could be best described with a “Brownian motion” system, when the state of the system (ALSFRS total) drifts away from its original value of 40 according to the following general formula:

$ALSFRS = 40 - ct^{H}$ (1)

Where 40 is the starting value of ALSFRS total; *t* is the time (delta); *c* is a process-specific constant; *H* is the Hurst exponent [2]. The latter shows the propensity of the process to continue in a given direction. The Hurst equation above is a generalization of original formula of Brownian motion (*H*=0.5) and is being used nowadays to describe a range of complex phenomena, from financial markets, to dynamical diseases.

Methods

First attempt has been made to model the declining part of ALSFRS curves using linear regression of ln(ALSFRS) vs. time (*H*=1). This method, however, was prone of underestimating the 3 to 12 month slope (see Fig. 2 below).

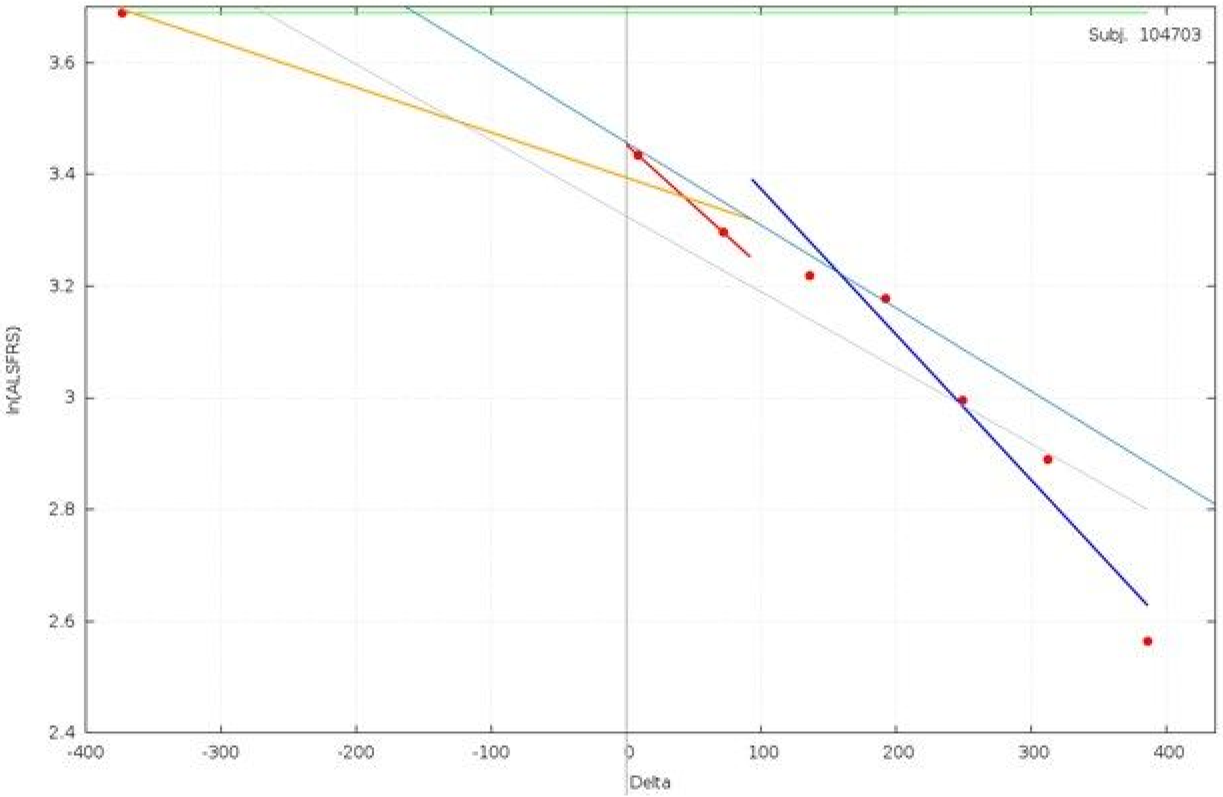

Figure 2. Example of 3-12 month slope prediction using simple linear regression (light blue line). Dark blue: actual 3-12 month progression; red: actual 0-3 months progression; orange: actual onset to 3 months progression; grey: linear regression over all available data points.

The second attempt was to do a proper non-linear fit of ALSFRS data to eq. 1. In this case the algorithm did overestimate the slope (Fig. 3).

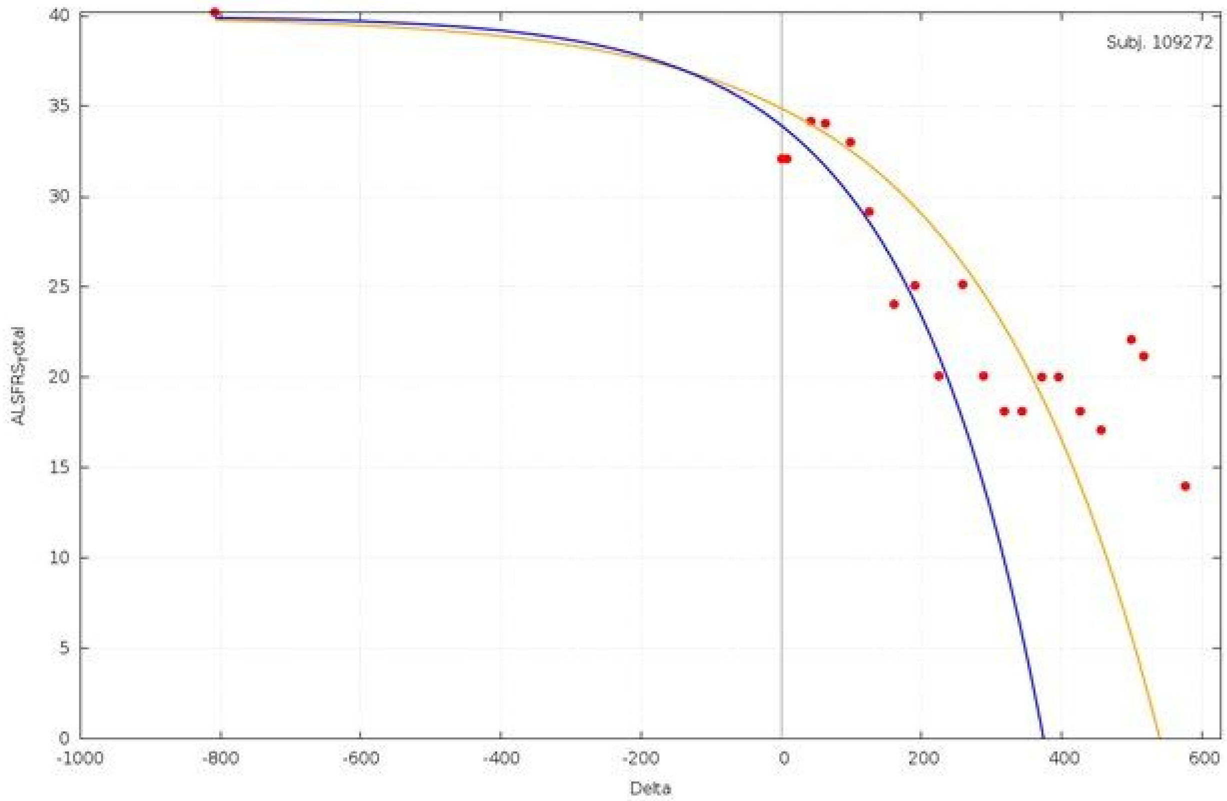

Figure 3. Example of fitting ALSFRS data to eq. (1). Blue line: prediction based on the data from onset to 3 months; Yellow line: regression using all data points.

The final model is a linear combination of these two approaches. The fraction contribution of the linear model is governed by a form of logistic equation:

y=11+(xx0)b (2)

In a typical case, the ALS progression is governed by the non-linear model on early deltas, gradually switching to the linear model (Figure 4), hence it could be best described as a Brownian motion with time-dependent Hurst exponent:

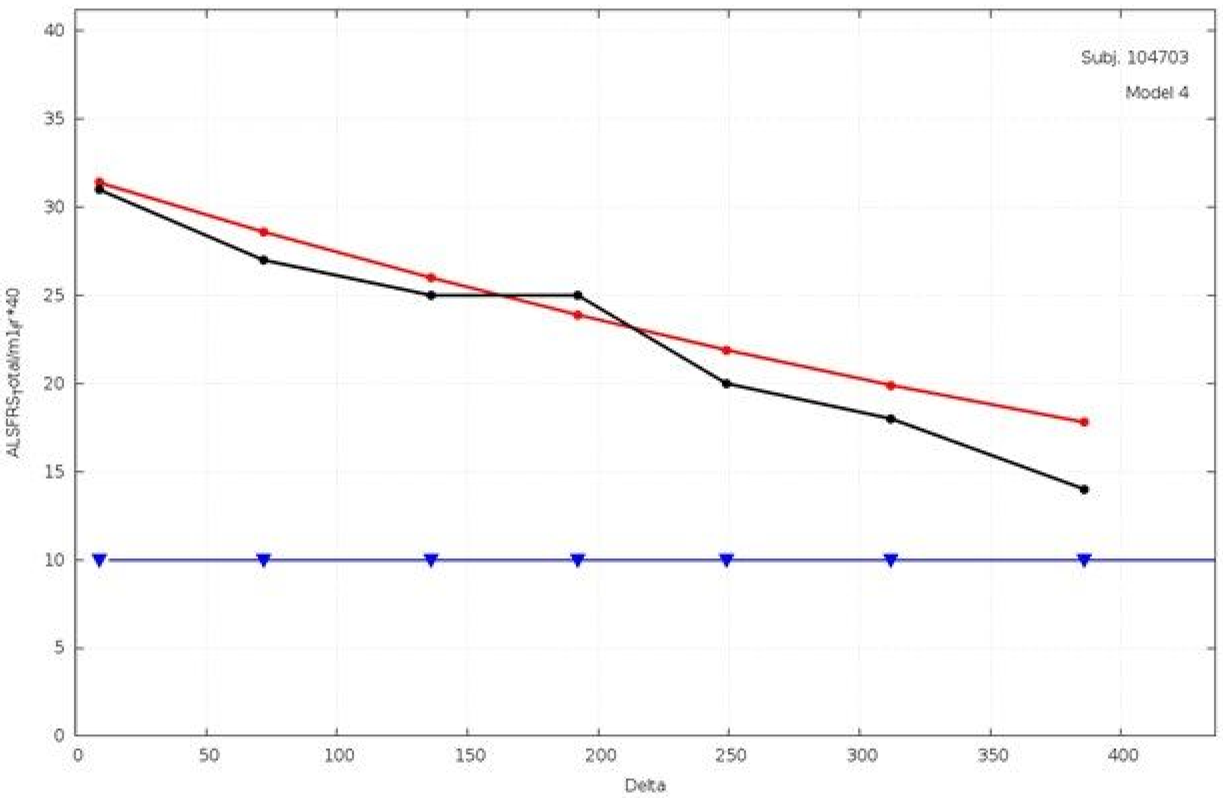
Subj. 104703
Model 4
ALSFRS_Total/m1_f*40
Delta

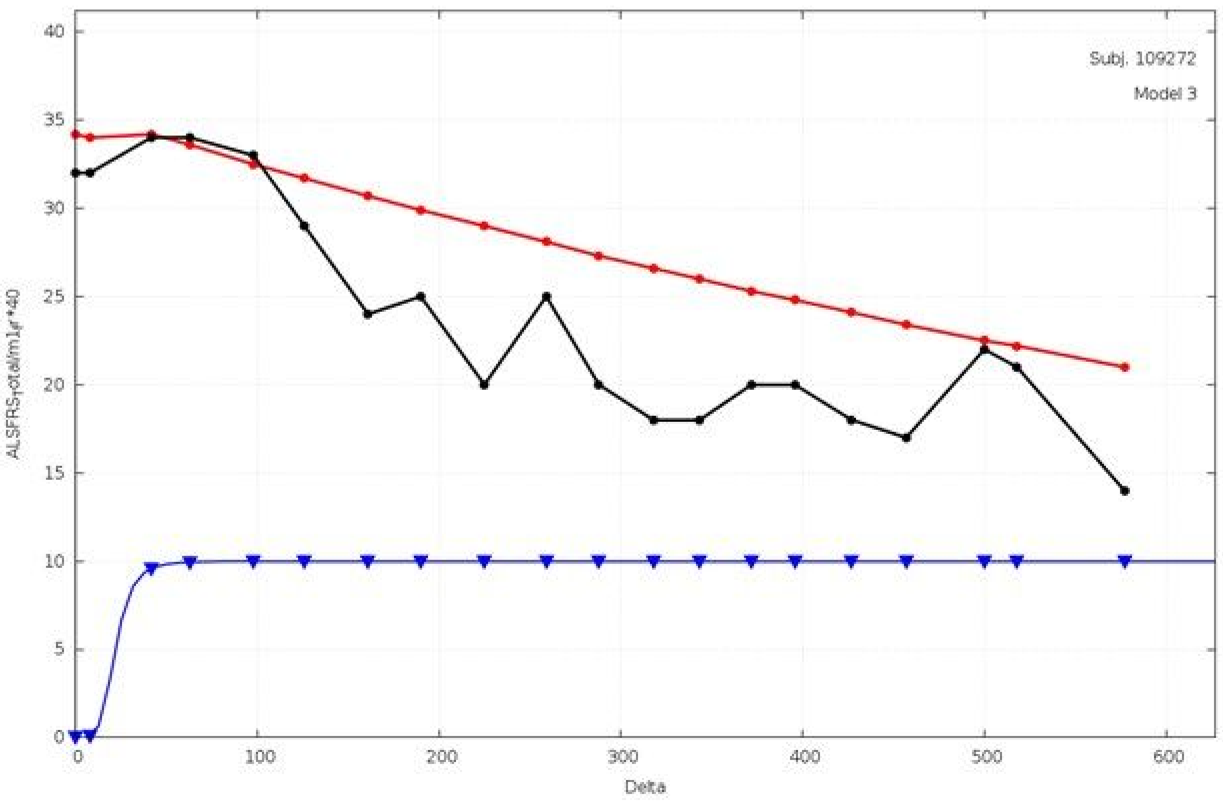
Subj. 109272
Model 3
ALSFRS_Total/m1_f*40
Delta

Figure 4. Examples of ALSFRS prediction using combined model (for the same subjects as on Fig. 2,3). Black: actual data from PROACT DB; Red: predicted progression; Blue: governing logistic function (x10) for the subject.

For the estimation of death probabilities, a logistic modelling was undertaken. There were three strong correlations found: between logit(P) and age, logit(P) and ALSFRS2, logit(P) and 20 days ALSFRS slope. ALSFRS and its slope has been calculated from the model above, no actual data was used. The corresponding plots are given on Fig. 5–7.

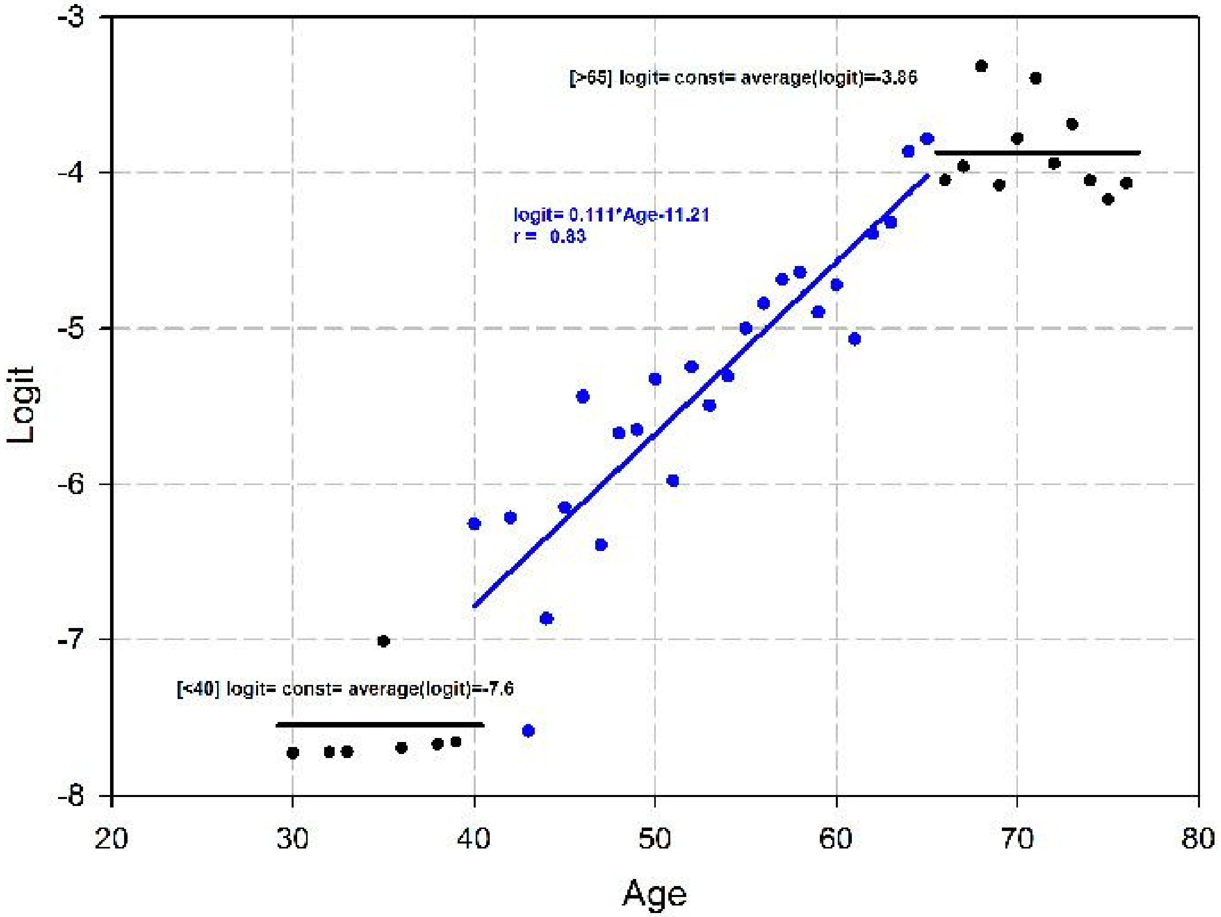

Figure 5. Plot of logit(P) vs age. At the age below 40 and above 65 years the logit is taken to be a constant (-7.60 and -3.86 respectively).

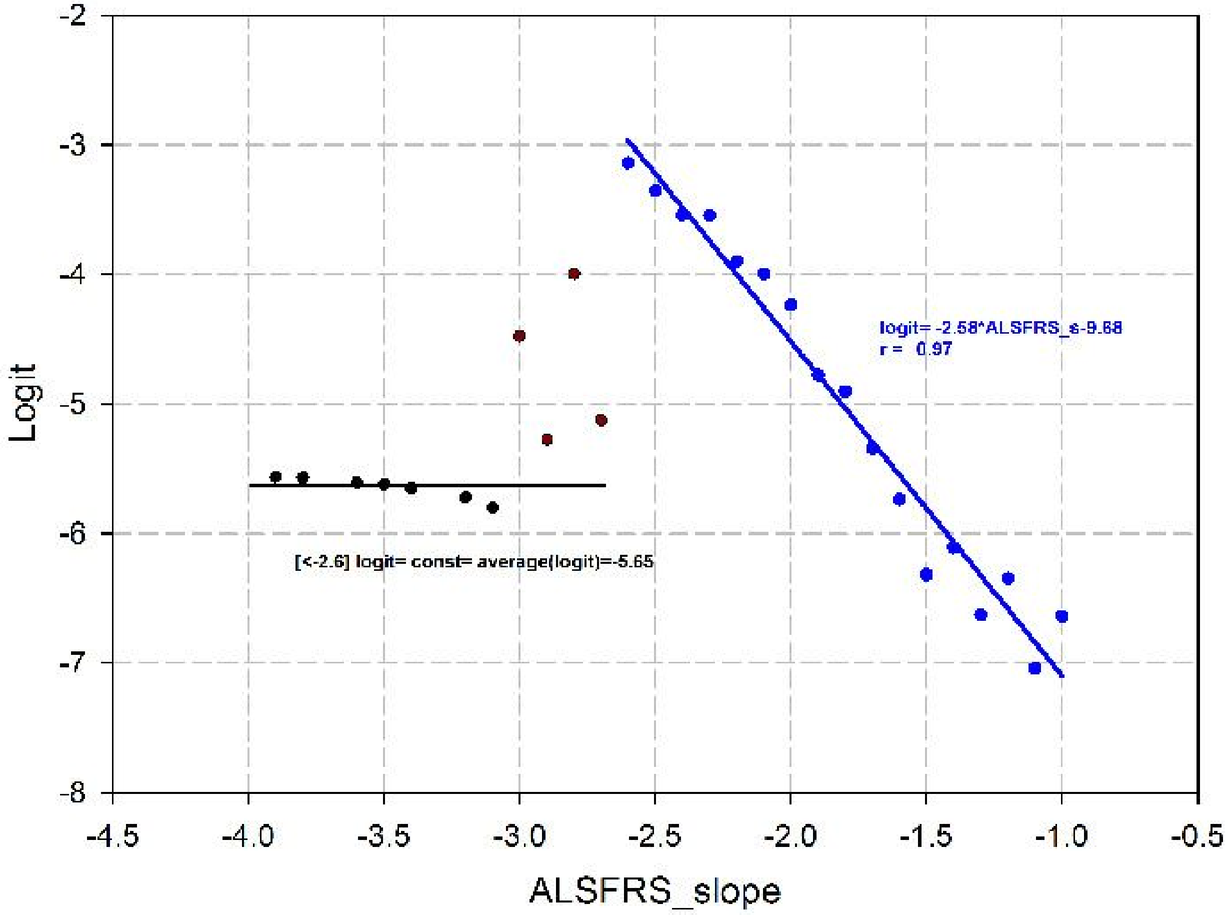

Figure 6. Plot of logit(P) vs 20 days ALSFRS slope (as calculated from the model). When the slope is less than -2.6, the logit is taken as constant (-5.65).

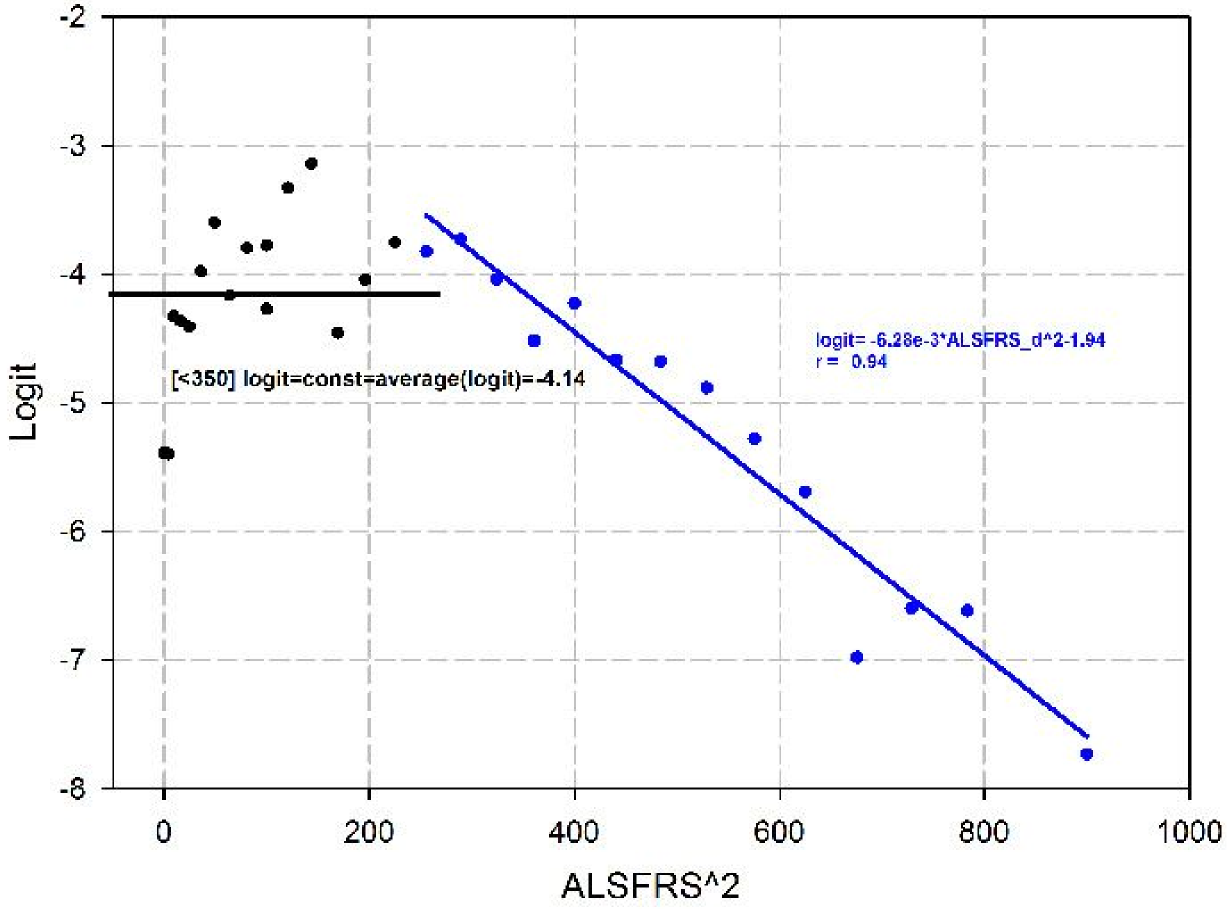

Figure 7. Plot of logit(P) vs $ALSFRS_2$ (as calculated from the model). When $ALSFRS_2$ is less than 350, the logit is taken as constant (-4.14).

A daily probability of death is calculated by addition of three logistic equations:

$$P(death)=\frac{1}{1+\exp(-logit(age))}+\frac{1}{1+\exp(-logit(ALSFRSslope))}+\frac{1}{1+\exp(-logit(ALSFRS_2))}$$

Then for every period of interest (e.g. 12 months) the total probability is calculated as a sum of daily probabilities. A correction factor of 0.0244 is uniformly applied at the end to obtain the reported probability.

In the case when no ALSFRS data is available for a patient, the average values were assigned to the 3–12 months slopes, and to the death probabilities. Their values are as following:

3–12 months slope: -0.26;

Death probability (12 months): 0.20;

Death probability (18 months): 0.35;

Death probability (24 months): 0.49;

All programming was done in Ada language, using GNAT GPL 2015 compiler. The code is compiled using the following command lines:

gnatmake d5s.adb - to make the selector, which is the same for both progression and survival test

gnatmake d5p.adb - to make the survival predictor

gnatmake m5p.adb - to make the progression predictor

Other relevant files are als_stat.ads and als_stat.adb (package containing common services).

All other uploaded files are necessary dependencies, and must be present in the compilation directory.

Conclusion

The suggested model offers unified approach to predict ALS progression, using well grounded concept of dynamical diseases [3]. Despite its relative simplicity, the model has demonstrated quite good performance compared to modern data analysis software. Further developments will largely depend on the level of interest from ALS community. I have no objections to reveal this submission to the public.

References

1. Murray, B., Natural History and Prognosis in Amyotrophic Lateral Sclerosis, in Amyotrophic Lateral Sclerosis, H.P. Mitsumoto, S and P. Gordon, Editors. 2006, Taylor & Francis: New York. p. 227-255.
2. Hurst, H.E., The Long-Term Storage Capacity of Reservoirs. Trans. Am. Soc. Civ. Engineers, 1951. 116: p. 770-799.
3. Glass, L., Dynamical disease: Challenges for nonlinear dynamics and medicine. Chaos, 2015. 25(9): p. 097603.

Team CompassRose: Feature Selection and Prediction in ALS Disease Progression

Joseph Durgin[1], David Wadden[1]
[1] Bryn Mawr College Postbaccalaureate Premedical Program
Submission will be made part of public archive.

Background and Introduction

Like the first Synapse ALS challenge, this ALS stratification challenge asks participants to predict disease progression in ALS patients. Unlike the first challenge, however, it asks for this prediction to be made using only six features per patient, and also asks participants to predict probability of survival at three time points.

In formulating our approach to this problem, we began with methods that had proved fruitful for others in the first ALS challenge: transform the time-series data to extract features, and use a tree-based ensemble model for prediction. In particular, we used an implementation of gradient boosting (implemented in R's `gbm` package). To extend the model to meet the requirements of this challenge, we used a combination of statistical hypothesis testing and examination of variable importances obtained from our models to select the 6 most informative features for prediction of survival and disease progression.

Tree-based ensemble methods, and the `gbm` package in particular, seemed well-suited to the data and goals of this challenge for a number of reasons.

1. It is robust against irrelevant features.
2. It easily handles missing data without requiring imputation before the data are passed to the model.
3. It is not overly sensitive to data transformations.
4. It provides a measure of variable importance, which can be used for feature selection.

Gradient boosting is a very popular and well-known machine learning technique. Our contribution here is in applying this technique to a complex data set of potentially great importance, and extracting the variables which allow for optimal prediction.

Methods

Variable Selection and Transformation

Static data

Static data can be specified with a single time-independent value. In the PROACT data, static data included demographic information, family history, and whether or not the subject used Riluzole and was included in a treatment group. It also included information about ALS history, for instance the onset_delta. Since there were relatively few static variables and boosting is fairly robust to irrelevant features, all static data were included in the model.

Episodic data

Episodic data can be specified with a stop time and a start time. These data included concomitant medications and adverse events. There were a lot of these; nearly 6,000 medications and about 250 adverse events. Since there were only roughly 2,000 patients in the PROACT data set with ALSFRS measurements, this seemed like too many variables to include. To pre-screen from the medications and adverse events, we did the following:

- Throw out all entries with a start time after 3 months. Eliminate the date information from the remaining entries. That is, score a patient as "yes" if he ever took the medication, and "no" otherwise.
- From the remaining data, throw out all medications and events with data for less than 30 subjects. This yielded 87 medications and 50 adverse events.
- For each of the remaining medications and events, perform a Mann-Whitney U test (implemented via the R function `wilcox.test` to determine whether the ALSFRS slope is significantly higher or lower in subjects taking the drug or experiencing the event.
- Examine the p-values. If any seem significant, keep the top few hits.

This analysis yielded 6 medications and 6 adverse events to be included as features in the model. For illustration, the 6 concomitant medications are included, along with a plot of the p-values for all medications and the distribution of ALSFRS slopes for those who took and did not take amitriptyline (the top hit).

form_name	p

AMITRIPTYLINE	0
LUVOX	0.0023
RILUZOLE	0.0038
NORTRIPTYLINE	0.0126
QUININE SULFATE	0.0128
BACLOFEN	0.0166

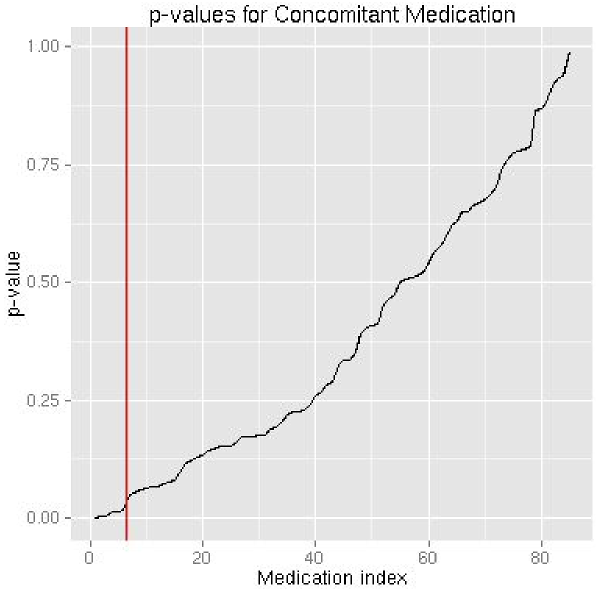

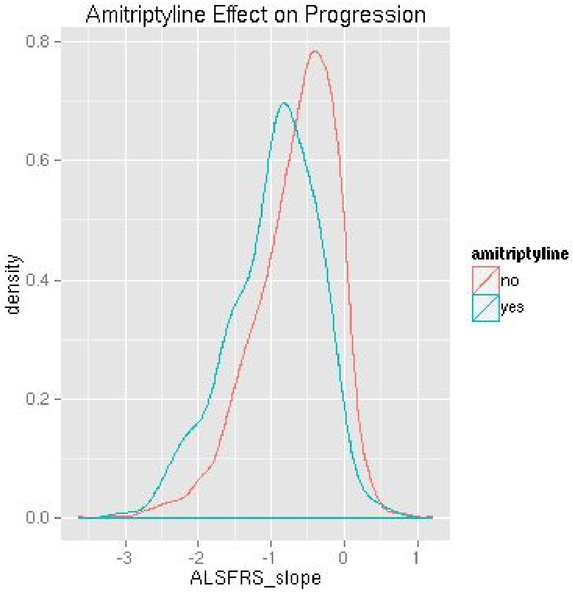

Left: Distribution of p-values for concomitant medications. The medications to the left of the red line were included in the model.
Right: Subjects who took amitriptyline tended to have faster progressions.

Time Series data

Time series data consist of measurements of an attribute across a number of visits. Many data in the PROACT data set were time series data, including the ALSFRS data, forced and slow vital capacity, lab test, and vital statistics (e.g. weight, pulse). Slow vital capacity measurements were present in roughly 200 patients who also had ALSFRS slope data. Given the small number of measurements, we discarded these data. We also discarded the lab tests. Features were extracted from the remaining data in a manner similar to what was done by one of the participants in the first challenge. We computed the following summary statistics:

1. mean
2. standard deviation

3. first measurement
4. last measurement
5. max measurement
6. min measurement
7. slope, (y_last - y_first) / (x_last - x_first)

We also computed a "derivative" time series and computed the same summary statistics, for a total of 14 summary statistics per time series feature.

Differences in the Registry data

In addition to the ALSFRS data on ALS disease progression, the registry data had measurements of a number of "ALS_Staging" variables. We included these variables in a progression model, but the resulting generalization performance was worse than without them. Hence they were discarded. Similarly, all MEDHx (i.e. medical history) variables proved uninformative and were discarded.

Modeling and Feature Selection

Variable Selection

When a `gbm` model is fit, it provides a ranking of variable importances. For each time series variable, we took the maximum ranking of all the extracted features (this performed better in performance on the held-out "spike-in" set than, say, the average ranking). We then rank-ordered all variables by their model importance. For a given subject, the selector took the 6 most important features for which the subject had data.

var	rel.inf
onset_delta	33289.3018
ALSFRS_Total_deriv_slope	9446.7327
fvc_percent_last	3891.061
weight_slope	2977.7997
ALSFRS_Total_slope	2852.713
fvc_deriv_slope	2757.5096
fvc_percent_slope	2660.9968
BMI_slope	2622.9385

ALSFRS_Total_min	2454.0531
ALSFRS_Total_last	2397.2311

Prediction

Since `gbm` can natively handle missing data in both the fitting and prediction stages, we made predictions by simply passing the fitted `gbm` model the 6 features selected for each patient. No imputation was required by us in either the training or prediction phase. Prediction was made using the number of trees that yielded the smallest cross-validation error.

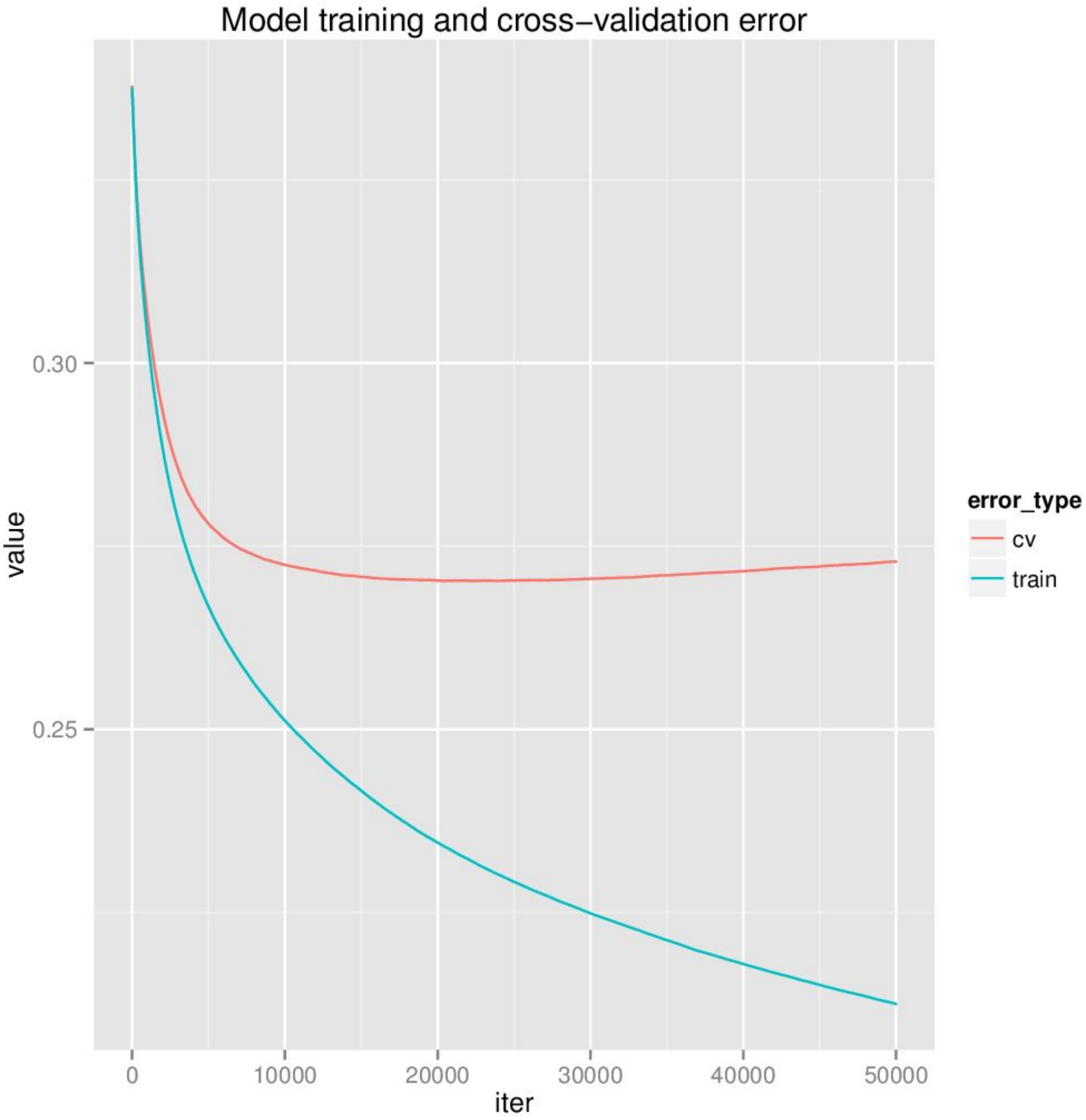

Training and cross-validation error of `gbm` model as a function of the number of boosting iterations.

Conclusion/Discussion

Perhaps the most interesting discovery from this project is that six features are sufficient to make predictions of ALS outcomes that were not drastically worse than what was possible using all features. As in the first challenge, by far the most informative feature was onset delta. Disappointingly but not

unsurprisingly, not one of the more than 5000 medications taken by patients had a demonstrably strong affect on ALS progression.

There are a number of possible improvements that could be made upon our work here. We were unable to use the lab test data due to time constraints. In the interest of time, we also performed all variable selection based on performance on ALSFRS slope prediction. It is quite possible that some variables that were uninformative for slope could have been informative for survival. We also did not take advantage of the stratification aspect of this challenge, in the sense that we simply assigned all patients a cluster ID of 1. There has been discussion on the forums about the difficulties of using cluster ID's without leaking data. It is our hope that other teams have explored these possibilities.

Authors Statement

J.D. and D.W. contributed equally to this work.

References

1. Hastie, T., Tibshirani, R., & Friedman, J. The Elements of Statistical Learning.
2. Küffner, R. et al. Crowdsourced analysis of clinical trial data to predict amyotrophic lateral sclerosis progression. Nature Biotechnology 33, 51-57 (2015)
3. Greg Ridgeway with contributions from others (2015). gbm: Generalized Boosted Regression Models. R package version 2.1.1. http://CRAN.R-project.org/package=gbm

Team dinithins: ALS Survival Prediction With Decision Trees

Dinithi N. Sumanaweera
Department of Computer Science and Engineering, University of Moratuwa, Sri Lanka
I am able to make my submission public as part of the challenge archive.

Introduction

In sub challenge 2, the task was to predict the survival of ALS patients in terms of the first 12 months, 18 months and 24 months respectively since the trial onset. The problem was in the form of supervised learning as the clinical data of 7067 patients were available to be used for training a learning model which is capable of providing three survival probability outputs in the presence of a new patient instance. Basically it falls under the category of a classification problem and thus, an attempt was made to firstly understand the nature of data and ways of preprocessing them for resolving any inconsistencies, for achieving completeness through missing value imputation and for preparing them in such a way that the intended prediction model will be able to exploit certain hidden features. The ultimate goal of the challenge was to find a clustering of patients so that only the most relevant 6 features for a particular cluster have to be selected for training the model. However, in this gradual approach, only a single cluster is assumed to be existing. The six features were selected only after deciding on the most appropriate type of model to be used. In this approach, a random forest model was identified to be suitable for the purpose. The six features were selected upon feature importance.

Methods

Background of data

The training dataset is somewhat complex as it is longitudinal (in which, multiple records are there per each subject), right censored (as the subject might have left the study before the event has occurred) and left truncated (as the subject might have been at risk before entering the study). There are 12 major types of parent features; namely "Adverse Event", "ALSFRS", "ALSHX", "Concomitant Medication", "Demographic" , "FamilyHx", "FVC", "Lab Test" "Riluzole", "SVC" , "Treatment" and "Vitals", with 2230 different features. This high dimensionality along with the fact that some of these features account for multiple value instances over different time points and the considerably large amount of missing values have

contributed towards the extremely intrinsic nature of the data. Following are some observed descriptive statistics.

- Only 6565 patients have demographic information
- Out of the patients, 0.033% are American Indian, 0.58% are Asians, 1.074% are Black, 0.281% are Hispanic, 0.1983143% are other, 23.99603% are unknown, 73.83903% White.
- Only 6051 patients have gender data: 38.60519% is female and 61.39481% male.
- We can observe a skewed class distribution for survival: 33.60691% have not survived whereas 66.39309% have survived or not reported with death.
- Only 4838 patients have ALSFRS data

Data Preprocessing

This longitudinal dataset was first transformed into a normal data set where each patient has a unique feature vector. The feature units were ignored in the process and the redundant records were removed. Some notable data transformations and data aggregations were made as follow.

- Average height was computed as it is very unlikely to change with time
- BMI value was transformed into the units of Kilograms and meter cube (the standard) as the original values were in Kilograms and centimetres. The values were rounded to 2 digits.
- New features were created for time series attributes by computing mean and standard deviation of the values as well as the mean and standard deviation of the rate of change in the values during the period of given time points.

Method

Firstly an elementary train data set was constructed with age + gender + race + family_ALS_hist + treatment + treatment_start + diag_delta + onset_delta + onset_site + if_use_Riluzole + riluzole_delta. Before predicting 3 survival probability figures with 6 features, the initial attempt was made to predict the overall survival by having all features and compare accuracies of 3 models; decision tree from [party: https://cran.r-project.org/web/packages/party/index.html], random forest from [party: https://cran.r-project.org/web/packages/party/index.html] with 100 trees, ADA Boost from [adabag: https://cran.r-project.org/web/packages/adabag/index.html] with 100 trees. These three models were chosen because the decision tree is an intuitive classification algorithm which relies upon attribute importance at rule generation and the latter two are ensemble approaches of the former. Two methods; roughfix (using median/mode) from [randomForest: https://cran.rproject.org/web/packages/randomForest/randomForest.pdf] and K nearest neighbour (kNN) imputation from [VIM: https://cran.r-project.org/web/packages/VIM/index.html] were primarily used for missing value imputation. However, with the initial results, it was clear that the kNN is more appropriate for the task.
After training the 3 models over elementary train data, they were evaluated on test data using ROC plots [1]. Then in the next step, the 3 models were trained upon the merge of elementary dataset and Total ALSFRS score statistical data (mean_alsfrs + sd_alsfrs + mean_alsfrs_rate + sd_alsfrs_rate). It was observed that the statistics gave quite a good improvement. Finally, vitals data were added in terms of statistical figures upon time deltas. Then the 6 features were selected upon the understanding of feature importance by observing the measures via generalised boosting model from [gbm:

https://cran.r-project.org/web/packages/gbm/gbm.pdf] as well as the random forest from [party: https://cran.r-project.org/web/packages/party/index.html].

method	auc	threshold	sensitivity	specificity
Decision Tree	56.503	0.190	88.889	24.031
Random Forest	55.383	0.224	88.889	26.357
ADA Boost	53.592	0.188	84.444	30.233

method	auc	threshold	sensitivity	specificity
Decision Tree	57.218	0.208	84.444	30.233
Random Forest	45.874	0.335	51.111	55.039
ADA Boost	58.898	0.447	53.333	65.116

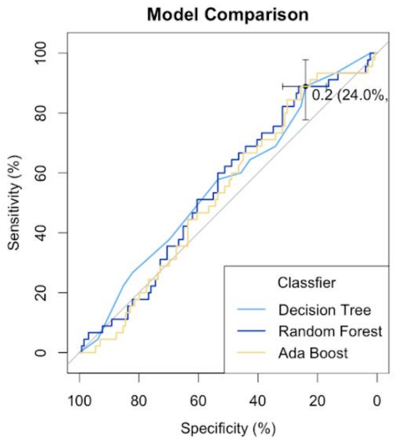

with median/mode imputation

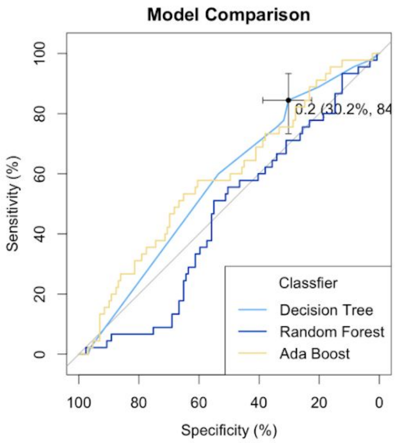

with kNN imputation

Figure 1: ROC plots for the models trained over elementary train data

method	auc	threshold	sensitivity	specificity
Decision Tree	75.125	0.511	64.444	79.845
Random Forest	77.847	0.356	86.667	62.016
ADA Boost	77.158	0.324	88.889	56.589

method	auc	threshold	sensitivity	specificity
Decision Tree	79.793	0.185	93.333	56.589
Random Forest	81.309	0.276	80.000	76.744
ADA Boost	74.987	0.367	88.889	51.163

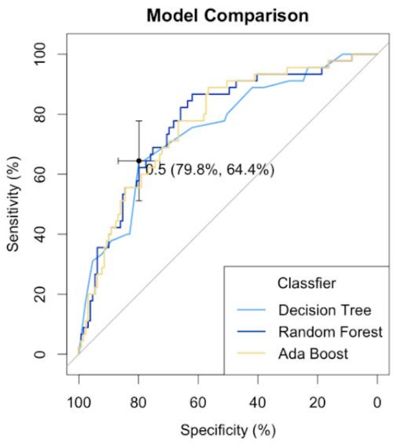

with median/mode imputation

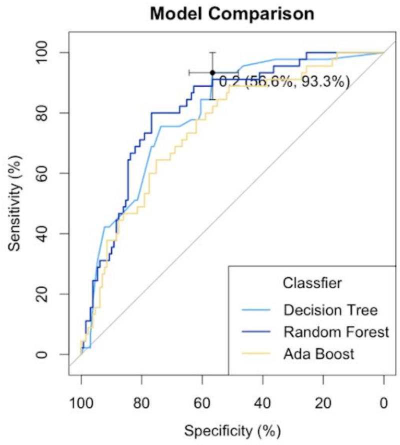

with kNN imputation

Figure 2: ROC plots for the models trained over elementary train data + ALSFRS Total score statistics

Since kNN gave good results with ALSFRS statistics, it was decided to be used as the imputation technique.
As it was observed in previous results that the statistical figures over time deltas can improve predictions, it was decided to compute them for each time period of 0-12 months, 0-18 months and 0-24 months, for ALSFRS and Vital data. Following results are for model training over the entire data set.

method	auc	threshold	sensitivity	specificity
Decision Tree	75.693	0.315	82.222	68.217
Random Forest	80.810	0.202	88.889	65.891
ADA Boost	71.817	0.456	64.444	72.093

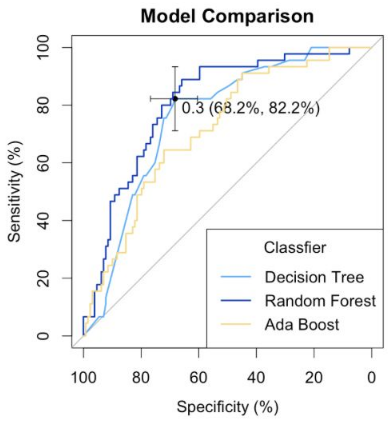

Figure 3: ROC plots for the models trained over elementary train data + ALSFRS statistics + Vital statistics

Then the 6 features were selected according to the feature importance measures and relative influence measures given by the randomForest and gbm packages. The 3 models were trained (with 500 trees for random forest and adaboost) this time. With time constraints, I was only able to check over two 6 feature combinations.

(1) "mean_alsfrs_rate1","sd_alsfrs_r1","mean_alsfrs_r_rate2","sd_alsfrs_hands_rate2", "mean_weight_1" , "treatment_start" ,"mean_bp_systolic_rate3"

(2) age + sd_alsfrs_r1+mean_alsfrs_r_rate2+ sd_alsfrs_hands_rate2 + mean_weight_1 + treatment_start + mean_bp_systolic_rate3 + sd_pulse_rate3

method	auc	threshold	sensitivity	specificity
Decision Tree	66.270	0.444	64.444	68.217
Random Forest	68.148	0.060	91.111	42.636
ADA Boost	64.031	0.325	86.667	40.310

method	auc	threshold	sensitivity	specificity
Decision Tree	68.424	0.191	80.000	55.814
Random Forest	70.370	0.409	60.000	76.744
ADA Boost	69.905	0.376	77.778	58.915

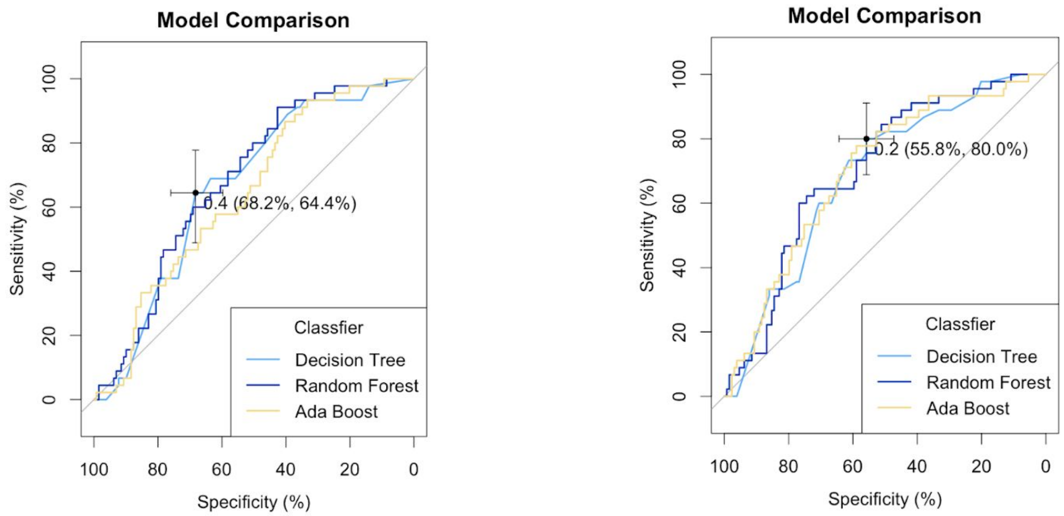

(1) (2)

Figure 4: ROC plots for the models in Fig. 3 trained with two 6 feature combinations

My final submission for the challenge was made with the random forest model of 500 trees, which was trained on the feature combination (1). In order to obtain the probabilities of survival for all the three time periods, a separate random forest was trained for learning the survival at each period. In case of the missing outputs due to subjects leaving the study before the end of 24 months, the survival status was imputed using the same kNN algorithm.

Discussion

In overall, the results illustrate that the prediction accuracy of Random Forest which is an ensemble of decision trees tends to be better than a single decision tree. However, boosting is not at the favourable side for this task. Also it can be seen that the statistics over time series can be used to improve the model accuracy of ALS survival prediction. However, more statistical tests need to be performed in terms of ROC measurements in order to obtain statistically significant results. Moreover, the approach does not perform any clustering for incorporating other major attributes such as adverse events and lab tests in model learning and thus, there is more to be studied; especially the use of hierarchical clustering for the purpose of finding patient groups. In conclusion, the best results of this approach came from learning a random forest model with 100 trees over elementary data + ALSFRS Total score statistics (age + gender + race + family_ALS_hist + treatment + treatment_start + diag_delta + onset_delta + onset_site + if_use_Riluzole + riluzole_delta+ mean_alsfrs + sd_alsfrs + mean_alsfrs_rate + sd_alsfrs_rate) with 80% sensitivity and 76.744% specificity, to predict overall survival.

References

[1] Xavier Robin, Natacha Turck, Alexandre Hainard, Natalia Tiberti, Frédérique Lisacek, Jean-Charles Sanchez and Markus Müller (2011). pROC: an open-source package for R and S+ to analyze and compare ROC curves. BMC Bioinformatics, 12, p. 77. DOI: 10.1186/1471-2105-12-77.

Authors Statement

The data preprocessing, model preparation and evaluation were done using R 3.2.0 version with RStudio on a personal machine as well as using the provided IBM computational resource for the challenge. The mentioned data mining work and this method write-up preparation were solely done by D.N Sumanaweera.

Acknowledgements

Dr. A. Shehan Perera, Department of Computer Science and Engineering, University of Moratuwa, Sri Lanka

Team openneuron: Model using Bayesian Additive Regression Trees for predicting ALS slope and survival

Xiang Yu[1]

1. University of Pennsylvania

Abstract

Model based on Bayesian Additive Regression Trees (BART) were used for predicting ALS slope and overall survival. For predicting ALS slope of PROACT dataset, the data set was performed unsupervised cluster using Clustering for Large Applications.

Introduction

The neurodegenerative disease Amyotrophic lateral sclerosis (ALS) is heterogeneous across the patient population. Therefore, stratification of ALS patients into subgroups is important for developing better drug for patients in specific subgroups. The Amyotrophic Lateral Sclerosis Functional Rating Scale (ALSFRS) is a method to measure the functional status of patients with ALS over time. Survival in ALS is time until either death or tracheostomy. The prediction of ALS slope and Survival are both important for ALS diagnosis.

Method

Data preprocessing

PROACT dataset

(1) Totally the PROACT dataset have 12 different forms (Table 1), and the first 10 forms were used for further analysis.
(2) For feature of each ID that have more than 1 time points, the feature value of feature with last feature_delta less than 92 days were used.
(3) For predicting ALS slope in sub1, features in those 10 forms containing more than 1000 ID with missing value were filtered. Totally, 72 features were retained and were performed imputation of missing data using the approach of Multivariate imputation by chained equations (MICE) [1]. For predicting survival in sub2, features in those 10 forms containing more than 2000 ID with missing value were filtered.

National Registries dataset

(1) Totally the National Registries dataset have 68 features. For feature of each ID that have more than 1 time points, the feature value of feature with last feature_delta less than 92 days were used.
(2) Features containing more than 100 ID with missing value were filtered.

imputation and cluster

For predicting ALS slope of PROACT dataset (sub1), numeric features without missing data after imputation were performed unsupervised cluster using CLARA (Clustering for Large Applications) with k =2 [2].

Feature selection in each cluster

For predicting ALS slope of PROACT dataset (sub 1), data subset in each cluster were performed feature selection using Recursive Feature Elimination (RFE) with random forests model [3]. The top 6 features were selected for each cluster.
For Sub2-Sub4, the top important features were selected by function in bartMachine [4].

Model building

Model for prediction ALS slope of PROACT dataset (sub 1) in two subgroups were built using Bayesian Additive Regression Trees (BART), respectively. The 6 features of the cluster 1 are "onset_delta", "ALSFRS Total", "fvc percent", "trunk", "Creatinine" and "Q1 Speech", while features of cluster 2 are "onset delta", "ALSFRS Total", "fvc percent", "Q1 Speech", "mouth" and "trunk".
For Sub 2, the 6 features for model building are "Potassium", "Bilirubin..Total.", "treatment group", "if use Riluzole", "Age" and "Gender".
For Sub 3, the 6 features for model building are "onset delta", "onset_site", "ALS Staging Breath", "R3 Respiratory Insufficiency", "Q8 Walking" and "Q4 Handwriting".
For Sub 4, the 6 features for model building are "onset delta", "Age", "Gender", "onset site", "Q7 Turning in Bed" and "R3 Respiratory Insufficiency".
All the model are built using the Bayesian Additive Regression Trees (BART) without imputation. **For Sub 2 and Sub 4, the model predict the probability of staying alive.**

Model predictor

For Sub. 1, each test individual were merged with training dataset, and performed CLARA without imputation for predicting the cluster class of test individual. Based on the cluster class, 6 features were selected from test individual and the ALS slope were predicted based on the corresponding BART model.
For Sub 2-4. the selected features of each test individual were extracted for prediction using corresponding BART model.

Discussion

For Sub1, cross validation shows the average Root-mean-square deviation (RMSE) and person correlation (PC) of model with features selected for cluster 1 were 0.5651788 and 0.4339461, respectively; RMSE and PC of cluster 2 were 0.5659873 and ~0.43, respectively. For Sub2, the mean RMSE is 188.6991, while the mean PC are 0.3285618.
For Sub3, cross validation shows the mean RMSE is 0.7151283, and mean PC is 0.4888758. For Sub 4, the mean RMSE is 648.8162 while the mean PC are 0.6812798.

Author statement

The submission will be made public.

Reference

[1] Stef van Buuren, Karin Groothuis-Oudshoorn (2011). mice: Multivariate Imputation by Chained Equations in R. Journal of Statistical Software, 45(3), 1-67. URL http://www.jstatsoft.org/v45/i03/.
[2] Wei, Chih-Ping, Yen-Hsien Lee, and Che-Ming Hsu. "Empirical comparison of fast clustering algorithms for large data sets." System Sciences, 2000. Proceedings of the 33rd Annual Hawaii International Conference on. IEEE, 2000.
[3] http://topepo.github.io/caret/rfe.html
[4] Kapelner, Adam, and Justin Bleich. "bartMachine: Machine Learning with Bayesian Additive Regression Trees." arXiv preprint arXiv:1312.2171 (2013).

Team Davide Chicco: A k-means clustering method for prediction of ALS slope progression and survival

Davide Chicco
Princess Margaret Cancer Centre, University of Toronto
davide.chicco(AT)gmail.com

- Will you be able to make your submission public as part of the challenge archive? Yes

Abstract Our methods utilizes a k-means clustering technique to group together the patients features and select the six most "important" ones. In the selector script, once the patients' features are grouped into clusters, our approach select the 6 clusters that are nearest to the k-means centers. In the predictor phase, our algorithm selects the values of the patients features having the same form name, feature name, and feature value of the selected features, and then computes the average among their (slope or survival) values. This value will be the predicted ALS progression slope or the survival of the input patient.

Background/Intro

We implemented a k-means [1] [2] [3] clustering method that can be able to group together the input features based on their position in the hyper-space. Since the input features are categorical data and not numerical data, we first map them into integer values, and then compute the distances between rows through a k-means technique.
We know our approach suffers from a big problem: the mapping of the feature names into number introduces some geometrical information that do not reflect the real semantic distances between feature concepts. We are aware of this big flaw, but we think that this method could quite effective in recognizing similar feature rows that show similar values.

Methods

[Subchallenge 1 ProActProgression and SubChallenge 3 NatRegProgression]

[selector] In the selector phase, our script first read the input patient data and the training data for all the patients. All the feature names of these data are associated to natural numbers, to be elaborated by the k-means method.
We compute the number of clusters (k) as the square root of the dimension of the input data divided by two.
Once applied the k-means method, we save up the cluster that is more present among the data (majority_cluster): this will be the final selected cluster. We then select the 6 rows of the input data which are closer to the k-means centers. We save the 6 indices of these input rows.
Finally, we select the feature names associated to these 6 indices, that will be the final selected features.
The selector script returns these 6 selected features and the majority cluster.
[predictor] In the predictor script, we read the selected cluster and the selected 6 clusters, and the patient gold standard file data. From the patient golden standard, we retrieve all the patients ("ids") that have the same form_name, feature_value and feature_name of the selected 6 features.
We then read the slope gold standard dataset, and we retrieve all the slope values of the retrieved patients "ids". Once we have these slope values, we compute the average among then and return it as the predicted progression slope.
[Subchallenge 2 ProActSurvival and SubChallenge 4 NatRegSurvival]
[selector] For the survival prediction, the selector script behaves the same way of the progression slope pre
[predictor] In the predictor script, we read the selected cluster and the selected 6 clusters, and the patient gold standard file data. From the patient golden standard, we retrieve all the patients ("ids") that have the same form_name, feature_value and feature_name of the selected 6 features.
We then read the survival gold standard dataset, and we retrieve all the survival values of the retrieved patients "ids". Once we have these survival values, we sever the survival values of the patients that are dead (status==1) and alive (status==0).
We then select the patients that have a time_event lower than 12 months, 18 months and 24 months and sever them between the dead patients and the alive patients.
Once we have this six categories (months12_patients_Dead, months12_patients_Alive, months18_patients_Dead, months18_patients_Alive, months24_patients_Dead, months24_patients_Alive). We then compute the likelihood of survival of a patient by computing the number of alive patients on the total of patients (alive ones + dead ones), for 12 months, 18 months and 24 months.
We then return these three values as final output of the predictor script.
The final generated resulted values of these models represent the likelihood of survival of the input patients.

Conclusion/Discussion

Our approach is based upon the k-means clustering of the patient data followed by the retrieval of the progression and survival values of similar patients present in the gold standard data. Once these values of the "similar" patients are retrieved, we return an average of these values as final output.
As I already mentioned, our k-means approach suffers from a big conceptual flaw: the geometrical mapping of the feature names do not respect any semantic distances. For this reason, we expect our

method to work well for the patients' data that are in similar position in the input dataset, while to perform badly for the patients' data that are very far in the input dataset.
For the next time, we should study how to proper associated the numerical values to the feature names, in order to make them respect a semantic distance between concepts.

References

1. James MacQueen. "Some methods for classification and analysis of multivariate observations." Proceedings of the fifth Berkeley symposium on mathematical statistics and probability. Vol. 1. No. 14. 1967.
2. John A. Hartigan, and Manchek A. Wong. "Algorithm AS 136: A k-means clustering algorithm." Applied statistics (1979): 100-108.
3. k-means, R package. https://stat.ethz.ch/R-manual/R-devel/library/stats/html/kmeans.html

Authors Statement

The method was entirely designed and implemented by Davide Chicco

Re-execute tests

Regarding my code files that I uploaded in the "files" section, I confirm that they do not contain any data. Any interested user who is interested in re-running the tests with my code (after registering and obtaining the Challenge Data from the ProAct organization), can read the README files contained among my code package.

Team zsh-2015: A random forests model for predicting survival of ALS patients using PRO-ACT database

Wenwen Min[1], Wenkai Ji[2] and Shihua zhang[2]*
1School of Computer, Wuhan University, Wuhan 430072, China
2National Center for Mathematics and Interdisciplinary Sciences, Academy of Mathematics and Systems Science, Chinese Academy of Sciences, Beijing 100190, China
Email: zsh@amss.ac.cn

Summary

We adopt a random forests model to select six variables, predict the probability of death within a 0-12 months, 0-18 and 0-24 months from trial onset for ALS patients.

Introduction

Amyotrophic lateral sclerosis (ALS) is a rapidly progressing neurodegenerative disease that typically leads to death within 3-5 years [1]. The PRO-ACT database contains clinical data for over 7,500 ALS patients from completed clinical trials. The entire PRO-ACT database can be available for research purposes since December 2012. The ALS Prediction Prize will use a subset of this data . The goal of this challenge is to predict the progression of disease of ALS patients based on the patient's current disease status.
Random forests [2] is an ensemble machine learning algorithm for classification and regression. Here, we adopt a random forests model for predicting survival. We put all_forms_PROACT data as training data. We obtain an R package "randomForest" to model this data and chosen six variables (Age, weight, fvc, ALSFRS_Total, fvc_percent, onset_delta) based on the "importance" function. Among the six variables, two variables (Age and onset_delta) are static ones and the other are time-series ones.

Data preprocessing

We carefully check the data types and find many data types are very noisy and incomplete. Thus, we consider the nine data types as follows:
1.Demographic (3 features)
2.ALS history (ALSHX) (3 features)

3.Riluzole (1 features)
4.Treatment (1 features)
5.Family History of ALS (FamilyHx) (1 features)
6.ALS functional rating scale (ALSFRS) (23 features)
7.Vitals (8 features)
8.Forced Vital Capacity (FVC) (3 features)
9.Slow Vital Capacity SVC (3 features)
We preprocess the data and get static variables and time-series variables (Figure 1A). We adopt five summary statistics (as features) to characterize each time-series variable, including min, max, mean and MaxMinslope (slope) (Figure 1A). For example, we take the time-series variable weight as an example (Figure 1B). Accordingly, we compute the five summary statistics: min.weight (80 kg), max.weight (89 kg), mean.weight (85.5 kg), and slope (89-80)/[12*(9-100)/365.24]=-3.01, respectively.

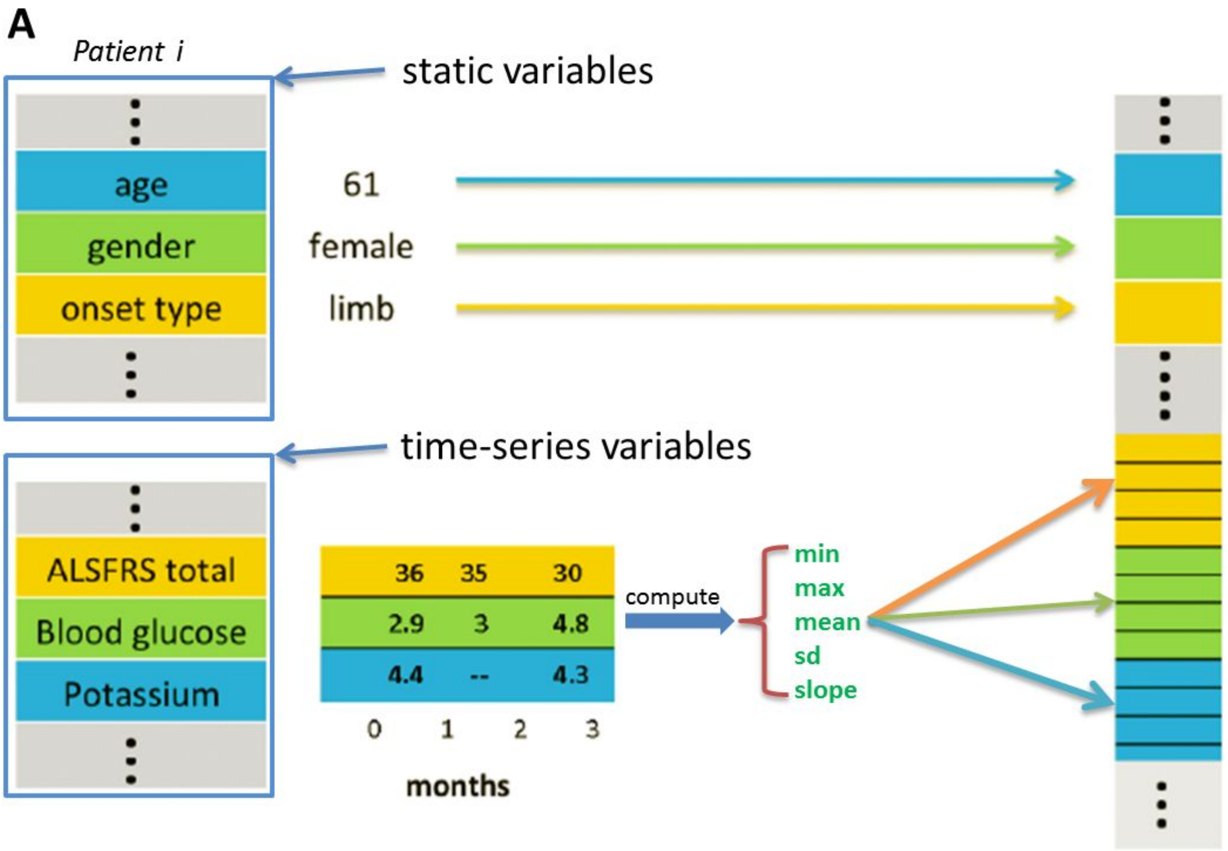

B

SubjectID	form_name	feature_name	feature_value	feature_unit	feature_delta
992259	Vitals	weight	89	kg	9
992259	Vitals	weight	88	kg	24
992259	Vitals	weight	85	kg	50
992259	Vitals	weight	80	kg	100

Fig.1 Illustration of data processing for each patient.
We process the PRO_ACT_data and get a dataset with 174 features. The data contains 9 static variables and 33 time-series variables.

For the features that have very few missing data, we try two alternative methods to impute the missing data: (1) use the "impute" R package, which implemented the k-nearest neighbor algorithm (knn) method; (2) simply use the mean of considered features. We find that these two methods only have little effect on the final results. Thus, we simply adopt the second method to impute the missing data.
We take 20% as the threshold. If less than 20% of patients for one feature, then we remove it (Figure 2). We remove 17 features. Finally, we obtain a data matrix with 157 features and 7067 patients.

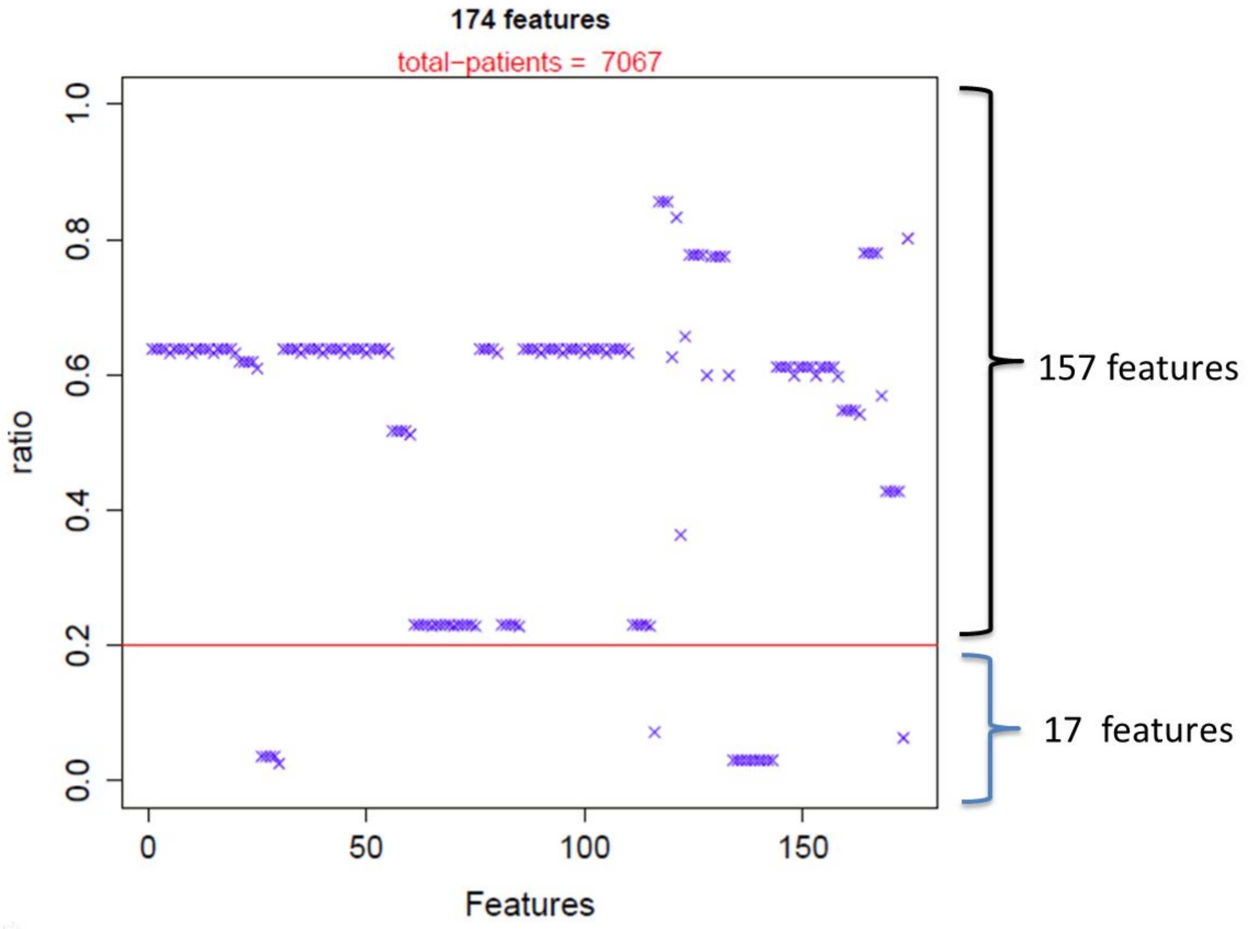

Fig. 2 Missing ratio of features. The red line indicates the threshold we used.

Model

There are many machine learning methods for classifying binary clinical outcomes. However, it is a challenge for dealing with censored survival data. For predicting the probability of death within a 0-12 months, 0-18 and 0-24 months from trial onset for ALS patients, we first put censored survival data into binary clinical outcomes. We dichotomized the censored continuous survival data through some cutoff time points (e.g., 12 months, 18 and 24 months). For each considered time point (12 months, 18 and 24 months), we can get a series new binary clinical outcomes based on ALS patients' tiem_event and status.
In the Subchallenge 2, we use the all_forms_PROACT data as a training set, and the leaderboard data as a validation set. We adopt a standard random forests classifier model [2]. We use the "randomForest" R package to implement the algorithm to train our model and predict the survival. Forests are created with at least 500 trees and otherwise default settings.

Feature section

We dichotomize the censored continuous survival data through some cutoff time 18 months for all_forms_PROACT data. The model is fitted to the 7076 training patients (all_forms_PROACT data). We first train a random forests classifier with the training data and then list the top 30 features (Figure 3) based on the “varImpPlot” function in the RandomForest R package. We extract the most important six variables (Age, weight, fvc, ALSFRS_Total, fvc_percent, onset_delta). Note that, in the 6 variables, two variables (Age and onset_delta) are static-variable, the other are time-series variables. So we can get 22 features (2 static features and 20 statistics).

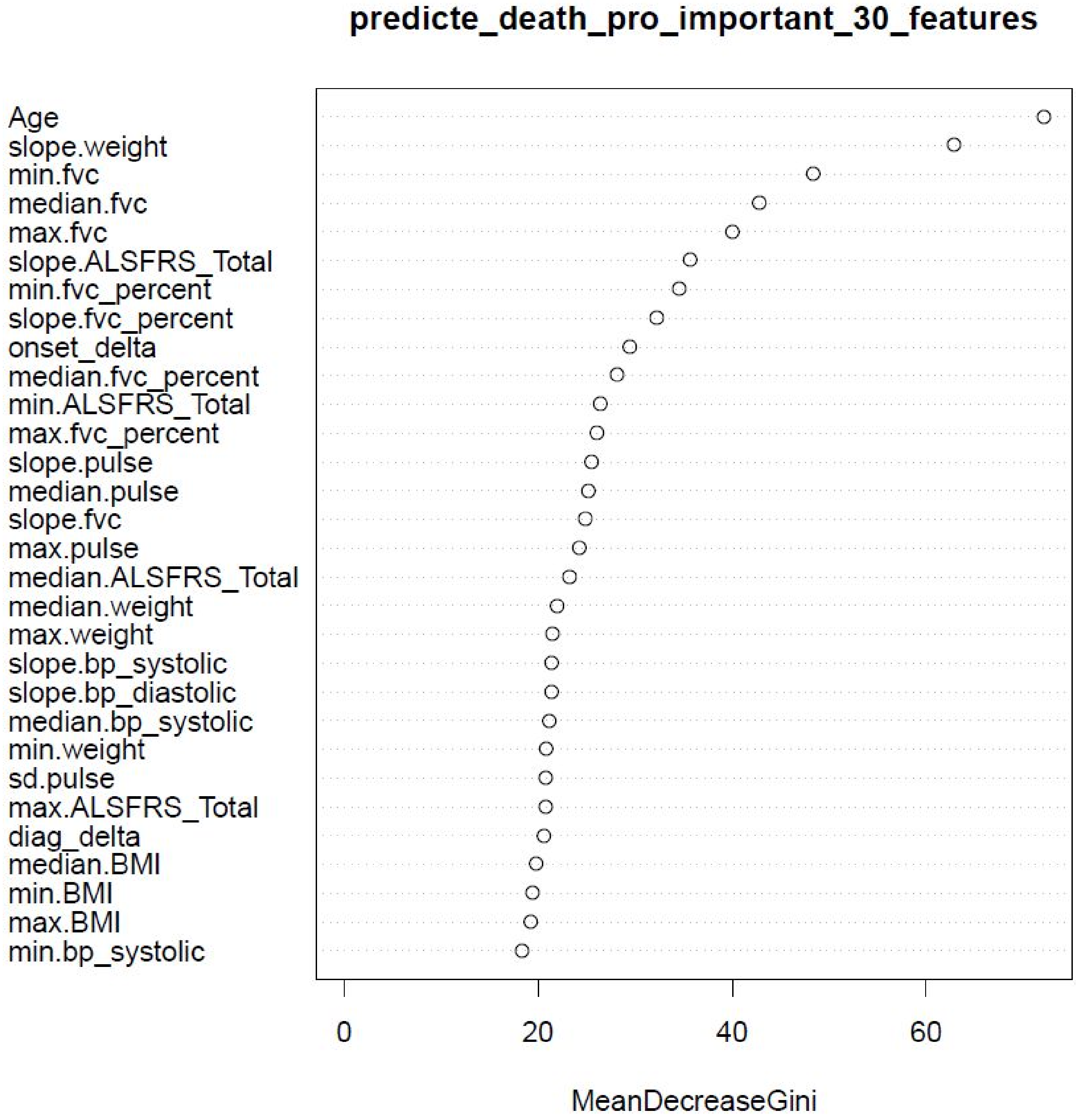

Fig. 3 The top 30 important features.

Discussion

We also consider to divide the training samples into different classes and then predict the slopes in the validation data. However, we achieve the best performance with respect to concordance index (CI) without classification. Here, we employ a standard machine learning algorithm random forests to predict the survival of ALS.

Author Statement

W.M., W.J. and S.Z. conceived the study. W.M. and W.J. purified the datasets. W.M., W.J. and S.Z. contributed to establishing and refining the prediction model. S.Z. supervised the analysis. W.M. and S.Z. wrote the manuscript. W.M., W.J. and S.Z. proofread the manuscript.

Reference

[1] Küffner R, Zach N, Norel R, et al. Crowdsourced analysis of clinical trial data to predict amyotrophic lateral sclerosis progression. Nature biotechnology, 2015, 33(1): 51-57.
[2] Breiman L. Random Forests. Machine Learning, 2011, 45(1):5-32

DREAM ALS Stratification Prize4Life Challenge: Team ALS-Theodore

Authors: Xihui Lin[1], Dorota Sendorek[1], Christopher Lalansingh [1]
Affiliations:
[1]Informatics and Biocomputing Program,Ontario Institute for Cancer Research (OICR), Toronto, Canada

Data Processing

The ProAct data was processed by forms.

1. Data collected after 3 month were all dropped.
2. The concomitant medication form were discarded as the features either have too many missing or does not show any significance by univariate ANOVA test using ALSFRS slope as response.
3. Adversed event were considered, only 8 features were selected as they were significant in univariate ANOVA test to ALSFRS slope.
4. We summarized ALSFRS_Total by its minimum, maximum, average and last observation. While other features with multiple visits were summarized as average.
5. Missing values were imputed by its median if continuous and mode if categorical.

Methods

Sub-challenge 1:

1. Random forest was our final model, used for feature selection and modeling.
2. No clustering was done, i.e., all patients were in cluster 1.
3. The six features will finally came up with by variable importance were
    - onset time
    - minimum ALSFRS in the first 3 months

- FVC percent
- onset site
- pulse
- phosphros

1. Hyper-parameters `mtry` and bootstrap sample size `sampsize` in the R's `randomForest` function were tuned by cross validation to minimize the root mean square error (RMSE). In the final model, `mtry = 2` and `sampsize = 100`.
2. The confidence field in the output were all fixed to 0.5 as we did not do an re-sampling.

Sub-challenge 2:

1. Gradient boosted Cox model (*GBM* package in R), were used for both feature selection (using variable influence index similar to variable importance in random forest) and final modeling.
2. Again, no clustering was done and thus all patients were assigned to cluster 1.
3. The most predictive features for patient survival by variable influence index from *GBM* were
   - last observation of ALSFRS total in the first 3 months
   - age
   - FVC percent
   - onset time
   - bicarbonate
   - chloride

1. The `shrinkage` parameter was fixed to 0.001, tree depth were fixed at 3 (indeed, 1-5 were tried) and number of boosting trees were tuned by 3 fold cross validation to minimize Cox's partial likelihood.
2. The output probabilities in our model are probabilities of death.

Conclusion/Discussion

1. A total of 8 adverse event features were kept after preprocessing, but none of them were finally chosen by models either in predicting progression or survival.
2. For sub-challenge 1, *onset delta* was the most important features by random forest variable importance index. We also started the model using the selected six features and added other top features (ranked by variable importance) to the model in sub-challenge 1, but none of them seems to contribute to the predictability. Thus we conclude that the six features we chose are sufficient for predicting progression, and clustering may not be a necessary for improving predictability.

Code

1. To run the code for sub-challenge 1, one needs to put `sub1.R`, `sub1_data_cleaning_new.R` in the some directory as the data. Then run `sub1.R` to generate an random forest model which will be saved as `sub1_rf_0908.RData`. Then copy this file to the IBM server, and run the script on the IBM server.
2. To run the code for sub-challenge 2 is similar to the above, except that one will use `sub2.R` instead and it generated `sub2_GBM.RData` for prediction.

Acknowledgements

The authors thank all members of the Boutros Lab for their help and support.

ML-BGU: A layered random forest approach for clustering ALS patients and predicting disease progression

Jonathan Gordon, Boaz Lerner, Noa Ben David, Michael Kozak, Eyal Ben Zion
Department of Industrial Engineering and Management, Ben Gurion University, Israel

- We are willing to make our submission public

Introduction

Our method 1) utilizes random forest models to find low dimensional representations of the patients based on the predicted slopes for the different aspects of the disease; 2) clusters the patients in this lower dimension feature space; 3) uses non-parametric predictor importance evaluators to associate an ordered list of features by importance to each cluster; 4) constructs a random forest model that predicts a new patients progression slope using only the most important features from his cluster that were documented for him.

The main approach of our method was to find some meaningful low dimensional representation of the patients around which we could cluster them into meaningful groups. To do this, we trained a set of five random forests. Each forest represented a mapping from the initial high dimensional feature space (the raw data) in the first three months in the database, to a slope for one of the groups of ALSFRS functions (represented in the database as mouth, hands, trunk, leg, respiratory) in months 4-12. This feature space is highly meaningful, as it encodes the high dimensional data in a supervised manner that is directly connected to the response variable. We then learned a clustering schema around this 5 dimensional feature representation of each patient using the K-means algorithm. Following that we utilized non-parametric feature importance metrics to associate each such cluster with and ordered list of the features from the raw data. For a new patient, first the random forests were used to map his raw features to the low dimensional representation, and then he was assigned to one of the clusters (based on the minimum euclidean distance to the cluster centroids). Finally, the most important features from his cluster that were documented for that patient were extracted, and a cluster specific random forest model is trained and used to predict his final slope. Final slope prediction is done via training separate random forests to predict the slope for each of the ten ALSFRS variables, and then summing the predictions for the final slope prediction.

The benefits of using our model are two-fold:

1. The meaningful low representation of the data allows us to find important and easily interpretable clusters. The cluster centroids can be seen as distinct disease progression patterns. Not only do the different centroids represent fast, slow and moderate progressors, but they also show different patterns of disease progression (e.g. one cluster may have moderate progressors whose progression is highly emphasized in the hands and legs, while another may have moderate progressors where progression is quicker in the trunk and mouth).
2. By predicting each of the ten ALSFRS functions separately and then summing them into a final prediction, predictive performance is improved.

The novelty in our model is mainly based on the supervised dimension reduction of the data, i.e. the mapping from the entire feature space to the five-dimensional representation of the different predicted disease slopes. We believe that clustering around this feature space may hold potential beyond the scope of disease progression prediction: it seems that the clustering algorithm identifies a number of distinct predicted disease progression patterns. We can furthermore use only the data from the first three months of a patient's clinical trial to classify him to one of these patterns, which could prove to be tremendously beneficial for care-givers of ALS patients.

Methods

Feature Selection

Our initial goal was to identify a finite number of features which we would be used for the remainder of the model. Two main criteria were selected by which to choose features:

1. Availability - we did not want to impute too much data, and therefore decided to work only with features which were available for above a threshold value of patients (60%).
2. Importance - we searched for features which showed potential importance in predicting the response variable. To this end we trained a large number of linear regression, CART and random forest models which predicted the different slopes of the patients in the training data. We then analyzed the importance of the different variables in these models using standard predictor importance metrics (mostly based on the caret R package).

After this analysis, we were left with the following feature set with which we continued to the modelling:
Chloride, Alkaline phosphatase, CK, basophils, hemoglobin, potassium, lymphocytes, phosphorus, albumin, creatinine, bp diastolic, bp systolic, pulse, respiratory rate, weight, fvc percent, age, race, onset delta, onset site, if use Riluzole, treatment group and all of the ALSFRS functions and groupings.

Feature extraction

We differentiate between the handling of static and dynamic variables (i.e. time series and non-time series variables). All static variables were introduced into the feature matrix as is. For time series data, we extracted for each variable the following statistical moments and features (using only data from the first three months): max, min, last, mean, sum, slope, sum of mean square, standard deviation and some indicators for number of values per variable. Thus, our feature matrix consisted of a total of 380 features.

Missing data

We decided to impute missing data in the initial feature matrix using the supervised data imputation method in the random forest package in R. For this, we retained in the feature matrix only patients who had a recording of the response variable (2,712 patients all together). Having imputed the missing data in the feature matrix, we constructed a vector of the median and mode values (for numerical and categorical features respectively). This would allow us to impute the missing values for patients from the test set in the selector and predictor programs when necessary.

Splitting the database

As shall soon be discussed, our model contains two main layers in it. We tested empirically (using a CV-10 methodology) and found that when the two layers were trained on separate and independent databases, the model outperformed a model where both layers were trained using the same (complete) data set. Thus, we split the training data set into two subsets, one for the training of each layer. We found the optimal size for each training set empirically, testing the performance of the entire model with different distributions of the training set among the two layers. Eventually, the first layer received 700, and the second layer received the remaining 2000 patients.

First Layer - dimension reduction and clustering

In this layer, which is part of the offline training of the model, we use the first training set to train the following components:

1. A set of five random forests which map from our initial feature matrix to the five predicted slopes for the patients.
2. A clustering schema which is comprised of k=4 five dimensional centroids, pertaining to the five predicted slopes.

Training the random forests for the dimension reduction is straight-forward: we simply use the random forest function in the package to train forests mapping from the complete high dimensional feature representation of the patients in the first training set (as stored in our initial feature matrix) to each of the five slopes depicted (optimal values for the hyper-parameters were determined using a CV-10 methodology). We then set aside this set of random forests for later use.

In order to achieve an unbiased matrix containing the predicted slopes of the first training set, we constructed an inner-loop which sets aside 10% of the patients in the first training set, train random forests with the remaining 90%, and use them to predict the slopes of the 10%. In this fashion we construct a matrix containing the unbiased predictions of the slopes for the patients in the first training set. Finally, we run a K-means algorithm over this matrix and store the cluster centroids. We then discard the first training set, as to avoid over-fitting of the model.

Cluster	Mouth	Hands	Trunk	Leg	Respiratory	Total (sum)
1	-0.345	-0.103	-0.131	-0.139	-0.056	-0.776
2	-0.051	-0.074	-0.097	-0.083	-0.022	-0.329
3	-0.316	-0.322	-0.323	-0.286	-0.069	-1.317
4	-0.084	-0.223	-0.227	-0.164	-0.050	-0.750

The above table shows the cluster schema learned by the K-means algorithm. Inspecting the cluster centroids, we notice a number of interesting observations; 1) looking at the total slope for each cluster, it seems that the algorithm has found one "fast progressing cluster" (cluster 3), one "slow progressing cluster" (cluster 2), and two "moderate progressing clusters" (clusters 1 and 4); and 2) the two "moderate progressing clusters" differ from each other in the pattern of progression, where in cluster 1 progression seems to be more related to mouth and leg deterioration, whereas cluster 4 seems to be oriented around hands and trunk deterioration.

Second Layer - cluster specific predictor importance and training of the models

In this layer, which is the final stage of the offline preparation of the model, we wish to associate each cluster with a list of the potential features, and order that list according to some measure of feature importance. We do this using the second training set so as to avoid over-fitting of the model. The first task is to assign each patient in the second training set to a cluster. To this end, we use the set of random forests trained in the first layer to predict the five slopes for each of the patients in the set. We then assign each patient to a cluster based on minimum euclidean distance between his feature representation (the five predicted slopes) to the centroids of the different clusters.

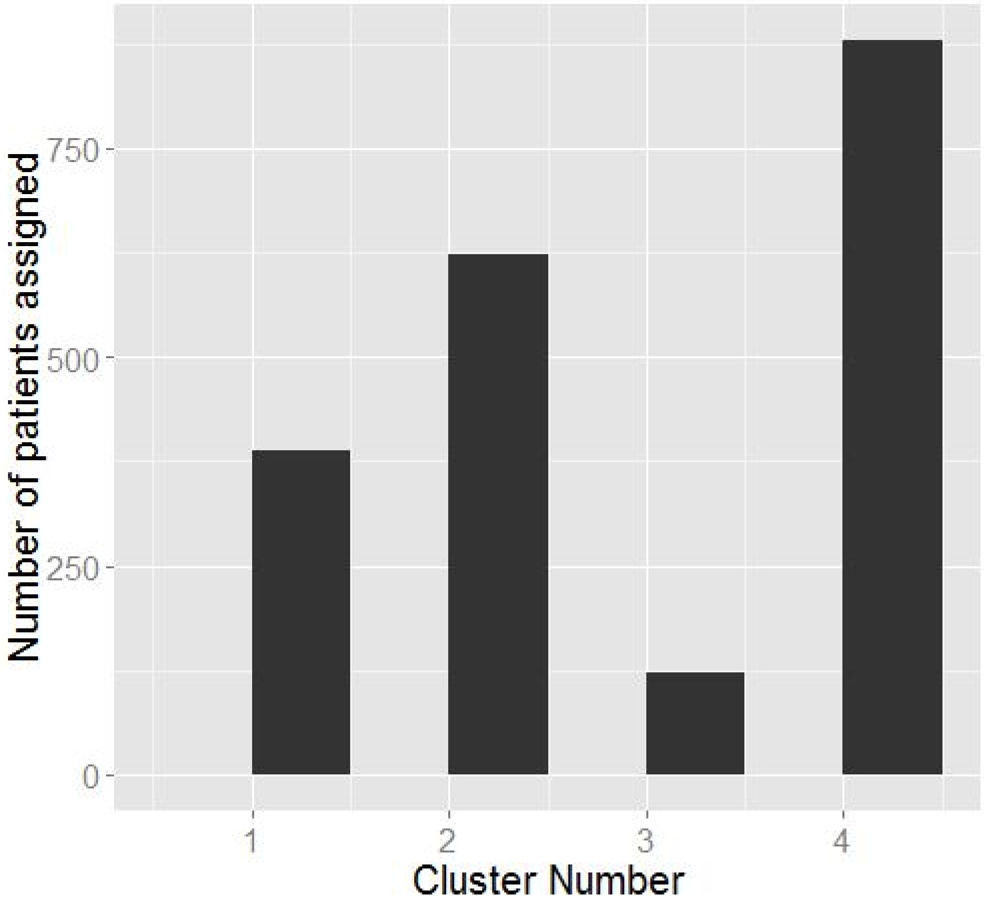

We can see from the above figure that cluster 4 (moderate hands and trunk progressors) is by four the most populated by the patients in the second training set. Furthermore, we can see that cluster 3 (fast progressors) is much less populated. We believe that learning important feature specific to the different patterns of progression greatly improves the accuracy of our model.

Having assigned each patient to a cluster, we learn a random forest model mapping from the entire feature representation of patients (separately for each cluster) to the final response variable. We then use the caret package to assign predictor importance to each feature. Since some of the variables are time-series (and thus represented by a number of features), it is necessary to aggregate the predictor importance for each variables over all of the representative features in the model. We choose this aggregation to be the maximum predictor importance for each variable. We then can discard the current set of cluster-specific random forests, and retain only the variable importance list achieved.

Cluster 1	Cluster 2	Cluster 3	Cluster 4	
onset_delta	onset_delta	respiratory	onset_delta	
Q4_Handwriting	weight	Q3_Swallowing	fvc_percent	
Alkaline.phosphatase	Alkaline.phosphatas e	fvc_percent	leg	

ALSFRS_Total	fvc_percent	weight	ALSFRS_Total	
fvc_percent	leg	Alkaline.phosphatase	bp_diastolic	
bp_systolic	Hemoglobin	Lymphocytes	trunk	
Q6_Dressing_and_Hygiene	bp_diastolic	mouth	Alkaline.Phosphatase	
Potassium	Lymphocytes	respiratory_rate	Q9_Climbing_Stairs	
trunk	Chloride	Hemoglobin	Q5_Cutting	
mouth	Basophils	Q7_Turning_in_Bed	Hemoglobin	

The above table details the ten most important features (as found by our model) for each cluster. We can see that onset_delta plays an important role for three out of the four clusters, and FVC in all of them. Barring those two, important features seem to differ between the clusters. We believe this is one of the key insights of our model.

Selector

This is the first part of the online side of the model. The role of the selector is to first assign the new patient to a cluster, and to filter the six features for the new patient which are most important to his cluster and documented for him. As such, the selector first uses the set of random forests to map the patient from the high level representation using all of his available features, to the five dimensional representation of the predicted slopes. It then assigns the patient to one of the 4 clusters, based on the minimum euclidean distance from the patient's feature representation to the cluster centroids. Having assigned the patient to a cluster, the selector turns to the relevant ordered feature list (associated with the patient's cluster). It then extracts the raw values for these features in months 1-3, and writes them to the designated path.

Predictor

This is the final stage, and also happens online. The role of the predictor is to output the final slope prediction for the patient, alongside a confidence measure. It does this in a number of stages: first, it extracts all of the relevant features from the six variables it received from the selector (i.e. statistical moments and indicators from the first three months, similar to the initial feature extraction). It then trains ten random forest models (one to predict the slope of each of the ten ALSFRS variables) using the patients from the second training set, with only the features selected by the selector, and the cluster ID. Finally, it predicts ten slopes for the new patient, and sums them to achieve the final prediction.

The final task for the predictor is to determine a confidence measure in his prediction. We define confidence as follows:

$$\text{Confidence}(\hat{y}\ )=1-V(Y|X)V(Y)$$

where $V(Y)$^ is the standard deviation of the slopes seen in the training set, and $V(Y|X)$^ is the standard deviation of the predictions of the trees in the forest. Thus, if the standard deviation among the trees in the forest is much lower than the standard deviation of the slopes from the training set, we can say that the confidence in the prediction is greater.

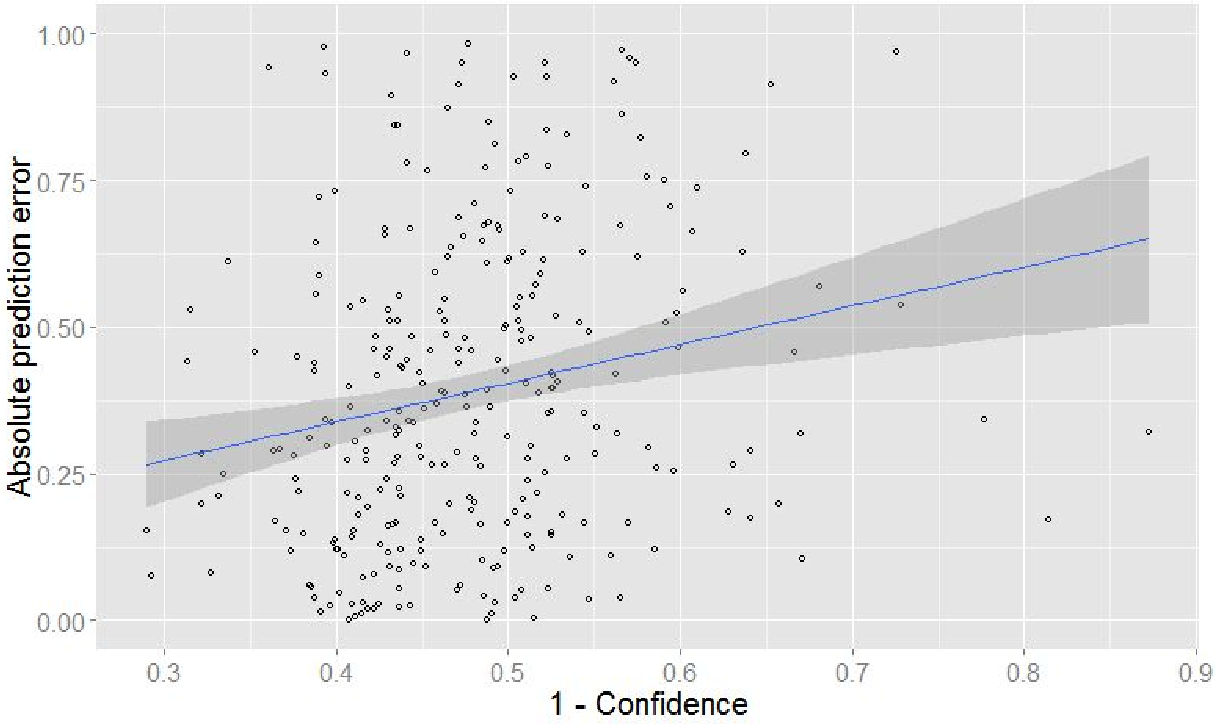

Compiling the source code

Running our source code is very simple. You must first load the RData file provided, named "OnlineWorkSpace". It is necessary that the R packages "randomForest" and "dplyr" be installed in the environment that the source code is meant to run on. All of the functions, data structures and statistical models necessary to run the model successfully are contained within the work-space provided. Additionally, the source code for the selector.R, predictor.R, selector.sh, and predictor.sh have been uploaded as requested. Once the OnlineWorkSpace has been loaded, it is possible to call selector (which should receive two input arguments that specify the path for the input file to be loaded, and the output file to which selector should write its output), and predictor (which also receives two arguments; the path to an input file identical to what selector outputs, and a path for a file where predictor should write the final output).

Discussion

In this competition, we designed a two-layer model which first performs a supervised dimension reduction to a low dimensional representation of the data which is highly meaningful in respect to the response variable. This dimension-reduction allowed us to cluster the data effectively, where each cluster represents a distinct disease progression pattern. We then associated each cluster with a list of the six most meaningful features available in the database, and trained a final set of random forests to predict the final slope of patients.

One interesting aspect of this model are the easily interpretable distinct disease progression patterns that arise from the clustering component of the system. It is feasible to use this methodology to identify a distinct set of predicted disease progression patterns, and classify patients into one of these patterns at an early stage of disease onset. We believe this mandates further inquiry and could be beneficial to the ALS community at large.

Another interesting aspect was that a significant boost in performance was achieved by 'disconnecting' the two layers of the model with respect to the training sets. In the initial implementation of the model, both layers (i.e. the dimension reduction and clustering, and the final prediction) were trained using the entire training set. Later, we split the data into two mutually exclusive sub-sets, and used a separate one for the training of each layer. However, this emphasized a problem of small data sets, and we believe that the model could still be greatly improved by training it with much larger data sets, should those be at some point available.

Furthermore, due to the definitions of the competition, much useful information is discarded during the model's prediction phases. We believe that prediction could be greatly improved by introducing the low dimensional representations as features for the final prediction phase.

We expect that the model work well when predicting slopes for patients who have the documented values for the most important features used by their cluster. In cases where few or none of the six features are available for the patient, we expect that the model will output a less meaningful prediction. Furthermore, this model requires a lot of training data, due its layered nature. Our own tests have showed that the improvement of the model as an increasing function of the size of the training data it is fed is monotonous, and does not seem to be converging within the sizes of datasets currently contained in the PRO-ACT database. We believe therefore, that this model could still dramatically improve its performance as more data regarding ALS patients is collected.

Team Jyguo: Model ALS disease progression using sigmoid function

John(Yuelong) Guo
RTI International, Research Triangle Park, NC, USA

Introduction

The analyses described below focused only on modeling ALS disease progression using the PRO-ACT database (sub-challenge 1). The goal of this sub-challenge is to predict the rate of ALS disease progression using ALSFRS slope. Modeling with only the slope implicitly assumed that the ALS disease progression follow a linear pattern. However, this assumption may not be ture. Based on patient self-report, PatientsLikeMe collects ALS patient ALSFRS-R score over time (Frost and Massagli, 2008). Evidence shows that ALS may indeed progress with variable rate at different disease status. A first-pass analysis of the challenges data also showed that the fastest progression ALS patient (i.e. of whom the most negative slopes were observed from 3-12 month) are almost exclusively having moderate ALSFRS score at the first three months. This implies a possibility that ALS may indeed progress at a variable rate, and that patient with ALS progress the fastest when the patient has already lost some but not all of their motor function. I hypothesis that ALS disease progression can be modeled better with a sigmoid curve than a linear slope. If ALS patients indeed progress following the implied pattern, explicitly modeling the ALSFRS score progression would 1) better predict the ALSFRS score end point at 12 months, thus the slope, and 2) provide more accurate disease progression prediction at any given time point, thus adds clinical benefit to the patients. Data for more than 5 time points were available in most patients in the PRO-ACT database, which provided an acceptable basis for estimating the underlying sigmoid curve. Unfortunately, there are much fewer time points available for patients in the national registry, therefore the model was not extended to apply to the national registry data.

Methods

I used the following sigmoid model to approximate the implied disease progression pattern:
$(40 - y)/40 = 1 / (1 + \exp(a * t + b))$

where y is the ALSFRS score (range 0-40), t is the time (in days), a and b are two parameters - a models variability of disease progression among patients, and b models the disease progression status at time t=0.

1. training end point estimation

The pre-calculated slope were not used directly as training end point. Instead, two end points, a, and b, were estimated from the data using ALSFRS total score data. Because of the limited number of data points available for each patient, maximum likelihood estimations using numeric approximation method (such as Metropolis–Hastings algorithm) generally does not perform very well. Linear regression was applied instead:

log(40 / (40 - y) - 1) = a * t + b

However, there is the drawback that the BLU estimator minimizes the squared distance of log(40 / (40 - y) - 1) from the predicted, instead the squared distance of y from the predicted. I will discuss more about this in the discussion section.

1. data cleaning

Features in the first three months of study were retained as the training data.
Run python script 00_first_3_months.py should filter the by_subject data by feature_delta.

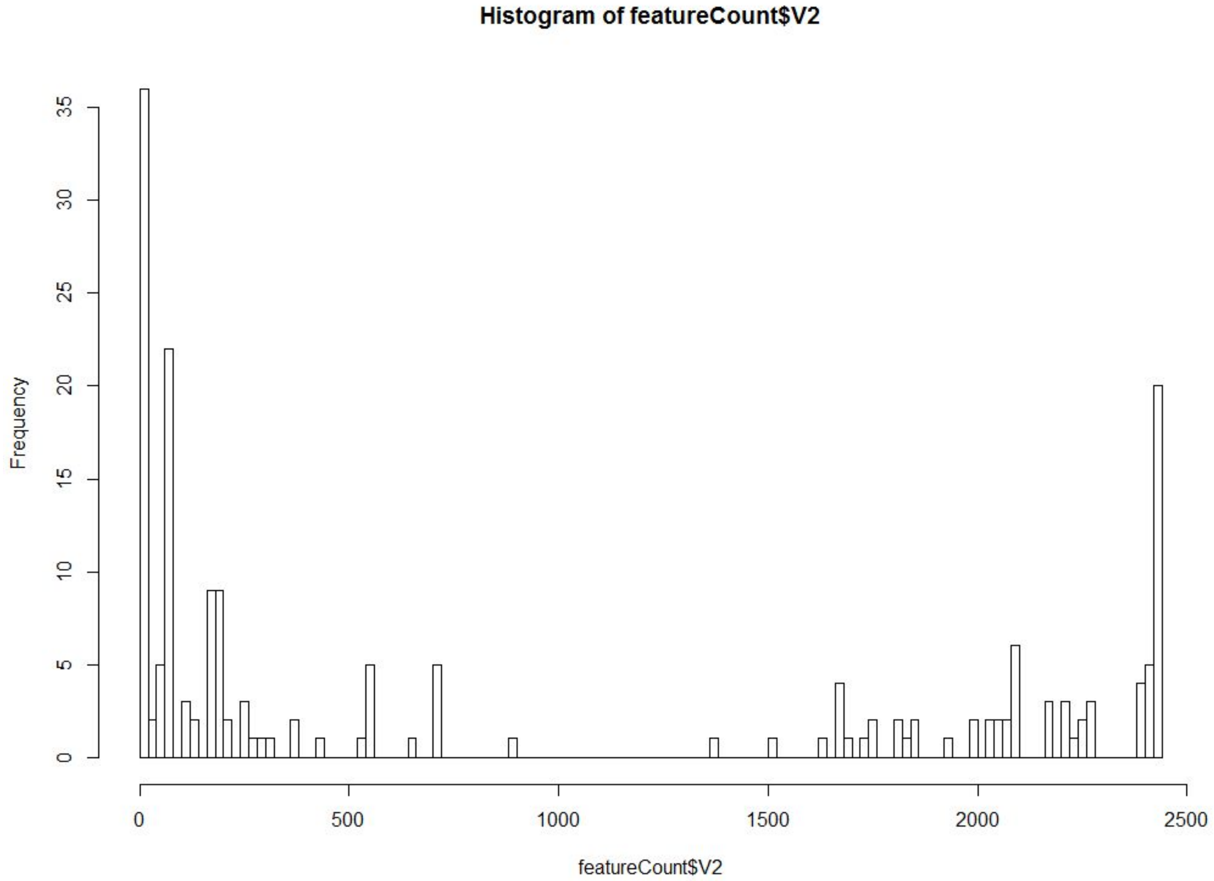

According to the availability of features in patients (above figure), features available in more than 1500 patients were retained for further analyses, the rest were discarded.

Run python script 01_count_features.py should filter feature by this criteria

For each feature, the first, last, min, max, mean, and 3_month_slope of the feature were derived using python script 02_makeTable.py

The derived features were merged into a data table of size 2424 * 391 using python script 03_combineTable.py

Please note that one may need to edit the directory paths in the python script to run the code.

Categorical variables 'if_use_riluzole', 'gender', 'onset_site', 'treatment_group' were converted into numeric variables '0' and '1', and split into two or more variables whenever needed (e.g. onset_site was split into onset_site_Bulbar and onset_site_limb). Categorical variable 'race' were discarded as majority of the study subjects (>95%) were white.

Missing data was imputed with median value of the corresponding feature. No normalization was performed. Feature selection step was run with the non-imputed data.

1. feature selection

Because a total of two parameters were used as training end, and the limitation of 6 features total for estimating both parameters, systematically selecting the best set of Features were selected using a semi-manual way using forward/backward selection of a linear model. The final set relies on 1. onset delta, 2. ALSFRS questionnaires, 3. FVC, and 4. weight. Although this set may not be the most powerful set in making the prediction, because all above mentioned variables are commonly used in clinical settings and are measured using standard protocol, these variables are potentially 1. available in more patients, and 2. subject to less measurement error.

R script lmSigmoidNoImpute.R was used to assist feature selection.

forward/backward feature selection for a:

.	Estimate	Std. Error	t value	P
(Intercept)	5.42E+00	3.23E-01	16.759	< 2e-16 ***
fvc_percent.._min	-1.16E-02	2.41E-03	-4.821	1.52E-06 ***
q9_climbing_stairs.na_first	2.69E-01	3.73E-02	7.199	8.08e-13 ***
alsfrs_total.na_min	-2.22E-01	1.84E-02	-12.057	< 2e-16 ***
alsfrs_total.na_first	1.85E-01	2.16E-02	8.541	< 2e-16 ***
fvc_percent.._slope	-7.48E-01	1.69E-01	-4.423	1.02e-05 ***
onset_delta.na	4.20E-03	3.10E-04	13.549	< 2e-16 ***
onset_delta.na^2	1.25E-06	1.58E-07	7.897	4.33e-15 ***
weight.kg_slope	-3.50E+00	8.32E-01	-4.203	2.73e-05 ***

q4_handwriting.na_mean	2.07E-01	5.22E-02	3.956	7.86e-05 ***

forward/backward feature selection for b:

.	Estimate	Std. Error	t value	P
(Intercept)	-6.11E-03	8.92E-02	-0.068	0.9454
"onset_delta.na"	-6.30E-05	9.05E-06	-6.958	4.46e-12 ***
"alsfrs_total.na_mean"	7.24E-02	9.05E-03	7.997	1.98e-15 ***
"alsfrs_total.na_mean"^2	-3.53E-03	1.44E-04	-24.56	< 2e-16 ***
"alsfrs_total.na_max"	-7.90E-03	3.76E-03	-2.102	0.0356 *
"q9_climbing_stairs.na_min"	2.76E-01	2.79E-02	9.889	< 2e-16 ***
"alsfrs_total.na_max"*"q9_climbing_stairs.na_min"	-8.86E-03	8.48E-04	-10.44	< 2e-16 ***

Collectively, find 6 features for both a and b.
Final features selected for predicting parameter a:
onset delta; alsfrs scores; fvc_percent; q9_climbing_stairs; weight
Final features selected for predicting parameter b:
onset delta; alsfrs scores; q9_climbing_stairs; trunk

1. random forest

Random forest method, as implemented in R package 'randomForest' were used to make the predictions. 1000 trees were generated for a, and b independently using the above mentioned feature set. Default was used for all other variables in the function.
R script lmSigmoidRandomForestALLSamples.R was used to implement the method.

Conclusion/Discussion

The method implemented here highly dependent upon two core features: ALSFRS total score, and onset delta. In cases where these two features are not available, this method will not be able to make accurate prediction. Because clustering was not used prior to prediction in this method, one can potentially cluster samples into different clusters based on feature availability, and learn model for each cluster. However, due to time constraint, this was not implemented for this challenge.
I would consider myself a beginner of applying machine learning. Improvements, including a more systematic feature selection, may further boost model performance.
Does ALS disease truly follow a sigmoid like progression? I don't know.

Comparing a linear model fit of ALSFRS total score with a sigmoid model fit resulted the figure below:

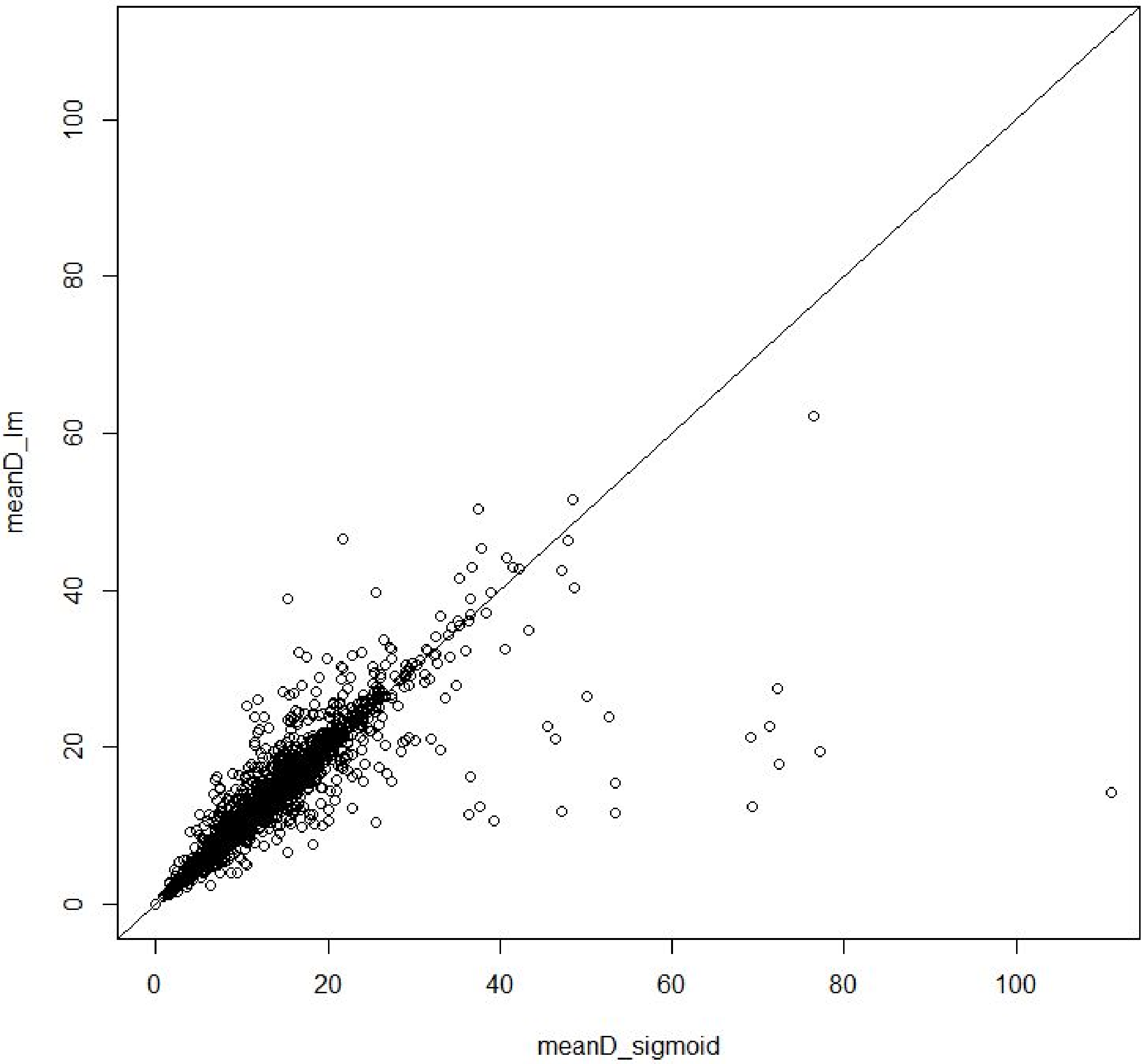

Each dot represent one patient. Sum of absolute distance from predicted value from linear model to observed ALSFRS score is shown on y- axis. Sum of absolute distance from predicted value from sigmoid model to observed ALSFRS score is shown on x-axis. On the first look of this figure, it appears that linear model has better fit. However, as mentioned above, because the parameters a and b for sigmoid model was estimated using a linear model where squared distance of log(40 / (40 - y) - 1) from the predicted was minimized instead of y, the a and b may not be the best estimator minimizing the sum of absolute value. Further comparison on which patient fits linear better, and which patient fits sigmoid like curve better may result in further insight.

Finally, just for fun, I am attaching a sloppy figure - extract everyone whose ALSFRS slope is less than -2, plot ALSFRS score against ALSFRS delta, regardless which patient it is from. It also suggests a somewhat sigmoid like curve rather than linear :)

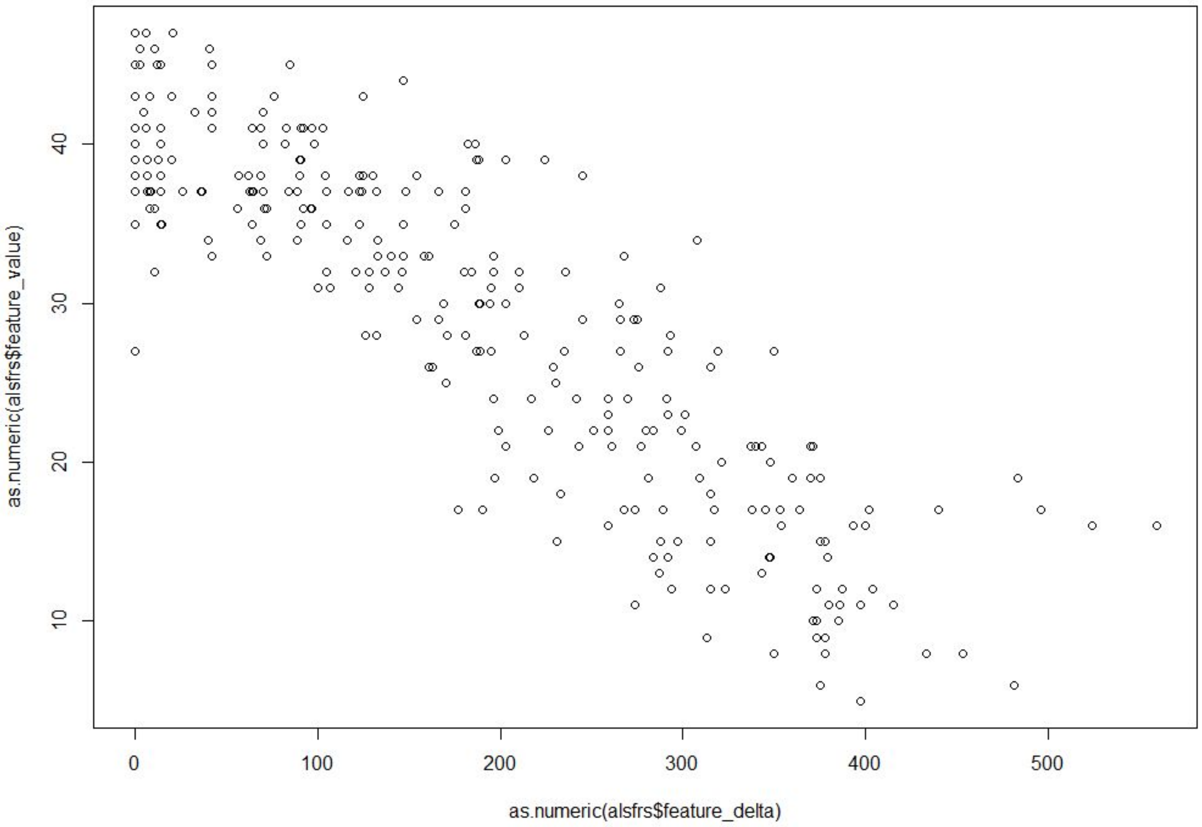

References

Liaw, A. and Wiener, M. (2002). Classification and Regression by randomForest. R News 2(3), 18--22.
Frost, J. and Massagli, M. (2008). Social Uses of Personal Health Information Within PatientsLikeMe, an Online Patient Community: What Can Happen When Patients Have Access to One Another's Data. J Med Internet Res 10(3):e15

Team Jinfeng Xiao: Random Forest Based Solution to ALS Stratification Challenge

Jinfeng Xiao
Center for Biophysics and Quantitative Biology, The University of Illinois at Urbana-Champaign, USA
This project has been made public as part of the challenge archive.

Introduction

My solutions to the sub-challenges 1 & 3 are based on random forest (R package *randomForest*[1]), and those to subchallenges 2 & 4 are based on survival random forest (R package *randomForestSRC*[2,3,4]). For sub-challenges 1 & 3, I used a home-made pipeline for feature selection, while for sub-challenges 2 & 4 the built-in importance function of the package was used to select features.

Methods

The followings are my steps to train models and make predictions. Here I would like to make it clear what I mean by writing "covariates" or "features". By "covariates" I refer to the variables, possibly longitudinal, given in the original data file. As we know the challenge rule requires me to first select 6 covariates from a whole bunch of them. By "features" I mean the information I extracted from the covariates and fed into machine learning algorithms. Details about how I did that will follow. Source codes and instructions to run them are given in the files part of this project.

1. Put together all data for each patient.

The first thing I did was to read in and rearrange the training data of each sub-challenge into matrices. Each row contains the full information about a patient. Each entry contains the full information in the first 90 days about a covariate of that patient, formatted like this:

FeatureValue1;FeatureDelta1;FeatureValue2;FeatureDelta2; ... . Then I dropped those features whose missing rate across training data is no less than 50%.

2. Encode covariants into features

Since the random forest algorithm requires the same number of inputs (some of which may be imputed) from all patients in training data, the covariates and their delta values must be converted to some features that on one hand satisfy the requirements from random forest algorithms and on the other hand contains as much information from the covariants as possible. Therefore I divided the covariates into 3 classes and encoded each class into features in a specific way. Note that for sub-challenge 3 & 4 a binary feature "I?" was added to reflect whether there was an "I" in patient ID.

A) Static categorical covariates with uniform delta

Covariates of this type are each converted into (n+1) binary features, where n is the number of levels of a covariates. The first n binary features indicates which level the covariate is at, while the last 1 extra binary feature indicates whether this covariate is missing.

B) Static continuous covariates with uniform delta

Covariates of this type are simply each converted into 1 continuous features whose value is the value of that covariate.

C) Longitudinal covariates

Covariates of this type are all considered as continuous and each converted into 13 continuous features: number of records, mean, 1st recorded value, 1st time of record, last recorded value, last time of record, maximum value, minimum value, standard deviation, mean slope between consecutive records, standard deviation in slopes between consecutive records, maximum slope between consecutive records, and minimum slope between consecutive records.

3. Fill in missing data

The way I converted covariates to features basically avoided the missing data problem from binary features. For continuous features, I applied different strategies to fill in missing data. For sub-challenge 1, I used the function na.roughfix in *randomForest* package, which replaced NAs with medians of known values. For the rest sub-challenges I assigned (100 * maximum of known values) to all NAs.

4. Feature selection and prediction

The way I selected the 6 covariates and assigned the cluster number differ a lot from sub-challenges 1 & 3 from sub-challenges 2 & 4. The method for sub-challenges 1 & 3 was selected based on 10 repeated local sub-sampling validation and leaderboard feedback. A different method was used for sub-challenges 2 & 4 so that not all eggs were put in the same basket.

A) Sub-challenges 1 & 3: Weighed path across the forest

In sub-challenges 1 & 3 I first trained a random forest with all available features. Then I dropped the patients in training data down the forest and tracked their path. Every time a patient went through a tree, the first node he visited has weight 6, 2nd with weight 5, ..., 6th with weight 1, and all the rest with weight 0. I added up the weights of each feature across all paths of a patient across the forest. Now each patient was represented as a point in N-dimensional space, where N was the number of features and the coordinates were the weights of the features. Then that space was shrunk to a n-dimensional space, where n is the number of covariates. The way I converted weights of features to weights of their covariates was to take maximum in sub-challenge 1 and mean in sub-challenge 3. Then I applied k-medoid clustering (k=6) to the patients in the n-dimensional space. Each cluster had 6 covariates whose features had the highest weight, and a random forest were trained for each cluster with those features. When a new patient came in, I assigned the cluster number of the training patient with minimum distance to the new patient in that space as the cluster number of the new patient, and selected the 6 most significant covariates of his cluster. Those 6 covariates were then turned in features again and fed into the corresponding random forest to make predictions.

B) Sub-challenges 2 & 4: Importance function of *randomForestSRC*

In sub-challenges 2 & 4 I first trained a random survival forest with all available features and ranked the features with their importance given by R package randomForestSRC. When a new patient came in, I took out his 6 highest-ranked non-NA covariates, and used those covariates to train a new survival forest and make predictions. The patients with the same list of 6 highest-ranked non-NA covariates share the same cluster number and the same new forest. The predicted values are negative risks: the greater the value is, the longer the patient is supposed to live.

Results

The important features of each cluster of sub-challenges 1 & 3, and the important feature lists for sub-challenges 2 & 4 to choose non-NA ones from, are given below in the order of descending importance.

Sub-Challenge 1

Cluster 1: onset delta, Q1 Speech, ALSFRS Total, fvc, fvc percent, Hemoglobin.
Cluster 2: onset delta, Q1 Speech, ALSFRS Total, trunk, fvc, fvc percent.
Cluster 3: onset delta, Q1 Speech, ALSFRS Total, trunk, Potassium, fvc.
Cluster 4: onset delta, Q1 Speech, ALSFRS Total, Potassium, fvc, fvc percent.
Cluster 5: onset delta, Q1 Speech, ALSFRS Total, Hemoglobin, fvc, fvc percent.
Cluster 6: onset delta, Q1 Speech, Hemoglobin, ALSFRS Total, Platelets, Glucose.

Sub-Challenge 2

fvc percent, fvc, pulse, Age, bp systolic, ALSFRS Total, onset delta, leg, Creatinine, weight, Q8 Walking, Potassium, ALT, bp diastolic, fvc normal, Bicarbonate, Sodium, Q3 Swallowing, AST, mouth, Q9 Climbing Stairs, Bilirubin, Alkaline Phosphatase, trunk, hands, Q7 Turning in Bed, Blood Urea Nitrogen, Q5a Cutting without Gastrostomy, respiratory rate, Platelets, Gender, treatment group, Phosphorus, Q1 Speech, Chloride, height, Q6 Dressing and Hygiene, Q2 Salivation, CK, Protein, Q5 Cutting, Glucose, Hemoglobin, Absolute Lymphocyte Count, Calcium, Absolute Neutrophil Count, Absolute Basophil Count, Red Blood Cells, Hematocrit, Q4 Handwriting, Absolute Eosinophil Count, Gamma-glutamyltransferase, White Blood Cell, respiratory, Albumin, Absolute Monocyte Count, Q10 Respiratory, if use Riluzole, onset site, Race.

Sub-Challenge 3

Clusters 1 to 5: onset delta, Age, onset site, ALSFRS Total, ALSFRS R Total, mouth.
Cluster 6: onset delta, Age, onset site, ALSFRS Total, ALSFRS R Total, Q3 Swallowing.

Sub-Challenge 4

onset delta, hands, mouth, ALSFRS Total, ALSFRS R Total, Age, Q4 Handwriting, Q1 Speech, R2 Orthopnea, respiratory R, Q6 Dressing and Hygiene, Q5 Cutting, trunk, Q2 Salivation, Q3 Swallowing, respiratory, ALS Staging Total, R1 Dyspnea, Q9 Climbing Stairs, onset site, Q8 Walking, R3 Respiratory Insufficiency, leg, Q7 Turning in Bed, ALS Staging Com, ALS Staging Move, ALS Staging Eat, ALS Staging Breath, MEDHx Thyroid, Gender, MEDHx Diabetes, I?, family ALS hist, MEDHx Hypertens.

References

[1]A. Liaw and M. Wiener (2002). Classification and Regression by randomForest. R News 2(3), 18-22.
[2]Ishwaran H. and Kogalur U.B. (2015). Random Forests for Survival, Regression and Classification (RF-SRC), R package version 1.6.1.
[3]Ishwaran H. and Kogalur U.B. (2007). Random survival forests for R. R News 7(2), 25-31.
[4]Ishwaran H., Kogalur U.B., Blackstone E.H. and Lauer M.S. (2008). Random survival forests. Ann. Appl. Statist. 2(3), 841-860.

Acknowledgements

Sincere thanks to Dr. Jian Peng for very useful thoughts and discussion.

Team barsinai: A Combined Model for predicting ALSFRS slope

Yoav Bar-Sinai
Medial Research, Kfar Malal, Israel

- I agree to publish my project

Abstract

My method utilizes the Lasso and GBM for choosing 6 features per individual. GBM is used for predicting ALSFRS slope based on the chosen features.

Background/Intro

Feature Generation

For quantitative features such as ALSFRS and lab tests, I measured values such as the mean value and the slope over time.
Qualitative features were handled using dummy variables in the standard way.
An extended model table was constructed in the following way: each time period of a year for each subject corresponds to a subject in the extended table. For example, the subject with id 649 has data records of two years, thus he appears as two different subjects in the extended table.

Feature Selection

In the first stage I used the Lasso. I have used cross validation to determine the set of features which give the best prediction (in the sense of MSE).

Using these features on the second stage, I have built a GBM. The package gbm in R specifies feature importance, giving a natural feature importance ranking. I have used this ranking to select features based on availability of the features in the test patient.
In addition I have performed k-means clustering on the training data. Choosing k=8 I have added the relevant cluster of the test patient to the output of the selector and used it as another feature for the predictor.

Prediction

For each subject, I choose features by the feature importance list according to the features existing for this subject. Again, I use the lasso to select a subset of the variables and then apply GBM for the prediction.
The novelty in my approach is the combination between the Lasso and GBM for feature selection. The Lasso serves as a primary coarse filter that does not specify feature importance but rather sends less important features to 0.
The second and more fine filter is the GBM which specifies feature importance and enables a natural ranking of features.

Methods

What clinical variables did you use in your selector and predictor models?
In the selector model I used all clinical variables except adverse event and medications.
In the predictor model I have obviously used the variables from the selector output. The feature ranking defines my feature selecting method:
[1] "onset.u.delta"
[2] "ALSFRS.u.Total"
[3] "leg"
[4] "weight"
[5] "Age"
[6] "diag.u.delta"
[7] "Creatinine"
[8] "pulse"
[9] "bp.u.diastolic"
[10] "Q5a.u.Cutting.u.without.u.Gastrostomy"
[11] "Q1.u.Speech"
[12] "Red.s.Blood.s.Cells.s..o.RBC.c"
[13] "Q6.u.Dressing.u.and.u.Hygiene"
[14] "Phosphorus"
[15] "svc.u.percent"
[16] "Blood.s.Urea.s.Nitrogen.s..o.BUN.c"
[17] "Sodium"
[18] "ALT.o.SGPT.c"

[19] "AST.o.SGOT.c"
[20] "Potassium"
[21] "Glucose"
[22] "Hemoglobin"
[23] "Hematocrit"
[24] "White.s.Blood.s.Cell.s..o.WBC.c"
[25] "Albumin"
[26] "Calcium"
[27] "Urine.s.Ph"
[28] "Chloride"
[29] "Bicarbonate"
[30] "Absolute.s.Neutrophil.s.Count"
[31] "Absolute.s.Eosinophil.s.Count"
[32] "Absolute.s.Monocyte.s.Count"
[33] "Platelets"
[34] "Bilirubin.s..o.Total.c"
[35] "Absolute.s.Lymphocyte.s.Count"
[36] "Protein"
[37] "Alkaline.s.Phosphatase"
[38] "Gamma.d.glutamyltransferase"
[39] "Total.s.Cholesterol"
[40] "Eosinophils"
[41] "Monocytes"
[42] "Basophils"
[43] "Lymphocytes"
[44] "CK"
[45] "Neutrophils"
[46] "Triglycerides"
[47] "HbA1c.s..o.Glycated.s.Hemoglobin.c"
[48] "ALSFRS.u.R.u.Total"
[49] "Q10.u.Respiratory"
[50] "Q2.u.Salivation"
[51] "Q3.u.Swallowing"
[52] "Q4.u.Handwriting"
[53] "Q5.u.Cutting"
[54] "Q7.u.Turning.u.in.u.Bed"
[55] "Q8.u.Walking"
[56] "Q9.u.Climbing.u.Stairs"
[57] "R1.u.Dyspnea"
[58] "R2.u.Orthopnea"
[59] "R3.u.Respiratory.u.Insufficiency"
[60] "hands"
[61] "mouth"
[62] "respiratory"
[63] "respiratory.u.R"
[64] "trunk"
[65] "Q5b.u.Cutting.u.with.u.Gastrostomy"
[66] "Gender"

[67] "Race"
[68] "onset.u.site"
[69] "if.u.use.u.Riluzole"
[70] "bp.u.systolic"
[71] "respiratory.u.rate"
[72] "temperature"
[73] "BMI"
[74] "height"
[75] "fvc.u.percent"
[76] "family.u.ALS.u.hist"
[77] "treatment.u.group"

Did you perform any filtering, data normalization, data filtering? Was the data manipulated in any way?

I used almost all data without filtering. I normalized the data in the final training table so I can use the Lasso.

What was your approach regarding missing values and data imputation?

I used the average value to impute missing values.

Detailed description of the underlying method: how did you perform the model and feature selection in the selector program? what methodology did you use for the predictions?

See Backgroung\Intro.

Description of statistical tests used.

- MSE Cross validation in the selection stage with the Lasso.
- The rest was based on tests on the spike and leaderboard data which were left out of the training set until the very end.

Conclusion/Discussion

Feature generation was probably one of the hardest tasks in the challenge for me. The variability of some of the features as a function of time raises many options for feature generation. I used trial and error to choose the technique at this stage.

Choosing best features - I used common techniques - the Lasso and GBM. The novelty is the combination.

**Handling the source code

The source code is given in the project files under the name final.zip.

The project is all writen in R. The predictor and selector are quite simple. They use some other functions (from other files) that are also pretty easy to understand.

To load the project using the predictor and selector, you may use the .sh files that are also given in the zip file.

If you want to use my Cross - Validation method you should first move the predictor and selector files from the project (or alternatively erase the last row in each of them), and then source CrossValidation.R. There is a method called SetParams() in which some parameters related to the model can be chosen (I didn't have so much time for optimization so it is probably possible to improve the results of my final model).

References

Friedman, J., Hastie, T. & Tibshirani, R. glmnet: Lasso and elastic-net regularized generalized linear models. R package version 1.9-5. http://cran.r-project.org/web/packages/glmnet/index.html (2013).
Ridgeway, G., with contributions by Edwards, D., Kriegler, B,. Schroedl, S., and Southworth, H. https://cran.r-project.org/web/packages/gbm/gbm.pdf (2013)

Team Veltys: A Mixture Model for Predicting ALSFRS Slope

Jeanne Avril[1], Philippe Février[1,2], Thomas Larrieu[1,3], Violette Nahmias[1] and Jean-Bernard Salomond[1,4]
1 Veltys
2 Centre de Recherche en Économie et Statistique (CREST)
3 Département d'Économie École Polytechnique
4 CEREMADE Université Paris-Dauphine
Abstract: We created algorithms using boosted trees. The first algorithm uses a Boosting tree model and a Classification Expectation-Maximization (CEM) method to construct patient specific subgroups and define subsets of 6 features that are especially predictive for each patient subgroup. The second algorithm uses boosted trees to predict the ALSFRS slopes of any patient based on the 6 relevant features selected by the first algorithm. Our algorithms shows that the optimal allocation of patients to predict their ALSFRS slope is a single cluster where all patients share the same top 6 relevant features.

1- Introduction

Amyotrophic lateral sclerosis, or ALS, is a neurodegenerative disease that involves the degeneration and death of the nerve cells in the brain and spinal cord that control voluntary muscle movement. It typically leads to death within 3-5 years but the disease progression is very heterogeneous across the patient population.

The goal of this challenge is to use computer science tools to help ALS' specialists to better understand and predict the disease progression throught the ALSFRS slope in order to decrease the costs of trials and research.

To do so, we develop a Mixture of Boosting tree model estimated with Classification Expectation-Maximization algorithm. These algorithms are well suited for heterogeneous subjects and sparse data, which is the case for the PRO-ACT database.

2- Methods

2.1 - Data pre-processing

The PRO-ACT database contains information about ALS patients who participated in various clinical trials. The original training database contains information on 8 635 patients. 2 230 features are recorded but many of them are given for small numbers of patients only. Note that only 16 features among this 2 230 are recorded for more than 70% of the patients.
In order to deal with this issue, we define, for each feature, a binary variable to indicate if it is missing or not. In the algorithms, missing data are automatically imputed based on reduction on training loss.
As shown in the Figure below, there are also endogeneity issues, as patients whose symptoms have appareared a long time before the beginning of the trial (low onset delta) are more likely to be slow progressers (have a slope close to zero) since they have survived. Patients with onset delta close to zero are more heterogeneous.

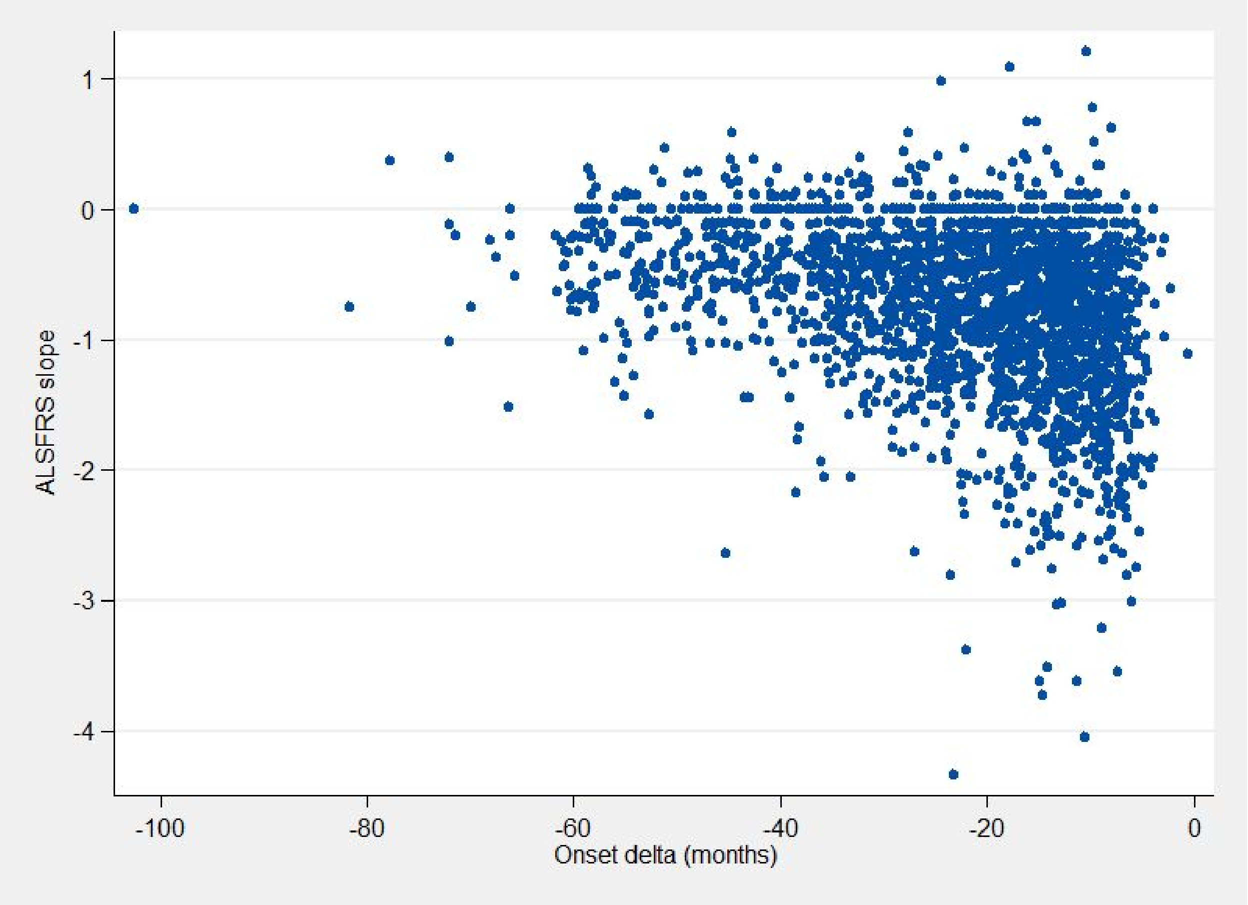

Because of this specificity, links between the slope and the other features are more difficult to see using simple statistics.
Most features are time-varying (e.g. weight), but measures are taken at different dates for each patient and each feature. To take this specificity into account, we include, for each time-varying feature, the following statistics as subject features:

- The maximum, minimum, last, first and mean measurement values
- The median absolute deviation of measurement values

- The standard deviation of measurement values
- The sum of all measurement values
- The delta value associated with the first measurement value
- The delta value associated with the last measurement value
- The slope of the time series, defined as (last measurement value – first measurement value)/(last delta value – first delta value)
- We compute each statistic using only records with delta value ≤ 91 days.

Finally, we use all the features available in the training dataset except features included in the Adverse Events and Concomitant Medication categories. Those categories are so heterogeneous and patient-specific that they are not predictive.

2.2 - Statistical models

To fit the data, we develop a *mixture model* which has the advantage of being both very flexible and well known. This kind of modelling is very useful for clustering individuals when the number of clusters is unknown, which is the case here.

A mixture model is a probabilistic model for representing the presence of subpopulations within an overall population, without requiring that an observed data set should identify the sub-population to which an individual observation belongs. Formally a mixture model corresponds to the mixture distribution that represents the probability distribution of observations in the overall population. In the following we will denote by Z_i the hidden variable indicating to which population the patient i belongs. Let $Y_i$be the output variable (i.e. the 3-12 month ALSFRS slope here), and let x be the observed clinical data.

We fit a mixture model for Y_i of the form:

$Z_i|x \sim p(Z_i|x)$,

$Y_i|Z_i=k,x \sim N(f_k(x),\sigma)$

The likelihood of such a model is highly difficult to handle. We thus choose to compute the maximum likelihood estimator using numerical technique.

We thus obtain an approximated maximum likelihood estimator for the allocation Z_i and the regression functions f_k.

We now quickly introduce the method used to estimate each regression function f_k.

Our estimation models are based on Extreme Gradient Boosting. The term “Gradient Boosting” is proposed by Friedman[1].

Boosting is a machine learning ensemble meta-algorithm for reducing bias primarily and also variance. Boosting algorithms consist of iteratively learning weak classifiers with respect to a distribution and adding them to a final strong classifier. Therefore, these algorithms are very efficient on predicting outcomes for heterogenous subjects. Here we used the already implemented method *XGBoost* available for R.

The XGBoost package has many advantages :

- Speed: it can automatically do parallel computations on Windows and Linux, with OpenMP. It is generally over 10 times faster than the classical gbm;
- Sparsity: it accepts sparse input for both tree booster and linear booster. Missing data are automatically imputed based on reduction on training loss;
- Customization: it supports customized objective functions and evaluation functions;

- Cross Validation: it allows to directly cross validate the result on a given test set.

2.3 - Fitting the model

The first step of the challenge requires to construct patient-specific clusters and define, for each cluster, a subset of 6 features that are especially predictive. This is obtained by analysing the approximated maximum likelihood estimator of the mixture model described in the previous section.
To fit such a model we use an approximated classification expectation-maximization (CEM) algorithm[4].
The CEM algorithm iterates the two following steps:
1- The expectation step, which creates a function for the expectation of the log-likelihood evaluated using the current allocation of patients in X number of clusters.
2- The maximization step, which re-allocates patients to maximize the expected log-likelihood found on the first step.
The CEM Algorithm has proven to perform nicely for clustering purposes, which led us to this choice.
For the maximization step, we estimate f_k given $(Z_i)_i$ using a restricted boosted tree. More precisely, our estimator is a boosted tree trained on only 6 features that are selected for their predictive performances. To select those 6 features, we train another boosted tree on all the features to predict the slope, and obtain their predictive performances as the sum of L_2 loss reduction for each split based on this variable.
A difficulty of this kind of algorithms is their tendency to get stuck in local mode of the (extremely) complex likelihood. A solution to avoid such problems is to run the CEM algorithm using different initialization points. Another possibility is to initialize the algorithm at a point *not too far* from the optimum. This solution is highly attractive as it both speeds up the computations and avoids the tricky choice of initialization points. In our case, we initialized the CEM by allocating each patients to a group obtained by fitting a simple regression tree.
The hyperparameters of both the gradient boosting the CEM algorithm and the initial regression tree were all found using cross validation. We now go on and present the two steps of our solution to the challenge.

2.4 - Predicting the ALSFRS slope

We now present how, given a new patient's clinical data x, our model can predict its ALSFRS slope. The first difficulty is to allocate a new patient to a given component (or cluster) of the mixture. To do so, we choose to estimate his allocation variable $(Z x) for the new patient using the marginal maximum likelihood estimator denoted (\hat{z}). Once the patient is allocated to a cluster, we simply predict his or her slope with the fitted regression function (f_\hat{z}(x)). Once this is done, we compute the confidence index using a Bootstrap estimate of the standard deviation of our prediction (\hat{s}). Denoting (S)$ the standard deviation of the ALSFRS slope on the training data, our confidence index is then

$CI(x)=1-\hat{s}\ S.$

If the CI is 1, this indicates that according to our model, the prediction is extremely precise. A confidence index of 0 on the contrary is given when the precision of our prediction is worse than simply giving the mean of the ALSFRS slope as an estimator.

3- Discussion

Our approach is designed to respond to the challenge rules by finding the optimal allocation of patients in clusters. As it turned out, our method converged to a single cluster model, indicating that there is -according to our model- not enough differences between patients *prediction function* to differentiate them. This was observed even though we ran our Approximated CEM algorithm from different initialization points, including allocation Z obtained with a single regression tree, and when tuning the different hyperparameters.

We believe that result is important and robust. The 6 tops features selected by our model are the following:

- onset_delta
- fvc_percent
- ALSFRS_Total
- weight
- Q1_Speech
- trunk

These top features are consistent with the results of the first ALS challenge. Time from Onset is the most predictive features identified by our algorithm and FVC, Weight, ALSFRS_Total were also present in the top of predictive features.

The implementation of our algorithms leads to the following out of samples cross validation (repeated 10 times):

RMSE :

Min.	1st Qu.	Median	Mean	3rd Qu.	Max.
0.4946	0.5207	0.5974	0.5761	0.6212	0.6501

COR :

Min.	1st Qu.	Median	Mean	3rd Qu.	Max.
0.3225	0.3521	0.3925	0.3910	0.4197	0.4683

The model shows good results and seems to be close to the top leaderboard scores.

We agree to make this submission public.

4- Running scripts

According to the rules of the challenge we created 2 scripts "selector_2.R" and predictor_2.R". The first one can take any patient data as input and can outputs a text file containing the cluster number of the

patient and the 6 features selected. The second script take the output of the first script as input and predict the ALSFRS slope of the patient using the 6 selected features only.
Both of these scripts use R objects created by the "train_Mixture_2.R" and the "Bootstrap_Train_2.R" scripts. These two files are loaded in the Files section of this project. They require the installation of 'xgboost', 'rpart', 'FactoMineR', 'data.table' packages.
One only needs to run "train_Mixture.R" first and then "Bootstrap_Train.R" to create the R objects used in the selector and predictor algorithms. The first script train the Mixture model described in section 2.2 on the trainning + leaderboard data. The second script uses a Bootstrap method to evaluate the confidence interval of our prediction. The predictor script along with the ALSFRS slope outputs this confidence interval.

References

1Jérome H.Friedman, Greedy Function Approximation: A Gradient Boosting Machine (1999)
2Tianqi Chen, Tong He, Michael Benesty. Package 'xgboost'.R package version >= 2.10 https://cran.r-project.org/web/packages/xgboost/xgboost.pdf (2015)
3Lindsay, B. G. Mixture Models: Theory, Geometry, and Applications. NSF-CBMS Regional Conference Series in Probability and Statistics 5. Hayward: Institute of Mathematical Statistics (1995).
4Gilles Celeux, Gerard Govaert. A classifcation EM algorithm for clustering and two stochastic versions. [Research Report] RR-1364 (1991)
5Terry Therneau, Beth Atkinson, Brian Ripley. Package 'rpart'. R package version >= 2.15 https://cran.r-project.org/web/packages/rpart/rpart.pdf (2015)

Team uci-cbcl: An Ensemble Method for Predicting Amyotrophic Lateral Sclerosis Progression

Yeeleng S. Vang[1] and Xiaohui Xie[1]
[1] Department of Computer Science, University of California Irvine, Irvine, CA, 92697, USA

Abstract

Our method utilizes k-means clustering for stratification of patients and then an ensemble model consisting of gradient boosting machine and bayesian additive regression tree to predict ALS Functional Rating Scale slope.

Introduction

In subchallenge 1, we were tasked with making prediction of patient's 3-12 month ALS Functional Rating Scale (ALSFRS) slope using the PRO-ACT database. In subchallenge 3, we were tasked to make similar ALSFRS prediction using National Registries database. In both subchallenges, static features and time series features were considered. Both challenges resulted in stratifying patients into 16 groups using k-means clustering. A separate predictive model was generated for each individual clusters using an ensemble of gradient boosting machine and bayesian additive regression tree.

Methods

Regression models

Two main regression models were used in ALSFRS slope prediction: gradient boosting machine (gbm) and bayesian additive regression tree (BART). R's "xgboost" package[1] was used to implement gbm and "BayesTree" package[2] was used to implement BART. The "xgboost" package provides user with parameters max.depth and eta. These parameters were chosen after exhaustive line search using 10-fold cross-validation of patients within the cluster. The "BayesTree" package provided user with parameters: ndpost, ntree, and sigquant. These parameters were set based on Team 1's result[3] and similar to what Team 1 did, ten runs of BART were averaged to minimize prediction variance.

Data pre-processing

The PRO-ACT training set, additional data, and leaderboard set were all pre-processed the same way as described below. Our model training was decided to be completed in a strictly supervised manner, therefore only patients whom were provided with gold standard ALSFRS slopes were kept. Since the feature set of the entire PRO-ACT database exceeded 6000 features, we focused on those that were used successfully by Team 1 in the first ALS competition[3]. Static features used includes age, gender, race, onset_delta, diag_delta, onset_site, if_Use_riluzole, treatment_group, and family_als_hist. Time series features used were restricted to those that were associated with form name alsfrs, fvc, svc, and vitals. For these time series features, the max value, min value, mean value, and last value were extracted and included as patient features. Also like what Team 1 did, a derivative time series of pairwise slopes were calculated for each time series features. This slope is defined as the ratio of the difference between consecutive pair of measurement value over the difference in their delta values. Max slope, min slope, mean slope, and last slope were extracted and included as patient features. Missing data in real-valued features were imputed with the feature's average while missing data in categorical features were imputed with the most frequent category of that feature. Binary features where mapped to 0 and 1 while categorical features were transformed to binary features using one-hot encoding. All features were normalized with zero mean and unit variance.

The National Registries data set were pre-processed in a similar manner but since it lacked categorical features, no one-hot encoding was required. As before, all features were normalized with zero mean and unit variance.

Feature selection

Both subchallenges necessitated a feature selection step to reduce our feature set into a more manageable number. The selection by filtering function in "caret" package[4] was use to check the strength of relationship between individual features and the target response and returned a reduced feature set. The six features of each clusters are obtained by training a linear model and ranking the features by the absolute value of their t-statistics.

Stratification

Stratification was obtained using k-means unsupervised clustering for both subchallenges. The number of clusters chosen for our models were 16 and was found via 10-fold cross-validation minimizing the

average RMSE across all clusters using the gbm predictor. During the clustering step, if a cluster was found to consist of only one patient, e.g. an outlier, that patient was remove from the training set and k-means were applied again to the now reduced training set.

Ensemble model

The ensemble model is given as:

$$.5*gbm+.5*BART$$

Conclusion

Based on previous DREAM challenge experiences, we found gradient boosting approaches worked well for clinical data and these types of challenges. For this particular challenge, the "xgboost" package was used in lieu of the standard "gbm" package as "xgboost" is much faster and has won numerous Kaggle competitions[5]. BART was included as a result of its top performance in the previous ALS competition[3]. For subchallenge 1, "onset_delta" feature seems to be the most important single feature as it appears in 14 of the 16 cluster. The second most important feature is "Q1_Speech" as it appears in 10 of 16 clusters, with "Q2_Salivation" and "Q3_Swallowing" tied for third appearing in 9 of 16 clusters each. For subchallenge 3, "onset_delta" and "Q5_Cutting" were tied for morst important single feature as each appeared in 13 of 16 clusters. "Q1_Speech" is the second most important and "Q6_Dressing_and_Hygiene" the third most important features.
In conclusion, our best results were obtained using an ensemble model consisting of gbm and BART.

References

1. Chen, T. xgboost: Extreme Gradient Boosting. R package version 0.4-2. https://cran.r-project.org/web/packages/xgboost/index.html (2015).
2. Chipoman, H. & McCulloch, R. BayesTree: Bayesian Additive Regression Trees. R package version 1.6-3.2. https://cran.r-project.org/web/packages/BayesTree/index.html (2014).
3. Kuffner, R. et al., Crowdsourced analysis of clinical trial data to predict amyotrophic lateral sclerosis progression. *Nature Biotechnology*., **33** (2014).
4. Kuhn, M. caret: Classification and Regression Training. R package version 6.0-52. https://cran.r-project.org/web/packages/caret/index.html
5. Chen, T. xbgoost. GitHub repository, https://github.com/dmlc/xgboost (2015).

Authors Statement

X.X. conceived the study and supervised the analysis. Y.S.V. implemented the code and wrote the manuscript. This write-up will be made publicly available after the stated date.

Team jjacob_cub: A model for prediction of progression slope in newly diagnosed ALS patients. (Submission for subchallenge 1)

Submission by: Jeremy Jacobsen (jjacob_cub)
Research asst., Old lab, University of Colorado, Boulder
Dept. of Biochemistry
Abstract: For the prediction of ALSFR slope I utilized Gradient Boosting Regression for the imputation of a sparse feature matrix. The same model was used for feature ranking and selection.
Introduction: In order to predict the progression of a subject, potentially relevant data must be reduced to an important predictive feature subset. This can be challenging based on lack of consistency of measurements across subjects. For this reason, it is paramount to be able to impute missing features across subjects, based on existing data. My approach attempts to learn from the present data, in order to impute missing data for all features across subjects, to create a more reliable predictive tool.

Methods

Generation of a training table was completed with the "output_training_table.py" script. I used an object oriented approach to assign feature objects to each patient object. A mapping of feature objects was assigned to each patient.

features:
average (float)
slope (float)
sum (float)
ndata (float)
variance(float)
feature data container:
timemap (map)

```
For each patient attribute, a "timemap" is constructed which maps (times→feature values).  If a feature is not of a type float or int, it is dealt with later by converting it to a numeric value and saving the conversion in a mapping file.  The string to numeric mapping is stored in  "training_table_valmap.txt" so it can later be used by the predictor/selector to ensure that strings already in the table map to common values.  Once all patients are processed, features are finalized into abstract features (average,sum, etc.) by doing simple calculations on the feature maps.
```

```
After finalizing, a pandas dataframe is generated that contains a matrix of all features for all patients.  Missing values are substituted with '-9999'.  A cutoff was selected to remove abstract features that contain less than 3 unique values.  This step removed columns from training that are likely to not be useful.  A final housekeeping step removes neighboring columns that are duplicates.
```

Note1: categories “Concomitant Medication” and "Adverse Event" were not considered.
Note2: Patient features with time >=92 days were excluded.
Note3: This was only run on patients that have an ALSFR slope assigned.

Imputation of Missing Values

My approach depends heavily on the imputation of missing values, because such a large portion of the data is missing. To accomplish this task, I used the script “impute_multi.py” (multi for multiprocessing) . This script uses a learned model to fill in the missing values. I used scikit-learn's GradientBoostingRegressor for the this. The algorithm iterates over the columns, and at each iteration slices the training table into a target set (current column minus values to be imputed), and a training input (all other columns collapsed minus value to be imputed). It then makes a prediction for each missing value in the current column.

The choice of GBR was important because it trains on the model faster than others that I tried (RFR, AdaBoost, etc.). This is particularly important because the model must re-train at every column. Fortunately in addition, cross validation showed GBR performance to be superior to other models.

I tried several variations on this algorithm. For instance, I separated the data into a table that only contains float features and another that only contains int types. This way I could predict continuous data with regression and discrete data with classification. Counter-intuitively, cross validation demonstrated this approach to be detrimental. I also tried the imputation using a training set generated using all patients (including those with no ALSFR) to leverage all possible data in a semi supervised manner. This approach also yielded a less than optimal result for Person correlation during cross validation, though this could just be symptomatic of my model over-fitting on the smaller dataset.

Note: There is room for improvement here. The algorithm is stochastic when threaded and I found a quick optimum choice of processes to be 8. Additionally, it is dependent on initial feature ordering. I did not thoroughly test all aspect for optimization.

Choice of an optimal learning Model

I experimented with several regression models in the scikit-learn portfolio. Including but not limited to:
Linear Regression
Logorithmic Regression
Random Forest Regression
AdaBoost Regression
Gradient Boosting Regression
Support Vector Regression

Of these, Random Forests and Gradient Boosting proved to be the best regression models using k-fold cross validation. After iterating parameters for optimization, I found the GBR to be superior to other models I tried. Cross validation was run using scikit-learn libraries. Using the GBR __feature_importances method I pulled out a ranked list of features by importance. I then ran k-folds cross validation on consecutive features by importance. As can be seen in the following plots, imputation led to dramatic improvements.

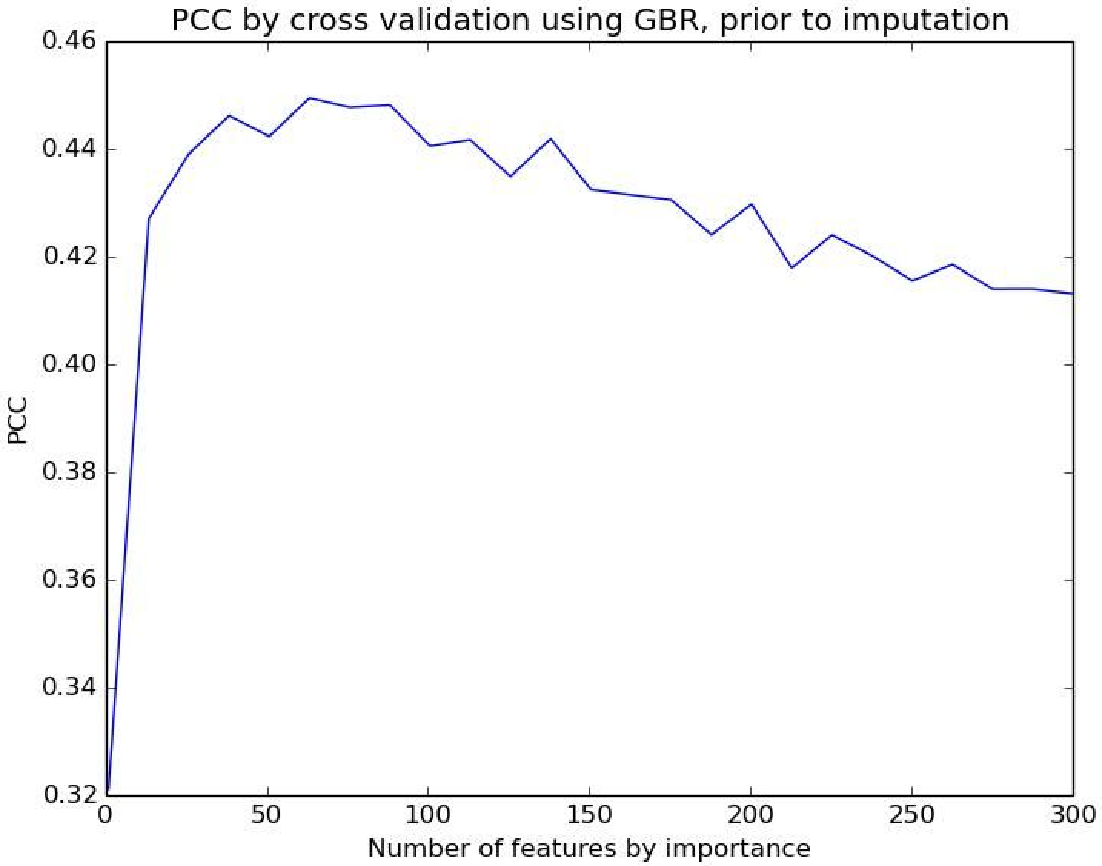
PCC by cross validation using GBR, prior to imputation
0.46
0.44
0.42
0.40
0.38
0.36
0.34
0.32
PCC
0
50
100
150
200
250
300
Number of features by importance

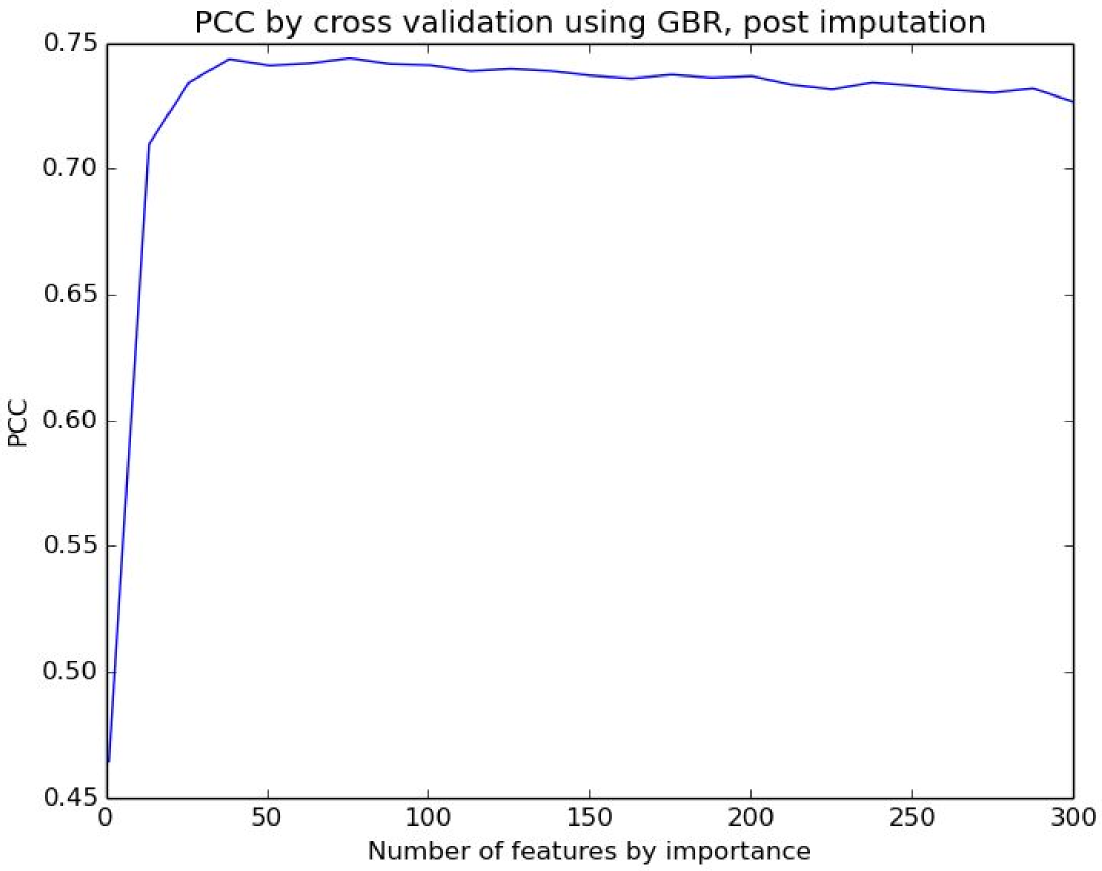

Clustering

Clustering of patients by features was tested using k-means on a normalized version of the training set. PCA clearly indicated that patients group into approximately 10 clusters (see figure below).

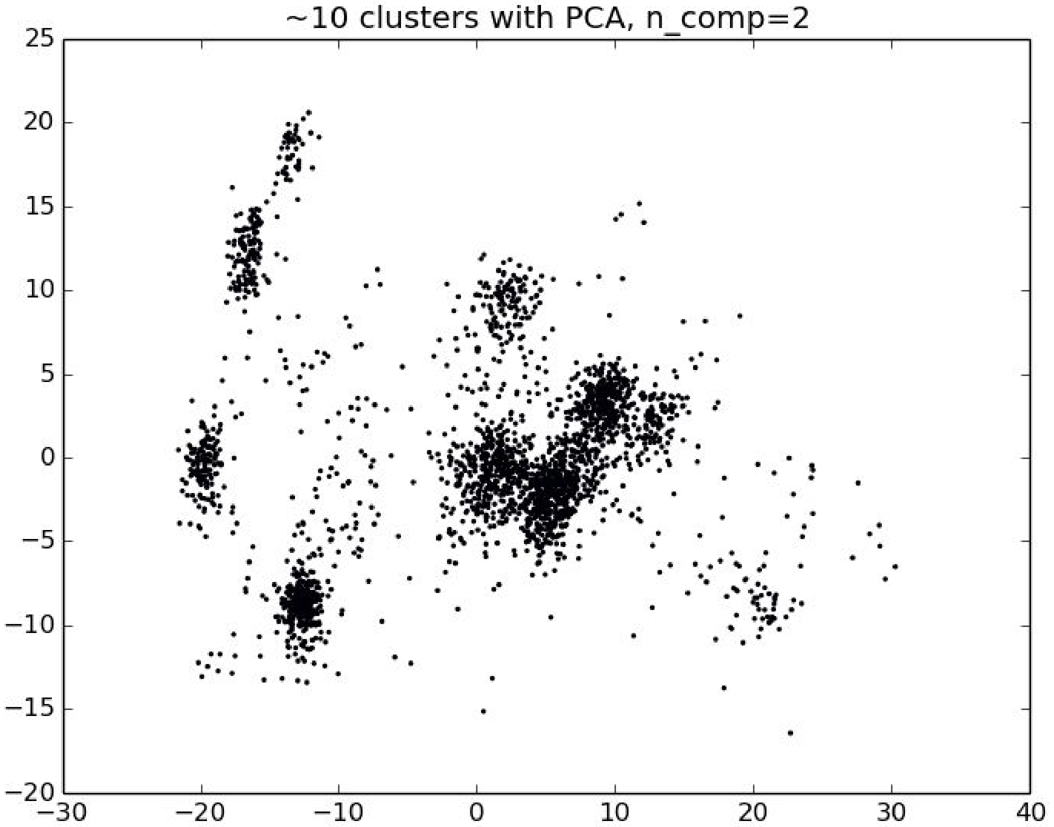

Kmeans parameters were adjusted until they reached convergence. Convergence was determined to be met when the distribution of patients (number of patients in each cluster) in each group stabilized. Once the clustering was complete I attempted to use the GBR model to get a list of features by importance, for only the patients in each cluster, thinking that this could aid in feature selection. I found through cross validation that it was not helpful to split the training set into clusters to predict slopes, nor was it helpful in the selection of the 6 patient features. I appended the patient clusters as a column in the training table so that the model could benefit from the cluster as an additional feature. This boosted performance by ~30% (from 42% to 55% for all patients PCC, with 6 features).

Selector.py

```
My selector script is simple.  It takes in a patient file, and does essentially the same thing as “output training table” to create all abstract features for the current patient.  For cluster assignment it then takes the patient feature values and compares them pair wise to each patient in the training set.  The comparison metric is a sum of correlation distance and hamming distance.  When the distance sum is minimized, it has found the patient in the training set that is nearest to the current patient.  It then looks up what cluster that patient belongs to and assigns that cluster to the current patient.
For feature selection, the selector begins at the top of a ranked list of features that was obtained using the GradientBoostingRegressor __feature_importance function.
```

```
If the patient has the current feature, it adds it to a list, if not it continues. This is done until the patient has 6 features assigned or until the end of the list is reached.  Then the file is printed out.  I tried several complex variations on the approach but could not improve on the simple version.
```

Predictor.py

```
Once again, the patient features are constructed, only this time from the 6 features selected.  The GradientBoostingRegressor is trained only on the 6 features, and a slope is predicted.  Output is returned.
```

Instructions for generating model from source:

1. Run output_training_table.py to generate raw training table.
2. Run impute_multi.py (will take some time).
3. Run column_normalize.py to feature normalizing prior to clustering.
4. Run PCA_view.py to view PCA output with 3 components
5. Run cluster_patients.py to generate patient clusters from training table
6. Run append_clusters.py to append patient cluster column to training table
7. Run xval_with_feature_imp.py to cross validate and output feature importance table.

This process should generate all necessary tables under the /tables /clustering_output folders.

Team Wonwingchung: Lasso regression model on predicting the progression on ALS patients

Contributed by Wing Chung WONG
Department of Computer Science, City University of Hong Kong
This submission is permitted to make public.

Introduction

The main challenge is to select a small amount of features over 200 features in the data set. The feature selection algorithm should select the most informative features. The target model selected is lasso regression model. This model have a built-in feature selection algorithm in order to select a small amount of features within a large group of features.

Method

2481 data objects with 118 features are the data set passing to the next stage after the following data preprocessing procedures.

1. Process of handling longitudinal features
2. Data filtering
3. Process of handling missing value
4. Data normalization

After the data preprocessing procedures, the data will be used to train the target model (lasso regression model) .

Handling longitudinal features

Only the features with ordinal data type, nominal data type, ratio data type are retained. The features value with alphabet are removed. Thus, only features with numeric value are retained in order to apply the average function to each longitudinal feature in a data object.

Data filtering

Data objects without the target feature (ALSFRS slope) are removed. In addition, the features with lower than 100 data object having the corresponding value are also removed.

Handling missing value

The missing value are filled with the mean of the corresponding feature.

Data normalization

The max-min normalization is performed. After the normalization, the data values lie between 0 and 1.

Model

Lasso regression model is used to train the data. The Lasso class in sklearn is used. And the cost function is show below.

$$J(\theta)=12m[\sum_{i=1m}(h_{\theta}(x_{(i)})-y_{(i)})_{2}+\lambda\sum_{j=1n}|\theta_{j}|]$$

$\lambda\sum_{nj=1}|\theta_j|$ is a regularization term used in lasso regression model to avoid overfitting. The coefficient of the less informative features will become 0.

1662 and 819 data object are the number of data object in training set (66%) and test set (33%).

In the training set with 1662 data objects, 10-folded cross validation was used to find the optimal θ in the equation iteratively as this input parameter will affect the feature selection algorithm in the equation. The set of features generating the smallest Mean Square Error (MSE) will be selected to be the 6 selected features.

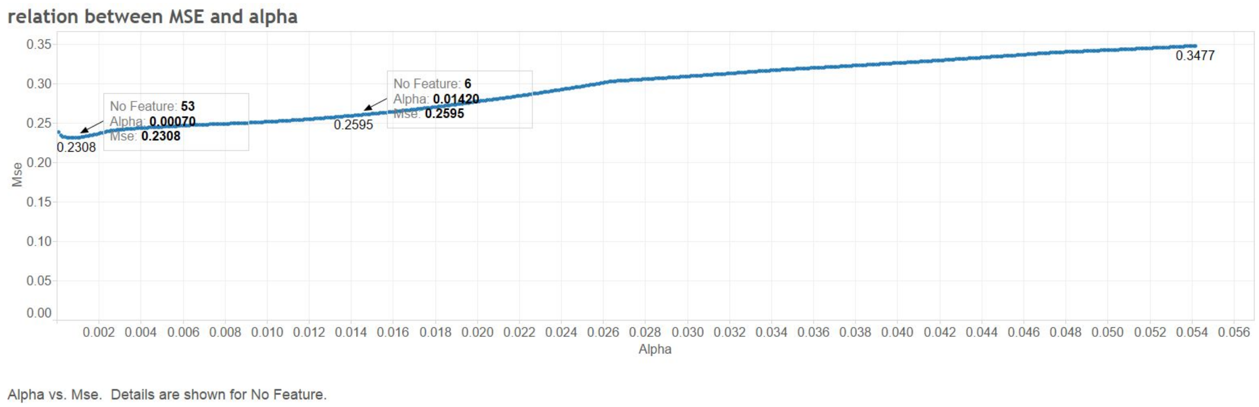

Fig. 1 showing the relation between the alpha and MSE in the iteration of cross validation with minimum MSE

Refer to Figure 1, the minimum MSE generated is 0.2308 when alpha reaches 0.0007. The MSE for the set of features selected is 0.2595 when alpha reaches 0.0142.

After the cross validation, the 6 selected features is listed below:

- onset_delta
- Q1 Speech
- Q5 Cutting
- Q7 Turning in Bed
- respiratory
- Q3 Swallowing

Model performance evaluation

Statistics are calculated to evaluate the performance of the model in different dimension.

- Training error is calculated by fitting the training set into the model.
- Generalization error is calculated by averaging the Mean squared error (MSE) or Root mean squared error (RMSE) among different iteration of cross validation.
- Test error is calculated by fitting the training set into the model.

	Training error	Generalization error	Test error
MSE	0.3081	0.3105	0.3203
RMSE	0.5551	0.5566	0.5659

The Pearson Correlation Coefficient (PCC) when fitting the model with the test set is 0.8072.

Program execution

In the File uploaded, there will be a archive file(programs.zip). The major programs required to implement model are inside this file.

The ProActProgression folder is the folder exactly identical to the one inside the linux server.
The selector program will invoke the make command. The make command is a linux command which executes the program in the Makefile. The program will handle all the compilation of the java source code. After running the make command, the java program will pass a configuration file into the program as an input parameter. The java program will select all the relevant rows based on the configuration file and these row will be the input for the predictor.
Similar to the selector program, the predictor program will also invoke the make command and the make command will handle all the compilation of the java source code. After running the make command, the java program will pass a configuration file into the program as an input parameter. The configuration file involves all the parameters required to fill the empty attributes, perform max-min normalization and the predicting the target feature (ALSFRS slope).
In the programs folder, there is a python program file. Before using this program, the data should be preprocessed. The data file should be stored in proact/data.csv and the file with target feature (ALSFRS slope) should be stored in proact/target.csv. Also, folder ,proact/10folded-cross-validation/, is also required to store the result of the cross-validation. After executing this python program, the output of the coefficient for the selected features will be printed in the terminal. For the details for each iteration in cross validation, you can refer to the 10 files in the folder,proact/10folded-cross-validation/. For the details related to the result of fitting the model with training data and test data, you can refer to the files,proact/train-result.csv and proact/train-result.csv, respectively.

Conclusion & Discussion

This project involves a complete procedure from data preprocessing stage to model evaluation stage. With the constraint on the number of the selected feature, lasso regression model is used to build the model. Lasso regression model works well in large scale of data analysis with large amount of features.

Limitation

The data objects in the dataset for building the model is just 452 which is a very small amount. When performing 10-folded cross validation, the 6 features selected for different fold are not exactly the same. As there is only a small amount of training dataset, the selected features are highly depended on the data objects in the training set. The submitted 6 features may not be the most informative one. The submitted may be overfitted to a specific set of data objects.

Further Enhancement

In addition to the 6 selected features, cluster id can be the input parameter to the predictor. This implies that the clustering algorithm can be applied to the dataset and there can be more than one lasso regression model to be built as it is possible to have different clusters and there are one lasso regression

model for each cluster. However, it may lead to a potential threat as the amount of training dataset for each lasso regression model will sharply decrease. The performance of the model cannot be guaranteed.
The features with multiple data as value are removed. Analyzing these features requires a large amount of human effort and the information from these features may not be in proportion. Improvement can be made if we can transform these values into numerical value to capture the information given by the features.

Author statement

This project is contributed by WC Wong from data cleaning to model evaluation and documentation.

References

Scikit-learn: Machine Learning in Python, Pedregosa et al., JMLR 12, pp. 2825-2830, 2011

Team Crazy Alvin

1. Background

Of the four ALS Stratification Prize4Life Challenge sub-challenges, our team dedicated their effort to sub-challenge one, whose goal was to predict 3-12 month ALSFRS slope using clinical trial data collected through the PRO-ACT database.
We elected to first cluster ALS patients with respect to their 0-3 month ALSFRS slope, which is an accepted measure of the disease's progression. Subsequently, encouraged by the relatively good performance of the Random Forest learner in the previous ALS challenge, published in Küffner et al. (2014), we independently fit a Random Forest regression model to each one of the generated clusters. It was hypothesized that this procedure would result in more specific and more precise predictions, since individual clusters are more homogenous than the full training dataset. Albeit, this hypothesis was not tested. Our choice of the Random Forest learner was also influenced by its ability to utilize mixed data (continuous and categorical). In addition to outcome predictions, Random Forest was also used for feature selection and data imputation. Features were selected based on their effect on the mean square error (MSE) in the case of regression (sub-challenge 1).
The clustering step is a novel component in our methodology which has been suggested in the challenge definition.

2. Methods

Subsequent to data preprocessing (described in the following section), our modelling procedure can be replicated by following these steps:

1. Patients were clustered via K-means clustering according to their [0-46] day and [46-92] day ALSFRS slopes. Patients missing slopes for either of these time-periods were placed in a generic cluster (cluster 0). A minimum size constraint of 50 patients was applied to a cluster number (K) selection algorithm that maximized average silhouette width (SW) of the clusters. The SW is an indicator of cluster quality, it ranges from 0 to 1. An average SW of less than or equal to 0.5 indicates poor clustering whereas an average SW greater than 0.5 indicates better clustering quality. Given our cluster size constraint, we got 6 clusters and an average silhouette width of 0.39, indicating poor overall cluster quality:
    - Cluster 1 contained 188 patients
    - Cluster 2 contained 271 patients
    - Cluster 3 contained 154 patients
    - Cluster 4 contained 56 patients
    - Cluster 5 contained 148 patients
    - Cluster 6 contained 57 patients
    - Cluster 0 contained 1584 patients

1. A design matrix was generated per cluster. For each design matrix, features with unknown values in 10% or more patients were eliminated. Random Forest was used to impute the remaining unknown values. Random Forest regression models were then trained on each of the clusters. The minimum terminal node size was constricted to 10 patients, to reduce training time. One thousand trees were grown per cluster. *mtry,* the number of randomly sampled features per node was tuned using the R Caret package via repeated (3x) 10-fold cross validation. The performance metrics of the tuned models are listed in Table 1. The purpose of this step was to rank the variables based on their importance (% increase in predicted MSE associated with permuting the variable values). Variables whose permutation resulted in relatively greater % increase in predicted MSE were regarded as more important. The top 10 most important variables per cluster are listed in Table 1. Please note that although not shown here, a tuned gradient boosting machine (GBM) model performed similarly to Random Forest with the exception of cluster 4, where GBM yielded a higher Pearson correlation than Random Forest (albeit with a similar standard deviation). Missing values were not imputed in GBM modelling.

Table 1: Variable Importance of Each Cluster
Query

Cluster	Top 10 Variables	% Increase in MSE	mtry	RMSE	c-index	Pearson coefficient	RMSE Standard Deviation (SD)	c-index SD	Pearson coefficient SD
1	last.value_._onset_delta	21	6	0.53	0.67	0.45	0.11	0.07	0.2
	last.value_._Calcium	9.3							
	mean.slope_._ALSFRS_Total	8.2							
	mean.slope_._Q1_Speech	7.9							

	last.value_._Gamma-glutamyl transferase	7.9							
	mean.slope_._mouth	6.5							
	mean.slope_._trunk	6.5							
	last.value_._Creatinine	5.5							
	last.value_._Phosphorus	4.8							
	last.value_._ALT(SGPT)	4.4							
2	last.value_._onset_delta	28.8	9	0.48	0.65	0.47	0.07	0.07	0.16
	mean.slope_._ALSFRS_Total	17.7							
	last.value_._Albumin	14.9							
	last.value_._Platelets	8							
	mean.slope_._hands	7.6							

	last.value_._Alkaline Phosphatase	6.7							
	last.value_._AST(SGOT)	6.4							
	mean.slope_._Q1_Speech	5.5							
	last.value_._Creatinine	4.9							
	last.value_._Gender	4.9							
3	last.value_._onset_delta	19.8	5	0.55	0.64	0.39	0.12	0.07	0.19
	mean.slope_._ALSFRS_Total	12.8							
	mean.slope_._mouth	12.6							
	mean.slope_._Q8_Walking	7.1							
	mean.slope_._Q6_Dressing_and_Hygiene	6.7							

1. In the selector script, a patient is first assigned to a cluster based on their distance from the cluster medoid. Subsequently, the available features of the patients are ranked with respect to their importance. The feature information is passed to the predictor script, which rebuilds a Random Forest regression model using only the most important features that are available and predicts patient outcome using the model. The prediction confidence score is arbitrarily assigned as 0.5. Note that the model is built using the cluster's training set data, not the new patient data.

2.1 Data Pre-Processing

The data pre-processing step proved significantly useful to remove features that have limited data and to aggregate feature values with multiple measurements. The process also fixes issues regarding unmatched feature units and simplifies the data by assuming the last or mean measurement. Each preprocessing file have annotations that describe their function and code structure in more detail. Below is a quick summary of the scripts and whether they were applied to the training models, testing set, or both.

Pre-Processing Scripts	Description	Training Model	Final Submission	
load_ALS_training	Reads all ALS data text files, and sets NA strings from 'NA' 'Unknown' and '-'.	YES	YES	
table_to_list	Transforms read-in table into list of lists: first level is the list of features; second level is a data frame with fields "SubjectID", "feature_value", "feature_unit", "feature_delta".	YES	YES	
get_deltas	Calculates slope for ALS time series data.	YES	YES	
merge_list_dfs	Merges list of data frames by "SubjectID".	YES	YES	
classify_features	Classify features according to their feature values: "non-informative", "bad_deltas", "numeric", "integer", "multiple_units", "factor". See script for more details.	YES	YES	
multiple_unit_curate	Manually curates features that have multiple feature units. Most are simply to fill in missing units, but others involve conversion between units. Any numeric values in combination with factor levels are set to NA.	YES	YES	
get_last	Grab last or mean measurement for each feature.	YES	YES	

find_outliers	Uses Grubbs' test to remove significant outliers.	YES	NO	
lab_test_preproc	Manually curate lab test data.	YES	NO	
rm_bad_features	Removes features that have "too many" NAs.	YES	NO	
main_preproc_script	Main preprocessing wrapper.	YES	YES -- encapsulated into selector and predictor	

The ProActProgression zip file contains the copies of scripts applied called to generate the training model, as well as those called to generate the submissions.

2.2 Compiling and Running Code

TRAINING

Executing the code will generate temporary .txt files that will be merged and can be deleted afterwards.

1. Unpack the ProActProgression zip file and use a clean directory that contains all the scripts in the ProActProgression/training_material folder.
2. Modify scripts to set the appropriate paths to input files.
    - Make sure load_ALS_training.R can load the data properly.
    - Ensure proper working directories in all scripts.
3. Run main_preproc_script.R to generate the refactored form-listed data (form.list.train.Rds) -- set variable "ready" to FALSE
    - FINAL OUTPUT: clusterModels.Rds, train_slope.Rds

TESTING

Execute the selector.sh and predictor.sh scripts as described in the challenge.

3. Conclusion/Discussion

Selecting the right features to use from the training data proved most difficult, as most of the feature values were missing and were marked NA. Most time measurements were unique between patients, which allowed for a simple slope calculation to predict feature values, while others only have a single time point. Furthermore, feature values range from numeric to factors, which can then be interpreted as all factors, or all numerics and NAs.

The quality of the clusters generated by the training model varied between clusters. Based on improvements in MSE, some clusters (1, 2, 3, and 5) showed the importance of at least the top 6 features, while others (4 and 6) limited this to only a few (< 6) defining features. This highlights the importance of classifying new patients into the right clusters. Moreover, it is interesting to note "onset_delta" being the most defining feature in all the clusters, except for cluster 5 where it appeared as the 7th most important.
Overall, the model performed on average with what was expected. Due to the complex and refined preprocessing performed on the training set, one bottleneck of the algorithm lies in running the same (and slightly modified) preprocessing steps to the input patient records, so they can be applicable to the training model in making predictions.
Improvements to the data preprocessing include better curation of the data, both qualitative and quantitative.

References

1. Küffner, R. *et al*. Crowdsourced analysis of clinical trial data to predict amyotrophic lateral sclerosis progression. *Nat Biotechnol*. **33(1)** 2015.

4. Authors Statement

Rached Alkallas Ontario Institute for Cancer Research
Shadrielle Melijah G. Espiritu Ontario Institute for Cancer Research
Julia Hopkins -- Ontario Institute for Cancer Research

Team Veristat

Title: A Bayesian Tree Model for Predicting the ALSFRS Slope using the PROACT dataset

Authors: John Balser, Barbara Balser, Susan Paadre, Robin Bliss, Zhiqing Xu, Li Zhang, Bhavna Ahuja Nicole Corriveau, Adrienne O'Donnell, Jialu Cai, Eve Desplats, Jean-Karl Sirois, Jarrett Lowe

Affiliation: Veristat, Inc.

We agree to make our submission public as part of the challenge archive.

ABSTRACT: Our method applies a Bayesian regression tree model to predict 3-12 month ALSFRS slope using clinical trial data collected through the PRO-ACT database.

INTRODUCTION:

We propose a Bayesian regression tree model to predict 3-12 month ALSFRS slope using 0-3 month ALSFRS slope as well as other clinical trial data collected through the PROACT database for our solution to the ALS challenge. We selected Bayesian regression trees as our final analysis and prediction method because of its nonparametric flexibility and the intrinsic use of observed "training" data to generate a posterior distribution through which we can predict the "testing" or "validation" data.

In our analysis we evaluated several methods for prediction, including frequentist regression tree models, factor analysis, and Bayesian regression tree models. While frequentist regression trees and factor analysis provided us with great information and aided our variable selection process, our final model uses the Bayesian regression tree. During our variable selection and model building, we compared and contrasted the output from the three methods. We found that factor analysis did not provide precise enough prediction for our purposes. When comparing frequentist and Bayesian regression trees, the Bayesian models provided higher pseudo-R2 values and better goodness-of-fit than the frequentist method.

For variable selection, we used multiple approaches to identify potential variables representing baseline and 0-3 month characteristics that may predict the ALSFRS Slope from 3 to 12 months. We ranked the variables through a variety of methods, including Pearson correlation analysis, factor analysis, and clinical judgement, including recommendations from medical professionals and the creation of composite and summary variables from assessments that were performed multiple times within the 0-3 month period. We then performed forward and backward model selection methods for linear regression models, frequentist regression trees, and Bayesian regression trees.

Our general method was to cast a wide net for variable selection, and to integrate the results of various approaches and see which variables were repeatedly identified as possible predictor variables using the different approaches. The novelty of our methods is the interactive and iterative collaboration for variable and model selection through a variety of analysis methods. Through this iterative and multifaceted process, we identified the key variables from the PROACT database that we believe best predict ALSFRS slope: ALS onset site, time since ALS onset, diastolic blood pressure, forced vital capacity (FVC), Riluzole use and creatinine level.

Analyses were performed using SAS v9.3 and R v3.2.2.

METHODS:

Note: Summary information is available in Table 1 and Table 2.

OVERVIEW:

We performed Pearson correlation tests to observe the correlations of various variables to each other and the 3-12 month ALSFRS slope. Variables for consideration included demographic characteristics, laboratory parameters, vital signs, and ALS assessments including the ALSFRS slope from 0-3 months, time since onset, and onset site. We also performed factor analysis to both cluster variables together and to identify which variables were independent from one another in predicting ALSFRS slope. Through this process we reduced the number of variables which would be considered as possible contributors in the subsequent analysis. After identifying a reduced set of variables for prediction, we performed forward and backward stepwise multiple linear regression, frequentist

regression tree models, and Bayesian regression tree models to further reduce the variables selected. The final model we propose is a Bayesian regression tree.

DATA PROCESSING:

For variables measured multiple times during the 0-3 month period, we computed the mean, median, and range by patient for possible inclusion in the models. We also selected the last value of each variable during the 0-3 month time period as a possible variable for consideration. For selected laboratory parameters we also computed ratios for consideration that may be meaningful based on clinical importance. Such ratios included:

- systolic blood pressure (SBP)/diastolic blood pressure (DBP);
- last force volume capacity (FVC) measurement/mean FVC measurement;
- respiratory rate/FVC; creatinine/alkaline phosphatase;
- creatinine/CK;
- calcium/sodium;
- creatinine/blood urea nitrogen;
- CK/blood urea nitrogen (BUN);
- calcium/phosphorous

In the training data, in order to increase the completeness of the data, when FVC was missing we imputed the value for slow volume capacity (SVC) in its place.

Variables were considered for model selection if at least 80% of patients had at least one measurement available in the 0-3 month time period.

SUMMARIES:

We produced summary descriptive statistics for all variables recorded during the 0-3 month time period. For categorical variables we calculated the number and percentage of patients in each category. For continuous features, the number of patients, mean, median, standard deviation, minimum, and maximum values were calculated.

VARIABLE SELECTION

- Pearson Correlation Analysis Variable Selection:

- We performed a Pearson correlation analysis to identify possible variables for inclusion in the final models. The analysis was performed in two ways:

1. We computed the Pearson correlation coefficient for all variables under consideration with ALSFRS slope.
2. We computed the Pearson correlation coefficient between all variables under consideration.

Variables found to be correlated with the ALSFRS slope with p-value < 0.05 included:

- ALS onset site,
- time since ALS onset,
- last value of pulse,
- mean pulse
- mean DBP,
- last value FVC,
- mean FVC,
- slope of FVC,
- ratio of respiratory rate/FVC,
- last value of calcium,
- mean calcium,
- last value of phosphorus,
- ratio of calcium/phosphorous,
- last value of total bilirubin,
- mean total bilirubin,
- last value gamma glutamyltransferase (GGT),
- mean GGT,
- assigned treatment group,
- Riluzole use

- Factor Analysis Variable Selection:
- We performed a factor analysis where we loaded variables with at least 80% completion (as defined above) into the model. The model resulted in a total of thirteen factors (see Table 1). Factor 1 was most highly associated with ALSFRS slope, followed by Factor 2, etc. We used the results of the correlation analysis in conjunction with the thirteen identified factors to identify one or two variables per factor to move forward to the model selection process. Variables were selected for continuation if they loaded highly to a single factor, were correlated with ALSFRS, and had low correlation with the other variables selected. Through

this process we aimed to identify variables that were linearly independent of one another while maintaining correlation with ALSFRS.

Variables selected from the Pearson correlation and factor analyses for inclusion in the multiple regression analysis included:

- time since ALS onset,
- ALS onset site,
- ratio of last value of pulse/mean pulse,
- ratio of last value DBP/mean DBP,
- ratio of last value FVC/mean of FVC,
- slope of FVC,
- ratio of last value of chloride/mean chloride,
- ratio of last value of creatine kinase/mean value of creatine kinase,
- mean phosphorus,
- treatment assignment,
- Riluzole use

- Multiple Regression Variable Selection:
- Our next step was to apply a model selection process using forward and backward stepwise multiple linear regression using the predictors identified from the previous selection processes. Both forward and backward selection yielded the same results. Based on the restrictions imposed by the rules of the Challenge, six features were selected, including: time since onset, onset site, ratio of last value FVC/mean FVC, ratio of last value DBP/DBP mean, phosphorus mean, and Riluzole use. The R2 for the model is 0.1816.

Out of these 6 features, phosphorus had a high percent of missing data in the validation data and so we removed it from the model. Excluding the variable phosphorus from the model on the training data, R2= 0.1663.

We tried the same model on the validation dataset, R2 =0.2125.

- Bayesian Tree Variable Selection:
- To apply Bayesian regression trees we used the R package bartMachine. We performed a model selection process again, this time using the Bayesian regression tree. The figure below displays the proportion of trees that included each of the proposed predictor variables. Those with black dots were identified by the modelling as likely important predictors.

We found the following variables were strongest predictors of ALSFRS: time since onset, onset site, last value FVC, last value creatinine, Riluzole, exposure to active treatment, and gender.

Final Model:

- To identify the final model we compared the lists of variables selected from each of the model selection methods. We selected five variables that appeared in one or more of the variable selection procedures as highly correlated with the outcome ALSFRS to include in the final model. The model includes: time since onset, onset site, last value FVC, last value creatinine, and use of Riluzole. We also include the 0-3 month ALSFRS slope as an independent predictor of 3-12 month ALSFRS slope.

When applied to the validation data, we found that the onset site posed issues as there were different responses (including an "other" field) than were included in the training data. As a result, during final submission we removed onset site from the model.

The Bayesian regression tree model was applied to the training data and the resulting goodness-of-fit was as follows: pseudo-R2=0.2321

- Missing Data
- In the validation data, we imputed missing values with information from the training dataset to provide complete cases and make prediction possible. If onset site was missing, the site was assumed to be the Limb. If Riluzole exposure was missing, we assumed patients were not exposed to Riluzole. Otherwise, for continuous variables, if the value was missing we imputed the mean value from the training data.

CONCLUSION /DISCUSSION:

Our final model is a Bayesian Tree model in which the 3-12 month ALSFRS Slope is predicted by time since onset, ALSFRS slope during 0-3 month period, last value for FVC during 0-3 month period, last value for the creatinine during 0-3 month point, and Riluzole use.

The measurements we included in our final model are commonly assessed for ALS patients and are non-invasive. While we prefer the simplicity of the proposed model, we also acknowledge that we have not considered particular subcategories or strata for patients. While our prediction model may be robust overall, it may be limited in some applications as all patients are predicted from the same model. In addition, while the prediction may be accurate, the Bayesian background may make the model more difficult for clinicians to describe to patients if it is used to predict risk.

AUTHORS STATEMENT:

John Balser provided statistical consulting to the team. Barbara Balser provided medical consulting to the team. Robin Bliss advised the brainstorming and model development throughout Veristat's work on ALS Challenge, performed final coding and document review. Susan Paadre contributed by assisting in team development, brainstorming, organization, and documentation. Li Zhang conceived the study, purified the datasets, contributed to establishing the prediction model, contributed to refining the prediction model, supervised the analysis, and proof-read the manuscript. Adrienne O'Donnell assisted in finalizing the model and establishing prediction variables. Bhavna Ahuja assisted in the correlation and factor analysis and wrote the manuscript. Eve Desplats assisted in data manipulation, factor analysis (R packages), and Bayesian regression tree modelling. Zhiqing Xu contributed by downloading the ALS challenge data, performing analyses to establish prediction variables, and by providing code and documentation for Bayesian regression tree modeling. Jean-Karl Sirois contributed by providing initial data explorations in R to establish predictor variables and by providing code to implement regression tree modeling. Jarrett Lowe was responsible for organization of internal team meetings and summarizing critical next steps and action items for all team members. Jialu Cai helped with meeting note taking and collaborating during brainstorming. Nicole Corriveau helped in brainstorming and organization.

REFERENCES:

1. Peter Dalgaard, Introductory Statistics with R
2. W. N. Venables, D. M. Smith and the R Core Team: An introduction to R
3. Norman Matloff: The art of R programming
4. A Czaplinski, A A Yen, S H Appel: Forced vital capacity (FVC) as an indicator of survival and disease progression in an ALS clinic population, J Neurology Neurosurgery and Psychiatry 2006;77: 390–392.
5. Merit E Cudkowicz, Sarah Titus, Marianne Kearney, Hong Yu, Alexander Sherman, David Schoenfeld, Douglas Hayden, Amy Shui, Benjamin Brooks, Robin Conwit, Donna Felsenstein, David J Greenblatt, Myles Keroack, John T Kissel, Robert Miller, Jeff rey Rosenfeld, Jeff rey D Rothstein,Ericka Simpson, Nina Tolkoff -Rubin, Lorne Zinman, Jeremy M Shefner, for the Ceftriaxone Study Investigators: Safety and efficacy of ceftriaxone for amyotrophic lateral sclerosis: a multi-stage, randomised, double-blind, placebo-controlled trial, www.lancet.com/neurology/ Vol12 November 2014 1083 – 1091
6. Robert Küffner, Neta Zach, Raquel Norel, Johann Hawe, David Schoenfeld, Liuxia Wang, Guang Li, Lilly Fang, Lester Mackey, Orla Hardiman, Merit Cudkowicz, Alexander Sherman, Gokhan Ertaylan, Moritz Grosse-Wentrup, Torsten Hothorn, Jules van Ligtenberg, Jakob H Macke, Timm Meyer, Bernhard Schölkopf, Linh Tran, Rubio Vaughan, Gustavo Stolovitzky, Melanie L Leitner :

Crowdsourced analysis of clinical trial data to predict amyotrophic lateral sclerosis progression, Nature Biotechnology 33, 51-59 (2015)

1. James. D. Berry, Robert Mille, Dan. H. Moore, Merit. E. Cudkowicz, Leonard. H. Van Den Berg, Douglas A. Kerr, Yingwen Dong, Evan . W. Ingersoll, Donald Archibald: The Combined Assessment of Function and Survival (CAFS): A new endpoint for ALS clinical trials, Informa Healthcare
2. Merit E Cudkowicz, Leonard H van den Berg, Jeremy M Shefner, Hiroshi Mitsumoto, Jesus S Mora, Albert Ludolph, Orla Hardiman, Michael E Bozik, Evan W Ingersoll, Donald Archibald, Adam L Meyers, Yingwen Dong, Wildon R Farwell, Douglas A Kerr, for the EMPOWER investigators: Dexpramipexole versus placebo for patients with amyotrophic lateral sclerosis (EMPOWER): a randomised, double-blind, phase 3 trial, www.lancet.com/neurology/ Vol12 November 2013 1059 – 1067
3. S. Abrahams, T. H. Bak: Edinburgh Cognitive and Behavioral ALS Screen – ECAS English Version (2013)
4. Dianne M. Finkelstein, David A Schoenfeld: Combining Mortality and Longitudinal Measures in Clinical Trials, Statistics in Medicine 18, 1999: 1341 -1354
5. Justin Bleich, Adam Kapelner, Edward. I. George, Shane. T. Jensen: Variable selection for BART: An application to Gene Regulation: The Annals of Applied Statistics, Vol 8, 3 March 2014 (1750 -1781)

ACKNOWLEDGEMENTS: We would like to thank Veristat for providing the support and the necessary resources to complete the challenge.
